# Supramolecular dye polymers for aggregation-induced photocatalysis

**DOI:** 10.1038/s41557-026-02151-4

**Published:** 2026-05-18

**Authors:** Marianna Barbieri, David Cappelletti, Luca Vaccarin, Marco Villa, Luca Catalano, Khai-Nghi Truong, Fabrizio Nestola, Elisa Pelorosso, Alessandro Aliprandi, Andrea Sartorel, Paola Ceroni, Francesca Arcudi, Luka Ðorđević

**Affiliations:** 1 Department of Chemical Sciences, University of Padova, Padova, Italy; 2 Department of Chemistry ‘Giacomo Ciamician’, Alma Mater Studiorum−University of Bologna, Bologna, Italy; 3 Department of Life Sciences, University of Modena and Reggio Emilia, Modena, Italy; 4 Rigaku Europe SE, Neu-Isenburg, Germany; 5 Department of Geosciences, University of Padova, Padova, Italy

**Keywords:** Self-assembly, Molecular self-assembly, Crystal engineering, Photocatalysis

## Abstract

Aggregation can profoundly alter the excited-state properties of organic chromophores; however, crystalline supramolecular polymers are often targeted for photocatalytic conversion of solar energy due to favourable charge delocalization or exciton transport. Here we exploit aggregation as a strategy for organic chromophore rigidification to activate photocatalysis by stabilizing localized excited states. Using amphiphilic distyrylanthracene derivatives, we show that aggregation in water enhances the availability of excited states, enabling light-driven transformations of solar energy into storable fuels. We found enhanced reactivity to correlate with increased local excited-state population, underlining the role of self-assembly in restricting intramolecular motion and suppressing unproductive non-radiative decay. We observed photocatalysis to be maximized in kinetically trapped aggregates, outperforming their thermodynamic counterparts, to challenge conventional paradigm that favourable activities require extended assemblies. By achieving excited-state confinement and reactivity instead of charge delocalization, this work reports aggregation-induced photocatalysis as a strategy for preparing photostable, emissive and functional organic photocatalysts in water.

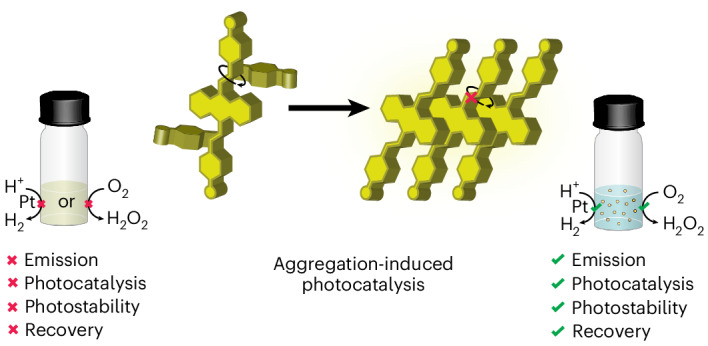

## Abstract (web summary)

Aggregation usually quenches the excited states of organic dyes, limiting photocatalysis. Now it has been shown that supramolecular aggregation of amphiphilic chromophores can rigidify the molecules, stabilize localized reactive excited states and enable light-driven H_2_ and H_2_O_2_ production in water, establishing aggregation-induced photocatalysis as a general design principle.

## Main

The direct conversion of sunlight into chemical energy by organic molecular materials remains a central objective in artificial photosynthesis^1–5^. Organic molecules are versatile photocatalysts because they offer structural tunability, high extinction coefficients and rational molecular engineering. They have traditionally been utilized either as molecular dyes in homogeneous solution^6–9^ or as semiconductors in heterogeneous systems^10–13^. Although molecular systems offer defined excited-state redox potentials and facile processing, their use is often limited by poor photostability and low photocatalytic efficiencies. Conversely, organic semiconductors benefit from delocalized charge transport and robustness but often sacrifice molecular precision and processability.

A major challenge in leveraging organic dyes for light-driven catalysis is the loss of reactivity upon aggregation. In many cases, chromophore–chromophore interactions can quench excited states through trap states, excimer formation, intermolecular charge or electron transfer, intersystem crossing and H-aggregate formation^14,15^. As a result, the excited states are typically deactivated quickly in the aggregated state. Strategies to overcome this and exploit molecules in photocatalysis include preparation of metallacages^16^, macrocycles^17^, using surfactants^18^, formation of inclusion complexes^19^ and co-assembling dyes with insulating (macro)molecules^20–23^.

Although aggregation is often detrimental to photocatalysis, it can also be beneficial for some organic dyes^12,24–27^. Inspired by biological photosystems, supramolecular assemblies have been used to enhance photocatalysis through exciton delocalization and long-range charge separation. In these systems, aggregation extends excited states across planar or rigid chromophores through *π*–*π* stacking or donor–acceptor interactions in crystalline assemblies^1–5,28–35^. In these systems, aggregation-caused emission quenching can enable exciton migration and charge separation across crystalline domains, thereby allowing organic nanostructures to drive reactions such as H_2_ evolution and H_2_O_2_ production. However, this strategy depends on strong electronic coupling between chromophores and thus often requires planar scaffolds and specific packing motifs that are difficult to predict and design rationally.

**Fig. 1:**
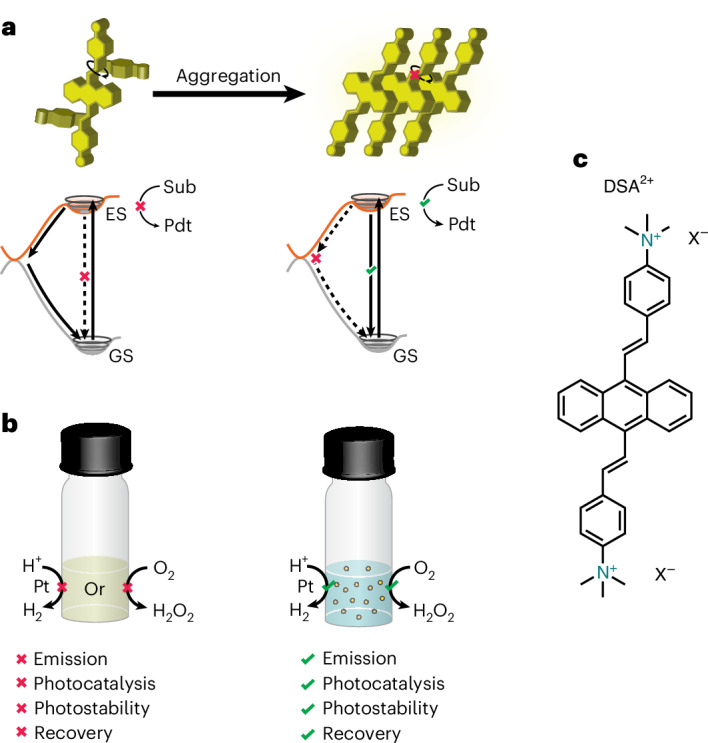
AIP with the DSA^2+^ supramolecular dye polymers. **a**, Overview of the rigidification in DSA^2+^ amphiphiles through supramolecular polymerization, obstructing otherwise rapid non-radiative decay of the photoexcited state. **b**, Aggregation leads to excited-states enhancement and photocatalysis (H_2_ or H_2_O_2_ photoproduction), with improved dye properties such as stability and recovery. **c**, Chemical structure of distyrylanthracene (DSA^2+^) amphiphile, where X^−^ indicates the counteranion. GS, ground state; ES, excited state; Sub, substrate; Pdt, product.

Here, we report aggregation-induced photocatalysis (AIP), a mechanism for organic dye photocatalysis that does not rely on solubilized chromophores or exciton-delocalized assemblies. Instead, molecular packing restricts intramolecular motion and increases the population of reactive excited states (Fig. 1a,b), paralleling the rigidification mechanism of aggregation-induced emission (AIE)^36–43^. Using an amphiphilic distyrylanthracene dye (DSA^2+^, Fig. 1c), we show that aggregation can be tuned by concentration, counterion identity or salt addition to form nanocrystals in water that drive photocatalytic H_2_ and H_2_O_2_ production with enhanced photostability. In contrast to self-assembled photocatalysts that rely on charge delocalization, these materials operate through localized excited states enabled by rigidification. The photocatalytic performance further depends on the aggregation pathway and extends to a structurally unrelated fluorophore scaffold.

## Results and discussion

### Photophysical and morphological properties of dye aggregates

The DSA^2+^ amphiphile has a distyrylanthracene core bearing two trimethylammonium groups (Fig. 1c). It was synthesized as diiodide salt (DSAI), and counterion exchange reactions afforded DSAX compounds as chloride (DSACl), bromide (DSABr), nitrate (DSANO_3_) and hexafluorophosphate (DSAPF_6_) salts (Supplementary Information section 3). The molecules were characterized by ^1^H and ^13^C nuclear magnetic resonance spectroscopies, mass spectrometry, ultraviolet (UV)–visible absorption spectroscopy and infrared spectroscopy (Supplementary Figs. 1–32).

**Fig. 2:**
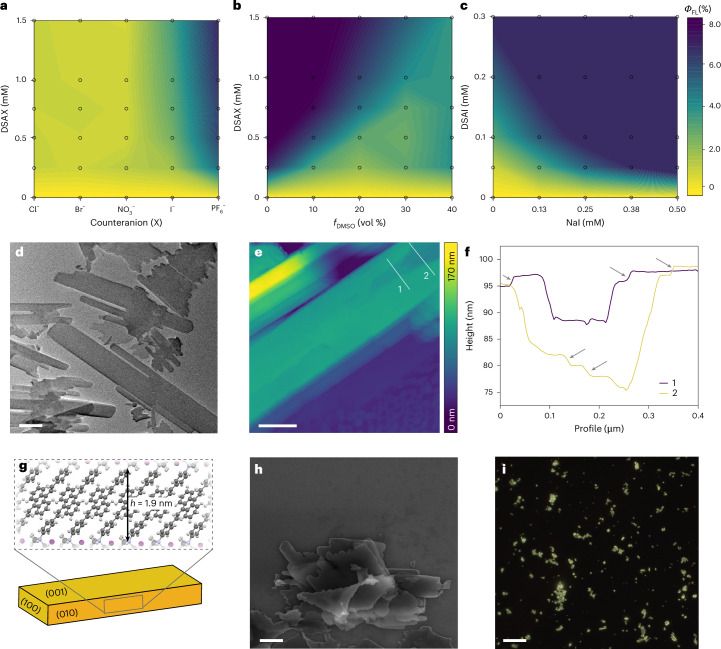
Supramolecular polymerization of DSA^2+^ amphiphiles into fluorescent and crystalline nanostructures. **a**, Assembly landscapes at different concentrations of DSA^2+^ amphiphile (mM) with selected counteranions (Cl^−^, Br^−^, NO_3_^−^, I^−^ and PF_6_^−^). **b**, Assembly landscape at different concentrations of DSAI amphiphile (mM) under various ratios between water and organic solvent (DMSO). **c**, Assembly landscape at different concentrations of DSAI amphiphile (mM) with different amount of salt (NaI, mM). **d**, Transmission electron microscope (TEM) image of DSAI supramolecular nanostructures in water (DSAI 0.05 mM, NaI 0.5 mM). Scale bar, 500 nm. **e**, Atomic force microscope image of drop-casted aqueous suspension of DSAI supramolecular nanostructures (DSAI 0.05 mM, NaI 0.5 mM). Scale bar, 400 nm. **f**, Height profiles by AFM of selected sections (indicated by 1 and 2 from **e**) and relative step heights of ∼2 nm (grey arrows). **g**, A schematic representation of the dominant crystallographic faces of a DSAI nanocrystal, with a zoom-in on the layered slipped *π*–*π* stacking viewed along the *b* axis, as determined from 3D-ED data (atoms shown as ball and stick). **h**, Scanning electron microscope image of DSAI supramolecular nanostructures in water (DSAI 0.05 mM, NaI 0.5 mM). Scale bar, 400 nm. **i**, WFM image of DSAI supramolecular nanostructures in water (DSAI 0.05 mM, NaI 0.5 mM). Scale bar, 20 μm. Source data

We first measured the absolute fluorescence quantum yields (*Φ*_FL_) of DSAX salts in water (0–1.5 mM) to monitor the aggregation, as *Φ*_FL_ is a sensitive reporter of restricted intramolecular rotation in the aggregate state and thus of AIE^44,45^. DSAX samples with hydrated counteranions, such as Cl^−^, Br^−^ and NO_3_^−^, showed no aggregation, as indicated by low fluorescence (*Φ*_FL_ ≤ 1.1%), whereas hydrophobic PF_6_^−^ caused the molecules to be always aggregated, as evidenced by relative high *Φ*_FL_ ≈ 6.9% in these suspensions (Fig. 2a and Supplementary Figs. 33 and 34). Among the DSAX salts, only the iodide form (DSAI) exhibited concentration-dependent aggregation and emission behaviour, from weakly emissive dilute solutions to progressively fluorescent yellow suspensions at higher concentrations (Fig. 2a and Supplementary Figs. 35–38). This mirrors previous observations that higher concentrations of amphiphilic monomers provide self-screening for repulsive electrostatic interactions^46^. The specific ion effect is opposite to the conventional Hofmeister series in which charge-dense ions (for example, Cl^−^, Br^−^ and NO_3_^−^) promote aggregation of macromolecules by reducing solubility^47,48^. Here, instead, charge-diffuse ions (I^−^ and PF_6_^−^) promote aggregation, consistent with a reverse Hofmeister effect driven by ion pairing and monomers stacking.

We further investigated the polymerization behaviour of DSAI, which can exist as either dissolved monomers or aggregates depending on concentration. Organic solvent minimizes interactions between monomers and promotes disaggregation through dissolution, whereas salts and electrolytes enhance charge screening and thus promote supramolecular polymerization. Hence, plotting *Φ*_FL_ as a function of [DSAI] and either fraction of organic solvent (*f*_DMSO_, as vol% with water) or [NaI] provide assembly landscapes that visualize the propensity of DSAI to self-assemble under different conditions (Fig. 2b,c and Supplementary Figs. 40–43). In both cases, aggregation is promoted either by increasing the water content, which strengthens hydrophobic interactions or by increasing [NaI], which screens the positive charges, reduces electrostatic repulsion and promotes hydrophobic collapse. Consistent with this mechanism, aggregation of DSAX (X = Cl^−^, Br^−^, NO_3_^−^ and I^−^) can be induced by addition of the corresponding sodium salts (NaX), which increase charge screening and promote aggregate formation (Supplementary Figs. 44–46). Aggregation can also be triggered by salts bearing counteranions different from those originally paired with the dye; for example, NaI induces aggregation of DSACl (Supplementary Figs. 47 and 48), showing that both ionic strength and counteranion identity contribute. Supporting this interpretation, counteranions are not merely dissociated but are embedded within the nanostructures (Supplementary Figs. 48–54).

We next characterized the nanostructures formed upon DSAI self-assembly using complementary techniques from the nanoscale to the bulk. Transmission electron microscopy revealed flat, elongated nanoribbons with high aspect ratio (Fig. 2d and Supplementary Fig. 55), indicating a preferential growth direction, probably via hydrophobic collapse of the aromatic cores. Atomic force microscopy showed that these flat nanoribbons are stacked lamellar structures with discrete step heights of ~2 nm (Fig. 2e,f and Supplementary Fig. 56). X-ray diffraction on the bulk powder revealed the high degree of crystallinity of the nanoribbons (Supplementary Fig. 57). Although bulk single crystals of pure DSAI suitable for X-ray analysis could not be obtained in water, continuous three-dimensional electron diffraction (3D-ED)^49,50^ provided atomically precise structural characterization of the nanocrystalline aggregates. 3D-ED analysis revealed face-on molecular packing in the aggregates (Fig. 2g and Supplementary Figs. 58–60), in which the DSAI chromophores adopt a typical slipped *π*–*π* stacked arrangement (*θ*_slip_ ≈ 44.6°, centre-to-centre distance of ~5.7 Å) with an interlayer spacing of ~1.9 nm typical for crystalline aromatic systems such as acenes and related polycyclic *π*-conjugated molecules. Scanning electron microscopy (SEM) showed that these nanoribbons assemble both laterally and through inter-ribbon stacking into micron-sized crystalline platelets (Fig. 2h and Supplementary Fig. 61), whereas widefield fluorescence microscopy (WFM) revealed bright emissive micron-sized domains (Fig. 2i and Supplementary Fig. 62).

Supramolecular aggregation can be systematically controlled by counterion, solvent environment and charge screening to yield structurally ordered, emissive nanoribbons, establishing the structural basis for the photocatalytic studies that follow.

### Aggregation-induced light-driven ROS and H_2_O_2_ generation

**Fig. 3:**
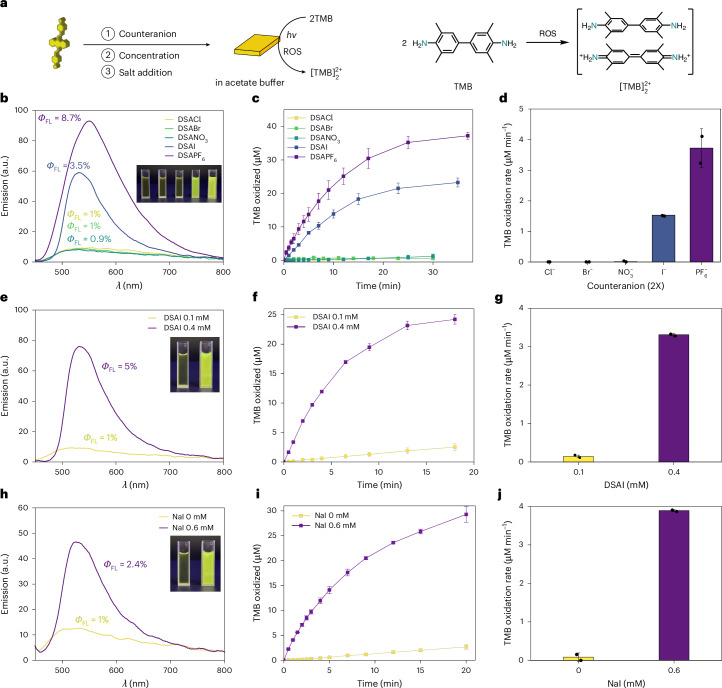
Light-driven ROS generation by DSA^2+^ nanostructures. **a**, A schematic representation of aggregation modes (counteranion used, concentration and ionic strength) to obtain fluorescent nanostructures that mediate oxidation of the TMB probe into a blue charge-transfer complex (white-light LED, 180 mW cm^−2^ under air). **b**, Fluorescence emission spectra and fluorescence quantum yields (*Φ*_FL_) of DSAX (0.3 mM) with different counteranions (X = Cl^−^, Br^−^, NO_3_^−^, I^−^ and PF_6_^−^). The inset shows the DSAX samples under UV light (from left to right: Cl^−^, Br^−^, NO_3_^−^, I^−^ and PF_6_^−^). **c**, Light-driven TMB (134 μM) oxidation by DSAX over time. Statistics are from two independent groups. **d**, TMB oxidation rate (0.0 ± 0, 0.0 ± 0, 0.01 ± 0.02, 1.5 ± 0 and 3.7 ± 0.6 μM min^−1^) extracted from traces in **c**. Statistics are from two independent groups. **e**, Fluorescence emission spectra and fluorescence quantum yields (*Φ*_FL_) of DSAI at 0.1 mM concentration (yellow trace) and 0.4 mM (purple trace). The inset shows DSAI 0.1 mM (left) and 0.4 mM (right) samples under UV light. **f**, Light-driven TMB (134 μM) oxidation by DSAI over time. Statistics are from two independent groups. **g**, TMB oxidation rate (0.14 ± 0.04 and 3.3 ± 0 μM min^−1^) extracted from traces in **f**. Statistics are from two independent groups. **h**, Fluorescence emission spectra and fluorescence quantum yield (*Φ*_FL_) of DSAI (0.1 mM) without (yellow trace, 0.0 mM NaI) and with presence of salt (purple trace, 0.6 mM NaI). The inset shows DSAI 0.1 mM samples without (left) and with NaI present (right) under UV light. **i**, Light-driven TMB (134 μM) oxidation by DSAI without and with presence of NaI over time. Statistics are from two independent groups. **j**, TMB oxidation rate (0.08 ± 0.11 and 3.9 ± 0 μM min^−1^) extracted from traces in **i**. Statistics are from two independent groups. Source data

We next investigated whether supramolecular assembly of DSA^2+^ improves light-driven generation of reactive oxygen species (ROS), modulating aggregation through counterion exchange, chromophore concentration and ionic strength (Fig. 3a). In each case, we assessed both fluorescence quantum yield (*Φ*_FL_) and light-driven ROS generation, the latter using the 3,3′,5,5′-tetramethylbenzidine (TMB)^51,52^ probe, which is oxidized by ROS to form a blue-coloured charge-transfer complex (Fig. 3a).

Under the buffer conditions used for the light-driven TMB oxidation (acetate buffer, 0.1 M, pH 5), hydrophobic and poorly hydrated anions, particularly I^−^ and PF_6_^−^, increased *Φ*_FL_ from ≤1.1% (for Cl^−^, Br^−^ and NO_3_^−^ salts) to 3.5% and 8.7% for DSAI and DSAPF_6_, respectively (Fig. 3b). This fluorescence enhancement strongly correlates with photocatalytic activity under white-light irradiation: DSAPF_6_ exhibited the highest rate of TMB oxidation (*v*_0_ = 3.7 μM min^−1^), followed by I^−^ (*v*_0_ = 1.5 μM min^−1^), whereas Cl^−^, Br^−^ and NO_3_^−^ showed no or minimal activity (Fig. 3c,d and Supplementary Fig. 63). This specific ion effect trend follows a reverse Hofmeister effect already observed in unbuffered water, suggesting that charge-diffuse anions reduce solvation and enable greater excited-states utilization in photocatalysis. Increasing DSAI from 0.1 mM to 0.4 mM in acetate buffer increased *Φ*_FL_ about fivefold, from 1.1% to 4.8% (Fig. 3e), consistent with AIE. Correspondingly, the rate of TMB oxidation increases by more than one order of magnitude, from *v*_0_ = 0.14 μM min^−1^ to *v*_0_ = 3.3 μM min^−1^ (Fig. 3f,g and Supplementary Fig. 64), confirming that concentration-driven self-assembly translates into enhanced ROS generation. Salt-induced aggregation was probed by adding NaI to a dilute DSAI solution in acetate buffer. Even at constant chromophore concentration (0.1 mM), the presence of NaI (0.6 mM) increased *Φ*_FL_ from 1.1% to 2.4% (Fig. 3h) and the ROS production rate from *v*_0_ = 0.08 μM min^−1^ to *v*_0_ = 3.9 μM min^−1^ (Fig. 3i,j and Supplementary Fig. 65). These results highlight the role of electrostatic screening in driving aggregation and suggest that subtle environmental changes can modulate functional output via supramolecular polymerization.

To assess how supramolecular aggregation affects the excited states, we measured fluorescence lifetimes (*τ*_avg_) of solution and aggregates by time-correlated single photon counting. In all cases, the nanosecond fluorescence decays (Supplementary Figs. 66–70) indicate singlet excited states and motivated further femtosecond and nanosecond transient absorption measurements (TAS). In solution, the monomeric chromophore displayed a transient band at 620 nm that shifted to 545 nm and then decayed with lifetimes of 17 and 79 ps, respectively, consistent with rapid styryl photoisomerization in DSAI (Supplementary Fig. 71) and reported femtosecond transient absorption measurements spectra of 9,10-distyrylanthracene derivatives^53^. By contrast, DSAI aggregates showed no shift of the excited-state absorption bands (Supplementary Fig. 72). Kinetic analysis identified a fast 21-ps component, similar to the monomer, and a slower 320-ps component, as expected for rigidification-induced suppression of photoisomerization decay and enhanced excited-state stabilization. At 7 ns, the excited-state absorption band at 539 nm was still present, consistent with the lowest triplet excited state; this was confirmed by ns-TAS (Supplementary Fig. 73), although no phosphorescence was observed in the solid state at 77 K (Supplementary Fig. 74). The emission and transient absorption spectra of the aggregates also resemble those of the monomer in rigid media^53^ (Supplementary Fig. 75), supporting the formation of localized singlet and triplet excited states in the aggregated state. These results show that aggregation enhances photocatalysis by increasing the population of localized excited states. Although weak coupling interactions in slip-stacked molecules cannot be excluded, the large *π*–*π* stacking distances (> 5 Å), the absence of notable chromic shifts between solution and aggregated states, and the pump-probe data collectively indicate that DSAI aggregates operate through localized singlet and triplet excited states.

**Fig. 4:**
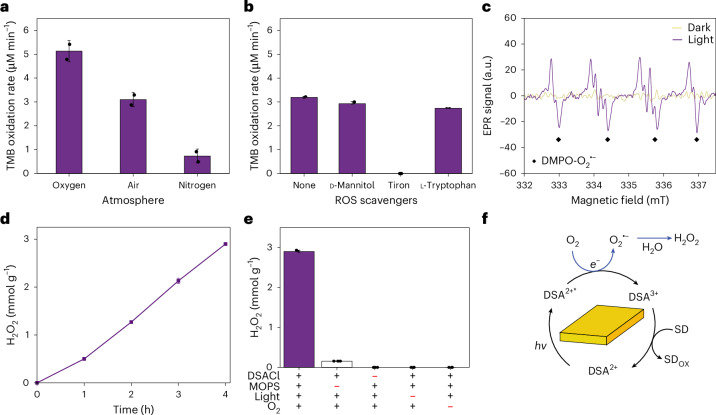
Mechanistic insights into ROS generation by irradiation of DSA^2+^ nanostructures. **a**, Light-driven oxidation rate (5.1 ± 0.4, 3.1 ± 0.3 and 0.73 ± 0.29 μM min^−1^) of TMB probe (134 μM) by DSAI nanostructures (DSAI 0.3 mM) with white light (LED, 180 mW cm^−2^) under different atmospheres (O_2_, air or N_2_). Statistics are from two independent groups. **b**, Light-driven oxidation rate (3.2 ± 0, 2.9 ± 0.1, 0.0 ± 0 and 2.8 ± 0 μM min^−1^) of TMB probe (134 μM) by DSAI nanostructures (DSAI 0.3 mM) with white light (LED, 180 mW cm^−2^) in presence of ROS scavengers. Statistics are from two independent groups. **c**, In situ EPR spectra for DSAI nanostructures (DSAI 0.5 mM) and DMPO trap in EDTA (0.1 M, pH 6) under dark and irradiation conditions. **d**, H_2_O_2_ light-driven production over time by irradiation of DSACl nanostructures (DSACl 0.5 mM, NaCl 0.9 M) in MOPS (0.1 M, pH 7.0) with 415 nm (LED, 140 mW cm^−2^). Statistics are from three independent groups. **e**, Control experiments for H_2_O_2_ produced by DSACl aggregates (2.9 ± 0, 0.15 ± 0, 0.0 ± 0, 0.0 ± 0 and 0.0 ± 0 mmol g^−1^). Statistics are from three independent groups. **f**, Proposed photocatalytic mechanism for H_2_O_2_ production by the DSA^2+^ nanostructures (SD is the sacrificial donor, specifically MOPS in this case). Source data

We then investigated the mechanism underlying this aggregation-induced ROS production. Using TMB oxidation as a readout, we evaluated the role of molecular oxygen. The oxidation rate was highest under pure O_2_ atmosphere, decreased under air, and was nearly abolished under N_2_, confirming that the photocatalytic process requires oxygen and light (Fig. 4a and Supplementary Figs. 76 and 77). Scavenger experiments^51,52^ showed that Tiron (3,5-pyrocatecholdisulfonic acid), a superoxide-specific scavenger,considerably suppresses TMB oxidation, whereas d-mannitol (hydroxyl radical scavenger) and l-tryptophan (singlet oxygen quencher) have negligible effect (Fig. 4b and Supplementary Fig. 78). These results point to superoxide (O_2_^•−^) as the primary intermediate generated by the photoexcited DSA^2+^ aggregates. Direct detection of O_2_^•−^ by spin-trapping electron paramagnetic resonance (EPR) spectroscopy with 5,5-dimethyl-1-pyrroline *N*-oxide (DMPO) showed the characteristic DMPO–O_2_^•−^ adduct upon irradiation^54^ (Fig. 4c and Supplementary Fig. 79). In addition, singlet oxygen emission and anthracene-9,10-dipropionic acid photodegradation experiments ruled out singlet oxygen sensitization (Supplementary Fig. 80). Both singlet and triplet excited states of aggregates are involved in electron transfer to O_2_ and superoxide formation, as shown by fluorescence quenching (Supplementary Fig. 81) and quenching of the triplet state by ns-TAS (Supplementary Fig. 73).

In the absence of TMB probe or ROS scavengers, photo-generated O_2_^•−^ can undergo disproportionation or a further one-electron reduction to yield hydrogen peroxide (H_2_O_2_)^55,56^. We therefore monitored the production of hydrogen peroxide (H_2_O_2_) under continuous illumination. H_2_O_2_ production was linear over time, reaching 2,900 μmol g^−1^ after 4 h (Fig. 4d and Supplementary Fig. 82). Sustained activity requires a sacrificial electron donor, here the buffer 3-(*N*-morpholino)propanesulfonic acid (MOPS), which reduces the oxidized chromophore (DSA^3+^) and regenerates its ground state, thereby closing the photocatalytic cycle (Supplementary Figs. 83 and 84). Control experiments confirm these requirements: removing DSA aggregates, light, oxygen or the sacrificial donor resulted in no or negligible H_2_O_2_ production (Fig. 4e). On the basis of these results, we propose that photoexcited DSA aggregates (DSA^2+^*) transfer an electron to O_2_ to form O_2_^•−^, which undergoes disproportionation or a second one-electron reduction to yield H_2_O_2_, while the oxidized DSA^3+^ is reduced by a sacrificial donor, to regenerate the ground state and complete the photocatalytic cycle (Fig. 4f). Crucially, aggregation enables this reactivity by stabilizing the excited states and suppressing non-radiative decay through dye rigidification.

The ability to generate superoxide and H_2_O_2_ under ambient conditions, without metal co-catalysts, highlights the potential of AIP-based supramolecular systems for sustainable photocatalysis. We also demonstrated this by using DSA^2+^ in light-driven oxidation of organic substrates such as 4-(methylthio)phenol to the corresponding sulfoxide in the presence of oxygen (Supplementary Fig. 85). We also investigated the oxidation of glycerol, an abundant feedstock that can be upgraded to value-added chemicals^57^, and found that DSA^2+^ aggregates (DSAI 0.1 mM, NaI 25 mM) exhibit excellent activity for its selective conversion to glyceraldehyde under white-light irradiation (Supplementary Fig. 86).

### Aggregation enhances photocatalytic hydrogen evolution

**Fig. 5:**
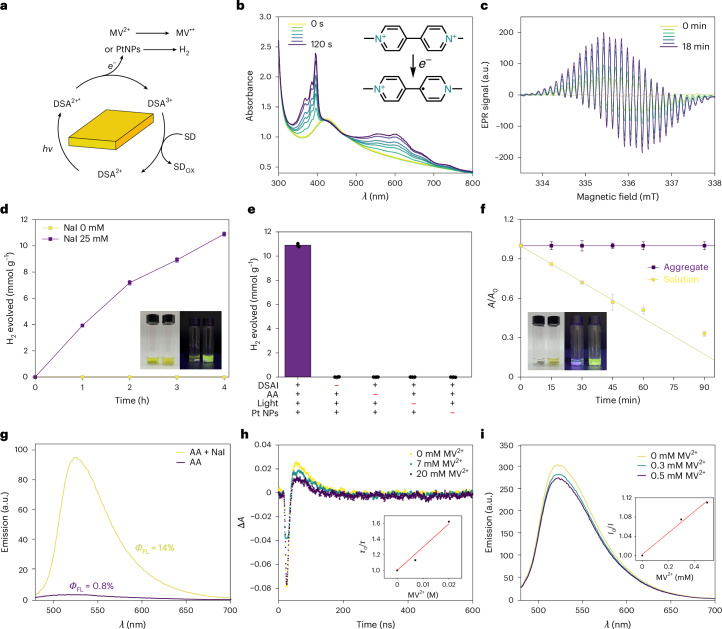
Light-driven hydrogen evolution by DSA^2+^ nanostructures. **a**, Proposed photocatalytic mechanism for methyl viologen (MV^2+^) reduction or hydrogen evolution in presence of a co-catalyst (PtNPs) and sacrificial donor (AA). **b**, UV–visible absorption spectra over time of a mixture of DSAI (1.0 mM) and MV^2+^ (10 mM) under 450 nm (LED, 700 mW cm^−2^) irradiation. **c**, EPR spectra over time of a mixture of DSAI (1.0 mM) and MV^2+^ (10 mM) under 450 nm (LED, 700 mW cm^−2^) irradiation. **d**, H_2_ light-driven evolution over time by irradiation of DSAI solution (yellow trace) or nanostructures (NaI 25 mM, purple trace) in AA (1.0 M, pH 4.0), in presence of PtNPs (8% mol) with white light (LED, 100 mW cm^−2^). The inset shows DSAI samples in AA without (left) and with (right) NaI under daylight and UV light. Statistics are from three independent groups. **e**, Control experiments for H_2_ produced by DSAI (10.9 ± 0.1, 0.00 ± 0, 0.00 ± 0, 0.00 ± 0 and 0.00 ± 0 mmol g^−1^). Statistics are from three independent groups. **f**, Absorbance over time by irradiation of DSAI solution or DSAI nanostructures in AA. The inset shows irradiated DSAI samples in AA without (left) and with (right) NaI under daylight and UV light. Statistics are from two independent groups. **g**, Emission spectra of DSAI 0.1 mM dissolved in AA 1.0 M (pH 4.0) and aggregated in AA 1.0 M (pH 4.0) with NaI 25 mM (*λ*_exc_ = 395 nm, at isoabsorbance). **h**, Transient absorption kinetic traces at 420 nm of an aqueous solution of aggregates of DSAI 0.05 mM and 2 mM of NaI (yellow trace) and after addition of methyl viologen ([MV^2+^] = 7 mM (green trace) and 20 mM (purple trace). *λ*_exc_ = 355 nm. *A*_355 nm_ = 0.31, 1 cm optical path, 4.4 mJ per pulse. The inset shows the Stern–Volmer analysis. **i**, DSAI 0.1 mM with NaI 0.1 M emission quenching with different MV^2+^ concentration (*λ*_exc_ = 470 nm at isoabsorbance). The inset shows the Stern–Volmer analysis. SD, sacrificial donor. Source data

The aggregates show promising electron transfer properties for photocatalysis, as also supported by their calculated excited-state oxidation and reduction potentials relative to commonly used photocatalysts (Supplementary Figs. 87 and 88). The reduction potential of DSA^2+^ is nearly unchanged from the molecular to the aggregate state (*E*_red_ = –1.01 V versus –1.09 V versus NHE, respectively), as evidenced by electrochemical measurements (Supplementary Fig. 89) and calculations (Supplementary Figs. 90–92). This could be an advantageous feature, because strong hydrophobic interactions and molecular orbitals mixing between monomers often lead to large changes in redox potentials^58^. Thus, preserving redox and excited-state potentials across monomer and aggregate states simplifies predictive design. To assess whether AIP extends beyond oxygen photoreduction, we investigated the ability of DSA^2+^ aggregates to drive hydrogen evolution (Fig. 5a). We first explored photoinduced electron transfer to methyl viologen (MV^2+^), a well-established redox probe (*E*°′ = −0.45 V versus NHE)^59^. Upon irradiation of DSA aggregates we observed an increase in the UV–visible absorption bands of the MV^•+^ radical cation (Fig. 5b), whose formation was confirmed by its characteristic EPR spectrum (Fig. 5c). The ability of the DSA^2+^ to photoreduce MV^2+^ implies that the aggregates could also drive proton reduction (*E*°′ = −0.35 V versus NHE at pH 6).

We then investigated light-driven hydrogen evolution in presence of Pt nanoparticles (PtNPs, pre-made 3-nm colloidal dispersion) as co-catalysts and ascorbic acid (AA) as sacrificial electron donor (Fig. 5a). Interestingly, adding the DSAI to an AA solution dissolved the powder, probably because DSA^2+^ becomes solubilized through counterion exchange with solvated ascorbate anions. This enabled direct comparison of hydrogen evolution in the molecularly dissolved and aggregated states, the latter obtained by adding NaI under otherwise identical conditions. In the absence of NaI, when DSA^2+^ is dissolved, no H_2_ evolution is observed (Fig. 5d and Supplementary Fig. 93). By contrast, NaI-induced charge screening and aggregation increased hydrogen production to over 10,900 μmol g^−1^ after 4 h of white-light irradiation (Fig. 5d). Control experiments confirmed that all components (DSAI, AA, PtNPs and light) are essential for the observed activity (Fig. 5e and Supplementary Figs. 94–97). The apparent quantum yield for photocatalytic H_2_ evolution was 0.2%, with the photocatalytic activity matching the absorption profile of the aggregate (Supplementary Fig. 98), consistent with reported values for nanostructured chromophore amphiphiles^25^. Further gains in efficiency could probably be achieved by improving electron-transfer kinetics and interfacial coupling between the aggregates and co-catalysts; for example, in situ photodeposition of PtNPs can promote more efficient electron transfer and a better co-catalyst distribution than impregnation with pre-formed, ligand-capped PtNPs^4^.

After photocatalysis, the supramolecular aggregates were recovered by filtration. The recovered solid remained active, whereas the filtrate showed no activity under identical conditions (Supplementary Figs. 99 and 100), confirming that the photocatalytic function resides in the aggregated state. Consistent with this, aggregated DSAI was also more photostable than the solution species under prolonged illumination, which underwent steady photobleaching (Fig. 5f and Supplementary Fig. 101). Aggregation therefore both enables photocatalysis and stabilizes the chromophore against non-radiative decay and degradation.

Fluorescence experiments revealed that salt-induced aggregation markedly increased emission, with *Φ*_FL_ going from 0.8% to 14% (Fig. 5g and Supplementary Fig. 102 for excitation–emission maps), whereas ns-TAS showed the emergence of a triplet state in the aggregated material (Fig. 5h). To quantitatively determine which excited state (singlet or triplet) dominates the productive electron-transfer step under catalytic conditions, we performed quenching experiments (Fig. 5h,i and Supplementary Fig. 103). These experiments revealed that both the excited states of the aggregated DSAI are quenched with increasing concentration of the acceptor MV^2+^, with the singlet (*k*_SV_^S^ = 222 M^−1^ from fluorescence titration) prevailing over the triplet state (*k*_SV_^T^ = 32 M^−1^ from ns-TAS titration). Altogether, these results show that aggregation enhances not only oxygen reduction but also proton reduction. Both reactivities arise from a common mechanism in which photoexcited DSA^2+^* aggregates undergo electron transfer to an external acceptor, either molecular oxygen or PtNPs. The higher fluorescence quantum yield of the aggregate indicates a greater abundance of excited states, increasing the probability of productive electron transfer either through singlet- or triplet-state reactivity. Aggregation also confers marked photostability, as shown by suppressed photobleaching under continuous irradiation. These features (higher excited-state utilization, enhanced reactivity and improved stability) further define the scope and advantage of the AIP approach.

### Photocatalytic performance depends on aggregation pathway

The DSA nanostructures described so far were obtained immediately upon sample preparation in water (or buffer) and are therefore considered kinetic aggregates. Supramolecular systems, however, often exhibit multiple assembly pathways, and structures formed under kinetic or thermodynamic control can differ markedly in packing, photophysical properties and functional output^2,60^. We therefore investigated how kinetically trapped or thermodynamically controlled aggregates differ in structure, emissive behaviour and photocatalytic function.

**Fig. 6:**
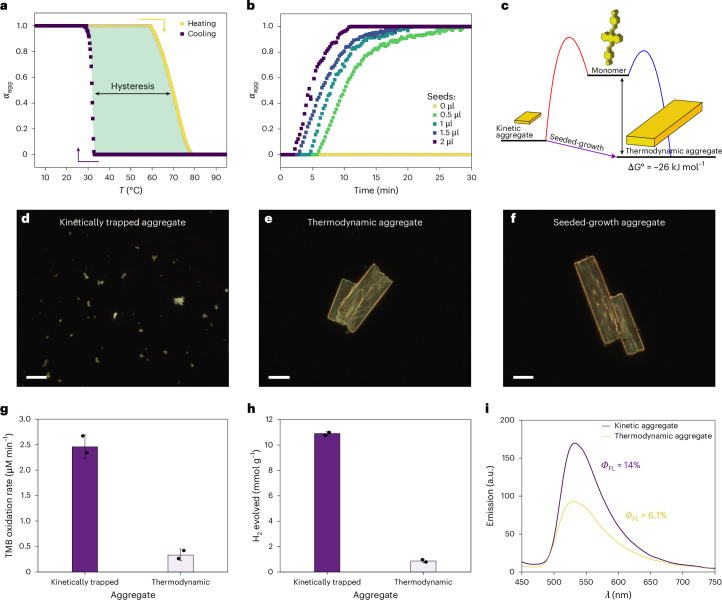
Supramolecular polymerization pathways and photocatalysis obtained with DSAI aggregates. **a**, Temperature-dependent degree of aggregation (*α*_agg_, estimated from the absorption at 410 nm) of DSAI (*c*_T_ 50 μM, NaI 5.0 mM) as a function of temperature upon heating (yellow traces) and cooling (purple traces) at a rate of 1.0 K min^−1^. **b**, Time-dependent degree of aggregation (*α*_agg_, estimated from the absorption at 410 nm) of DSAI (*c*_T_ 50 μM, NaI 2.5 mM) upon addition of various amounts of DSAI_seed_ nanostructures (0−2 μl) at 30 °C. **c**, A schematic representation of the free energy landscape in supramolecular self-assembly of DSAI aggregates. **d**, WFM image of kinetically trapped DSAI supramolecular nanostructures in water (DSAI 50 μM, NaI 5.0 mM). Scale bar, 20 μm. **e**, WFM image of thermodynamic DSAI supramolecular nanostructures in water (DSAI 50 μM, NaI 5.0 mM). Scale bar, 20 μm. **f**, WFM image of seeded-growth DSAI supramolecular nanostructures in water (DSAI 50 μM, NaI 5.0 mM). Scale bar, 20 μm. **g**, TMB oxidation rates (2.5 ± 0.2, 0.33 ± 0.11 μM min^−1^) by irradiation of various DSAI aggregated states with white light (LED, 180 mW cm^−2^). Statistics are from two independent groups. **h**, H_2_ evolution (10.9 ± 0.2, 0.87 ± 0.13 mmol g^−1^) by irradiation of various DSAI aggregated states (0.1 mM) with white light (LED, 100 mW cm^−2^) in presence of AA (1.0 M, pH 4.0) and co-catalyst (PtNPs, 8% mol). Statistics are from two independent groups. **i**, Fluorescence emission spectra and fluorescence quantum yields (*Φ*_FL_) of various DSAI aggregated states. Source data

To study the assembly pathway of DSAI (total concentration, *c*_T_ = 50 μM) in NaI solution (5.0 mM), we monitored variable-temperature UV–visible absorption. Upon heating, the spectral changes indicate solubilization of the supramolecular polymer into monomers, whereas cooling shows transition from monomeric DSAI to its aggregate state (Fig. 6a and Supplementary Fig. 104), which we plotted as degree of aggregation (*α*_agg_)^61–63^. The non-sigmoidal cooling transition and thermal hysteresis between the critical temperatures of heating (*T*_e_ = 351 K) and cooling (*T*_e_′ = 306 K) indicate a nucleation-elongation process^61–63^. This is further supported by *T*_e_′ shifting to higher temperature as the cooling rate was decreased progressively from 2.0 to 1.0 K min^−1^ (Supplementary Fig. 105). Upon dilution of DSAI, the elongation temperature (*T*_e_′) decreased linearly in the van’t Hoff plot, from which the standard enthalpy (Δ*H*°) and entropy (Δ*S*°) were estimated as −67 kJ mol^−1^ and −139 J mol^−1^ K^−1^, respectively (Supplementary Fig. 106). Furthermore, the lag time required to reach the thermodynamic equilibrium enabled supramolecular polymerization and crystal growth to be initiated by seeding. Seeding thermally annealed solutions with increasing amounts of pre-formed nanoribbons at 30 °C accelerated formation of extended crystalline structures and shortened the time to equilibrium (Fig. 6b). A schematic free energy diagram illustrates the higher energy barrier for direct nucleation than for seeded growth (Fig. 6c), consistent with the lag phase in unseeded samples. Microscopy confirmed that these different pathways lead to distinct morphologies: kinetically trapped aggregates appear as small particles (Fig. 6d and Supplementary Figs. 107–109), whereas seeded and thermodynamic aggregates yield large, well-defined platelets with anisotropic geometries (Fig. 6e,f and Supplementary Figs. 110–115). Strikingly, three-dimensional electron diffraction experiments showed that kinetic and thermodynamic aggregates share the same underlying crystal structure. However, kinetic aggregates display lower diffraction and higher mosaicity (consistent with faulted stacking, domain boundaries and disorder introduced during rapid, non-equilibrium assembly), whereas thermodynamic aggregates exhibit sharper diffraction indicative of better long-range order (Supplementary Figs. 116–118).

We then compared the photocatalytic performance of thermodynamic and kinetically trapped aggregates, the latter formed under high ionic strength conditions that suppress nucleation-elongation events. ROS generation, measured by TMB oxidation, was notably higher for the kinetically trapped aggregates (*v*_0_ = 2.4 μM min^−1^) than for the thermodynamic counterpart (*v*_0_ = 0.33 μM min^−1^) (Fig. 6g and Supplementary Fig. 119). Similarly, hydrogen evolution from Pt-catalysed proton photoreduction was higher for the kinetically formed structures (H_2_ 10,900 μmol g^−1^) than for aggregates obtained upon annealing (H_2_ 867 μmol g^−1^) (Fig. 6h and Supplementary Fig. 120). Moreover, kinetically trapped aggregates do not grow into their thermodynamic counterparts over several days, as shown by unchanged morphology and comparable photocatalytic activity upon aging (Supplementary Figs. 121 and 122). These functional differences probably originate from the distinct excited-state properties arising from the supramolecular polymerization pathway, as well as differences in surface/volume ratio (Supplementary Fig. 123). Indeed, steady-state fluorescence measurements showed that kinetically formed aggregates possess a higher quantum yield (*Φ*_FL_ = 14%) than thermodynamic aggregates (*Φ*_FL_ = 6.1%, Fig. 6i). This suggests that kinetic aggregation traps chromophores in a state of greater excited-state availability, thereby boosting photoredox efficiency. Taken together, these findings highlight the importance of pathway control in designing functional supramolecular photocatalysts, where transient or metastable states can outperform thermodynamic structures. This contrasts with established crystalline supramolecular polymers, in which the enhanced charge separation in extended nanostructures leads to better H_2_ production than in their kinetic counterparts^2,60^.

### Generality of AIP across chromophore scaffolds

**Extended Data Fig. 1:**
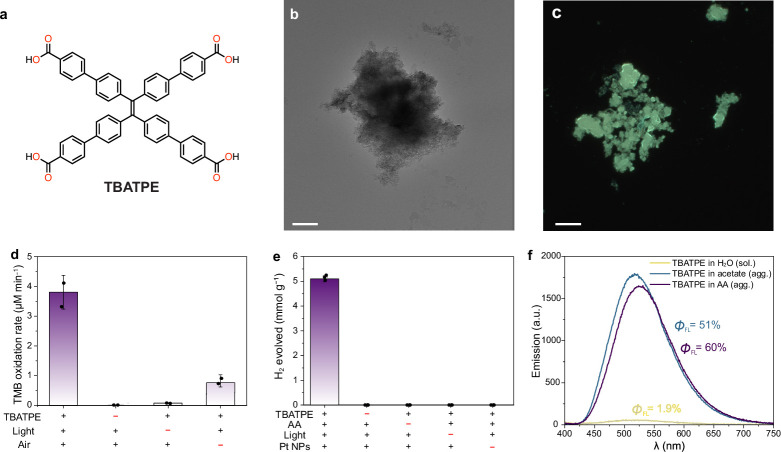
Generalizing aggregation-induced photocatalysis with a tetraphenylethylene derivative (TBATPE). **a**, Chemical structure of TBATPE derivative. **b**, Transmission electron microscope (TEM) image of TBATPE aggregates in AA (TBATPE 0.1 mM, AA 5 mM). Scale bar is 1 μm. **c**, Widefield fluorescence microscope (WFM) image of TBATPE supramolecular aggregates in AA (TBATPE 0.1 mM, AA 5 mM). Scale bar is 20 μm. **d**, TMB oxidation rates and controls (3.8 ± 0.6, 0.0 ± 0, 0.08 ± 0.01, 0.82 ± 0.12 μM min^−1^) by irradiation of TBATPE aggregates (0.2 mM) with white light (LED, 180 mW cm^−2^) in presence of acetate buffer (60 mM, pH = 5). Statistics are from 2 independent groups. **e**, H_2_ evolution and controls (5.1 ± 0.1, 0.0 ± 0, 0.0 ± 0, 0.0 ± 0, 0.0 ± 0 mmol g^−1^) by irradiation of TBATPE aggregates (0.1 mM) with 415 nm (LED, 140 mW cm^−2^) in presence of ascorbic acid (0.8 M, pH = 4.0) and co-catalyst (PtNPs, 8% mol). Statistics are from 3 independent groups. **f**, Fluorescence emission spectra and fluorescence quantum yields (*Φ*_FL_) of TBATPE solution and aggregates under the photocatalytic conditions. Source data

To test the generality of AIP, we decided to study an amphiphilic tetraphenylethylene derivative bearing four benzoic acid groups (TBATPE) (Extended Data Fig. 1a). Unlike the DSA^2+^ scaffold, the TBATPE lacks a central *π*-extended core, but the tetraphenylethylene scaffold is also known to exhibit strong AIE due to restricted intramolecular motion in the aggregate state^19,64–66^.

TBATPE further supports the generality of AIP. In acidic aqueous media, it forms emissive aggregates (Extended Data Fig. 1b,c and Supplementary Figs. 125 and 126), consistent with aggregation-induced rigidification. These aggregates photocatalyse both TMB oxidation under air (*v*_0_ = 3.8 μM min^−1^), with negligible activity in the absence of light or O_2_, and H_2_ evolution in the presence of PtNPs and AA, reaching 5,100 μmol g^−1^ after 24 h (Extended Data Fig. 1d,e and Supplementary Figs. 127 and 128). Aggregation is also accompanied by a marked increase in fluorescence quantum yield, from 1.9% in water to 51% in acetate buffer and 60% in AA (Extended Data Fig. 1f), mirroring the trend observed for DSAI. Together, these results show that even a non-planar, nonlinear chromophore such as TBATPE can access fluorescence and photocatalytic activity upon aggregation, despite forming amorphous rather than crystalline assemblies (Supplementary Fig. 129). This supports AIP as a general design principle whereby aggregation-induced rigidification activates otherwise inactive dyes across structurally diverse systems.

## Conclusion

We identify AIP as a supramolecular mechanism for organic dyes in water, in which aggregation-induced rigidification suppresses non-radiative decay and stabilizes localized reactive excited states, rather than relying on exciton delocalization or packing-induced changes in electronic structure. In this way, both singlet- and triplet-derived pathways can contribute to photocatalysis^67^, although singlet reactivity dominates in the present system. Using an amphiphilic distyrylanthracene dye, we show that aggregation controlled by concentration, ionic strength and counterion identity enhances emission, ROS generation and hydrogen evolution from the same molecular scaffold, while leaving the underlying excited-state energetics and redox potentials largely unchanged. The effects of counterion identity and aggregation pathway further show that supramolecular organization can tune function without requiring strong intermolecular electronic coupling. Aggregation-induced rigidification thus complements established covalent rigidification strategies, including sterically constrained structures^68^, covalent organic frameworks, macrocycles, molecular cages and organic–inorganic superlattices^17,42,69–71^. The observation of the same behaviour in a structurally unrelated tetraphenylethylene-based system, including amorphous aggregates, supports the generality of this concept. More broadly, AIP offers a rich route to molecular photocatalyst design based on localized excited-state reactivity, modular self-assembly and control over aggregation pathway, rather than extended electronic coupling. It thus bridges the defined excited-state reactivity of homogeneous molecular catalysts with the robustness, photostability and recyclability of heterogeneous systems, providing a design paradigm for soft photocatalysts in aqueous media.

## Methods

### Samples preparation

For counterion-dependent studies, DSAX (X = Cl^−^, Br^−^, NO_3_^−^, I^−^ and PF_6_^−^) stock solutions in dimethyl sulfoxide (DMSO) (30 mM) were diluted into water to give samples at 1.5, 1.0, 0.15, 0.10 and 0.05 mM in DMSO (5% *v*/*v*), with stirring before each measurement. For solvent-composition studies, a DSAI suspension in water (3 mM) was diluted with defined DMSO/H_2_O mixtures to obtain samples at 1.5, 1.0, 0.75, 0.50 and 0.25 mM containing 0–40% DMSO (*v*/*v*). For ionic-strength studies, aqueous DSAI samples (0.5 mM) were prepared with variable NaI concentration (0–0.5 mM) by dilution of stirred DSAI (1 mM) and NaI (25 mM) stock solutions. The DSAI-NaI samples were then diluted to obtain the final concentrations (DSAI 0–0.3 mM and NaI 0–0.5 mM) and were stirred for 10 min before *Φ*_FL_ measurements.

Microscopy samples were prepared with DSAX-based aggregates under defined ionic and thermal conditions. DSAI samples were prepared by adding a NaI solution (1 M) to a DSAI solution (0.05 mM) to reach a final concentration of DSAI 0.05 mM and NaI 0.5 mM. For the supramolecular pathways studies, kinetic aggregates (DSAI 0.05 mM, NaI 5 mM) were prepared by adding NaI stock solution (1 M) to a DSAI solution (0.05 mM); thermodynamic aggregates were obtained by heating the same mixture to 95 °C for 2 min followed by slow cooling overnight; for seeded-growth samples, a small aliquot of pre-formed DSAI seeds was added to a DSAI solution at 50 °C during the cooling process, giving a 49:1 ratio of dissolved DSAI to seeds. DSAX samples were prepared from DSACl, DSABr and DSANO_3_ at 0.05 mM with the corresponding added salts (NaCl, NaBr or NaNO_3_), or NaI for DSACl, followed by 5–15 min stirring. DSAPF_6_ samples were prepared either by direct sonication in water or by nanoprecipitation from DMSO (0.1 ml of 5 mM) into water (1.9 ml) under stirring.

### Absolute fluorescence quantum yields (*Φ*_FL_)

Measurements were performed with Quantaurus-QY Absolute PL quantum yield spectrometer C11347-11 integrating sphere in air-equilibrated condition using an empty quartz tube as a reference. For DSAX samples, 360–480 nm excitation wavelengths (20 nm steps) were used. The final *Φ*_FL_ represents the average, which was calculated from the quantum yields measured at different excitation wavelengths.

### ROS experiments

The photooxidation experiments of TMB were performed in air atmosphere using quartz cuvettes (10 mm path length) with a PTFE lid. A stock sample of DSAX (X = Cl^−^, Br^−^, NO_3_^−^ or I^−^) (0.4 mM) in acetate buffer (0.1 M, pH 5) was sonicated for 10 min and then diluted to 0.3 mM DSAX with acetate buffer (3 ml total volume), followed by addition of 0.1 ml of TMB (1 mg ml^−1^ in DMSO) under stirring. In the case of DSAPF_6_, a stock solution in DMSO (18 mM) was added (0.05 ml) to acetate buffer solution (3 ml) under stirring, followed by addition of TMB (0.05 ml, 2 mg ml^−1^ in DMSO). All DSAX samples (final concentration of 0.3 mM) were left under stirring at least 1 h at room temperature. For DSAI concentration-dependent studies, the DSAI stock sample (0.4 mM) was diluted with acetate buffer to obtain the final concentration (3 ml of 0.1, 0.2, 0.3 and 0.4 mM), before adding 0.1 ml of TMB (1 mg ml^−1^ in DMSO) under stirring and stirring for 1 h. For NaI concentration screening, different dilutions of a NaI stock (20 mM) in acetate buffer (0.1 M at pH 5) were prepared (1.5 ml), followed by addition of 1.5 ml of DSAI suspension (0.2 mM in acetate buffer) under stirring to obtain DSAI samples (0.1 mM) with final NaI concentrations of 0, 0.2, 0.4 and 0.6 mM, respectively. Then, 0.1 ml TMB (1 mg ml^−1^ in DMSO) was added to the vials and left under stirring under equilibration (monitored by UV–visible spectroscopy). TMB final concentration in all samples was 134 μM. Experiments under O_2_ and N_2_ atmosphere were performed in quartz cuvettes (10 mm path length) with screw cap and sealed with silicone/PTFE septum. Cuvettes were positioned into a homemade built photoreactor using a three-dimensional printed sample support and white-light light-emitting diodes (LEDs) (irradiation at 180 mW cm^−2^). The TMB oxidation process was monitored with UV–visible spectroscopy by focusing on the absorption band of oxidized TMB (*Ɛ*_652_ = 3.9 × 10^4 ^M^−1^ cm^−1^).

### Photoproduction of hydrogen peroxide

A stock solution of DSACl (0.5 mM) in 3-(*N-*morpholino)propanesulfonic acid (0.1 M, pH 7.0) was prepared. A total of 1.0 ml of this solution was transferred to a 9.0 ml screw cap vial. Then, powdered NaCl (53 mg, 0.90 mmol) was added to prepare the aggregated samples. Vials were then purged for 15 min with O_2_ by using steel needles through the septum as inlet (longer needle inside the solution) and outlet (shorter needle in the headspace). Then the purging was stopped, the long needle was retracted to the headspace, and then the short one was removed. The vials were illuminated under stirring at 300 rpm using a homebuilt photoreactor made of 415 nm light LEDs with a light intensity of 140 mW cm^−2^. Hydrogen peroxide concentrations were determined using horseradish peroxidase enzyme and TMB method^30^.

### Photocatalytic H_2_ evolution

A stock solution of DSAI (0.1 mM) in AA (1 M, pH 4.0) was prepared. Then, 973 μl of this solution was transferred to a 9.0 ml screw cap vial, followed by addition of pre-made PtNPs (1.6 μl; 3nm particle size, 1,000 ppm in H_2_O, Sigma-Aldrich). Then, 25 μl of a NaI stock solution (1 M) was added to prepare the aggregated samples, whereas 25 μl of H_2_O was added to prepare the solution samples. Vials were then purged for 15 min with N_2_, followed by equilibration to atmospheric pressure and irradiated with white-light LED (100 mW cm^−2^) under 300 rpm stirring. The produced H_2_ was detected using a gas chromatograph (Agilent Technologies 8860 with autosampler PAL3 coupled with TCD detector at 250 °C) by injecting 100 μl from the headspace, and the H_2_ evolved was calculated using a calibration plot.

For details of the molecular synthesis and the photophysical, photocatalytic, microscopic and electron diffraction experiments, please refer to the Supplementary Information.

## Online content

Any methods, additional references, Nature Portfolio reporting summaries, source data, extended data, supplementary information, acknowledgements, peer review information; details of author contributions and competing interests; and statements of data and code availability are available at 10.1038/s41557-026-02151-4.

## Supplementary information

Supplementary Information Supplementary Methods, Discussion, Synthetic procedures, Figs. 1–129 and Tables 1–7.

Peer Review File

## Source data

Source Data Fig. 2 Unprocessed PLQY maps, unprocessed microscopy images, height profile and crystallographic packing.

Source Data Fig. 3 Unprocessed spectra, unprocessed dot plots, kinetics data with statistical errors.

Source Data Fig. 4 Unprocessed dot plots, unprocessed EPR spectra, kinetic analysis with statistical errors.

Source Data Fig. 5 Unprocessed spectra, unprocessed dot plots, analysis with statistical errors and Stern–Volmer analysis.

Source Data Fig. 6 α aggregation plots, unprocessed microscopy images, unprocessed dot plots and unprocessed spectra.

Source Data Extended Data Fig./Table 1 Unprocessed microscopy images, unprocessed dot plots and unprocessed spectra.

## Data Availability

All the data supporting the findings of this study are available within the Article and its Supplementary Information and also from the corresponding author upon request. The X-ray crystallographic coordinates for structures reported in this study have been deposited at the Cambridge Crystallographic Data Centre (CCDC), under deposition number CCDC-2485494. These data can be obtained free of charge via The Cambridge Crystallographic Data Centre at www.ccdc.cam.ac.uk/data_request/cif. Source data are provided with this paper.
