# Supplementary material for: Supramolecular dye polymers for aggregation-induced photocatalysis
Source: Nat Chem. 2026 May 18;18(8):1372–82. doi: 10.1038/s41557-026-02151-4 (PMC13423815; doi:10.1038/s41557-026-02151-4)
Supplement: Supplementary file 1 — Supplementary Methods, Discussion, Synthetic procedures, Figs. 1–129 and Tables 1–7. [file 41557_2026_2151_MOESM1_ESM.pdf]

nature chemistry

Supplementary information https://doi.org/10.1038/s41557-026-02151-4

Supramolecular dye polymers for aggregation-induced photocatalysis

In the format provided by the authors and unedited

Table of Contents

1. Supplementary Methods 2
2. Supplementary Discussion 18
3. Synthetic Procedures 20
4. Supplementary Figures 32
5. Supplementary Tables 142
6. References 147

1. Supplementary Methods

Materials. All commercially available reagents and chemicals were purchased from Merck-Sigma Aldrich, Fisher Scientific, TCI, Avantor and used as received. Solvents, including dry solvents, were purchased from Sigma Aldrich and ThermoFisher and were used without further purifications, and deuterated solvents were obtained from Sigma Aldrich and VWR.

Thin layer chromatography (TLC) was performed on pre-coated Aluminum sheets with 0.20 mm Merck Silica gel 60 F254.

Melting points were measured on Cole-Parmer MP-100 Series apparatus in open capillary tubes.

NMR spectra were obtained on a Varian 400 MHz NMR or Bruker 400 Avance III HD (400 MHz ^{1}H and 101 MHz ^{13}C) spectrometer at room temperature (298 K). Chemical shifts are reported in ppm using the solvent residual signal as internal reference (Chloroform-*d*: δH = 7.26 ppm, δC = 77.23 ppm; DMSO-d_6: δH = 2.50 ppm, δC = 39.52, D_2O: δH = 4.79). The resonance multiplicity is described as s (singlet), d (doublet), t (triplet), …, m (multiplet), dd (doublet of doublets), bs (broad singlet). ^{1}H diffusion-ordered spectroscopy (DOSY) was acquired with the standard Bruker ledbpgp2s program on Bruker 400 Avance III HD spectrometer, using diffusion time (Δ) = 100 ms, gradient pulse duration (δ) = 1.1 ms, number of scans = 200, and 16 gradients within 5–95 % range.

Mass spectrometry (MS) was performed on a mass spectrometer detector (MSD Trap SL, model G2245D) equipped with an atmospheric pressure chemical ionization (APCI) or electrospray ionization (ESI) source. The injection was provided thanks to an HPLC system (Agilent Technologies, 1100 series). Signals are reported in *m/z* units with $[M]^+$ or $[M]^-$.

IR spectra were recorded with a Jasco FT/IR-4100, the samples were prepared by mixing the product with KBr and pressed into a pellet.

Photophysical measurements were performed as detailed below:

- **Absorption spectra** of compounds were recorded at room temperature with an Agilent Cary 60 UV-Vis spectrophotometer, using quartz cells with a path length of 10 mm. To estimate molar extinction coefficients (ε), each DSAX compound and TBATPE were weighted three separate times and used to prepare solutions in DMSO at 100 µM (DSAX, X = Cl, Br, NO_3, and TBATPE) or 60 µM (DSAX, X = PF_6, I). Then, the stock solutions were further diluted (20 and 30 µM for DSAX, 5

and 10 μM for TBATPE). Finally, the solutions were transferred to quartz cuvettes (10 mm path length), and UV-Vis spectra were recorded. Error related to the estimated ε indicates the standard error of the mean.

- **Fluorescence emission spectra** of liquids and suspensions were recorded at room temperature with an Agilent Cary Eclipse fluorescence spectrofluorometer using quartz cells with a path length of 10 mm. The emission spectra of solids were recorded on a FLS1000 Instrument (Edinburgh Instrument), with the solids diluted with $BaSO_4$. Phosphorescence spectrum of DSAI powder was measured with an Agilent Cary Eclipse at 77 K (time delay of 0.1 ms).
- **Fluorescence lifetimes** measurements were performed on an FLS1000 Instrument (Edinburgh Instruments) equipped with a pulsed ps laser diode at 402.6 nm (EPL405) as excitation source and an air-cooled single-photon counting photomultiplier (Hamamatsu R13456P) as detector. The decays were recorded using the time correlated single-photon counting (TCSPC) method and were analyzed with the Fluoracle® Software using the IRF convolution fitting procedure. The average decays were calculated as intensity weighted. Typically, measurements were carried out in 0.2 mm path length cuvettes using samples prepared according to the photocatalysis protocol – while TMB dissolved in DMSO was added in photocatalytic experiments, fluorescence lifetime measurements were performed by adding the same volume of DMSO only, ensuring identical solvent composition.
- **Absolute fluorescence quantum yields (Φ_{FL})** measurements were performed with Quantaurus-QY® Absolute PL quantum yield spectrometer C11347-11 integrating sphere in air-equilibrated condition using an empty quartz tube as a reference. For DSAX samples, 360–480 nm excitation wavelengths (step 20 nm) were adopted; while for TPE samples, 300–420 nm excitation wavelengths (step 20 nm) were used. The final Φ_{FL} represents the mean, which was calculated from the quantum yields at different excitation wavelengths. Typically, measurements were prepared according to the photocatalysis protocol – while TMB dissolved in DMSO was added in photocatalytic experiments, fluorescence lifetime measurements were performed by adding the same volume of DMSO only, ensuring identical solvent composition. Regarding the assembly landscapes, samples were prepared using the following procedures:

- Assembly landscapes at different concentration of DSA^{2+} amphiphile (mM) with selected counteranions. The DSAX (X = Cl, Br, NO_3, I, PF_6) stock solutions in DMSO (30 mM) were prepared. Under stirring, each solution aliquot (0.10 mL) was added to mQ-water (1.9 mL) to obtain DSAX sample (1.5 mM) in 5% DMSO (2 mL total volume). After stirring for 15 min, Φ_{FL} measurements were taken. Each sample was then diluted to 1.0 mM (2 mL total) using mQ-water with 5% DMSO and stirred for 10 min before measurements. The same procedure was followed to prepare and analyze 0.15, 0.10, and 0.05 mM samples.
- Assembly landscapes at different concentration of DSAI amphiphile (mM) under various ratios between water and DMSO**.** A DSAI suspension (3 mM) in mQ-water was prepared and stirred. Aliquots (1 mL) were transferred into five vials and diluted with various *v*/*v* of DMSO/H_2O mixtures to obtain the samples (1.5 mM) containing 0%, 10%, 20%, 30%, and 40% DMSO in mQ-water, respectively. After stirring for 15 min, Φ_{FL} measurements were taken. Each sample was then diluted to 1 mM (2 mL total) using the corresponding DMSO/H_2O *v*/*v* ratio and stirred for 10 min before measurements. The same procedure was followed to prepare and analyze 0.75, 0.50, and 0.25 mM samples.
- Assembly landscapes at different concentration of DSAI amphiphile (mM) with different amount of NaI (mM)**.** A DSAI suspension (1 mM) and NaI solution (25 mM) in mQ-water were prepared and stirred. Five vials were prepared by diluting 0, 10, 20, 30, or 40 μL of NaI (25 mM) with mQ-water to a total volume of 1 mL, followed by addition of 1 mL of the DSAI suspension (1 mM) to obtain DSAI samples (0.5 mM) with final NaI concentrations of 0, 0.125, 0.250, 0.375, and 0.500 mM, respectively. After stirring for 15 min, Φ_{FL} measurements were taken. Each sample was then diluted to 0.3 mM (2 mL total) using mQ-water containing the corresponding NaI concentration and stirred for 10 min before measurements. The same procedure was followed to prepare and measure 0.2, 0.1, and 0.05 mM DSAI samples with various NaI concentrations.

- **Transient absorption spectroscopy.** Transient absorption in the femtosecond range was performed by means of an Ultrafast Systems HELIOS (HE-VIS) femtosecond transient absorption spectrometer by using, as an excitation

source, a Coherent Astrella Laser, combined with an OPERA SOLO optical parametric amplifier (pulse width: 32 fs, 1 kHz repetition rate, selected output wavelengths: 420 nm). The Astrella system was composed of a Coherent Vitara™ that includes a modelocked Ti:Sapphire oscillator cavity pumped by the Coherent Verdi™ G-Series, a continuous-wave OPSL (optically pumped semiconductor laser) green laser, a Ti:Sa regenerative amplifier pumped by an intracavity-doubled, a DPSS (diode pumped solid state, REVOLUTION) Q-switched laser with a second-harmonic generator. Operating at 527 nm and a 1-kHz repetition rate, pump power for the regenerative amplifier module, an optical pulse stretcher, and an optical pulse compressor. The overall temporal resolution of the system is 300 fs. Air-equilibrated solutions in 0.2 cm optical path cells were analysed under continuous stirring. The pump energy on the sample was 4 μJ/pulse at 420 nm. Surface Xplorer V4 software from Ultrafast Systems was used for the data acquisition and analysis. The 3D data surfaces were corrected for the chirp of the probe pulse prior to the analysis. Lifetimes were taken as average of values derived from the fitting of several decays at different wavelengths. Errors on lifetimes were estimated as average of the errors reported by the fitting software for each lifetime. Steady-state absorption spectra were registered before and after transient absorption experiments to check the photostability of the investigated system.

The LP980 setup from Edinburgh Instruments equipped with an Nd:YAG laser from Litron (Nano) was employed for transient absorption and time-resolved emission spectroscopy. The frequency-tripled output with a wavelength of 355 nm served as the excitation source. The laser pulse duration was 7 ns, and the pulse frequency was 5 Hz. The typical pulse energy used for transient absorption and emission studies was 4.4 mJ. Detection of transient absorption spectra occurred by kinetic traces detected at selected wavelengths were recorded using a photomultiplier tube. The spectroscopic experiments were performed at 293 K using a cuvette holder that allows temperature control.

- **Temperature-dependent UV-Vis experiments** were performed in 10 mm cuvette using Agilent Cary 60 UV-Vis spectrophotometer coupled with Quantum Northwest TC1 temperature controller. Heating-cooling cycles were performed from 5 °C to 95 °C and backward for three cycles with DSAI 50 μM and NaI 5 mM in mQ-water, using a rate of 1.0 K min^{-1}. Experiments at different

concentrations (45, 50, 55, and 60 μM of DSAI with NaI 5 mM) were performed by cooling the solutions from 95 °C to 5 °C, using a rate of 1.0 K min^{-1}. Standard enthalpy, entropy and Gibbs free energy were calculated using Van't Hoff equation, as previously reported in literature.[1–3]

$$ln\left(\frac{1}{c_T}\right) = -\frac{\Delta H^0}{RT_e} + \frac{\Delta S^0}{R}$$

Experiments with different cooling rates (2.0, 1.5, and 1.0 K min^{-1}) were conducted by cooling a 50 μM DSAI and NaI 5 mM solution from 95 °C to 5 °C. Experiments for seeded-growth analysis were conducted with DSAI 50 μM and NaI 2.5 mM. The suspension was solubilized and annealed at 95 °C for 5 minutes, then quickly cooled at 30 °C. When 30 °C temperature was reached, different volumes of seeds (0, 0.5, 1.0, 1.5, 2.0 μL of DSAI 0.5 mM) were added. Absorbance at 410 nm was followed in each plotted measurement.

Microscopy samples were prepared as detailed below:

- DSAI with NaI (0.05 mM and 0.5 mM, respectively) was prepared in a vial by adding 1 μL of 1 M NaI in mQ-water to a 2 mL sample of 0.05 mM DSAI previously dissolved in mQ-water.
- In the case of the DSAI kinetic aggregate, a suspension of 0.5 mM DSAI in mQ-water was prepared in Eppendorf® tube. 100 μL was transferred to a new Eppendorf® tube, then 895 μL of mQ-water, and 5 μL of 1 M NaI in mQ-water were added to reach final concentrations NaI 5 mM and DSAI 0.05 mM.
- The DSAI thermodynamic aggregate was prepared in a vial following the same procedure reported in the previous point. Then, the vial was sealed and incubated in the Fishebrand™ Isotemp™ Water Bath at 95 °C for 2 min. Subsequently, the heating was turned off, allowing the bath to slowly cool down overnight.
- In the case of the DSAI seeded-growth sample, a suspension of 0.5 mM DSAI in mQ-water was prepared in Eppendorf® tube. 98 μL was transferred to a vial, then 895 μL of mQ-water, and 5 μL of 1 M NaI mQ-water were added. The vial was sealed and incubated in the Fishebrand™ Isotemp™ Water Bath at 95 °C for 2 min. Subsequently, the heating was turned off, allowing the bath to cool down naturally. The remaining 2 μL was added at a bath temperature of 50 °C (molar ratio 49:1 between dissolved DSAI molecules and DSAI seeds) to reach final concentrations of NaI 5 mM and DSAI 0.05 mM. Subsequently, the slow cooling process was continued overnight.

- DSACl with NaCl (0.05 mM and 1 M, respectively) was prepared in a vial by adding 1.1 mL of mQ-water, followed by 0.4 mL of 5 M NaCl to a 0.5 mL solution of 0.2 mM in DSACl previously dissolved in mQ-water. The sample was stirred for 15 min.
- DSACl with NaI (0.05 mM and 3 mM, respectively) was prepared in a vial by adding 1.5 mL of mQ-water, followed by 6 µL of 1 M NaCl to a 0.5 mL solution of 0.2 mM in DSACl previously dissolved in mQ-water. The sample was stirred for 5 min.
- DSABr with NaBr (0.05 mM and 0.03 M, respectively) was prepared in a vial by adding 1.5 mL of mQ-water, followed by 12 µL of 5 M NaBr to a 0.5 mL solution of 0.2 mM in DSABr previously dissolved in mQ-water. The sample was stirred for 15 min.
- $DSANO_3$ with $NaNO_3$ (0.05 mM and 0.1 M, respectively) was prepared in a vial by adding 1.5 mL of mQ-water, followed by 40 µL of 5 M $NaNO_3$ to a 0.5 mL solution of 0.2 mM in $DSANO_3$ previously dissolved in mQ-water. The sample was stirred for 15 min.
- $DSAPF_6$ samples were prepared either by sonication or nanoprecipitation. Sonicated samples were prepared by weighting $DSAPF_6$ and adding mQ-water, followed by sonication for 10 minutes. For the nanoprecipitated sample, a solution of $DSAPF_6$ in DMSO (5 mM) was prepared and then 0.1 mL of this solution was added to a new vial containing 1.9 mL of mQ-water and under stirring. The sample was stirred for 60 min.
- In the case of TBATPE, a 0.5 mM stock solution in mQ-water was prepared in the presence of 4.4 equivalents of NaOH in Eppendor® tube. The dissolution of TBATPE was aided by sonication for 30 min. 200 µL was transferred to a new Eppendorf® tube, then 750 µL of mQ-water, and 50 µL of 1 M AA were added to reach the final concentrations of TBATPE 0.1 mM and AA 5 mM.

Transmission electron microscopy (TEM) was performed using TEM JEOL F_{200} operated at 200 kV, equipped with a cold-FEG source. Samples were deposited on the grid (carbon-supported copper grids, 400 mesh size or 170 mesh size), then washed with mQ-water to remove dissolved salts, and then blotted with filter paper. Images were analyzed using Image J software.[4] Elemental analysis and mapping were performed using a JEOL 100 mm^2 silicon drift energy dispersive X-ray spectrometer (EDX).

Scanning electron microscopy (SEM) was performed with a Zeiss Sigma HD microscope, equipped with a Schottky FEG source at 5 kV power and an InLens detector. Sample preparation involved drop-casting of samples on silicium plates.

Atomic force microscopy (AFM) was recorded with an Agilent 5500 Scanning Probe Microscope equipped with an Agilent N9521A scanner. The measurements were performed in tapping mode using cantilever C (force constant: 7 N/m) of MikroMash XSC11 Series. A small volume (50 µL) of sample was placed on mica after peeling it off with tape. It was allowed to dry, then 50 µL of mQ-water was placed on the mica to wash away dissolved salts, and then blotted with filter paper. The mica was allowed to dry before imaging. Images were analyzed using Gwyddion software.[5]

Widefield fluorescence microscopy (WFM) was performed on an Axio Observer 7 inverted microscope using Colibri 5 (385 nm irradiation, RGB-UV), without reference color, and 63× oil immersion objective. Samples (20 µL) were deposited on a coverslip, blotted to remove the solvent excess, and then imaged.

Dynamic light scattering (DLS) analysis was performed on a Malvern instrument using a 1.5 mL cuvette (filled with about 1 mL) at 25 °C, selecting 173° as back angle for the measurement, viscosity of the solvent η = 0.8872, material refractive index = 1.59, dispersant refractive index = 1.33, 2 min equilibration time, 3 measurements for each sample.

3D Electron Diffraction. Electrons feature very strong interactions with the electrostatic potential of atoms. Subsequently, electron diffraction allows for performing experiments with crystallites in the nanometer range. However, it needs to be considered that the absorption of the samples is much stronger and the data are affected by dynamical diffraction as well as ionic scattering factors, compared to X-ray diffraction. This can lead to seemingly bad R-values for the refinement in the simplistic kinematic approximation.

Microcrystalline powders of both kinetic and thermodynamic aggregates of DSAI were first ground-up between microscope slides. The yellowish powder of each aggregate was then spread on a standard lacey-carbon-coated 200 mesh copper TEM grid. Yellow plate-like crystallites with a few 100 nm thickness were selected for 3D ED/microED measurements in both cases giving the same structural results. The best dataset was collected on the thermodynamic aggregates crystallized from an DMF/EtOH mixture via slow evaporation at ambient temperature.

Electron diffraction measurements for DSAI were collected using the Rigaku XtaLAB Synergy-ED, equipped with a Rigaku HyPix-ED detector optimized for operation in the continuous rotation 3D ED experimental setup.[6,7] Data acquisition was performed at ambient temperature under high vacuum with an electron wavelength of 0.0251 Å (200 kV). The instrument was operated and the diffraction data were processed in the program CrysAlis^{Pro}.[8] The structure was solved using ShelXT,[9] and subsequently, refined with kinematical approximation using ShelXL[10] in the crystallographic program suite Olex2.[11,12] By merging data of four individual grains/datasets of DSAI, a completeness of 98.0% up to a resolution of 0.83 Å was achieved. Non-hydrogen atoms were assigned anisotropic displacement parameters. The hydrogen atoms were placed in idealized positions and included as riding. Isotropic displacement parameters for all H atoms were constrained to multiples of the equivalent displacement parameters of their parent atoms with Uiso(H) = 1.2 Ueq(parent atom). Enhanced rigid bond restraints[13,14] with standard uncertainties of 0.001 Å2 as well as a few distance restraints (DFIX) were applied. The experimental and refinement details are given in Tables S1, S2, and S3. CCDC 2485494 contains the supplementary crystallographic data for this publication. These data can be obtained free of charge via www.ccdc.cam.ac.uk/data_request/cif, or by emailing data_request@ccdc.cam.ac.uk, or by contacting The Cambridge Crystallographic Data Centre, 12 Union Road, Cambridge CB2 1EZ, UK; fax: +44 1223 336033.

Computational details. To shed light on the aggregation and photophysical properties of DSAI, the molecular structure of the monomeric and dimeric species of this molecule were investigated by using quantum and classical theoretical calculations. The dimer can be regarded as the smallest aggregate and this approach been successfully used to understand the optical spectra of dye-based aggregates.[15–17] The monomer and dimer structures were extracted from the micro-crystal electron diffraction data and then optimized at the r^{2}SCAN-3c composite method[18,19] in water (SMD method), implemented in ORCA.[20] We also calculated single point energies and time-dependent density functional theory (TD-DFT) using CAM-B3LYP[21] functional (up to 6 states) and 6-31G(d)[22] or LanL2DZ[23] basis set (for C, H, N or I atoms, respectively), using the Gaussian 16 program package.[24] The spectra were extracted either with GaussView 5[25] or GaussSum,[26] assuming a half-width of 0.15 eV for proper simulation. The noncovalent interaction (NCI) method[27] was used to visualize weak interactions in the DSAI dimer, using Multifwn 3.8.[28,29]

Photocatalysis experiments were performed as detailed below:

- **3,3',5,5'-tetramethylbenzidine (TMB) oxidation.** The photooxidation experiments of TMB (TCI, T1023) were performed in quartz cuvette (10 mm path length) in air atmosphere with a PTFE lid. Experiments under O_2 and N_2 atmosphere were performed in quartz cuvettes (10 mm path length) with screw cap and sealed with silicone/PTFE septum (VWR, 13 mm, red/grey, PTFE, 2.0 mm, 50° hardness). The cuvettes were positioned into a homemade built photoreactor presenting a 3D printed sample support and LEDs (High Power LED Star, LEDsupply.com).[30] Each LED is equipped with a focus lens, and the system is cooled through an external fan. The samples were irradiated (white light LEDs, 180 mW·cm^{-2} intensity, measured using an Optical Power Meter PM100D at 450 nm with Optical Sensor S120VC from Thorlabs) for a variable amount of time before being analyzed. The TMB oxidation process was monitored with UV-Vis spectroscopy by focusing on the absorption band of oxidized TMB ($\mathcal{E}_{652}$ = 3.9 × 10^4 M^{-1} cm^{-1}).[31] TMB final concentration in all samples was 134 μM. Error bars denote the standard deviation of the mean, calculated from two runs.
  - DSAX anion screening. Samples of 0.3 mM DSAX (X = Cl, Br, NO_3, I, PF_6) were prepared in 3 mL of acetate buffer (0.1 M at pH = 5) by diluting a stock sample of 0.4 mM DSAX in acetate buffer (previously sonicated for 10 min). Then, 100 μL of TMB (1 mg mL^{-1} in DMSO) was added under stirring. In the case of DSAPF$_6$, 18 mM stock solution in DMSO was also prepared and added (50 μL) to 3 mL acetate buffer solution under stirring. Subsequently, TMB (50 μL, 2 mg mL^{-1} in DMSO) was added. All samples 0.3 mM DSAX were left under stirring at least 1 h at room temperature, transferred to cuvettes, and, before starting the irradiation, they were kept in the dark and under stirring until the aggregation level was stable (monitored via UV-Vis spectroscopy). The recyclability was tested by running 15 min irradiation experiments and filtering the samples after the quantification using polycarbonate membrane filter (0.1 μm pore size) under vacuum. DSAPF$_6$ 0.3 mM nanoprecipitated from DMSO in 3 mL acetate buffer was prepared as reported above. Subsequently, TMB (50 μL, 2 mg mL^{-1} in DMSO/mQ-water, 6:4 *v*/*v*) was added. After each cycle, fresh acetate buffer (3 mL) and TMB (50 μL) were added.
  - DSAI concentration screening**.** Samples of 0.1–0.4 mM DSAI were prepared in 3 mL of acetate buffer (0.1 M at pH = 5) from a suspension of 0.4 mM DSAI

in acetate buffer (previously sonicated for 10 min). Then, 100 μL of TMB (1 mg mL^{-1} in DMSO) was added under stirring. All samples were left under stirring at least 1 h at room temperature, transferred to cuvettes, and, before starting the irradiation, they were kept in the dark and under stirring until the aggregation level was stable (monitored via UV-Vis spectroscopy). Same procedure was used for control experiments where (1) 100 μL of DMSO instead of TMB was added, (2) 0 mM DSAI sample was prepared, and (3) irradiation under white LED was not performed. In the case of experiments under O_2 and N_2 atmosphere, cuvettes containing 0.3 mM DSAI in acetate buffer were also purged for 20 minutes with O_2 or N_2 using steel needles through the septum as inlet (longer needle inside the solution) and outlet (shorter needle in the headspace). After purging for the specified time, the purging was stopped, the long needle was placed in the headspace, and then the short one was removed. Finally, to identify the specific ROS involved in the TMB photooxidation process, 1.0 mg of each scavenger (D-mannitol, tiron, L-tryptophan) was also added to 3.1 mL of 0.3 mM DSAI samples.

- NaI concentration screening. Five vials were prepared by diluting 0, 30, 60, and 90 μL of 20 mM NaI in acetate buffer (0.1 M at pH = 5) with acetate buffer to a total volume of 1.5 mL, followed by addition of 1.5 mL of DSAI suspension (0.2 mM in acetate buffer) under stirring to obtain DSAI samples (0.1 mM) with final NaI concentrations of 0, 0.2, 0.4, and 0.6 mM, respectively. Then, 100 μL of TMB (1 mg mL^{-1} in DMSO) was added to the vials and left under stirring overnight at room temperature. All samples were transferred to cuvettes and, before starting the irradiation, they were kept in the dark and under stirring until the aggregation level was stable (monitored via UV-Vis spectroscopy).
- Annealed DSAI. 1.5 mM DSAI suspension in acetate buffer (0.1 M at pH = 5) was prepared. To prepare the DSAI thermodynamic DSAI aggregate, 400 μL were added to a vial, sealed, and incubated in the Fishebrand™ Isotemp™ Water Bath at 95 °C for 2 min. Then, the heating was turned off, allowing the bath to cool down naturally overnight. The next day, 200 μL was transferred to a new vial, then 2.8 mL of acetate buffer, 1.8 μL of NaI 1 M in mQ-water (to reach final concentrations NaI 0.6 mM and DSAI 0.1 mM), and 100 μL of TMB (1 mg mL^{-1} in DMSO) were added. Same procedure was used to prepare the DSAI seeded-growth sample. However, 396 μL were added to a vial instead

of 400 µL. The remaining 4 µL was added at bath temperature of 50 °C (molar ratio 99:1 between dissolved DSAI molecules and DSAI seeds). In the meantime, to prepare the DSAI kinetic aggregate, a new 1.5 mM DSAI suspension in acetate buffer was prepared. 200 µL was transferred to a new vial, then 2.8 mL of acetate buffer, 1.8 µL of NaI 1 M in mQ-water (to reach final concentrations NaI 0.6 mM and DSAI 0.1 mM), and 100 µL of TMB (1.0 mg mL^{-1} in DMSO) were added. Subsequently, the slow cooling process was continued overnight. All samples were left under stirring at least 1 h at room temperature, transferred to cuvettes, and, before starting the irradiation, they were kept in the dark and under stirring until the aggregation level was stable (monitored via UV-Vis spectroscopy).

- TBATPE. A 0.5 mM TBATPE stock solution in mQ-water was prepared in the presence of 4.4 equivalents of NaOH. The dissolution of TBATPE was helped by sonication for 30 min. Then, 800 µL of the stock was added to a 1.2 mL solution of acetate buffer (0.1 M at pH = 5) to reach the final concentration of 0.2 mM TBATPE and 60 mM acetate buffer. Subsequently, 66.6 µL of TMB (1.0 mg mL^{-1} in DMSO) was added under stirring. All samples were left under stirring at least 1 h at room temperature, transferred to cuvettes, and, before starting the irradiation, they were kept in the dark and under stirring until the aggregation level was stable (monitored via UV-Vis spectroscopy). Same procedure was used for control experiments where (1) 0 mM TBATPE sample was prepared, and (2) irradiation under white LED was not performed. In the case of experiments under N_2 atmosphere, cuvettes containing 0.2 mM TBATPE solution were also purged for 20 minutes with N_2 using steel needles through the septum as inlet (longer needle inside the solution) and outlet (shorter needle in the headspace). After purging for the specified time, the purging was stopped, the long needle was placed in the headspace, and then the short one was removed.

- **H_2O_2 production and detection.** Samples were prepared in 9.0 mL screw cap vials (VWR, 61×16.6 mm, 548-0821A) equipped with a micro stir bar (VWR 3×8 mm, 442-4520). When purging was performed, vials were sealed with silicone/PTFE septum (VWR, 13 mm, red/grey, PTFE, 2.0 mm, 50° hardness, 548-0487A) and cap (VWR, screw cap, ND15, open top, PP, 548-2416A), otherwise, for open air samples the septa were not used. The samples were positioned into a

homemade built photoreactor presenting a 3D printed sample support and LEDs (High Power LED Star, LEDsupply.com). Each LED is equipped with a focus lens, and the system is cooled through an external fan. In this case, we observed that DSAI and NaI led to oxidation of iodide to iodine, therefore we used DSACl aggregates as photosensitizer for photocatalytic H_2O_2 production. The preparation of the sample started with the making of a stock 0.5 mM solution of DSACl in 3-(*N*-morpholino)propanesulfonic acid. Then, 1 mL of this solution (0.50 μmol of photosensitizer) was taken and placed in a 9.0 mL screw cap vial. For aggregated samples, 53 mg (0.90 mmol) of NaCl was added. Vials were then purged for 15 minutes with O_2 by using steel needles through the septum as inlet (longer needle inside the solution) and outlet (shorter needle in the headspace). After purging for the specified time, the purging was stopped, the long needle was placed in the headspace, and then the short one was removed. The vials were illuminated under stirring at 300 rpm using the homebuilt photoreactor made of 415 nm light LEDs with a light intensity of 140 mW cm^{-2} (measured using an Optical Power Meter PM100D at 415 nm with Optical Sensor S120VC from Thorlabs). Error bars indicate standard error of the mean, calculated from two or three runs. Control experiments were conducted to assess the necessity of each component of the system: experiments without (1) 415 nm LED irradiation, (2) oxygen, (3) photosensitizer or (4) sacrificial agent were performed. Hydrogen peroxide concentrations were determined according to modification of a previously reported procedure.[30–32] In detail, in a cuvette, 20 μL of TMB (1.0 mg mL^{-1} in DMSO) was added to 2 mL of acetate buffer solution containing horseradish peroxidase (HRP) enzyme (0.90 mg of HRP enzyme 77332, Sigma in 50 mL of 0.1 M acetate buffer pH = 5). The background was recorded by UV-Vis spectroscopy. Then, 50 μL of the photocatalytic solution are placed into an Eppendorf® tube and centrifuged, recovering the supernatant. Either 1.0, 2.0, 10, or 20 μL of the supernatant were added, and the UV-Vis spectra were recorded. The absorbance intensity at 652 nm was used to determine the concentration of H_2O_2 ($\mathcal{E}_{652} = 3.9 \times 10^4$ M^{-1} cm^{-1}).[31]

- **Glycerol oxidation.** Samples were prepared in 9.0 mL screw cap vials (VWR, 61×16.6 mm, 548-0821A) equipped with a micro stir bar (VWR 3×8 mm, 442-4520). Vials were sealed with silicone/PTFE septum (VWR, 13 mm, red/grey, PTFE, 2.0 mm, 50° hardness, 548-0487A) and cap (VWR, screw cap, ND15, openTop, PP, 548-2416A). The preparation of the sample started with the making

of a 0.1 mM stock solution of DSAI in glycerol 40 mM and TEMPO 7.5 mM. Then, 975 µL of this solution (0.1 µmol of photosensitizer) were taken and placed in a 9.0 mL screw cap vial, where 25 µL of a stock solution of NaI 1 M was added. Vials were then purged for 15 minutes with O_2 by using steel needles through the septum as inlet (longer needle inside the solution) and outlet (shorter needle in the headspace). After purging for the specified time, the purging was stopped, the long needle was placed in the headspace, and then the short one was removed. The vials were then illuminated under stirring at 300 rpm using the homebuilt photoreactor made of white light LEDs with a light intensity of 100 mW cm^{-2} (measured using an Optical Power Meter PM100D at 450 nm with Optical Sensor S120VC from Thorlabs). Error bars indicate the standard error of the mean, calculated from two or three runs. Control experiments were conducted to assess the necessity of each component of the system: experiments without (1) TEMPO, (2) glycerol, (3) white light irradiation, (4) oxygen and (5) photosensitizer were performed.

Glyceraldehyde detection and quantification was performed by high performance liquid chromatography (HPLC) using an Agilent Technologies 1260 Infinity II LC system coupled to diode array detector; the instrument is equipped with Hi-Plex H column 300×7.7 mm. Condition of analysis: [H_2SO_4] = 5 mM in milliQ water as eluent, isocratic gradient, flow rate 0.7 mL·min^{-1}, injected volume 20 µL, temperature 50 °C.

- **Methyl viologen reduction.** The UV kinetic was performed using a sealed cuvette (1 mm path length closed with preassembled closure and septa GPI 13-425) filled with DSAI 1 mM in EDTA (0.1 M, pH = 6.0), and methyl viologen dichloride monohydrate (Sigma Aldrich, 856177) 10 mM. The cuvette and the solutions were degassed for 15 min under N_2 and irradiated *in situ* in the UV spectrophotometer. Spectra were acquired every 10 s until plateau was reached. The reaction kinetic was followed at 603 nm.
- **H_2 evolution.** Samples were prepared in 9.0 mL screw cap vials (VWR, 61×16.6 mm, 548-0821A) equipped with a micro stir bar (VWR 3×8 mm, 442-4520). Vials were sealed with silicone/PTFE septum (VWR, 13 mm, red/grey, PTFE, 2.0 mm, 50° hardness, 548-0487A) and cap (VWR, screw cap, ND15, open top, PP, 548-2416A). The preparation of the sample started with the making of a 0.1 mM stock solution of DSAI in ascorbic acid 1 M, pH = 4.0. Then, 973 µL of this solution (0.1

μmol of photosensitizer) were taken and placed in a 9.0 mL screw cap vial, where 1.6 μL (8.0 nmol) of a suspension of pre-made Pt nanoparticles were added (3 nm particle size, 1000 ppm in H_2O, Sigma Aldrich). 25 μL of a stock solution of NaI 1 M (or lower concentrations for control experiments) was added. Vials were then purged for 15 minutes with N_2 by using steel needles through the septum as inlet (longer needle inside the solution) and outlet (shorter needle in the headspace). After purging for the specified time, the purging was stopped, the long needle was placed in the headspace, and then the short one was removed. Vials were then equilibrated to atmospheric pressure. The vials were then illuminated under stirring at 300 rpm using the homebuilt photoreactor made of white light LEDs with a light intensity of 100 mW cm^{-2} (measured using an Optical Power Meter PM100D at 450 nm with Optical Sensor S120VC from Thorlabs). Error bars indicate the standard error of the mean, calculated from two or three runs. Control experiments were conducted to assess the necessity of each component of the system: experiments without (1) co-catalyst, (2) sacrificial agent, (3) white light irradiation, and (4) photosensitizer were performed. An optimization of the photocatalytic system was performed by changing the DSAI concentration and the PtNPs molar ratio. A comparison between kinetic, thermodynamic and seeded-growth aggregates was also conducted over 4 hours irradiation. Kinetic samples were prepared as described above. Thermodynamic samples were prepared annealing a suspension of 66.6 μL DSAI 1.5 mM in mQ-water; when the samples reached the room temperature, 1.6 μL of PtNPs, 25 μL of a stock solution of NaI 1.0 M and 908 μL of ascorbic acid (1.0 M, pH = 4.0) were added. Seeded-growth samples were prepared annealing 62 μL of DSAI 1.5 mM at 95 °C for 10 minutes. Then, 5 μL of DSAI 0.5 mM were added to the solution at 50 °C and left overnight. At room temperature, 1.6 μL of PtNPs, 25 μL of a stock solution of NaI 1.0 M and 906 μL of ascorbic acid (1.0 M, pH = 4.0) were added.

The recyclability of the samples was tested by running 4 hours experiment (DSAI 0.65 mM, ascorbic acid 1.0 M, pH = 4.0, PtNPs 8% mol, NaI 16.25 mM) and filtrating the samples after the quantification with polycarbonate membrane filter (0.1 μm pore size) under vacuum. Fresh ascorbic acid and PtNPs were added in each cycle.

A 0.5 mM TBATPE stock solution in mQ-water was prepared in the presence of 4.4 equivalents of NaOH. 200 μL of the solution were placed in a 9 mL screw cap

vial, PtNPs 8% mol and 800 µL of ascorbic acid (0.8 M, pH = 4.0) were added. Samples were irradiated under 415 nm LED (140 mW cm^{-2}).

Gas chromatography (GC) experiments were performed on an Agilent Technologies 8860 with autosampler PAL3 coupled with TCD detector at 250 °C. H_2 was detected on the GC equipped with columns PoraPLOT U and molecular sieves, the inlet temperature was at 100 °C, and the Ar carrier gas flow was at 6 mL/min at a pressure of 14 psi. Headspace samples were injected using PAL3 series 2 Autosampler Systems equipped with a gas-tight syringe (SGE autosampler syringe) injecting 100 µL.

Apparent Quantum Yield (AQY). Samples for H_2 apparent quantum yield determination were illuminated for 60 minutes using 450 nm LEDs (High Power LED Star, LEDsupply.com, 19.6 mW cm^{-2}). The illuminated area is 1.77 cm^2. 394 nmol of H_2 were detected after 1 hour of irradiation. The apparent quantum yield of 0.2% was calculated using the following equation:

$$AQY\ (\%) = \frac{2\ \times number\ of\ evolved\ H_2\ molecules}{incident\ photons} \times 100$$

Electron Paramagnetic Resonance (EPR) spectra were acquired on a Bruker ESR5000 operating at a microwave frequency of 9.4 GHz, under dark or *in situ* irradiation either with 450 nm LED or white light LED. The spectra were recorded using ESRStudio at room temperature with 20 mT microwave power, 0.03 mT modulation and 60−120 s sweep time. Methyl viologen reduction kinetic was performed with DSAI 1 mM, MV^{2+} 10 mM in EDTA (0.1 M, pH = 6.0). Experiments with DMPO electron trapping agent were performed using a suspension of DSAI 0.5 mM in EDTA (0.1 M, pH = 6.0) with DMPO 50 mM degassed with O_2 for 5 min.

Electrochemistry was performed on a Pine Research WaveDriver 100 potentiostat at room temperature, employing a standard three-electrode single-compartment cell: glassy carbon electrode (GCE, CH Instruments, *d* = 3 mm) as working electrode, a Pt wire as counter electrode, and Ag/AgCl as reference electrode. DSAI 0.25 mM solution was prepared in aqueous NaOAc 1 M and purged for 10 minutes with argon. The analysis of DSAI as aggregate was conducted by deposition of an ink on the glassy carbon working electrode (ink made of 1.0 mg of Vulcan C and 1.0 mg of DSAI dispersed in 1 mL of water) and left to dry (for 3 hour). CV experiments were performed in water with KCl 1 M as supporting electrolyte, electrode Ag/AgCl as reference, and a Pt electrode as counter electrode.

Cyclic voltammetry and differential pulse voltammetry of $DSAPF_6$ 1 mM were registered in DMF using $TBAPF_6$ as electrolyte (100 mM) and Ferrocene as internal standard (1 mM).

Reduction potential of the excited states were calculated employing the following equation:[33]

$$E_{red}^{*} = E_{\text{red}} + E_{0\text{-}0}(PS^{*}/PS) = 1.39 \text{ V vs Ag/AgCl}$$

The reduction peak potential (Supplementary Fig. 87) was used for E_{red} and $E_{0\text{-}0}(PS^{*}/PS)$ was estimated spectroscopically from the position of the long wavelength tail of absorption spectra (Supplementary Fig. 87). E_{red} is −1.24 V vs Ag/AgCl, while the position of the long wavelength tail is 472 nm.

Oxidation potential of the excited states were calculated employing the following equation:[33]

$$E_{ox}^{*} = E_{\text{ox}} - E_{0\text{-}0}(PS^{*}/PS) = -1.34 \text{ V vs Ag/AgCl}$$

The oxidation peak potential (Supplementary Fig. 87) was used for E_{ox} and $E_{0\text{-}0}(PS^{*}/PS)$ was estimated spectroscopically from the position of the long wavelength tail of absorption spectra (Supplementary Fig. 87). E_{ox} is 1.29 V vs Ag/AgCl, while the position of the long wavelength tail is 472 nm.

2. Supplementary Discussion

Fluorescence lifetimes of DSAX. To assess how supramolecular aggregation affects the excited states, we performed time-correlated single photon counting (TCSPC) to measure fluorescence lifetimes (τ_{avg}) of DSAX solution and aggregates. In all cases, the fluorescence decays show lifetimes in the nanosecond range (Supplementary Figs. 67-70), pointing to formation of singlet excited states. Consistent with the observed reverse Hofmeister effect in emission enhancement, counterion-dependent experiments revealed that charge diffuse ions anions extended the singlet-state lifetime, with the τ_{avg} increasing from 0.97 ns for Cl^- to 1.4 ns for PF_6^- (Supplementary Fig. 66). Increasing the DSAI concentration from 0.1 to 0.4 mM led to a decrease of τ_{avg} from 3.7 to 0.92 ns (Supplementary Fig. 67). Increasing the concentration of NaI from 0.0 to 0.6 mM led to a progressive decrease in τ_{avg}, from 3.7 to 2.6 ns (Supplementary Fig. 68), which we ascribe to a combination of triplet formation (see Main Text) and singlet-singlet annihilation (Supplementary Figs. 69-70).[34] In all cases, the fluorescence decays show lifetimes in the nanosecond range, pointing to formation of singlet excited states.

Electrochemical reversibility. We note that the limited redox reversibility observed in cyclic voltammetry (Supplementary Fig. 87), underscores that electrochemical irreversibility primarily reflects slow electron-transfer kinetics under electrochemical conditions rather than intrinsic instability under photocatalytic turnover. Under photocatalytic operation, rapid donor-mediated quenching and acid-assisted stabilization of radical intermediates[35] most likely prevents side reactions. In more demanding or donor-limited environments such stability may become less effective, potentially exposing structural reorganization or degradation pathways inferred from electrochemical measurements.

Crystal size and photocatalysis. To further examine how crystal size influences photocatalysis, $DSAPF_6$ aggregates were prepared using either sonication or nanoprecipitation. As observed above, the smaller crystals (generated by nanoprecipitation) displayed enhanced photocatalytic activity. However, they also showed distinct photophysical behavior, including greater excited-state availability (Supplementary Fig. 124), indicating a complex interplay between size and excited-state dynamics that governs the photocatalytic performance.

Tetraphenylethylene (TPE) derivative microscopy and photocatalysis. Transmission electron microscopy revealed that TBATPE forms aggregates in acidic

aqueous environments (Extended Data Fig. 1b, Supplementary Fig. 125), and widefield fluorescence microscopy showed that these micron-scale assemblies are emissive under 385 nm excitation (Extended Data Fig. 1c, Supplementary Fig. 126). These features confirm successful dye rigidification upon aggregation, a hallmark of AIE-active systems. Then, we evaluated photocatalytic oxidation of TMB under white-light irradiation. TBATPE aggregates promote light-driven TMB oxidation under air (v_0 = 3.8 μM min^{-1}), with negligible activity in the absence of light or O_2 (Extended Data Fig. 1d, Supplementary Fig. 127), corroborating the generation of reactive oxygen species via a photoinduced reductive pathway. Similarly, in the presence of Pt nanoparticles and ascorbic acid, TBATPE aggregates also catalyzed the evolution of H_2 under white-light irradiation, with cumulative yields up to 5100 μmol g^{-1} (Extended Data Fig. 1e, Supplementary Fig. 128) after 24 hours. This confirms that both photoreactions can be accessed with same supramolecular system via aggregation-induced excited-state activation. Fluorescence spectroscopy showed that the emission quantum yield increased dramatically upon aggregation, from Φ_{FL} = 1.9% in water (deprotonated molecules in solution) to 51% in acetate buffer and 60% in ascorbic acid (Extended Data Fig. 1f). The trend is reminiscent of the one for the DSAI system, reinforcing the link between supramolecular aggregation and enhanced photocatalysis. These results mirror the functional behavior of DSAI aggregates, indicating that observation of AIP could be a modular design strategy. Even in a non-planar, non-linear chromophores like TBATPE, aggregation to amorphous instead of crystalline structures (Supplementary Fig. 129) is sufficient to unlock both fluorescence and photocatalytic function. This supports the central premise of AIP that the monomer rigidification (or restricted intramolecular motion) through aggregation can activate otherwise inactive dyes, enabling multifunctional light-driven reactivity across chemically diverse systems.

3. Synthetic Procedures

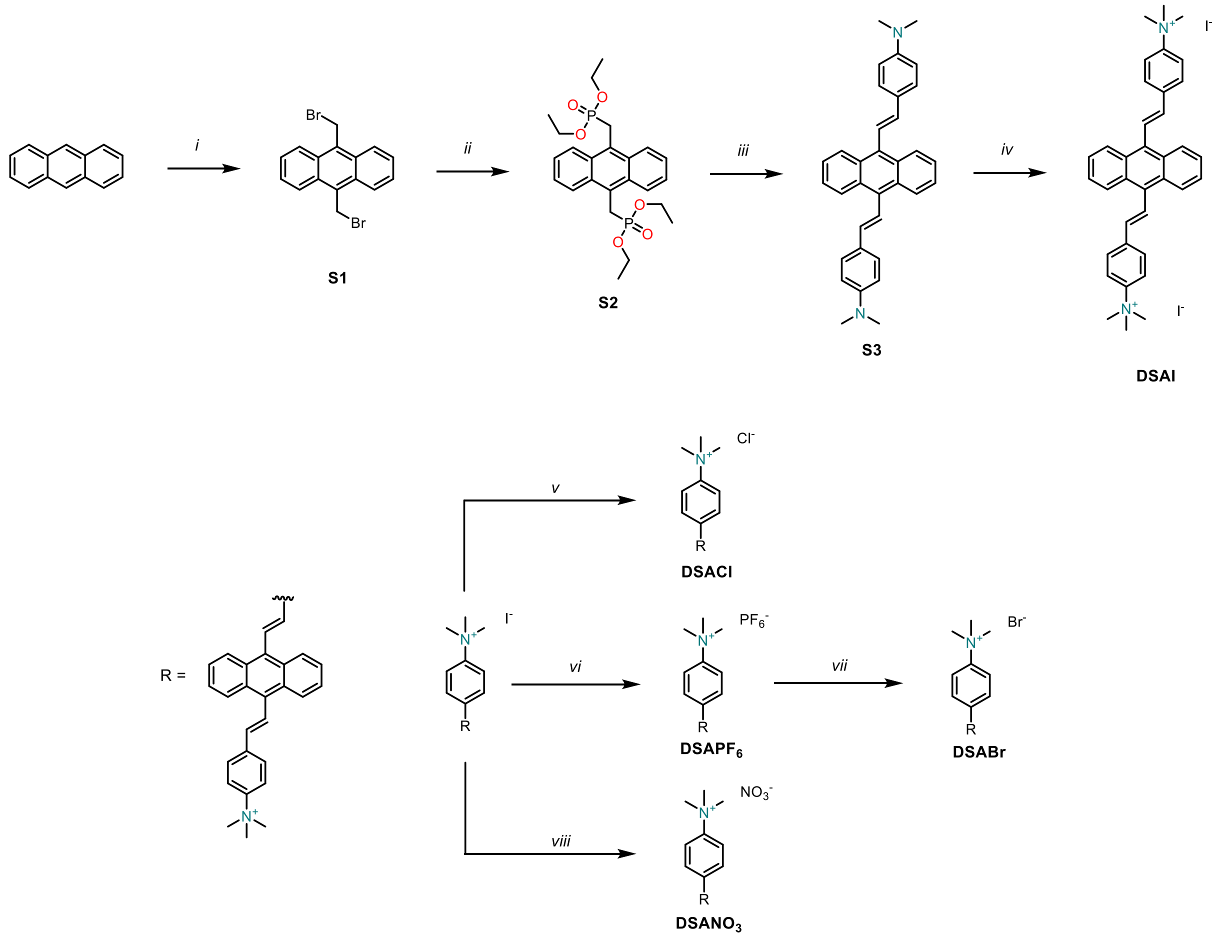

Supplementary Scheme 1. Overview of the synthetic procedures followed to obtain DSAX derivatives. Conditions: *i*) $(CH_2O)_n$, HBr 33% in CH_3COOH, $AlCl_3$, 50 °C, 3 h; *ii*) $P(OEt)_3$, 150 °C, overnight; *iii*) 4-dimethylaminobenzaldehyde, dry THF, under Ar, *t*-BuOK, 0 °C; then r.t. for 4 h. *iv*) CH_3I, $CHCl_3$, 50 °C for 5 days; *v*) Amberlite™ IRA-900 Cl-form, CH_3OH, r.t. for 1 day; *vi*) $AgPF_6$, CH_3OH, r.t., 3 h; *vii*) tetrabutylammonium bromide, acetone, r.t. for 15 minutes *viii*) $AgNO_3$, CH_3OH/H_2O, r.t., 1 h.

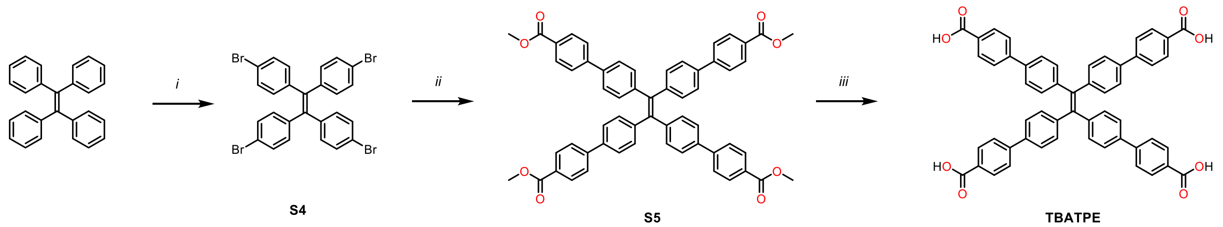

Supplementary Scheme 2. Overview of the procedure to obtain TBATPE. Conditions: *i*) Br_2 at 0 °C for 20 minutes, CH_3COOH then CH_2Cl_2 at 50 °C for 30 minutes; *ii*) 1,1,2,2-tetrakis(4-bromophenyl)ethylene, 4-methoxycarbonylphenylboronic acid, $Pd(PPh_3)_4$, CsF, 1,2-dimethoxyethane, under Ar and under reflux for 2 days; *iii*) NaOH in $THF/CH_3OH/H_2O$ at reflux overnight, then HCl.

Synthesis of 9,10-bis-bromomethylanthracene (S1)

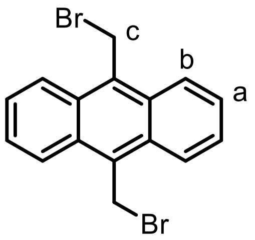

Compound synthesized according to literature procedures.[36] Anthracene (6.36 g, 35.62 mmol), paraformaldehyde (4.38 g, 142.5 mmol) and hydrobromic acid (33% in acetic acid, 50 mL) were added to a 250 mL round-bottom flask. A catalytic amount of $AlCl_3$ was added to the solution and the reaction mixture was heated at 50 °C for 3 h. A yellow precipitate was obtained. The reaction mixture was left to cool down at room temperature and water (75 mL) was added. The yellow solid was filtered and washed with water several times until the water exiting the filter was no longer acid. The solid residue was recrystallized in toluene and dried to obtain yellow crystals (7.99 g, 62% yield).

m.p.: 174−176 °C. **^{1}H NMR** (400 MHz, Chloroform-*d*) δ = 8.40 (dd, J = 6.9, 3.3 Hz, 4H, H_b), 7.71 (dd, J = 6.9, 3.2 Hz, 4H, H_a), 5.54 (s, 4H, H_c). **^{13}C NMR** (101 MHz, Chloroform-*d*) δ = 126.7, 124.4, 26.6. **IR** (FTIR, *v* max) cm^{-1} = 3082, 1619, 1439, 1194, 778, 762, 665, 596. **APCI(+)-MS** (DCM): 204 $[M – 2CH_2Br]^+$, 285 $[M – CH_2Br]^+$.

Synthesis of 9,10-bis(diethylphosphonomethyl)anthracene (S2)

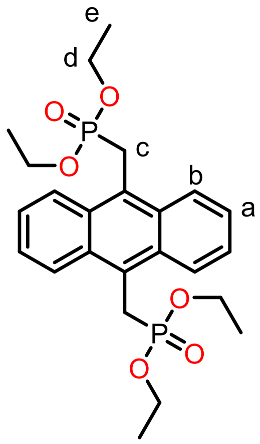

Compound synthesized according to a modified literature procedure.[36] Triethyl phosphite (58 mL) and 9,10-bis-bromomethylanthracene (7.0 g, 19.2 mmol) were added to a 250 mL round-bottom flask. The reaction mixture was heated at 150 °C overnight and it was left to cool down at room temperature. The solution was concentrated by evaporating the solvent until the volume was reduced to about 5 mL. Water (about 200 mL) was added to the solution and the mixture was cooled down at 0 °C. The precipitate was filtered and washed with water. After drying in the oven, the product was obtained as a pale yellow solid (6.22 g, 68% yield).

m.p.: 128−130 °C. **^{1}H NMR** (400 MHz, Chloroform-*d*) δ = 8.33 (dd, *J* = 6.8, 3.3 Hz, 4H, H_b), 7.53 (dd, *J* = 7.0, 3.2 Hz, 4H, H_a), 4.17 (d, *J* = 20.3 Hz, 4H, H_c), 3.93–3.69 (m, 8H, H_d), 1.03 (t, *J* = 7.1 Hz, 12H, H_e). **^{13}C NMR** (101 MHz, Chloroform-*d*) δ = 130.2, 130.2, 125.7, 125.5, 124.2, 124.2, 124.2, 62.2, 62.1, 62.1, 28.0, 28.0, 26.6, 26.6, 16.3, 16.2, 16.2. **IR** (FTIR, *v* max) cm^{-1} = 2983, 2904, 1623, 1430, 1366, 1252, 1057, 1025, 965, 943, 810, 771, 747, 717. **ESI(+)-MS** (ACN 99%, HCOOH 1%): 501 $[M + Na]^{+}$.

Synthesis of 9,10-bis(*p*-dimethylaminostyryl)anthracene (S3)

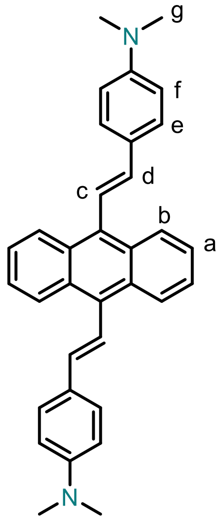

Compound synthesized according to a modified literature procedure.[37] In a 250 mL round-bottom flask, 9,10-bis(diethylphosphonomethyl)anthracene (2.36 g, 4.93 mmol) and 4-dimethylaminobenzaldehyde (1.75 g, 11.73 mmol) were dissolved in anhydrous THF (100 mL) under Ar. The solution was cooled down at 0 °C and *t*-BuOK (4.50 g, 40.1 mmol) was added to the flask. An orange solid precipitated and the reaction was stirred for 4 h at room temperature. The reaction was cooled down at 0 °C and quenched with methanol. The solid was filtered, washed with methanol and dried in oven (2.27 g, 98% yield).

m.p.: >220 °C. **^{1}H NMR** (400 MHz, Chloroform-*d*) δ = 8.45 (dd, *J* = 6.8, 3.4 Hz, 4H, H_b), 7.75 (d, *J* = 16.4 Hz, 2H, H_d), 7.61 (d, *J* = 8.4 Hz, 4H, H_e), 7.46 (dd, *J* = 6.9, 3.3 Hz, 4H, H_a), 6.91–6.81 (m, 6H, H_{f+c}), 3.06 (s, 12H, H_g). **^{13}C NMR** (101 MHz, Chloroform-*d*) δ = 150.4, 137.3, 133.0, 129.7, 127.6, 126.7, 126.0, 124.9, 120.7, 112.6, 40.6. **IR** (FTIR, *ν* max) cm^{-1} = 2977, 2852, 1603, 1521, 1439, 1357, 1184, 1162, 1129, 968, 951, 791, 760. **ESI(+)-MS** (ACN 99%, HCOOH 1%): 235 $[M + 2H]^{2+}$, 469 $[M + H]^+$.

Synthesis of 4,4'-(1*E*,1'*E*)-2,2'-(anthracene-9,1'-diyl)bis(ethene-2,1-diyl)bis(*N*,*N*,*N*-trimethylbenzenaminium) iodide (DSAI)

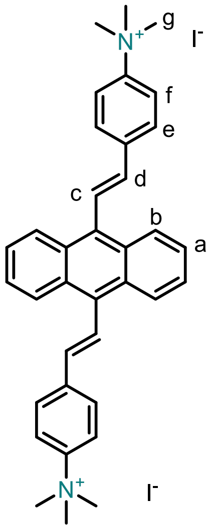

Compound synthesized according to a modified literature procedure.[37] 9,10-bis(*p*-dimethylaminostyryl)anthracene (2.00 g, 4.27 mmol), chloroform (150 mL) and methyl iodide (13.3 mL, 213 mmol) were added to a Schlenk tube and the reaction mixture was stirred at 50 °C for 5 days. The yellow precipitate was isolated via centrifugation and washed with diethyl ether (3×). After drying in the oven overnight, the product was obtained as a bright yellow solid (3.041 g, 95% yield).

m.p.: >220 °C (becomes orange). **^{1}H NMR** (400 MHz, DMSO-d_6) δ = 8.43 – 8.32 (m, 6H, $H_{b+d)}$, 8.14–8.02 (m, 8H, H_{e+f}), 7.60 (dd, *J* = 6.9, 3.3 Hz, 4H, H_a), 7.05 (d, *J* = 16.6 Hz, 2H, H_c), 3.68 (s, 18H, H_g). **^{13}C NMR** (101 MHz, DMSO-d_6) δ = 147.0, 139.0, 135.7, 132.6, 129.3, 128.5, 128.3, 126.7, 126.3, 121.4, 57.0. **IR** (FTIR, *v* max) cm^{-1} = 3020, 1617, 1511, 1486, 1467, 1440, 1396, 1382, 1123, 1011, 980, 952, 927, 841, 755, 722. **ESI(+)-MS** (ACN): 234 $[M - 2CH_3]^{2+}$, 242 $[M - CH_3]^{2+}$, 249 $[M - 2I]^{2+}$, 483 $[M - 2I - CH_3]^+$. **UV-Vis** (DMSO): $\mathcal{E}_{418}$ = (2.08 ± 0.14) × 10^4 M^{-1} cm^{-1}.

Synthesis of 4,4'-(1*E*,1'*E*)-2,2'-(anthracene-9,1'-diyl)bis(ethene-2,1-diyl)bis(*N*,*N*,*N*-trimethylbenzenaminium) chloride (DSACl)

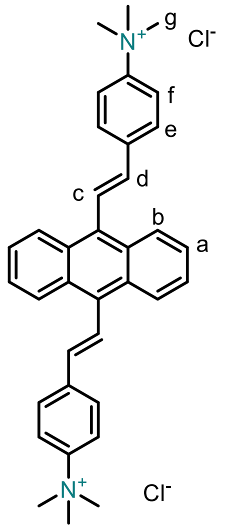

In a 50 mL round-bottom flask, DSAI (250 mg, 0.332 mmol) was dissolved in methanol (16 mL) and the solution was stirred for 24 h with Amberlite™ IRA-900 (3.0 g). The resin was filtered and washed with methanol. The solution was evaporated to obtain an orange solid (187 mg, 98% yield).

m.p.: >220 °C (becomes red). **^{1}H NMR** (400 MHz, DMSO-*d*$_6$) δ = 8.40–8.33 (m, 6H, H_{b+d}), 8.08 (s, 8H, H_{e+f}), 7.60 (dd, *J* = 6.9, 3.3 Hz, 4H, H_a), 7.05 (d, *J* = 16.6 Hz, 2H, H_c), 3.69 (s, 18H, H_g). **^{13}C NMR** (101 MHz, DMSO-*d*$_6$) δ = 147.0, 138.9, 135.7, 132.6, 129.3, 128.5, 128.3, 126.7, 126.3, 121.4, 56.9. **IR** (FTIR, *v* max) cm^{-1} = 3027, 1627, 1511, 1490, 1473, 1439, 1381, 1124, 981, 958, 934, 845, 756. **ESI(+)-MS** (ACN): 234 $[M – 2CH_3]^{2+}$, 242 $[M – CH_3]^{2+}$, 249 $[M – 2Cl]^{2+}$, 483 $[M – 2Cl – CH_3]^{+}$. **UV-Vis** (DMSO): $\mathcal{E}_{418}$ = (1.75 ± 0.21) × 10^4 M^{-1} cm^{-1}.

Synthesis of 4,4'-(1*E*,1'*E*)-2,2'-(anthracene-9,1'-diyl)bis(ethene-2,1-diyl)bis(*N*,*N*,*N*-trimethylbenzenaminium) hexafluorophosphate (DSAPF$_6$)

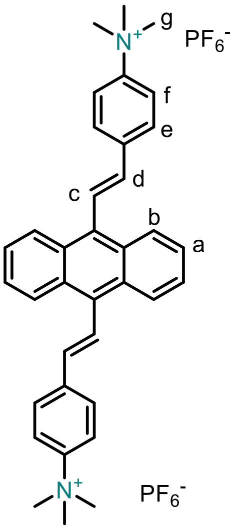

In a 250 mL round-bottom flask, DSAI (98.6 mg, 0.131 mmol) was dissolved in methanol (55 mL). Silver hexafluorophosphate (66.2 mg, 0.262 mmol) solution in methanol (2 mL) was added to the flask. The reaction was stirred for 3 h at room temperature and then it was filtered over celite to remove the AgI precipitate. The filter was washed with acetonitrile (3×). The recovered solution was evaporated, the solid obtained was recovered with water (50 mL), filtered, washed with water and dried. A bright yellow solid was obtained (81 mg, 79% yield).

m.p.: >220 °C (becomes red). ^{1}**H NMR** (400 MHz, DMSO-d_6) δ = 8.43 – 8.31 (m, 6H, H_{b+d}), 8.14–8.02 (m, 8H, H_{e+f}), 7.60 (dd, J = 6.9, 3.3 Hz, 4H, H_a), 7.06 (d, J = 16.6 Hz, 2H, H_c), 3.67 (s, 18H, H_g). ^{13}**C NMR** (101 MHz, DMSO-d_6) δ = 147.0, 139.0, 135.7, 132.6, 129.3, 128.5, 128.3, 126.6, 126.3, 121.4, 57.0. ^{19}**F NMR** (377 MHz, DMSO-d_6) δ −70.1 (d, J = 711 Hz). **IR** (FTIR, v max) cm^{-1} = 3079, 1629, 1509, 1492, 1476, 1380, 1125, 986, 956, 835, 767. **ESI(+)-MS** (ACN): 234 $[M - 2CH_3]^{2+}$, 242 $[M - CH_3]^{2+}$, 249 $[M - 2PF_6]^{2+}$, 483 $[M - 2PF_6 - CH_3]^+$, 643 $[M - PF_6]^+$. **UV-Vis** (DMSO): $\mathcal{E}_{418}$ = (1.82 ± 0.13) × 10^4 M^{-1} cm^{-1}.

Synthesis of 4,4'-(1*E*,1'*E*)-2,2'-(anthracene-9,1'-diyl)bis(ethene-2,1-diyl)bis(*N*,*N*,*N*-trimethylbenzenaminium) bromide (DSABr)

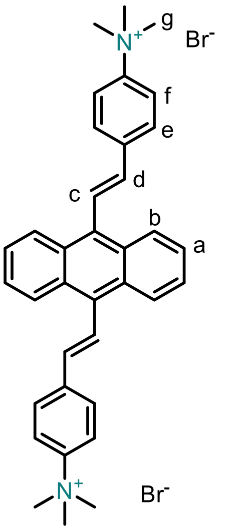

In a 20 mL vial, $DSAPF_6$ (20 mg, 0.025 mmol) was dissolved in acetone (10 mL). Under stirring, a saturated solution of tetrabutylammonium bromide in acetone was added drop by drop to the vial until complete precipitation of the product. The vial was left under stirring for 15 min. The precipitate obtained was separated via centrifugation and washed with acetone (3×). A dark yellow solid was obtained as product (12.9 mg, 79% yield).

m.p.: >220 °C (becomes orange). **^{1}H NMR** (400 MHz, DMSO-d_6) δ = 8.43 – 8.32 (m, 6H, H_{b+d}), 8.14–8.03 (m, 8H, H_{e+f}), 7.60 (dd, *J* = 6.9, 3.3 Hz, 4H, H_a), 7.05 (d, *J* = 16.6 Hz, 2H, H_c), 3.68 (s, 18H, H_g). **^{13}C NMR** (101 MHz, DMSO-d_6) δ = 147.0, 139.0, 135.7, 132.6, 129.4, 128.5, 128.3, 126.7, 126.3, 121.4, 57.0. **IR** (FTIR, *v* max) cm^{-1} = 3022, 1624, 1512, 1489, 1468, 1440, 1382, 1124, 980, 956, 935, 843, 757.721. **ESI(+)-MS** (ACN): 234 $[M – 2CH_3]^{2+}$, 242 $[M – CH_3]^{2+}$, 249 $[M – 2Br]^{2+}$, 483 $[M – 2Br – CH_3]^+$. **UV-Vis** (DMSO): $\mathcal{E}_{418}$ = (1.69 ± 0.21) × 10^4 M^{-1} cm^{-1}.

Synthesis of 4,4'-(1*E*,1'*E*)-2,2'-(anthracene-9,1'-diyl)bis(ethene-2,1-diyl)bis(*N*,*N*,*N*-trimethylbenzenaminium) nitrate (DSANO$_3$)

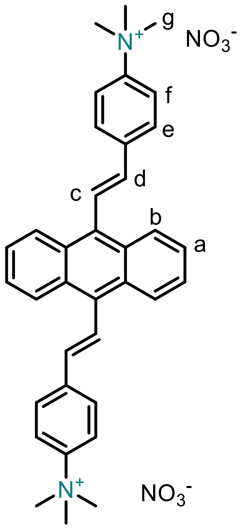

In a 250 mL round-bottom flask, DSAI (100.8 mg, 0.134 mmol) was dissolved in methanol (55 mL). Silver nitrate (44.1 mg, 0.260 mmol) solution in water (2 mL) was added to the flask. The reaction was stirred for 1 h at room temperature and then it was filtered over celite to remove the AgI precipitate. The filter was washed with methanol (2×). The solution was evaporated and a dark yellow solid was obtained (69.7 mg, 84% yield).

m.p.: >220 °C (becomes red). **^{1}H NMR** (400 MHz, DMSO-*d$_6$*) δ = 8.43 – 8.32 (m, 6H, H_{b+d}), 8.14–8.03 (m, 8H, H_{e+f}), 7.60 (dd, *J* = 7.0, 3.2 Hz, 4H, H_a), 7.06 (d, *J* = 16.6 Hz, 2H, H_c), 3.68 (s, 18H, H_g). **^{13}C NMR** (101 MHz, DMSO-*d$_6$*) δ = 147.0, 139.0, 135.7, 132.6, 129.4, 128.5, 128.3, 126.7, 126.3, 121.4, 56.9. **IR** (FTIR, *v* max) cm^{-1} = 3027, 1630, 1512, 1494, 1471, 1385, 1373, 1324, 1119, 978, 957, 938, 846, 757, 723. **ESI(+)-MS** (ACN): 234 $[M - 2CH_3]^{2+}$, 242 $[M - CH_3]^{2+}$, 249 $[M - 2NO_3]^{2+}$, 483 $[M - 2NO_3 - CH_3]^+$, 560 $[M - NO_3]^+$. **UV-Vis** (DMSO): $\mathcal{E}_{418} = (2.02 \pm 0.09) \times 10^4$ M^{-1} cm^{-1}.

Synthesis of 1,1,2,2-tetrakis(4-bromophenyl)ethylene (S4)

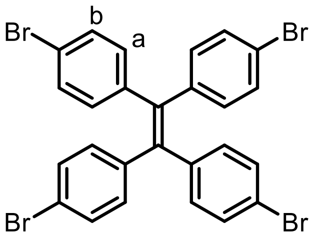

Compound synthesized according to a modified literature procedure.[38] 1,1,2,2-tetraphenylethene (0.50 g, 1.50 mmol) was suspended in acetic acid (3 mL) under an argon atmosphere. After degassing in an ultrasonic bath, the reaction mixture was cooled to 0 °C. At this temperature, bromine (1.92 g, 12.0 mmol) was added over a period of 20 minutes. After the addition of dichloromethane (2 mL), the slurry was stirred at 50 °C for 45 minutes. The mixture was then poured into deionized water. The suspension and washed several times with ethanol and water, and dried. A white solid was obtained as product (0.874 g, 1.35 mmol, 90%).

m.p.: >220 °C. **^{1}H NMR** (400 MHz, DMSO-d_6): δ = 7.39 (d, J = 8.3 Hz, 8H, H_b), 6.92 (d, J = 8.3 Hz, 8H, H_a). **^{13}C NMR** (101 MHz, DMSO-d_6): δ = 141.4, 139.3, 132.7, 131.1, 120.4. **IR** (FTIR, v max) cm^{-1} = 3045, 1904, 1584, 1486, 1396, 1106, 1073, 1006, 863, 817, 793, 736, 503. **APCI(+)-MS** (CH_2Cl_2): 648 $[M]^+$.

Synthesis of tetramethyl 4',4''',4''''',4'''''''-(ethene-1,1,2,2-tetrayl)tetrakis([1,1′-biphenyl]-4-carboxylate) (S5)

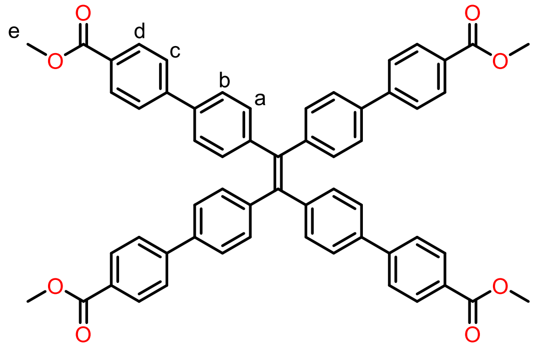

Compound synthesized according to a modified literature procedure.[39] A 250 mL Schlenk tube was equipped with a stir bar and then was dried with a heat gun under vacuum. 1,1,2,2-tetrakis(4-bromophenyl)ethylene (230 mg, 0.36 mmol), 4-methoxycarbonylphenylboronic acid (386 mg, 2,1 mmol), and cesium fluoride (651 mg, 4.3 mmol) were added under Ar. Degassed 1,2-dimethoxyethane (22 mL) was transferred to the Schlenk tube, the system was purged with Ar for 15 minutes, and then tetrakis(triphenylphosphine)palladium (38 mg, 0.034 mmol) was added, followed by Ar purging for 15 minutes. The solution was refluxed for 48 h. After the reaction mixture was cooled to room temperature, the solvent was evaporated, the obtained solid was dissolved in dichloromethane (100 mL) and washed with brine (50 mL, ×2) and water (50 mL). The organic phase was dried over Na_2SO_4, filtered, and concentrated to dryness. The crude product was purified by column chromatography (CH_2Cl_2 100% → 95% CH_2Cl_2, 5% EtOAc). All pure fractions were collected and dried under reduced pressure to give a light yellow solid as product (212 mg, 0.24 mmol, 67%).

m.p.: >220 °C. **^{1}H NMR** (400 MHz, Chloroform-*d*): δ = 8.07 (d, *J* = 8.1 Hz, 8H, H_d), 7.64 (d, *J* = 8.2 Hz, 8H, H_c), 7.45 (d, *J* = 8.1 Hz, 8H, H_b), 7.21 (d, *J* = 8.1 Hz, 8H, H_a), 3.93 (s, 12H, H_e). **^{13}C NMR** (101 MHz, Chloroform-*d*): δ = 167.1, 145.0, 143.6, 140.8, 138.2, 132.2, 130.2, 129.0, 126.9, 126.8, 52.3. **IR** (FTIR, *v* max) cm^{-1} = 3030, 2950, 1928, 1723, 1608, 1432, 1278, 1179, 1107, 1004, 967, 826, 809, 772, 747, 701. **APCI(+)-MS** (CH_2Cl_2): 868 $[M]^+$.

Synthesis of 4’,4’’’,4’’’’’,4’’’’’’’-(Ethene-1,1,2,2-tetrayl)tetrakis(([1,1′-biphenyl]-4-carboxylic acid)) (TBATPE)

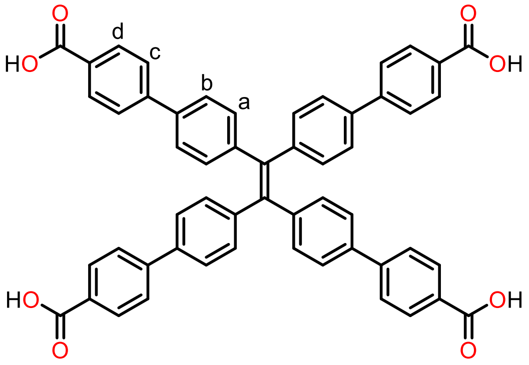

Compound synthesized according to a modified literature procedure.[39] Tetramethyl 4’,4’’’,4’’’’’,4’’’’’’’-(ethene-1,1,2,2-tetrayl)tetrakis([1,1′-biphenyl]-4-carboxylate) (100 mg, 0.12 mmol) and sodium hydroxide (169 mg, 4.2 mmol) were added into a mixture of THF, methanol, and water (15 mL, *v*/*v*/*v* = 1:1:1). The mixture was refluxed overnight. After removal of the organic solvents under reduced pressure, the aqueous phase was acidified with HCl (12 M aq. solution) to yield a yellow precipitate, which was filtered, washed with water, and dried (86 mg, 0.11 mmol, 93%).

m.p.: >220 °C. **^{1}H NMR** (400 MHz, DMSO-d_6): δ = 7.97 (d, *J* = 8.2 Hz, 8H, H_d), 7.78 (d, *J* = 8.2 Hz, 8H, H_c), 7.64 (d, *J* = 8.1 Hz, 8H, H_b), 7.21f (d, *J* = 8.1 Hz, 8H, H_a). **^{13}C NMR** (101 MHz, DMSO-d_6): δ = 167.1, 143.4, 143.1, 140.3, 137.1, 131.6, 130.0, 129.6, 126.54, 126.49. **IR** (FTIR, *v* max) cm^{-1} = 3025, 2654, 2530, 1918, 1688, 1605, 1418, 1397, 1311, 1270, 1176, 1107, 1104, 830, 795, 747, 700. **ESI(–)-MS** (MeOH): 811 $[M - H]^-$. **UV-Vis** (DMSO): $\mathcal{E}_{298}$ = (4.52 ± 0.30) × 10^4 M^{-1} cm^{-1}.

4. Supplementary Figures

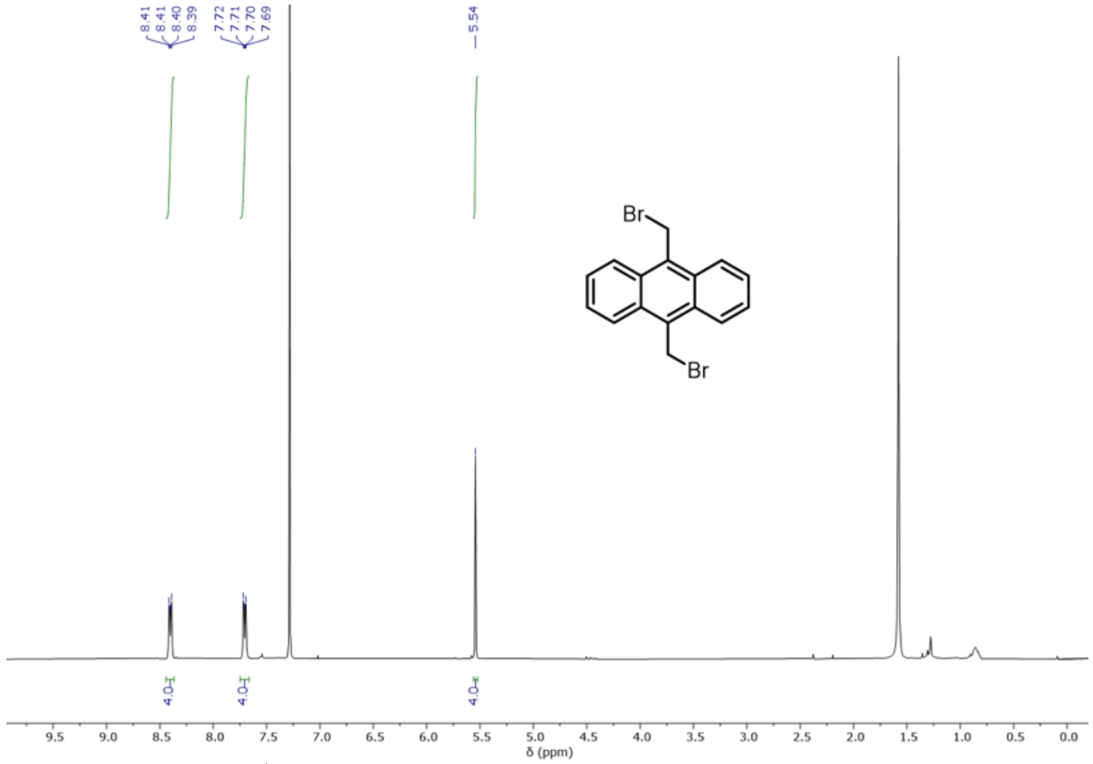

Supplementary Figure 1. ^{1}H NMR (400 MHz, Chloroform-*d*) of 9,10-bis-bromomethylanthracene (S1).

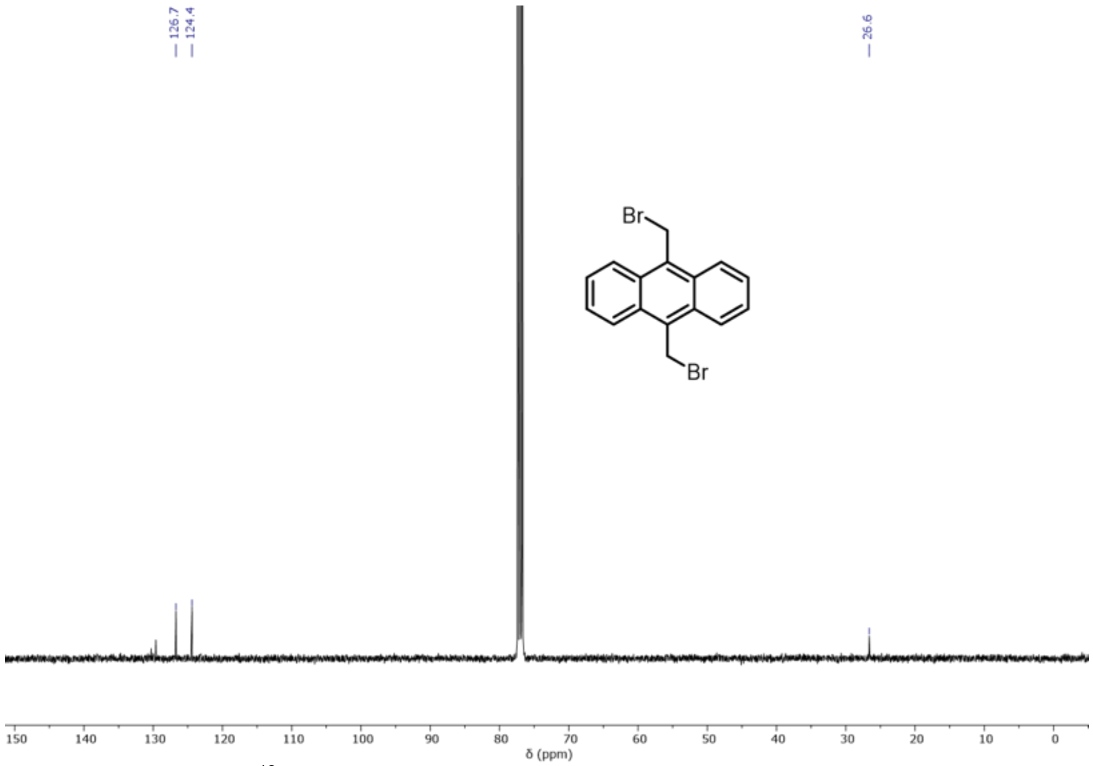

Supplementary Figure 2. ^{13}C NMR (101 MHz, Chloroform-*d*) of 9,10-bis-bromomethylanthracene (S1).

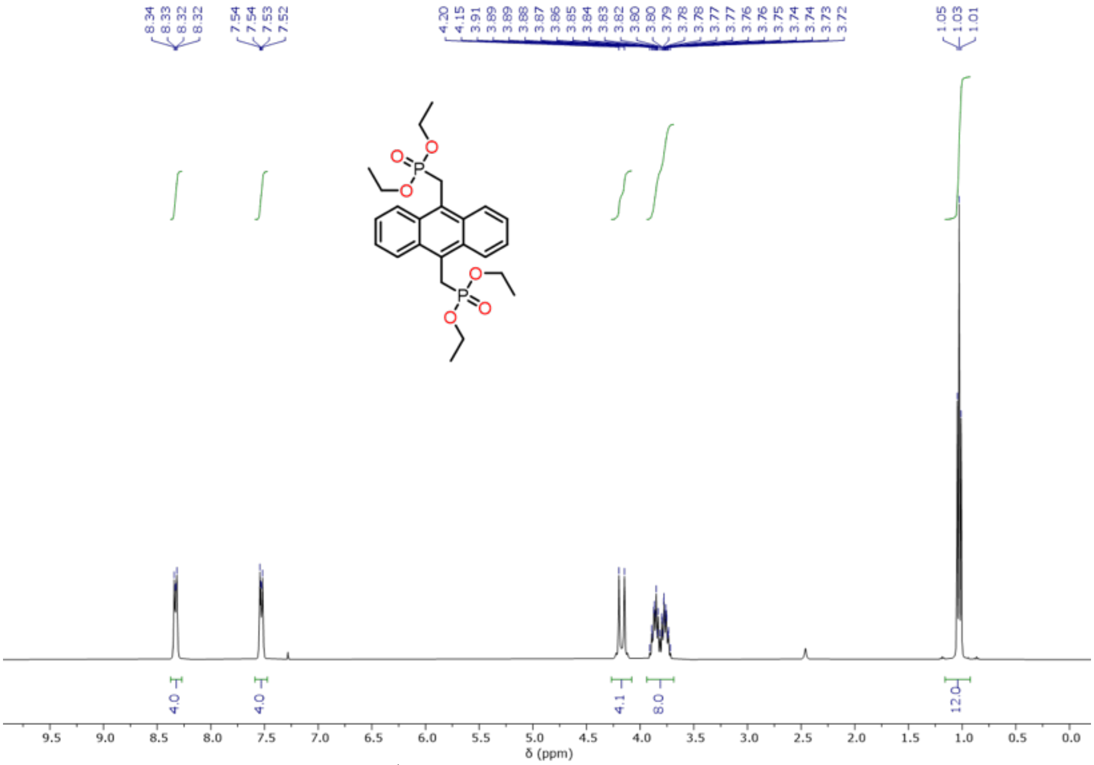

Supplementary Figure 3. ^{1}H NMR (400 MHz, Chloroform-*d*) of 9,10-bis(diethylphosphonomethyl)anthracene (S2).

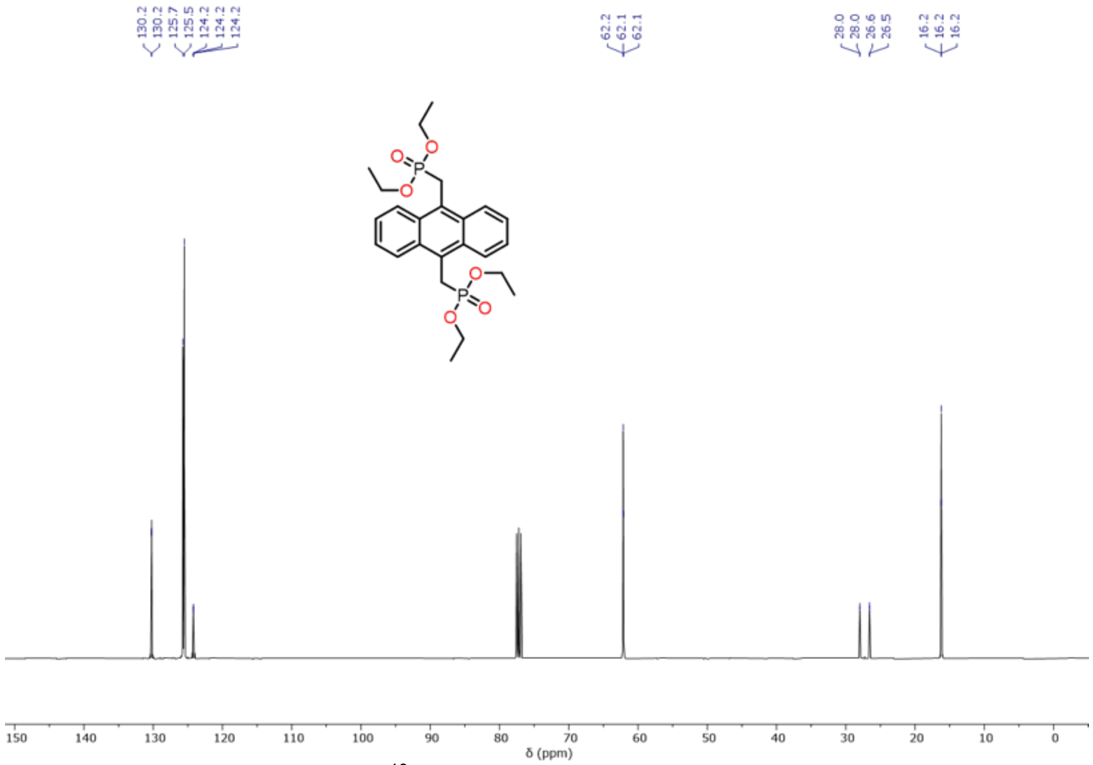

Supplementary Figure 4. ^{13}C NMR (101 MHz, Chloroform-*d*) of 9,10-bis(diethylphosphonomethyl)anthracene (S2).

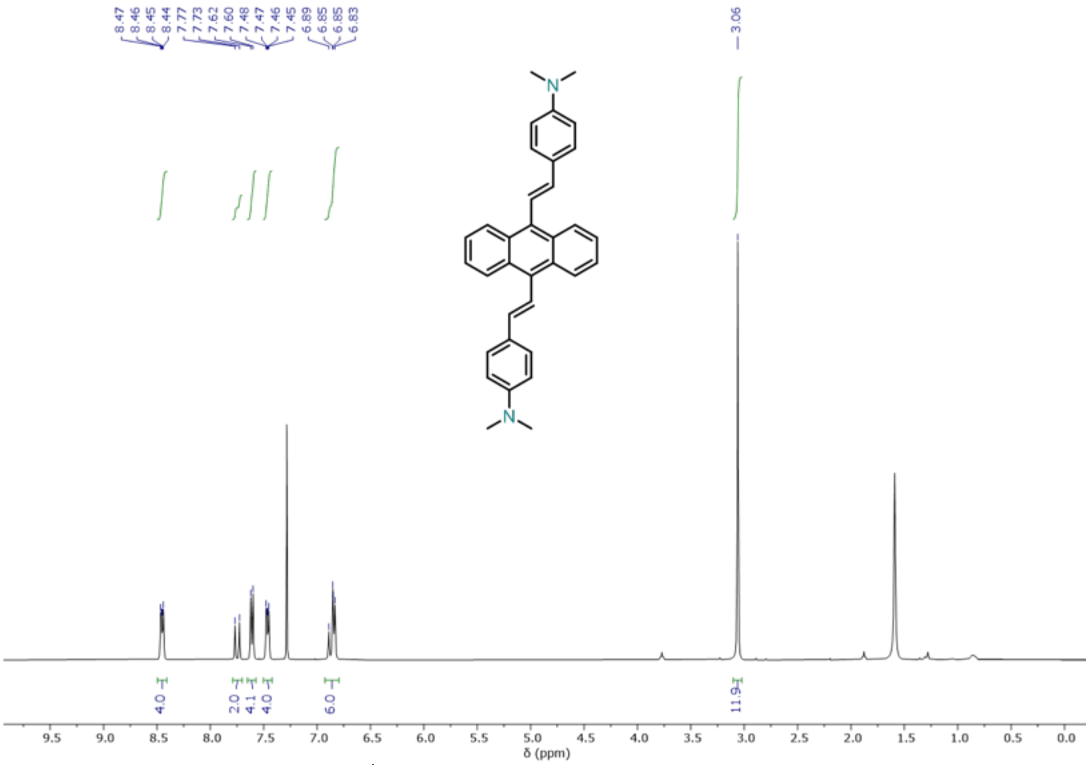

Supplementary Figure 5. ^{1}H NMR (400 MHz, Chloroform-*d*) of 9,10-bis(*p*-dimethylaminostyryl)anthracene (S3).

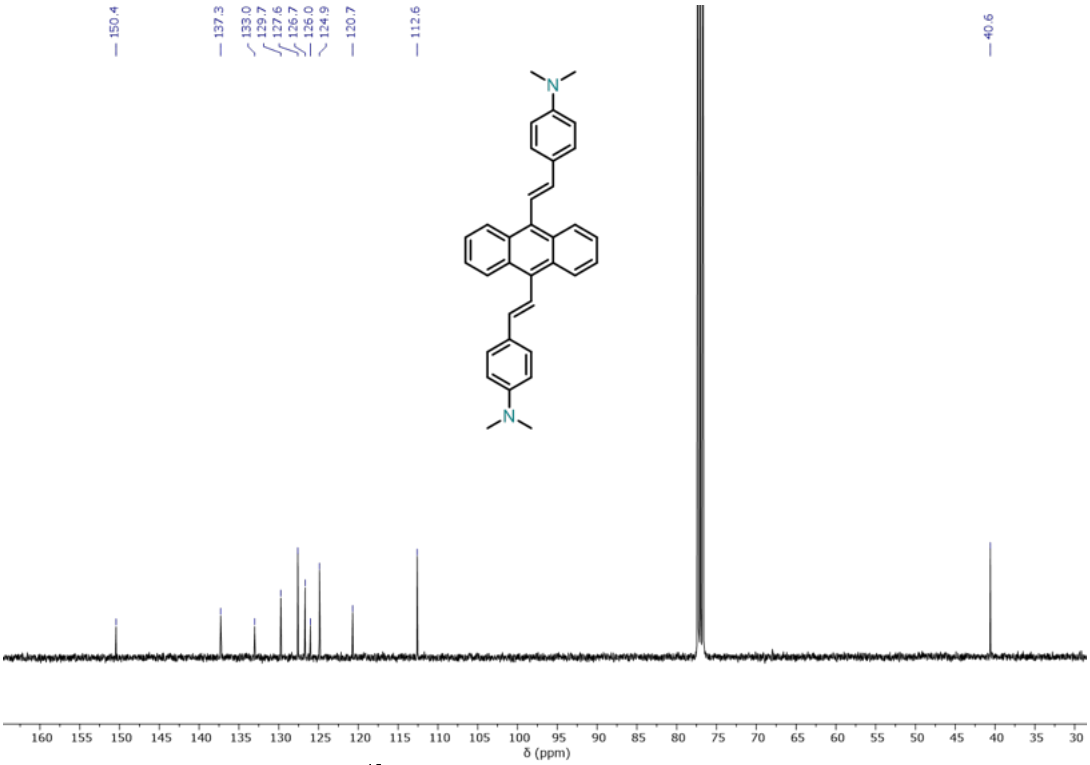

Supplementary Figure 6. ^{13}C NMR (101 MHz, Chloroform-*d*) of 9,10-bis(*p*-dimethylaminostyryl)anthracene (S3).

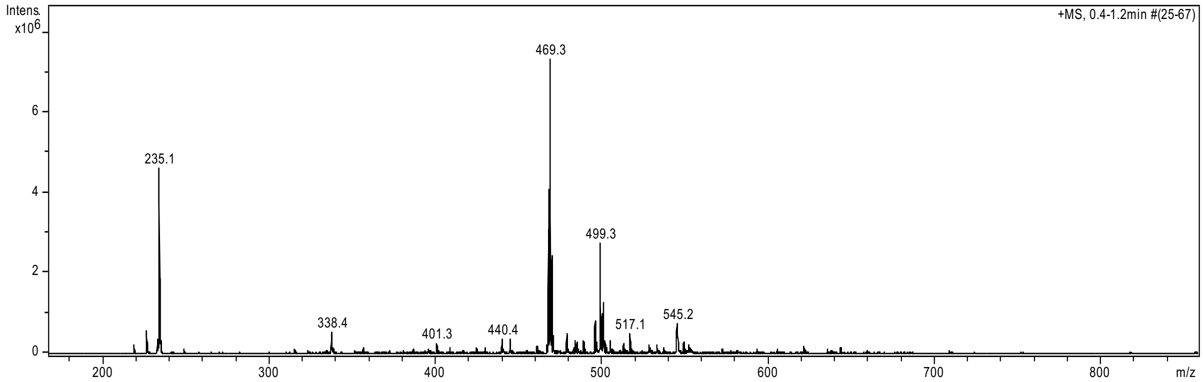

Supplementary Figure 7. ESI(+)-MS spectrum of 9,10-bis(*p*-dimethylaminostyryl)anthracene (S3).

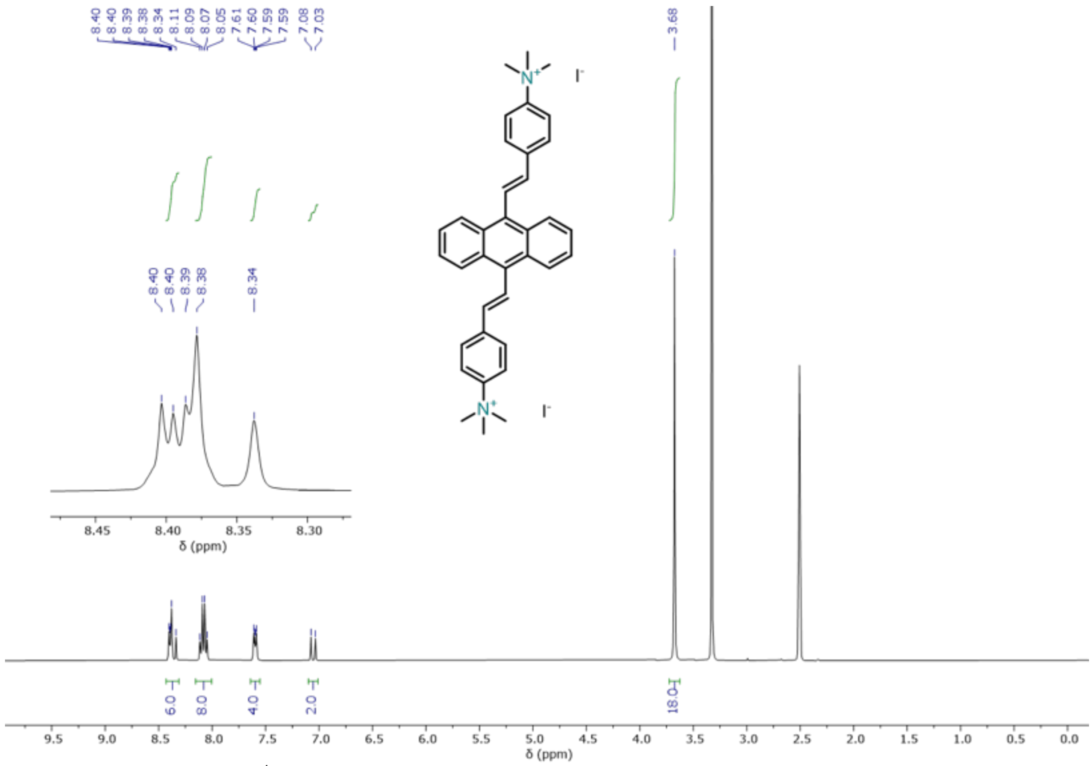

Supplementary Figure 8. ^{1}H NMR (400 MHz, DMSO-d_6) of DSAI.

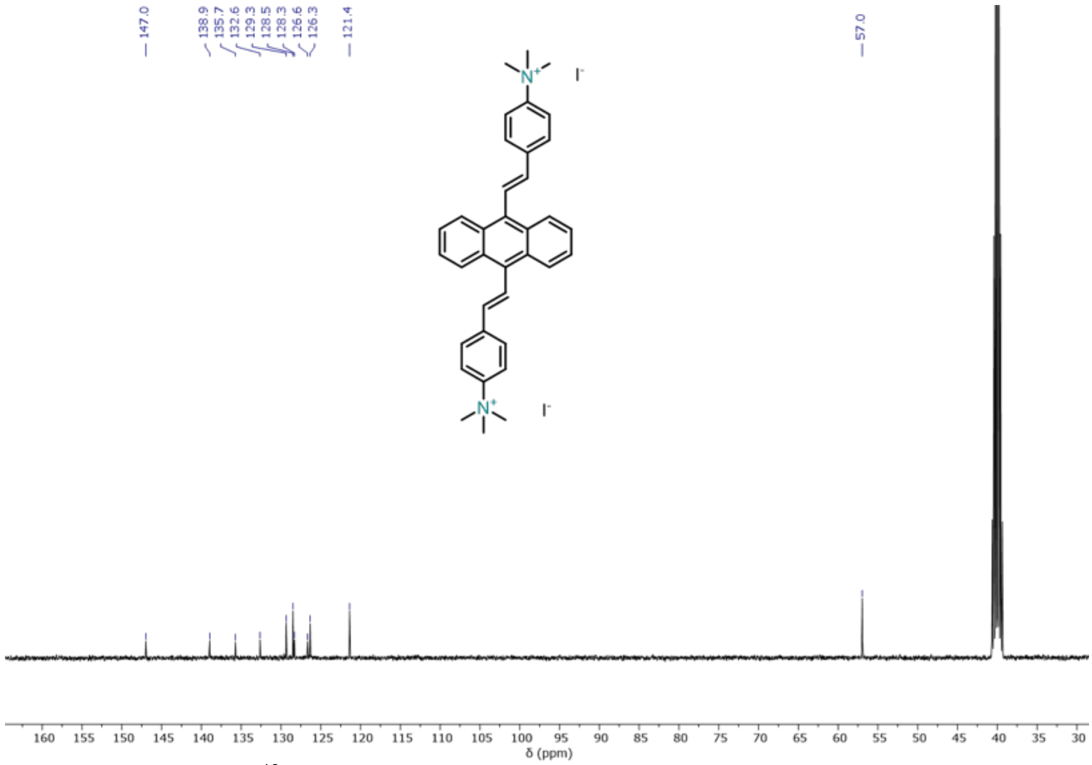

Supplementary Figure 9. ^{13}C NMR (101 MHz, DMSO-d_6) of DSAI.

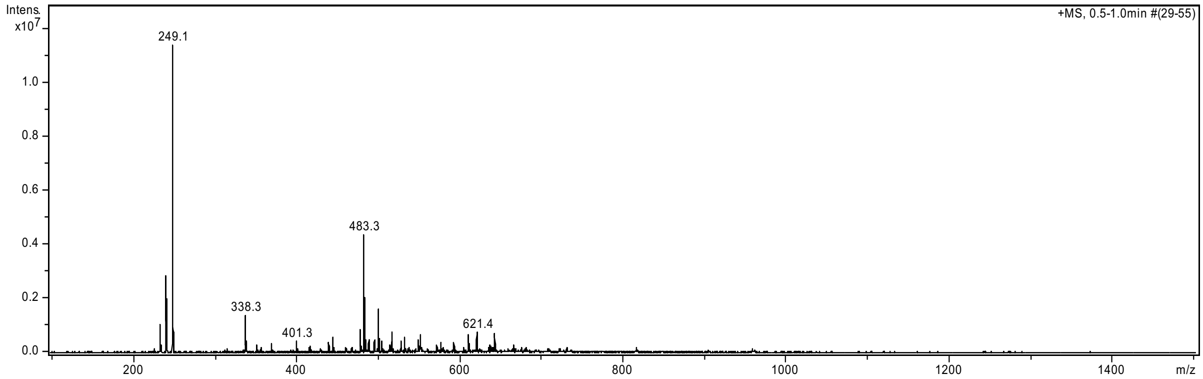

Supplementary Figure 10. ESI(+)-MS spectrum of DSAI.

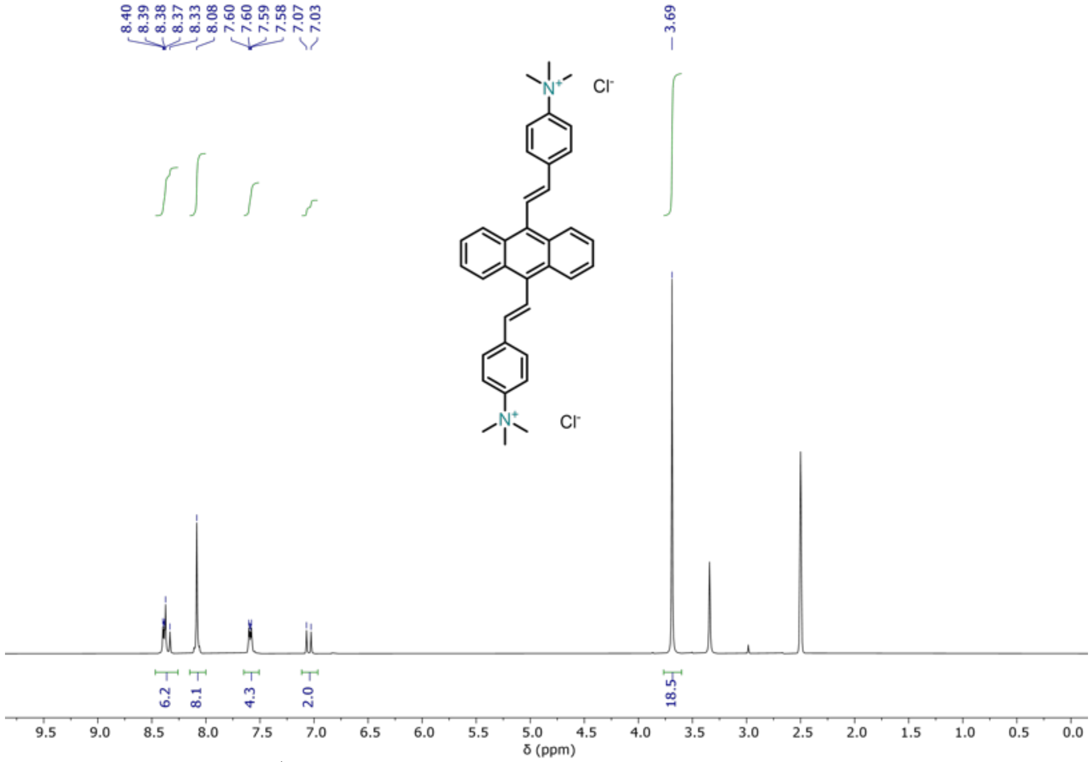

Supplementary Figure 11. ^{1}H NMR (400 MHz, DMSO-d_6) of DSACl.

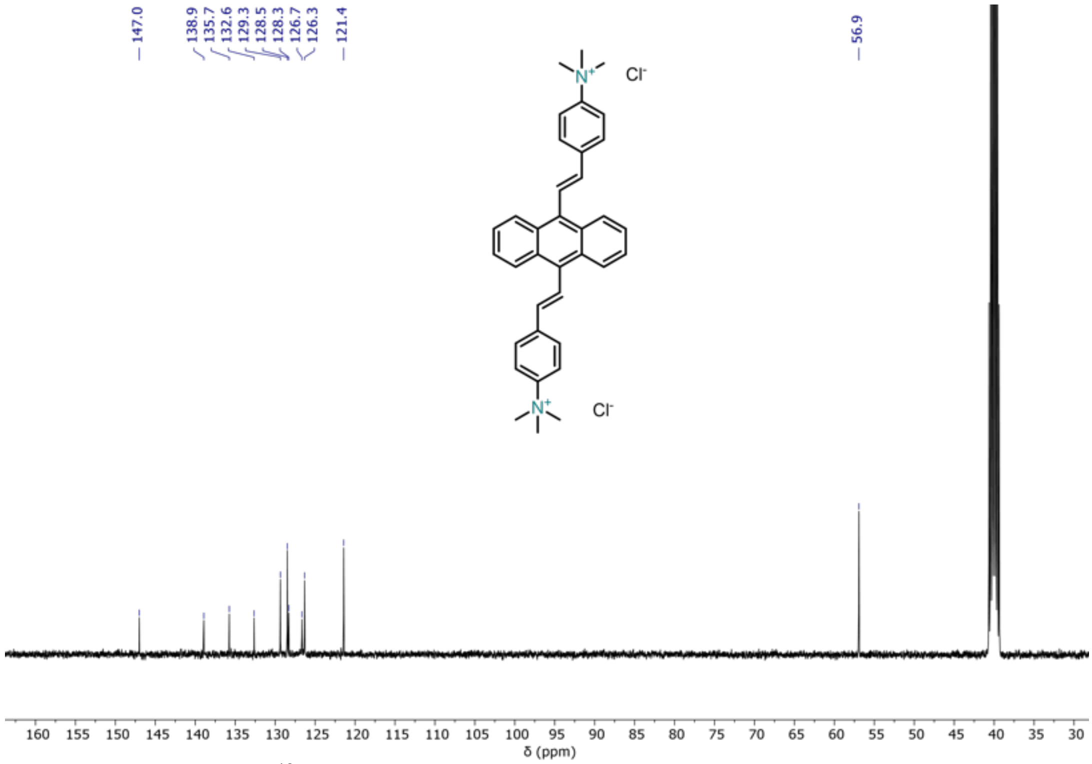

Supplementary Figure 12. ^{13}C NMR (101 MHz, DMSO-d_6) of DSACl.

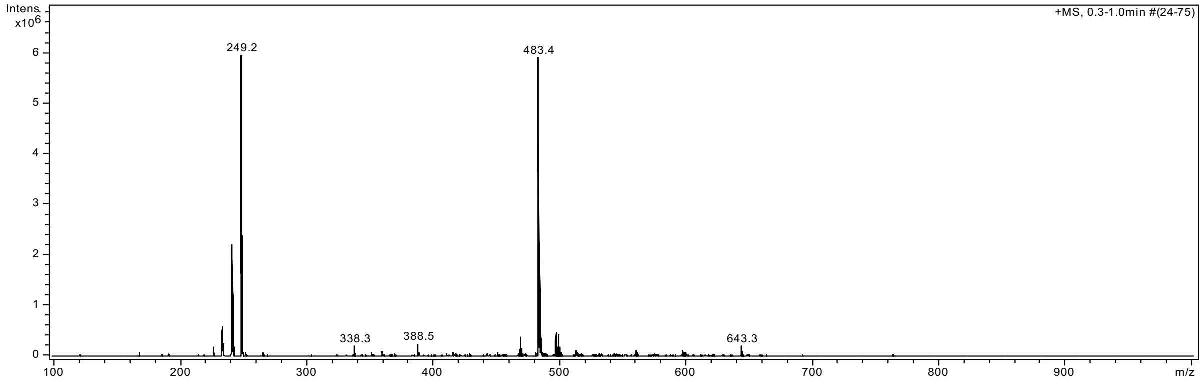

Supplementary Figure 13. ESI(+)-MS spectrum of DSACl.

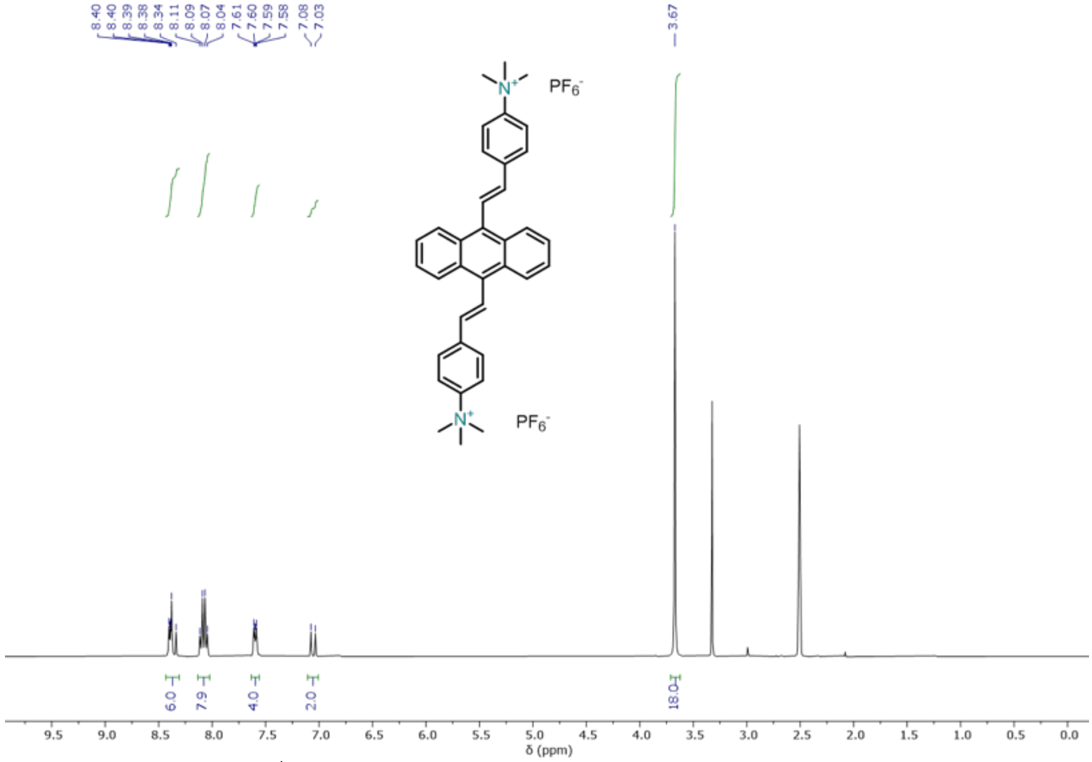

Supplementary Figure 14. ^{1}H NMR (400 MHz, DMSO-d_6) of DSAPF$_6$.

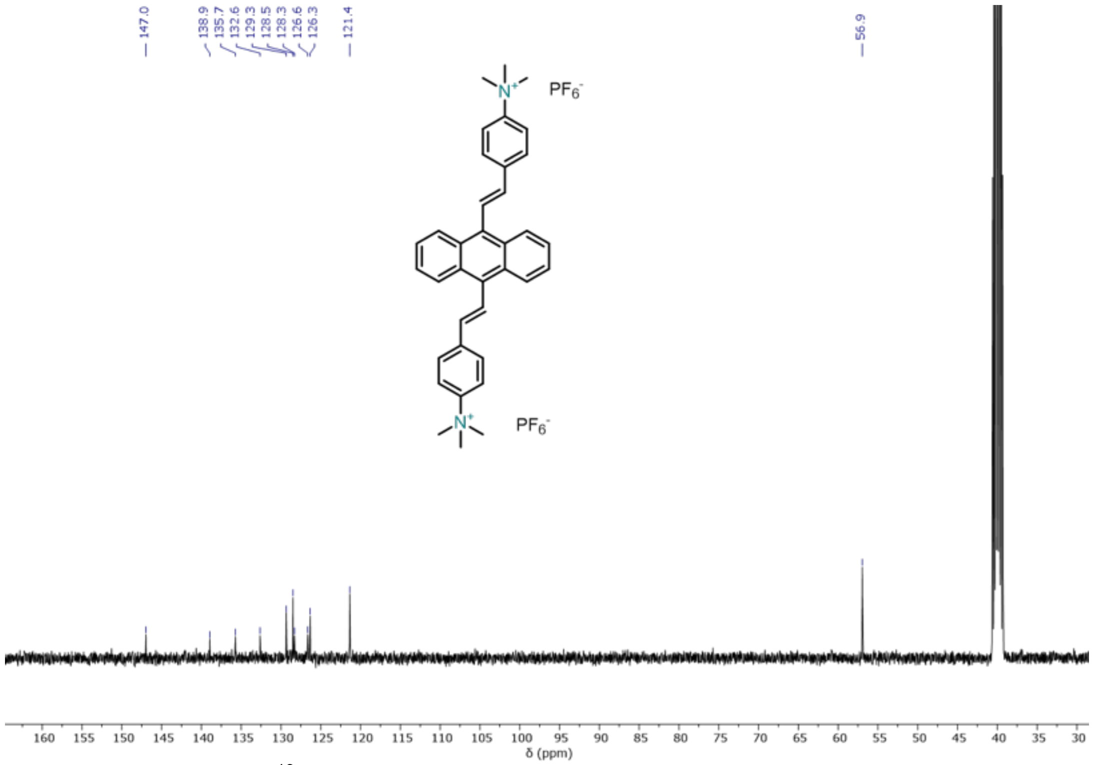

Supplementary Figure 15. ^{13}C NMR (101 MHz, DMSO-d_6) of DSAPF$_6$.

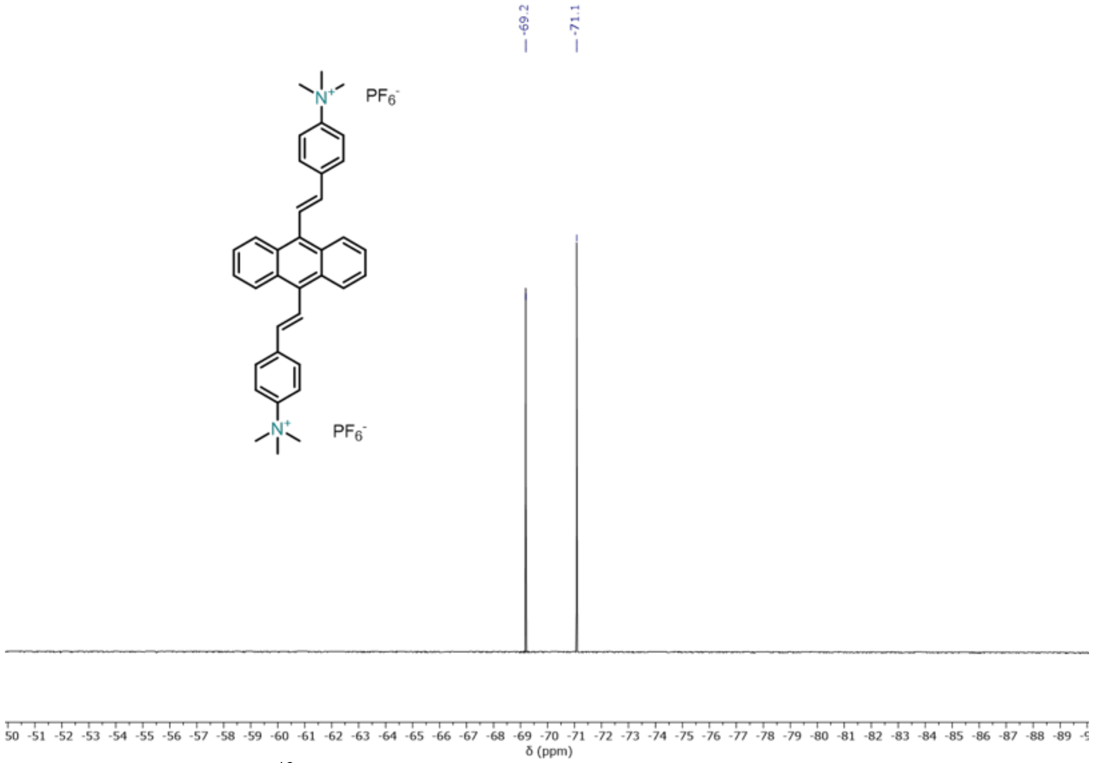

Supplementary Figure 16. ^{19}F NMR (377 MHz, DMSO-d_6) of DSAPF$_6$.

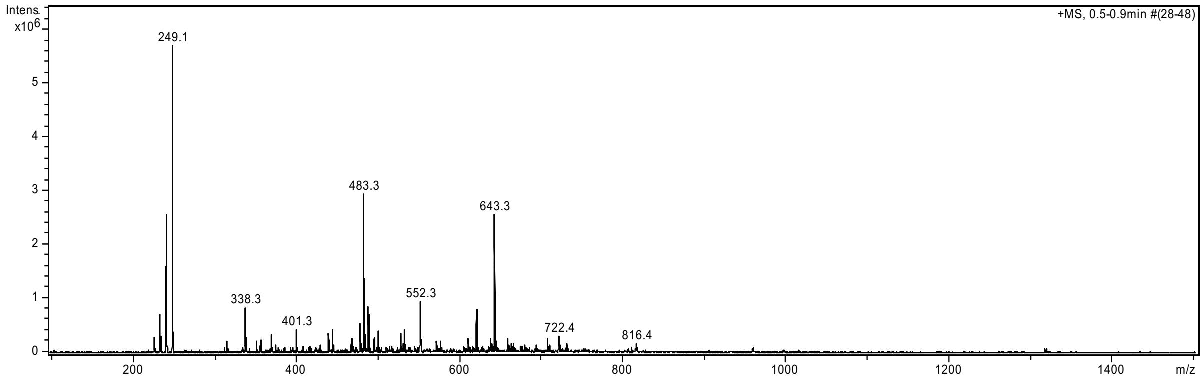

Supplementary Figure 17. ESI(+)-MS spectrum of $DSAPF_6$.

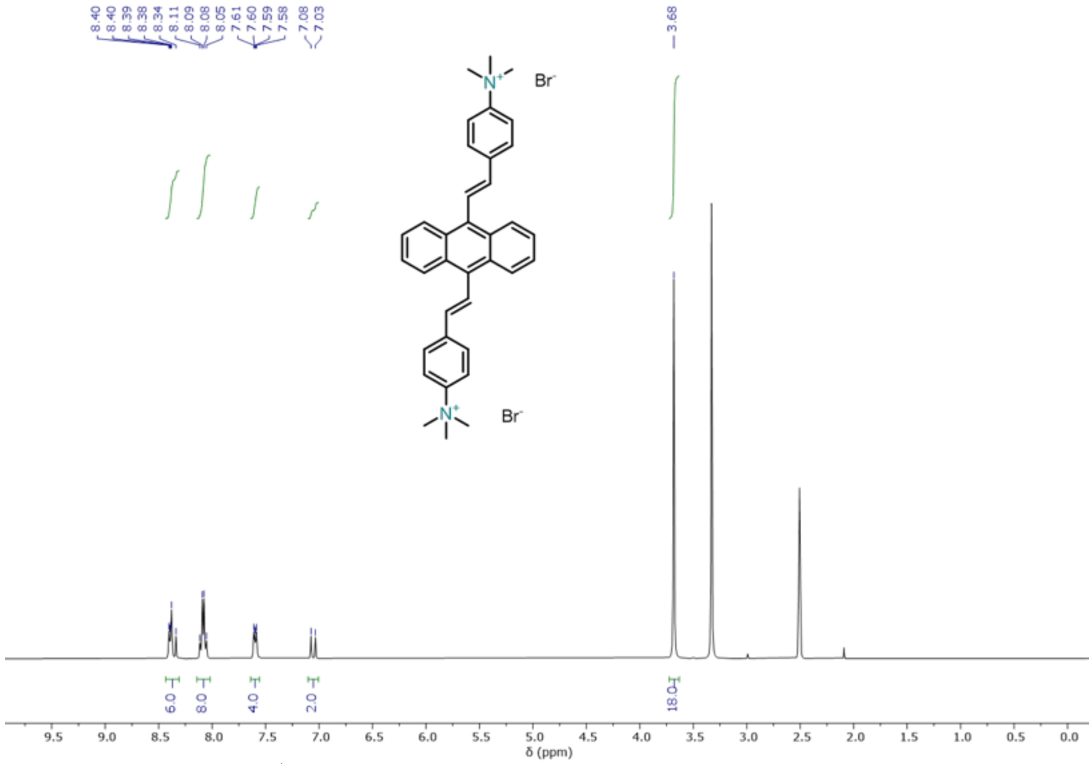

Supplementary Figure 18. ^{1}H NMR (400 MHz, DMSO-d_6) of DSABr.

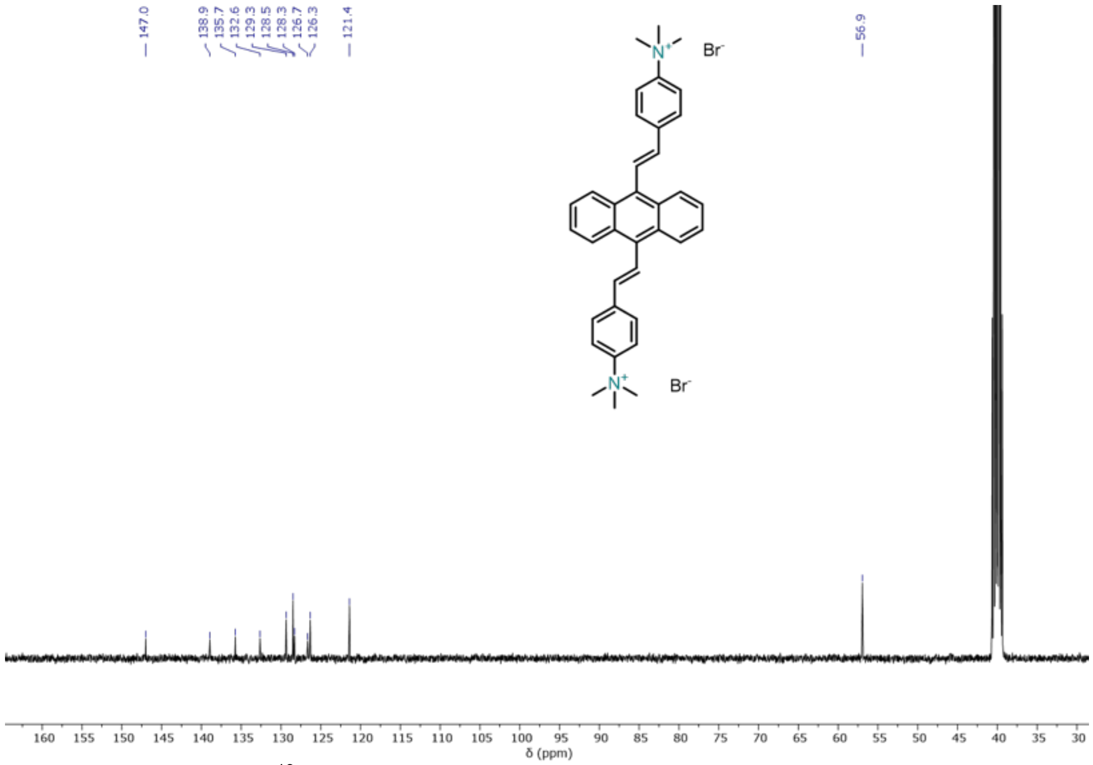

Supplementary Figure 19. ^{13}C NMR (101 MHz, DMSO-d_6) of DSABr.

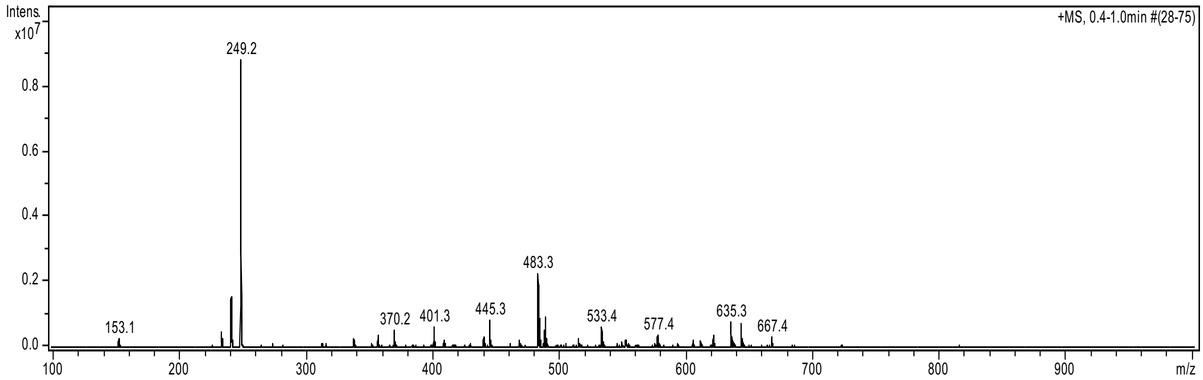

Supplementary Figure 20. ESI(+)-MS spectrum of DSABr.

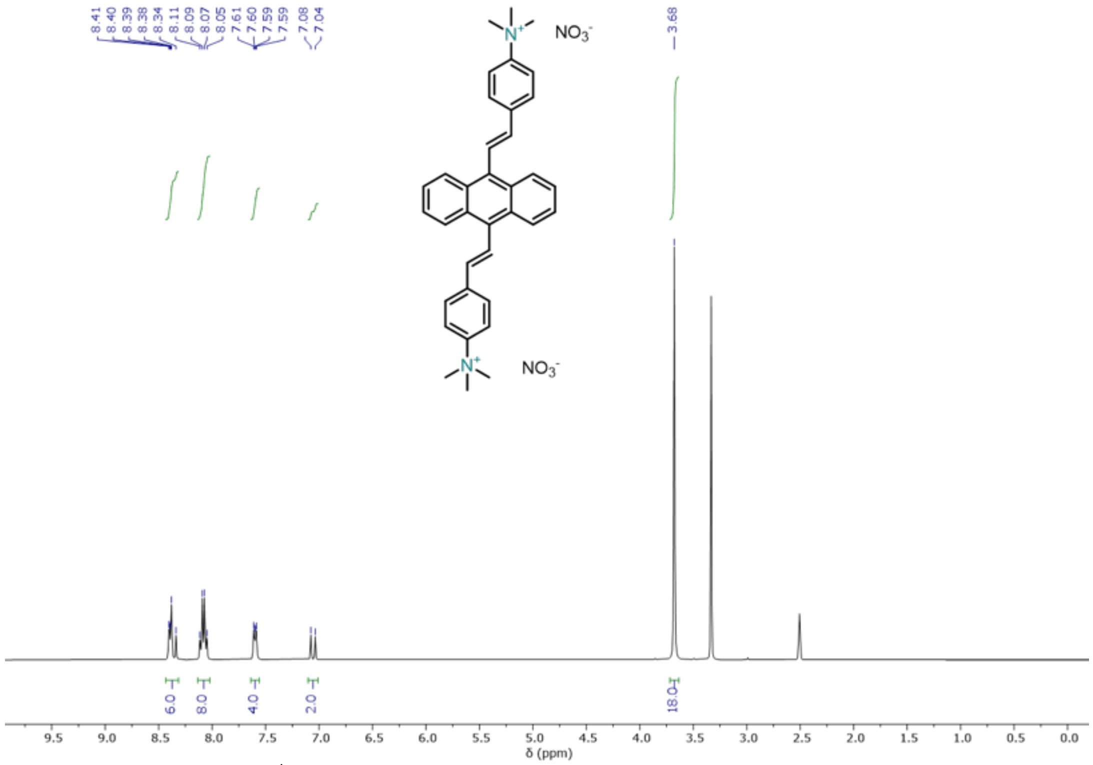

Supplementary Figure 21. ^{1}H NMR (400 MHz, DMSO-d_6) of $DSANO_3$.

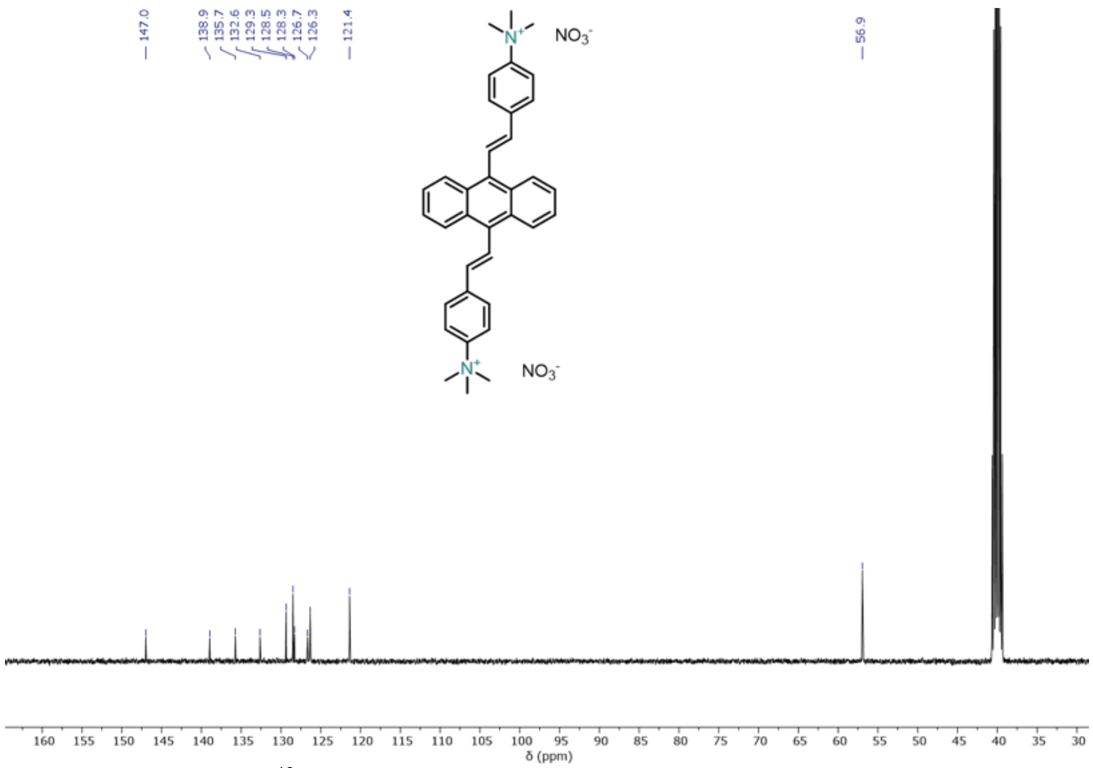

Supplementary Figure 22. ^{13}C NMR (101 MHz, DMSO-d_6) of $DSANO_3$.

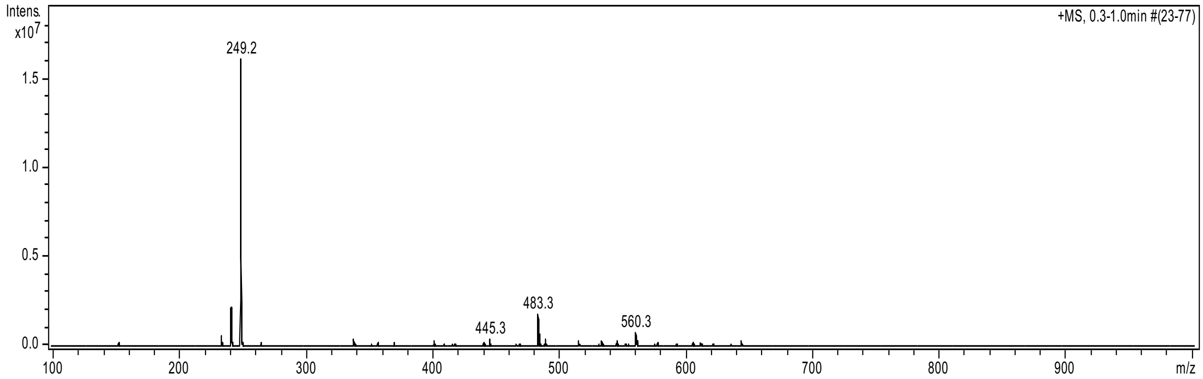

Supplementary Figure 23. ESI(+)-MS spectrum of $DSANO_3$.

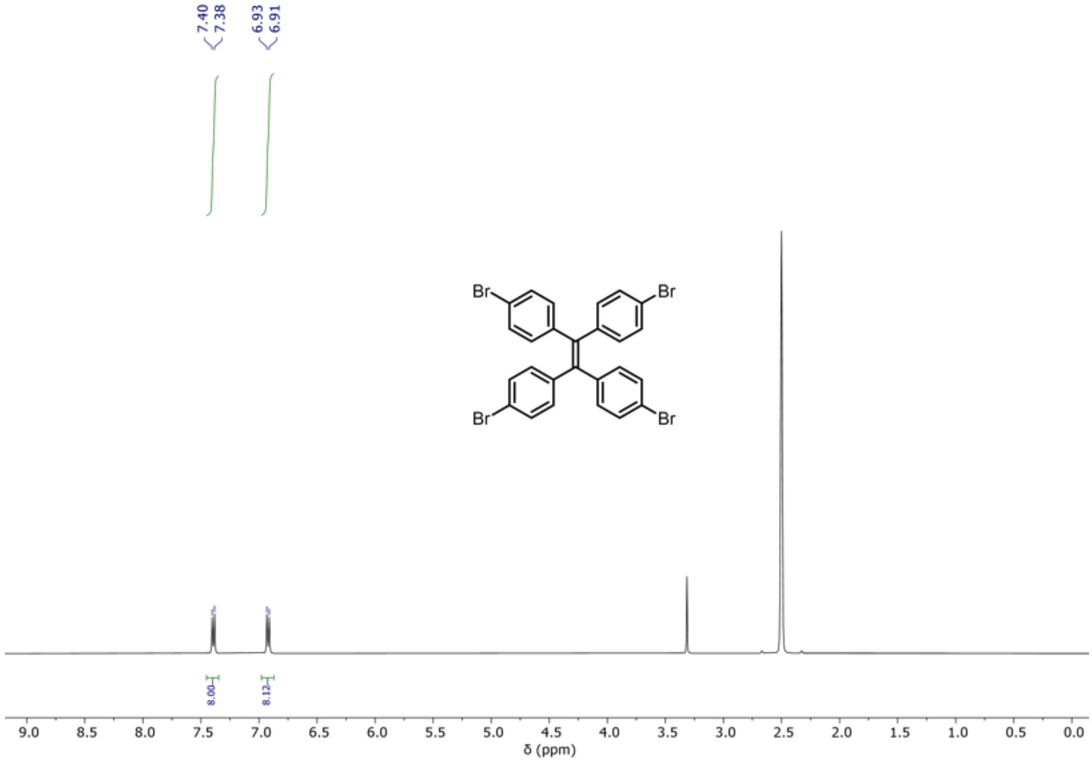

Supplementary Figure 24. ^1H NMR (400 MHz, DMSO-d_6) of 1,1,2,2-tetrakis(4-bromophenyl)ethylene (S4).

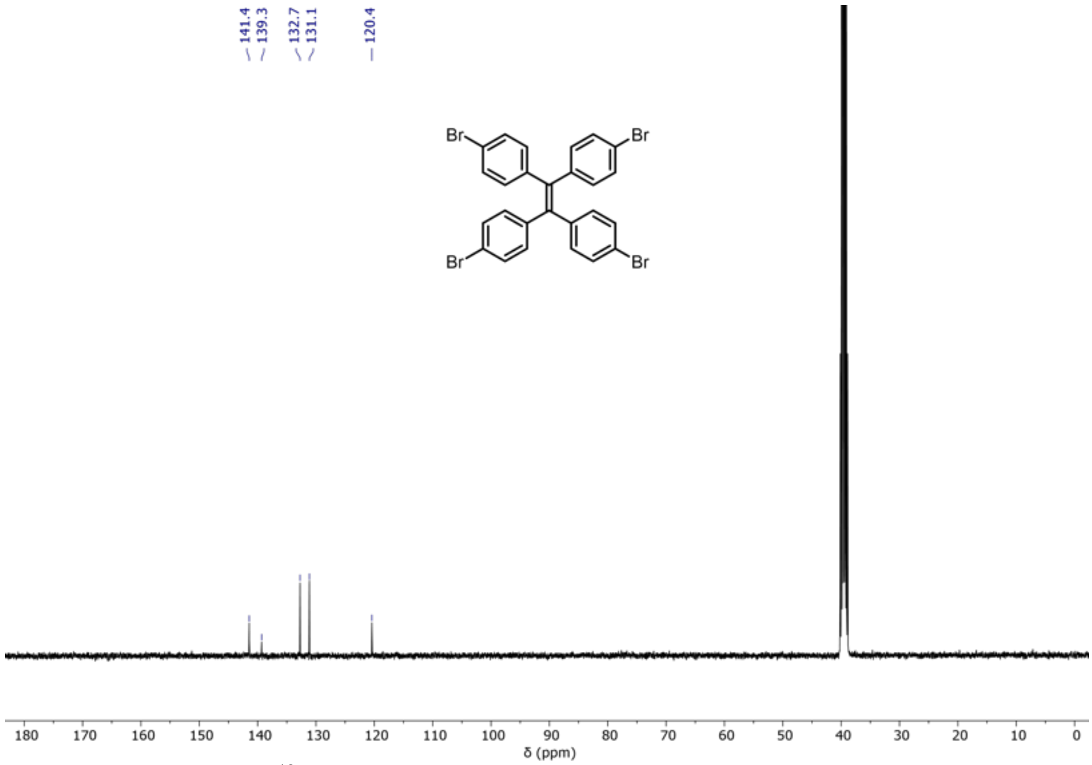

Supplementary Figure 25. ^{13}C NMR (101 MHz, DMSO-d_6) of 1,1,2,2-tetrakis(4-bromophenyl)ethylene (S4).

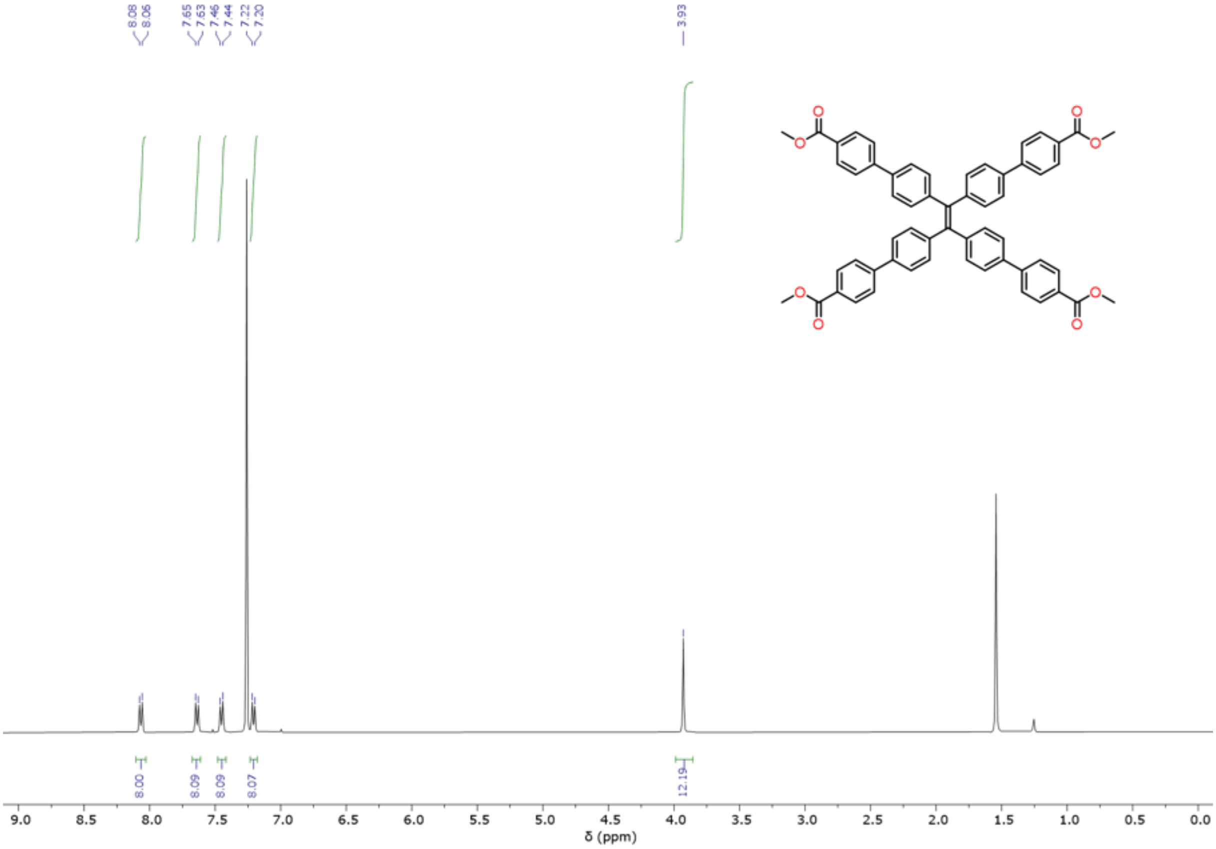

Supplementary Figure 26. ^{1}H NMR (400 MHz, Chloroform-*d*) of 4’,4’’’,4’’’’’,4’’’’’’’-(ethene-1,1,2,2-tetrayl)tetrakis([1,1′-biphenyl]-4-carboxylate) (S5).

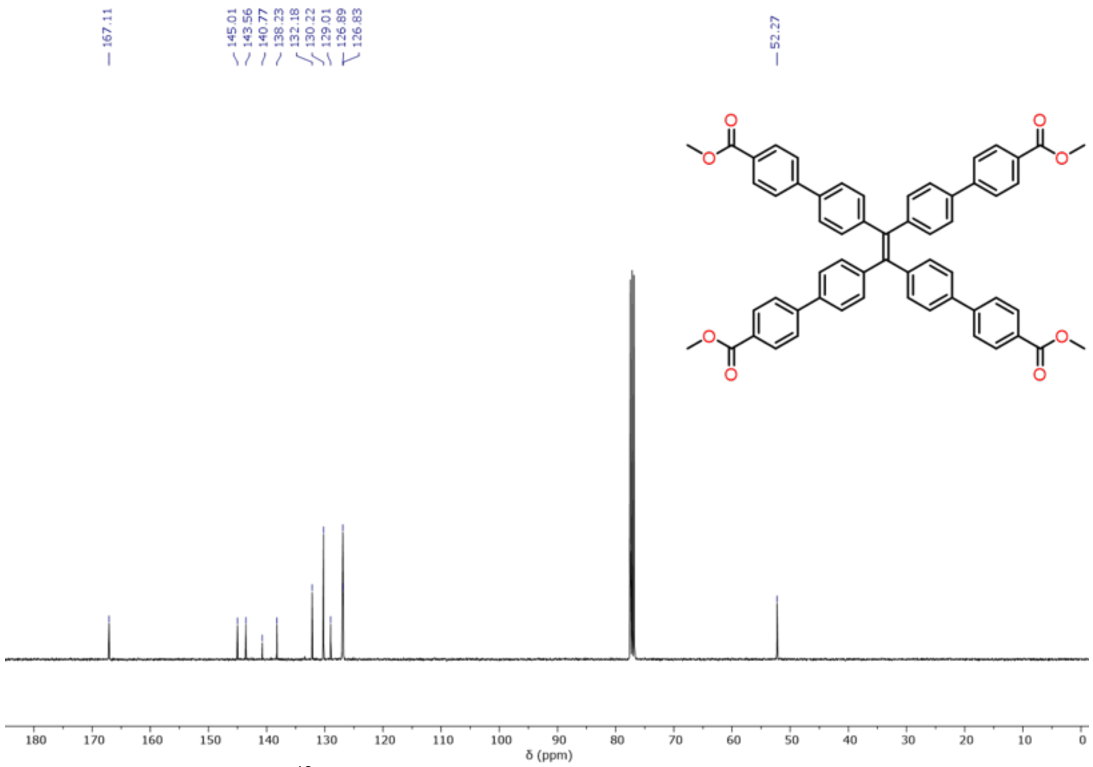

Supplementary Figure 27. ^{13}C NMR (101 MHz, Chloroform-*d*) of 4’,4’’’,4’’’’’,4’’’’’’’-(ethene-1,1,2,2-tetrayl)tetrakis([1,1′-biphenyl]-4-carboxylate) (S5).

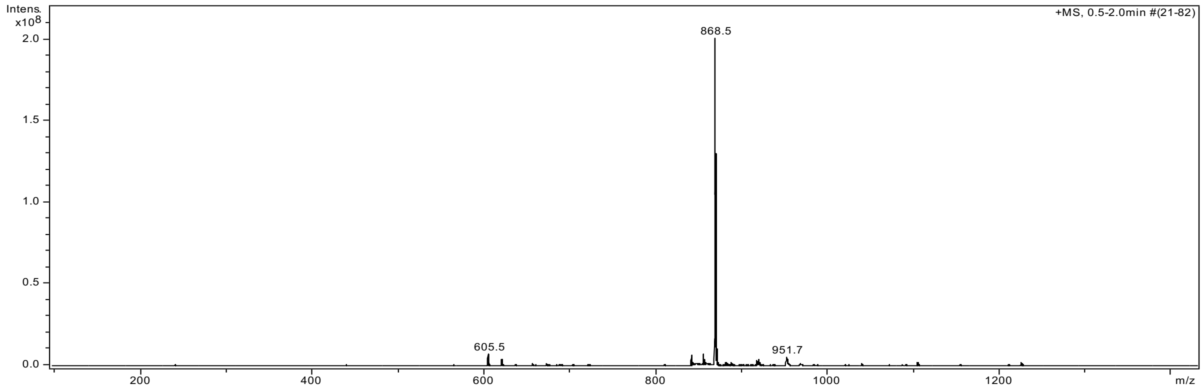

Supplementary Figure 28. APCI(+)-MS of of 4’,4’’’,4’’’’’,4’’’’’’’-(ethene-1,1,2,2-tetrayl)tetrakis([1,1′-biphenyl]-4-carboxylate) (S5).

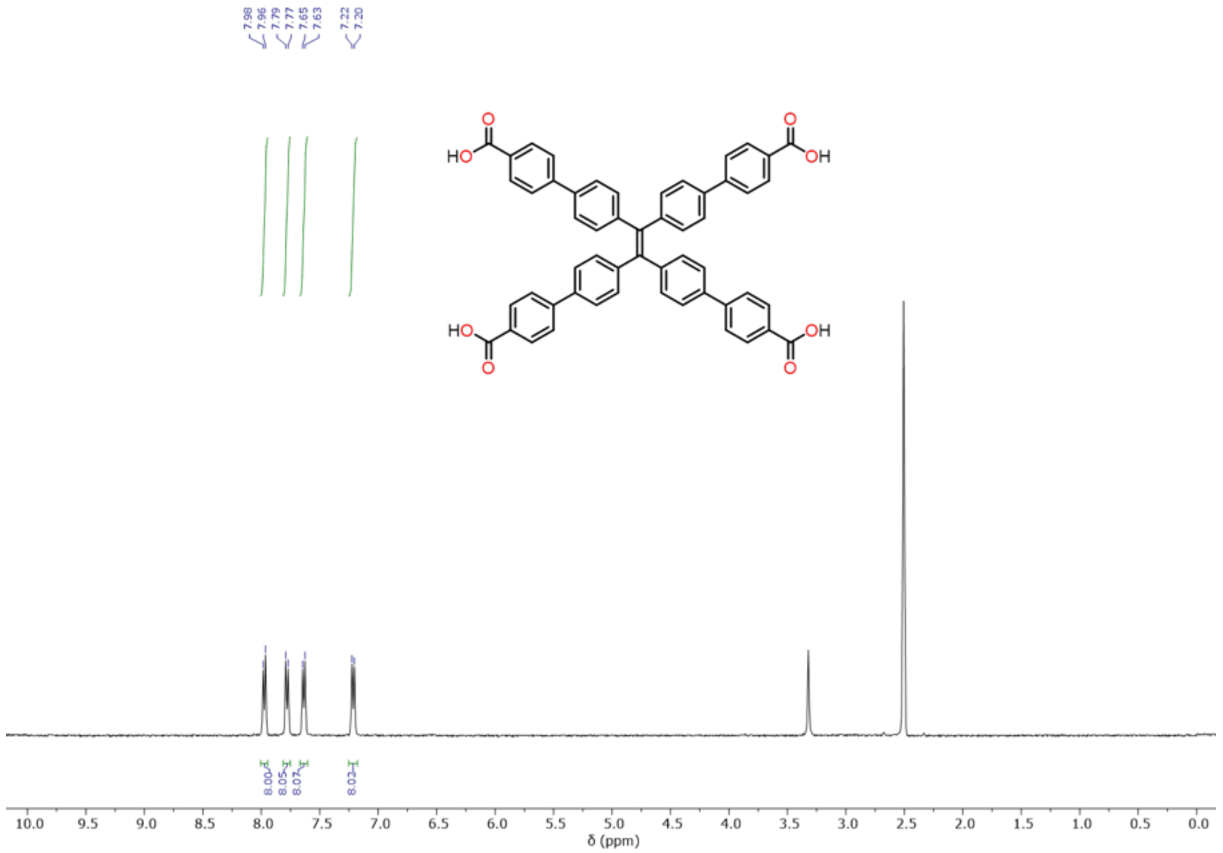

Supplementary Figure 29. ^{1}H NMR (400 MHz, DMSO-d_6) of TBATPE.

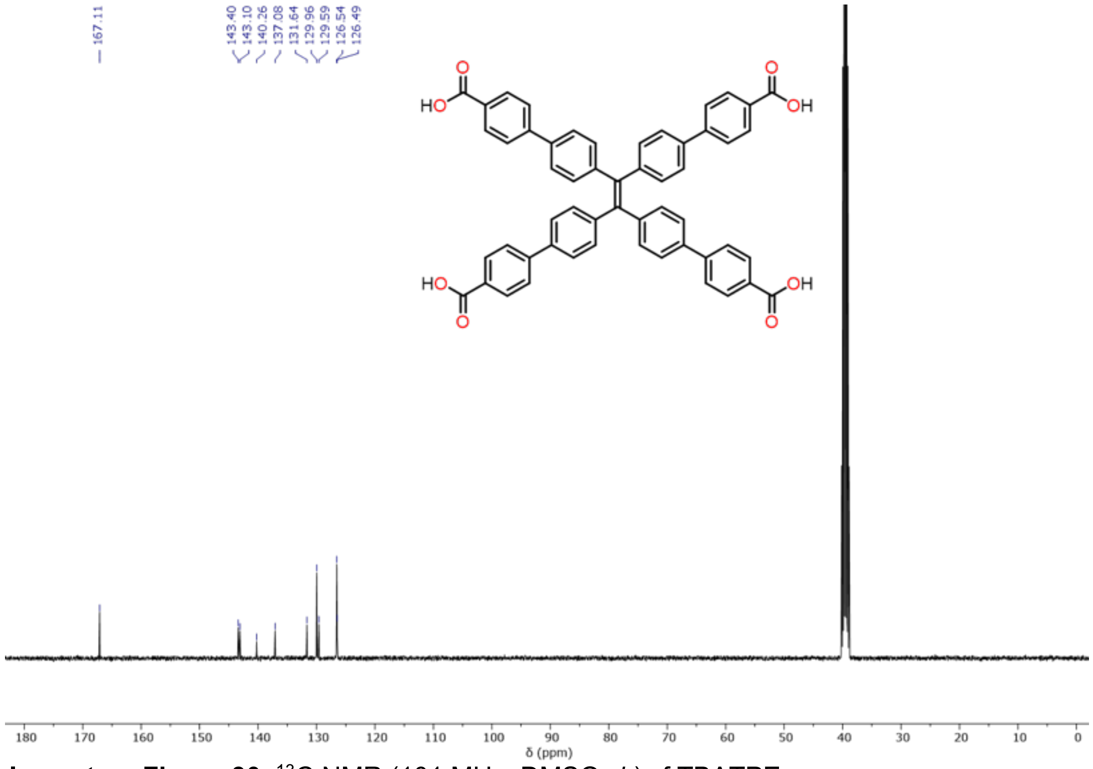

Supplementary Figure 30. ^{13}C NMR (101 MHz, DMSO-d_6) of TBATPE.

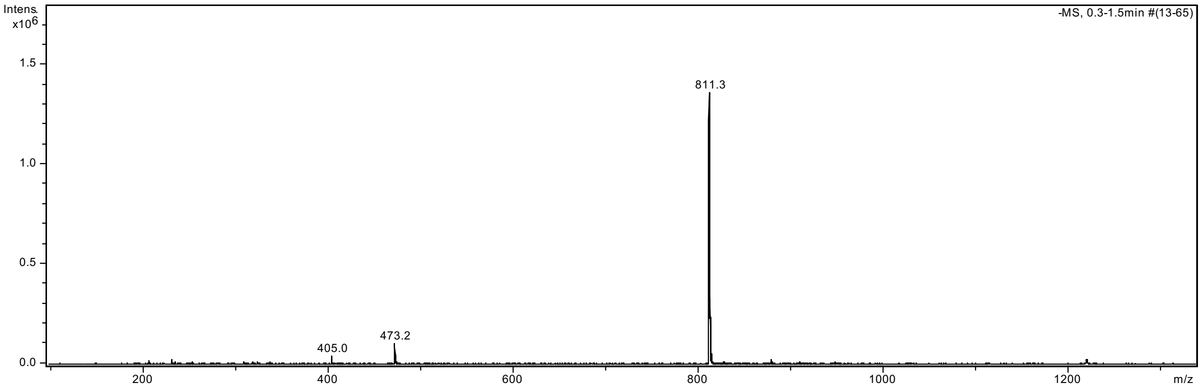

Supplementary Figure 31. ESI(–)-MS spectrum of TBATPE.

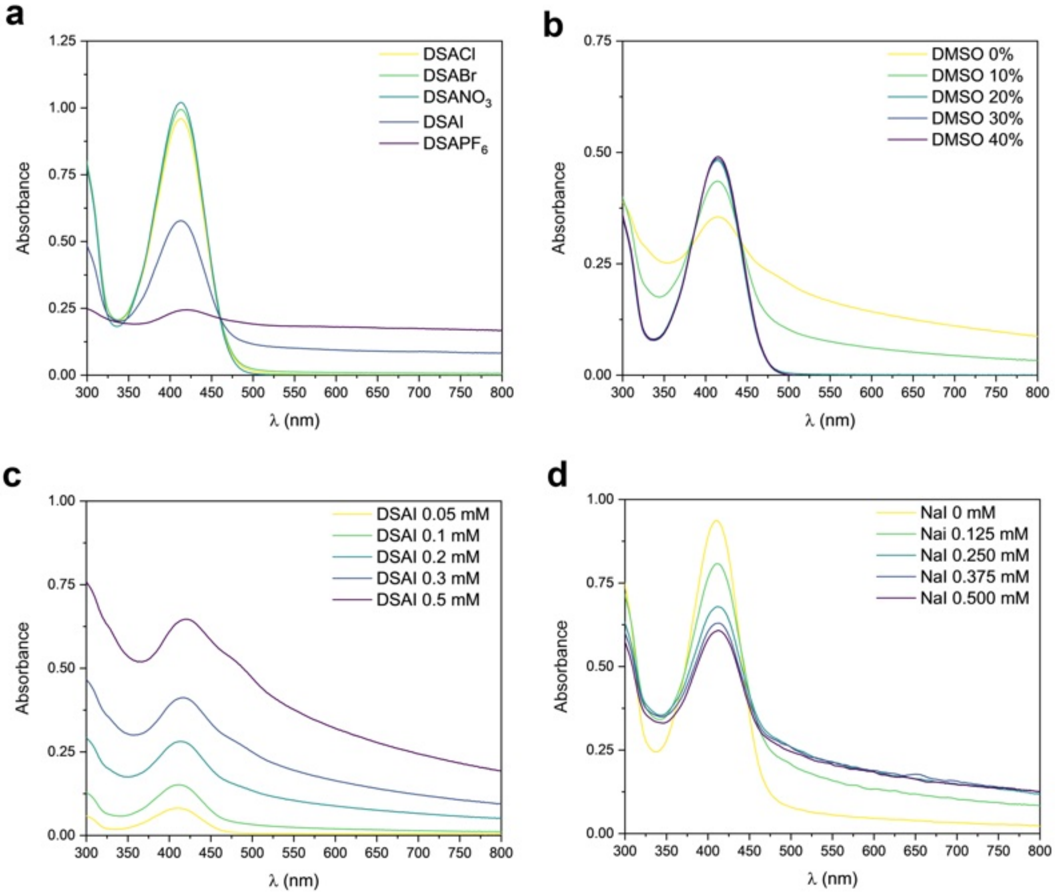

Supplementary Figure 32. a) UV-Vis spectra of DSACl, DSABr, $DSANO_3$, DSAI, $DSAPF_6$ 1 mM in mQ-water with 5% DMSO using 0.5 mm quartz cuvette, b) UV-Vis spectra of DSAI 0.25 mM in mQ-water with different % of DMSO (0, 10, 20, 30, 40%) using 1 mm quartz cuvette, c) UV-Vis spectra of DSAI at different concentrations (0.05, 0.1, 0.2, 0.3, 0.5 mM) in mQ-water using 1 mm quartz cuvette, d) UV-Vis spectra of DSAI (0.05 mM) with different concentrations of NaI (0, 0.125, 0.25, 0.375, 0.5) in mQ-water using 10 mm quartz cuvette.

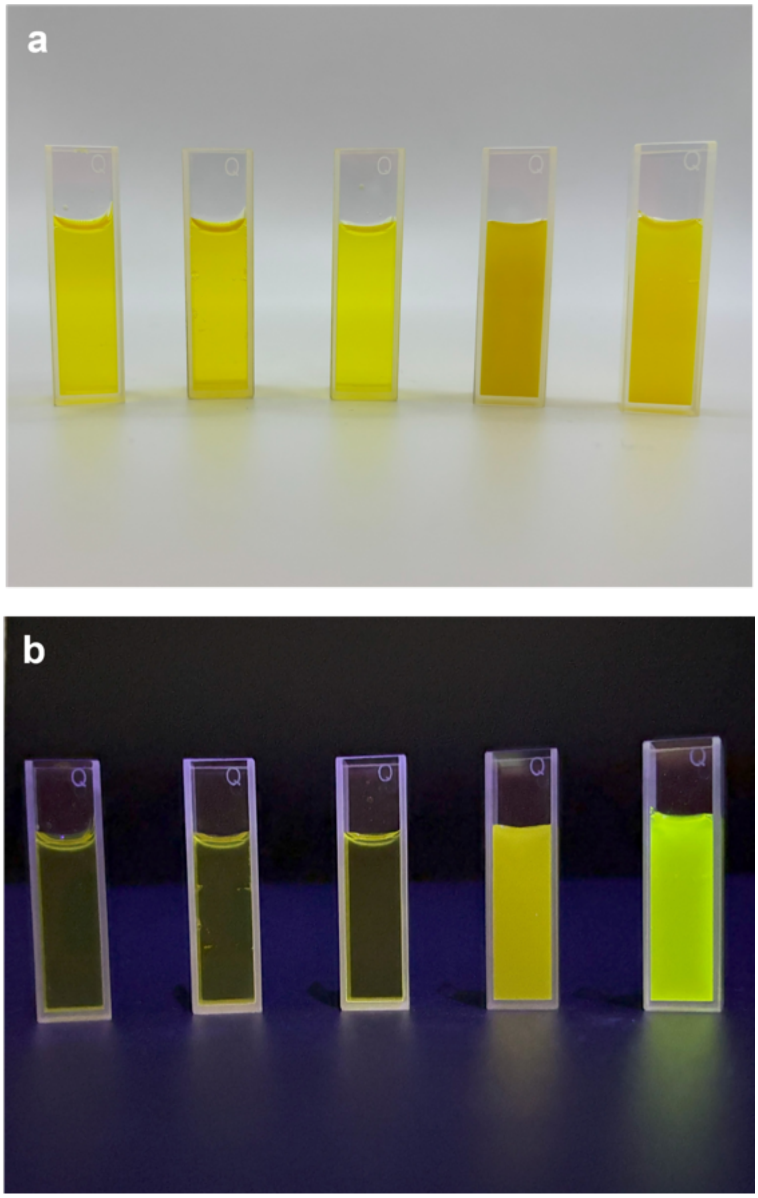

Supplementary Figure 33. Photographs of DSACl, DSABr, $DSANO_3$, DSAI, $DSAPF_6$ (1 mM, from left to right) in mQ-water with 5% DMSO: a) under daylight, and b) under 365 nm irradiation.

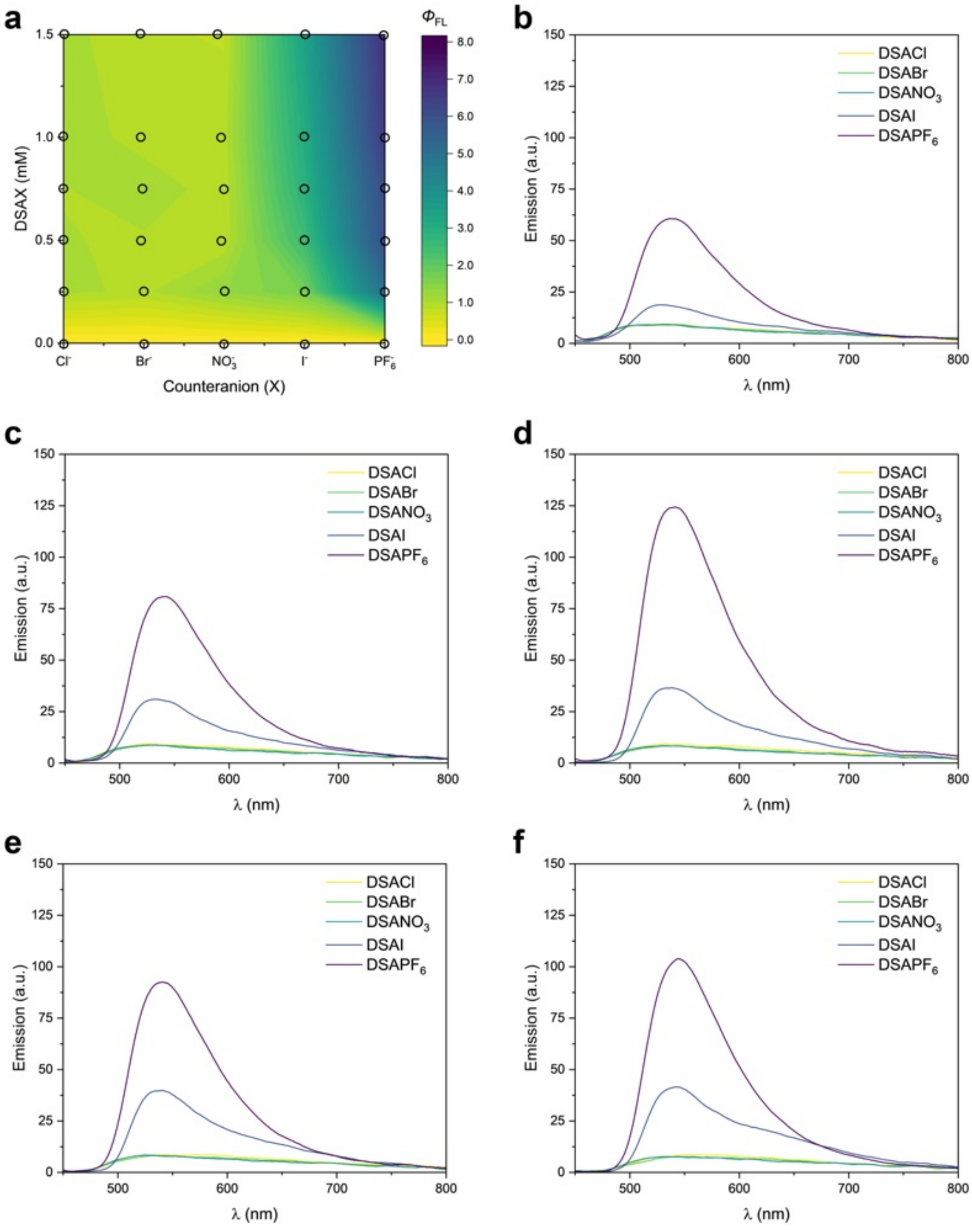

Supplementary Figure 34. a) Assembly landscape at different concentrations of DSA^{2+} amphiphile (0–1.5 mM) with selected counteranions (Cl^{-}, Br^{-}, NO_3^{-}, I^{-}, PF_6^{-}) and related emission spectra of DSAX b) 0.25, c) 0.50, d) 0.75, e) 1.0, and f) 1.5 mM in mQ-water with 5% DMSO. The black circles (in panel a) represent the Φ_{FL} measurements performed.

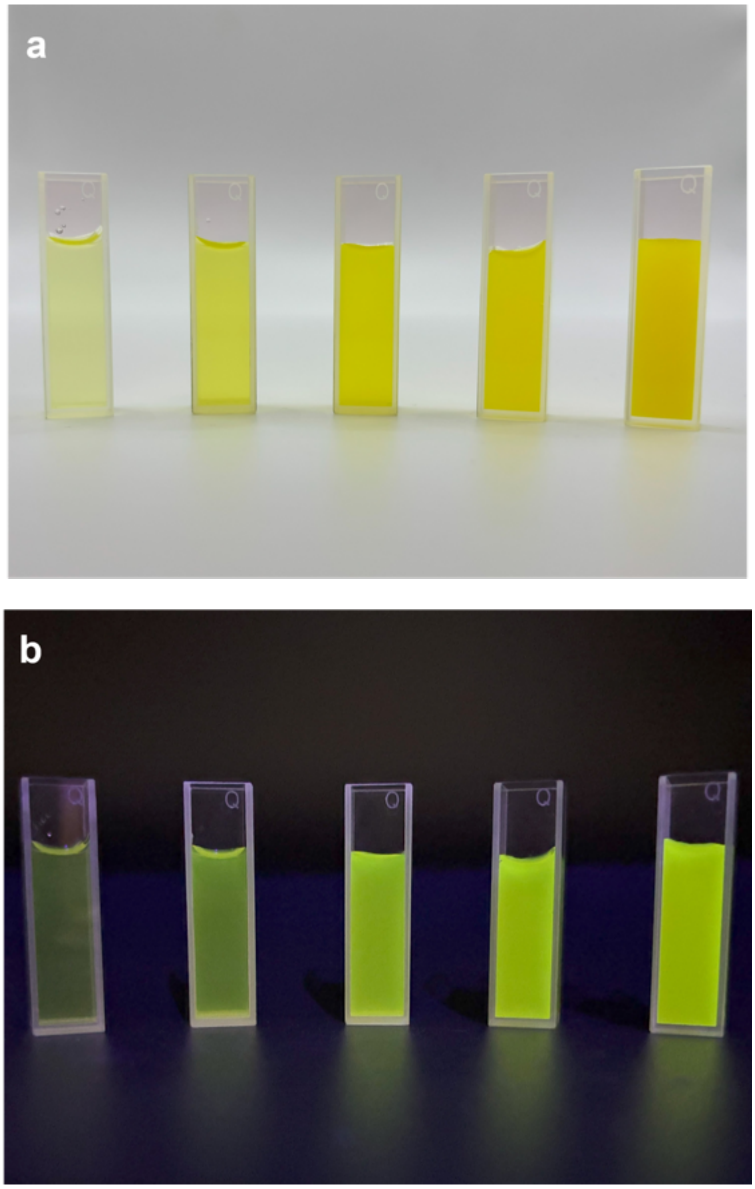

Supplementary Figure 35. Photographs of DSAI at different concentrations (0.05, 0.1, 0.2, 0.3, 0.5 mM, from left to right) in mQ-water: a) under daylight, and b) under 365 nm irradiation.

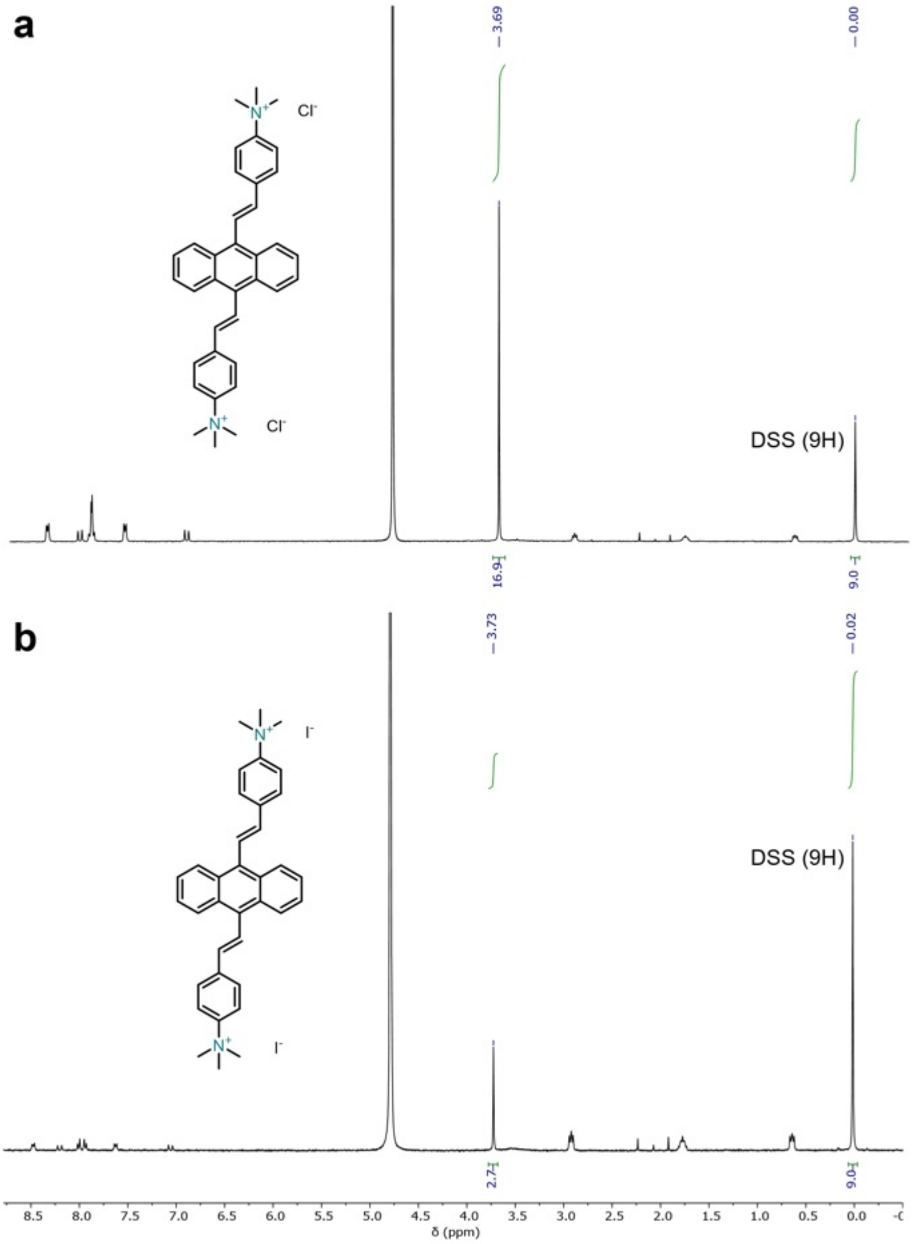

Supplementary Figure 36. a) ^{1}H NMR (400 MHz, D_2O) spectra of 0.5 mM DSACl and b) DSAI containing both sodium 3-(trimethylsilyl)propane-1-sulfonate (DSS) 0.5 mM as internal standard.

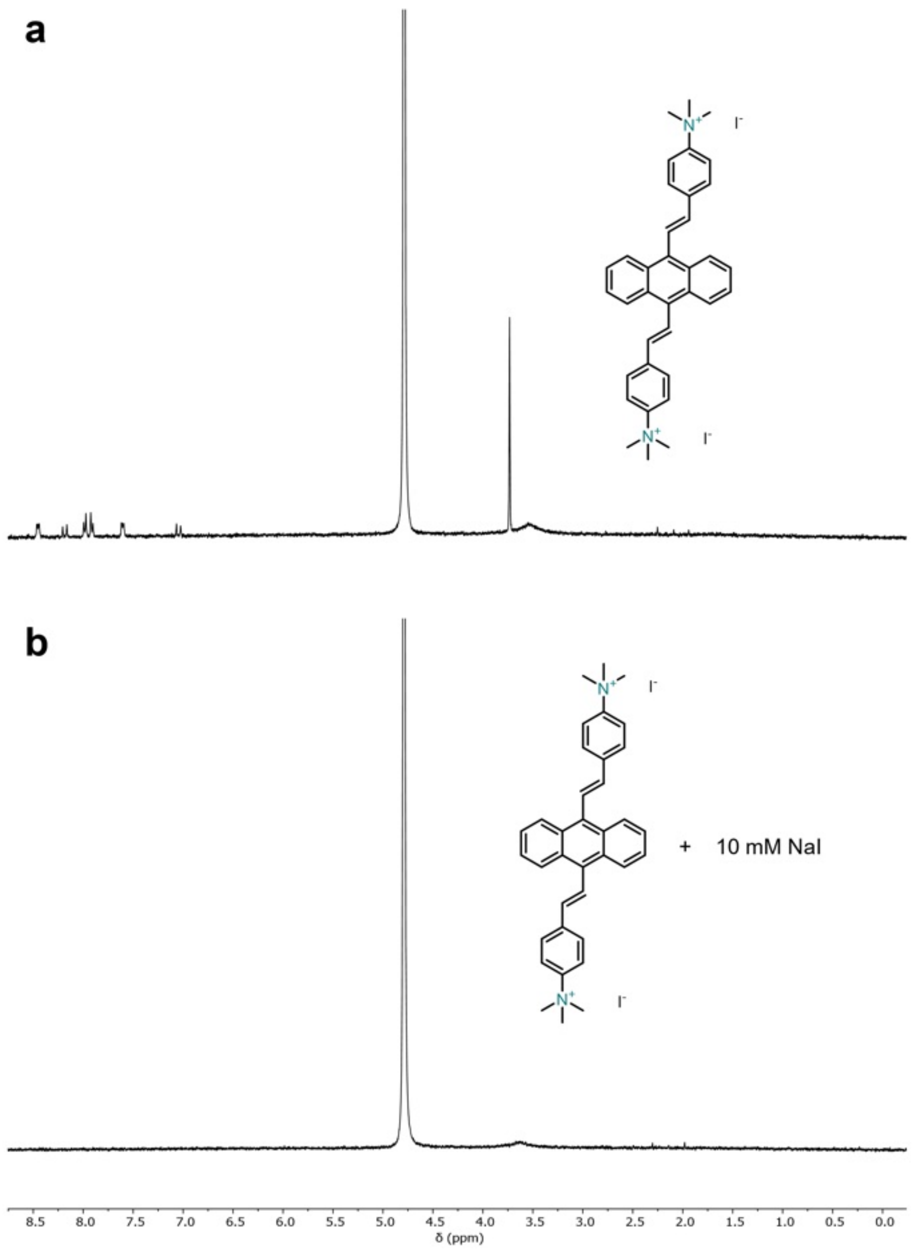

Supplementary Figure 37. ^{1}H NMR (400 MHz, D_2O) spectra of 0.5 mM DSAI a) in absence and b) in presence of 10 mM NaI.

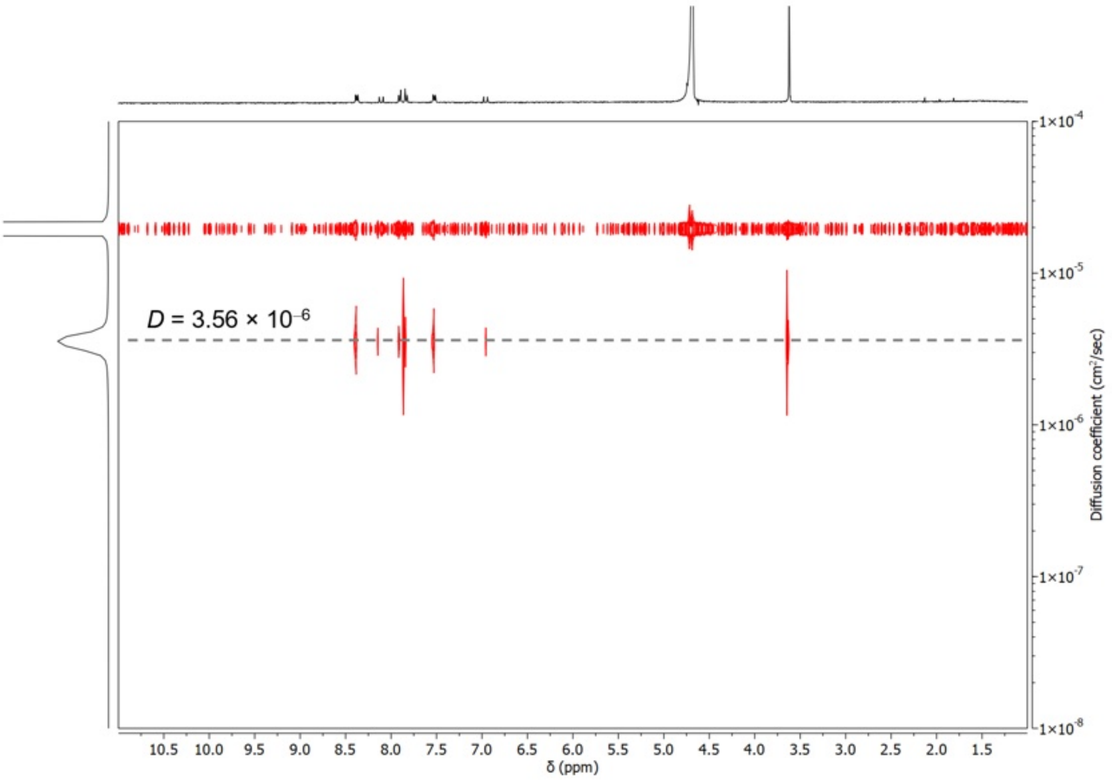

Supplementary Figure 38. ¹H diffusion-ordered spectroscopy (DOSY) of DSAI 0.5 mM in D_2O, with the diffusion coefficient indicating that the residual signals of DSAI are of its monomer species.

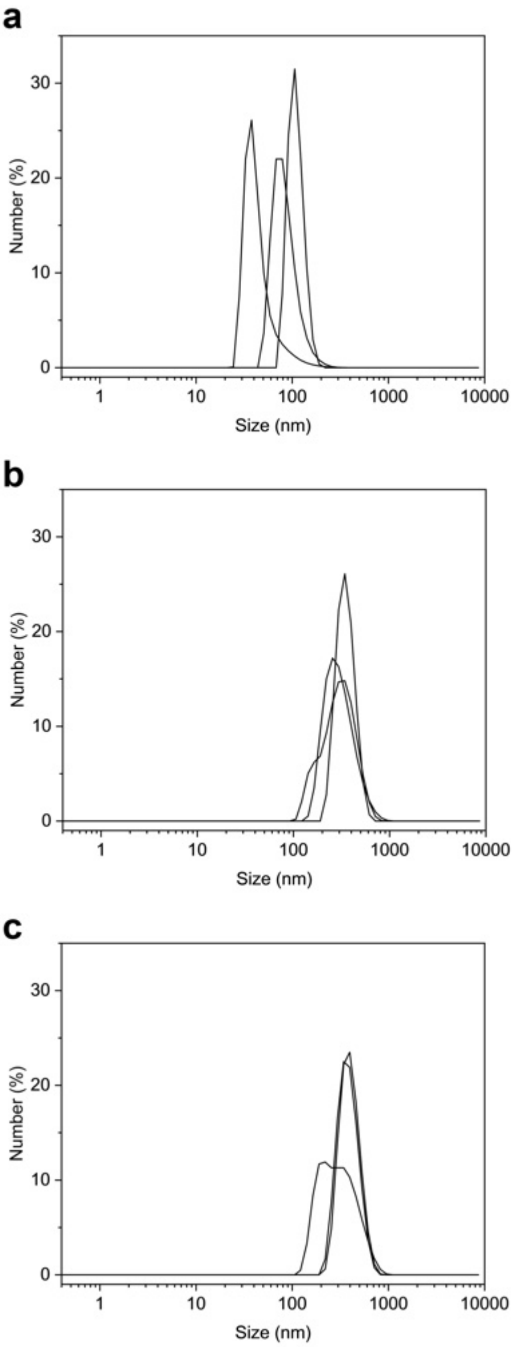

Supplementary Figure 39. DLS analysis (3 measurements per sample) of 0.05 mM DSAI in the presence of a) 0 mM, b) 0.25 mM, and c) 0.50 mM NaI in mQ-water. The presence of NaI leads to a higher hydrodynamic diameter of aggregates dispersed in water (from around 100 nm to 300 nm). The average size from DLS remains constant for 0.25 and 0.50 mM NaI samples.

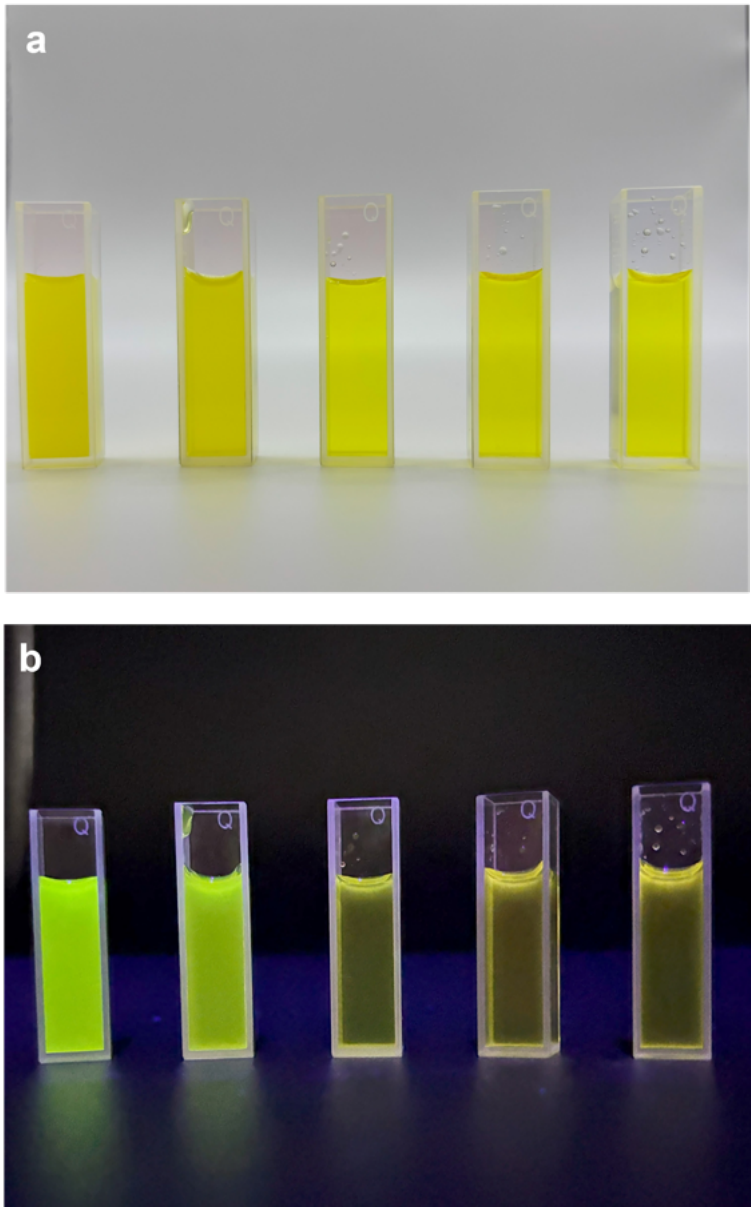

Supplementary Figure 40. Photographs of DSAI 0.25 mM in mQ-water with different % of DMSO (0, 10, 20, 30, 40%, from left to right): a) under daylight, and b) under 365 nm irradiation.

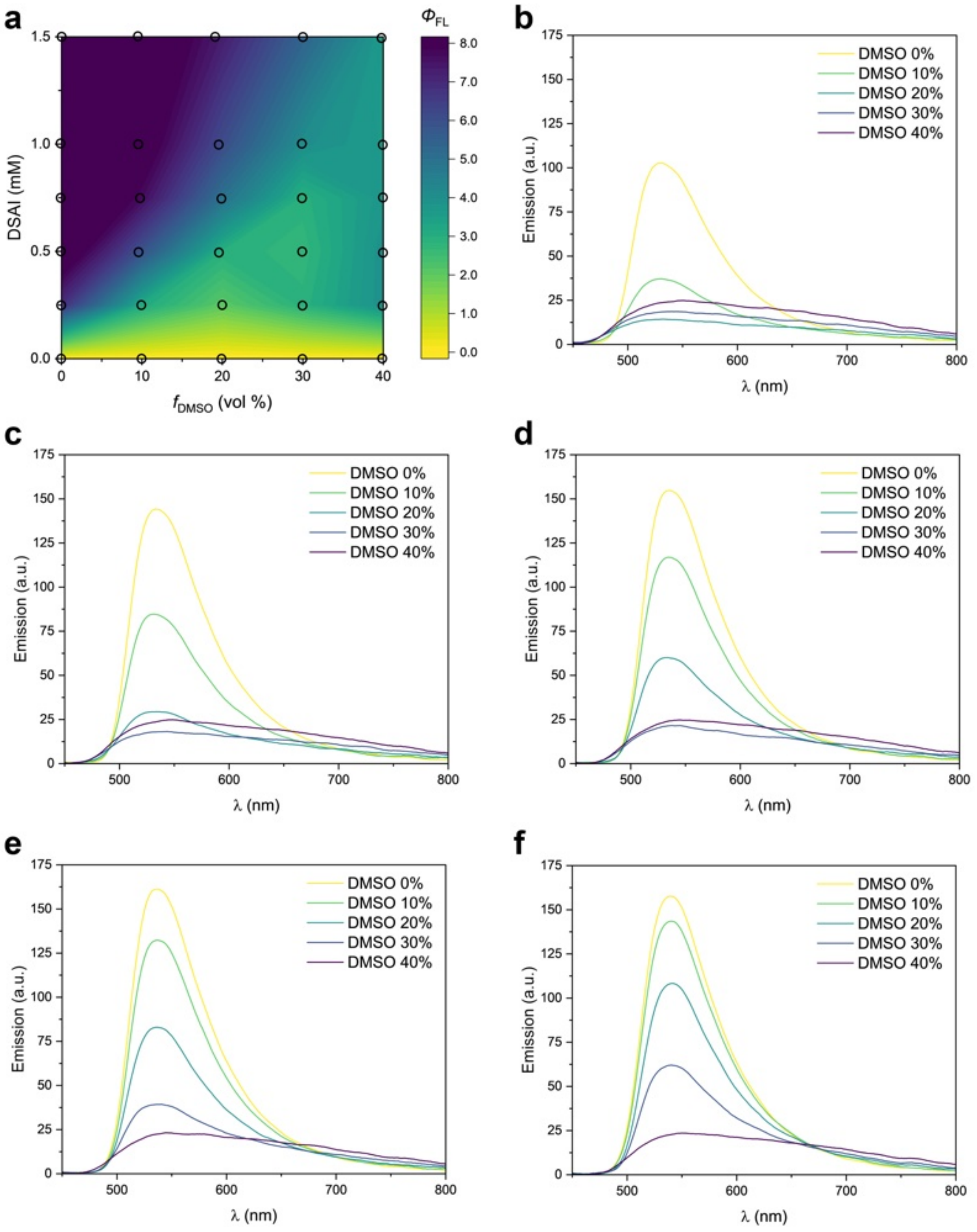

Supplementary Figure 41. a) Assembly landscape at different concentrations of DSAI amphiphile (0–1.5 mM) under various ratios between mQ-water and DMSO (0–40% of DMSO) and related emission spectra of DSAI b) 0.25, c) 0.50, d) 0.75, e) 1.0, and f) 1.5 mM. The black circles (in panel a) represent the Φ_{FL} measurements performed.

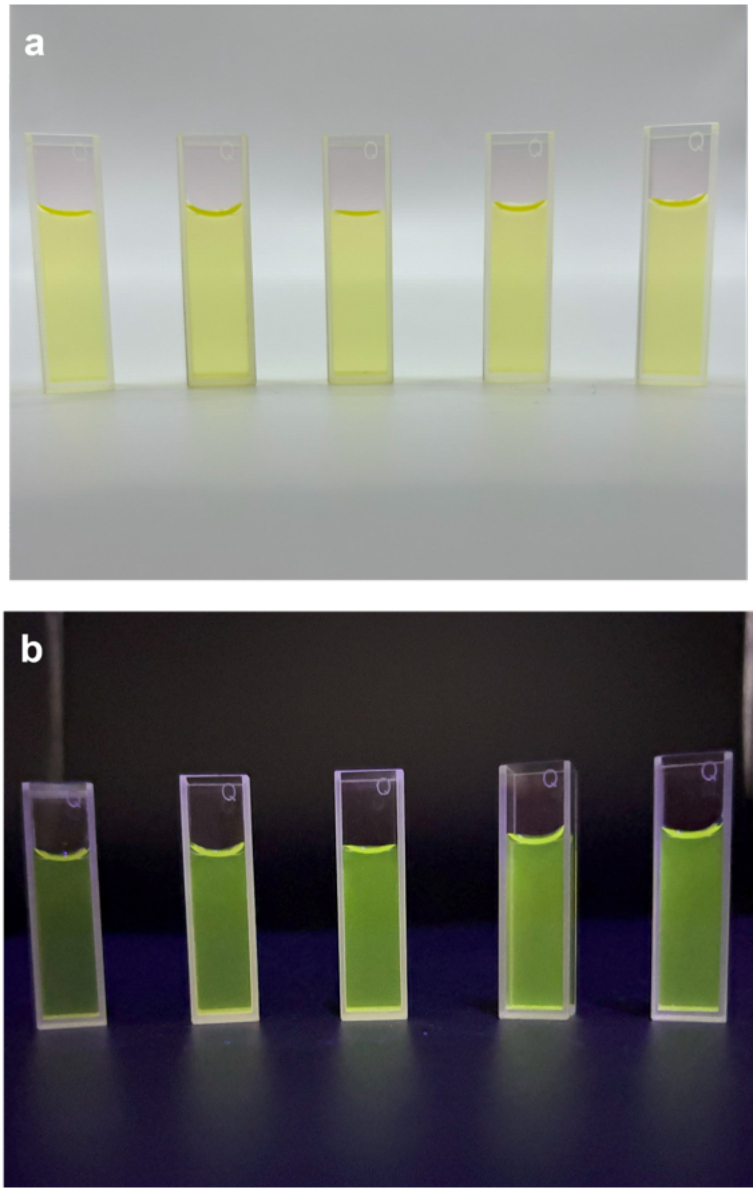

Supplementary Figure 42. Photographs of DSAI (0.05 mM) with different concentrations of NaI (0, 0.125, 0.25, 0.375, 0.5 mM, from left to right) in mQ-water: a) under daylight, and b) under 365 nm irradiation.

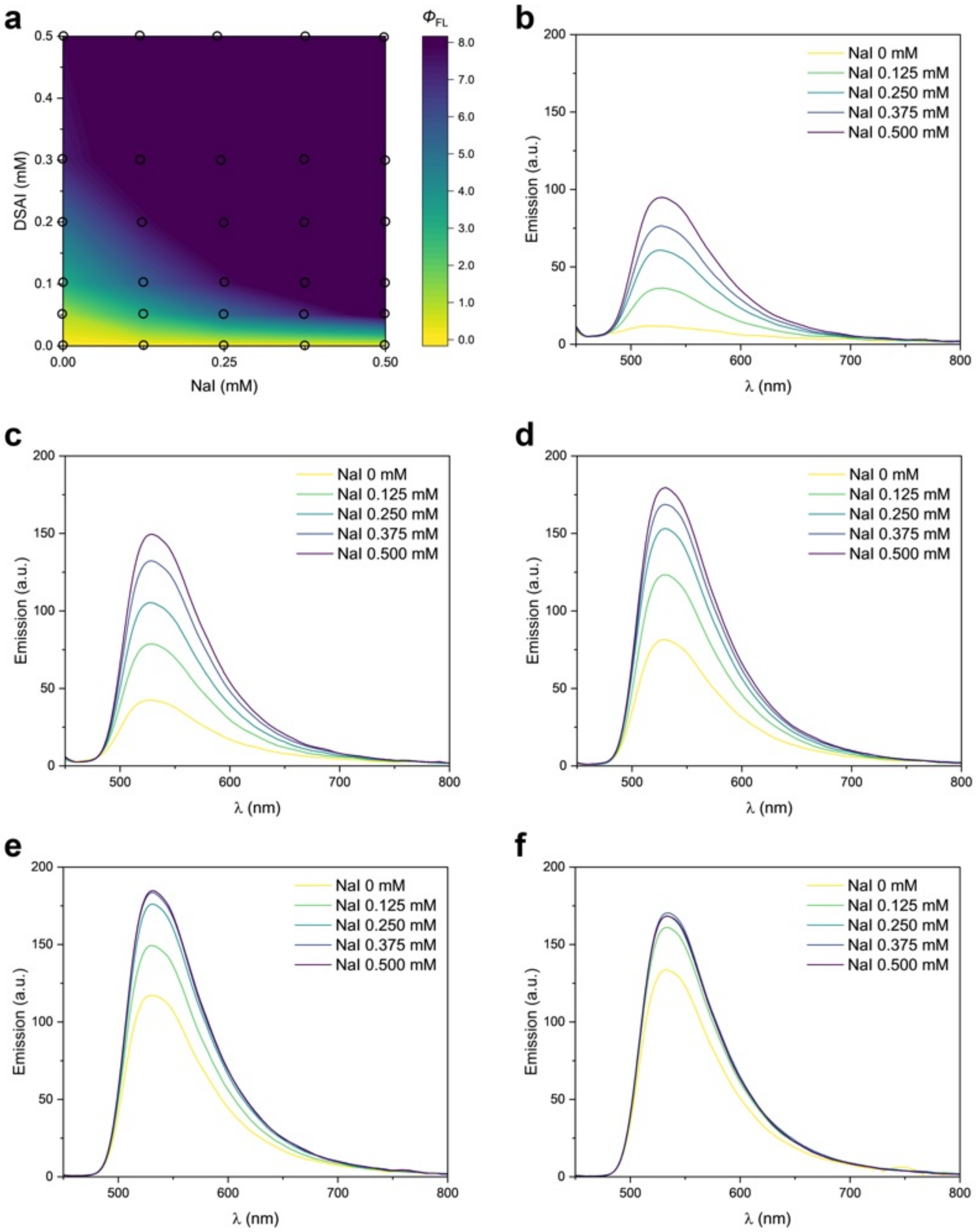

Supplementary Figure 43. a) Assembly landscape at different concentration of DSAI amphiphile (0–0.5 mM) with different amount of NaI (0–0.5 mM) and related emission spectra of DSAI b) 0.05, c) 0.1, d) 0.2, e) 0.3, and f) 0.5 mM with different NaI concentrations in mQ-water. The black circles (in panel a) represent the Φ_{FL} measurements performed. At low concentrations of DSAI, higher Φ_{FL} are observed when the amount of NaI is increased. However, when the concentration of DSAI is above 0.3 mM, increasing the amount of NaI no longer has much effect on Φ_{FL} because the samples are already aggregated without any salt.

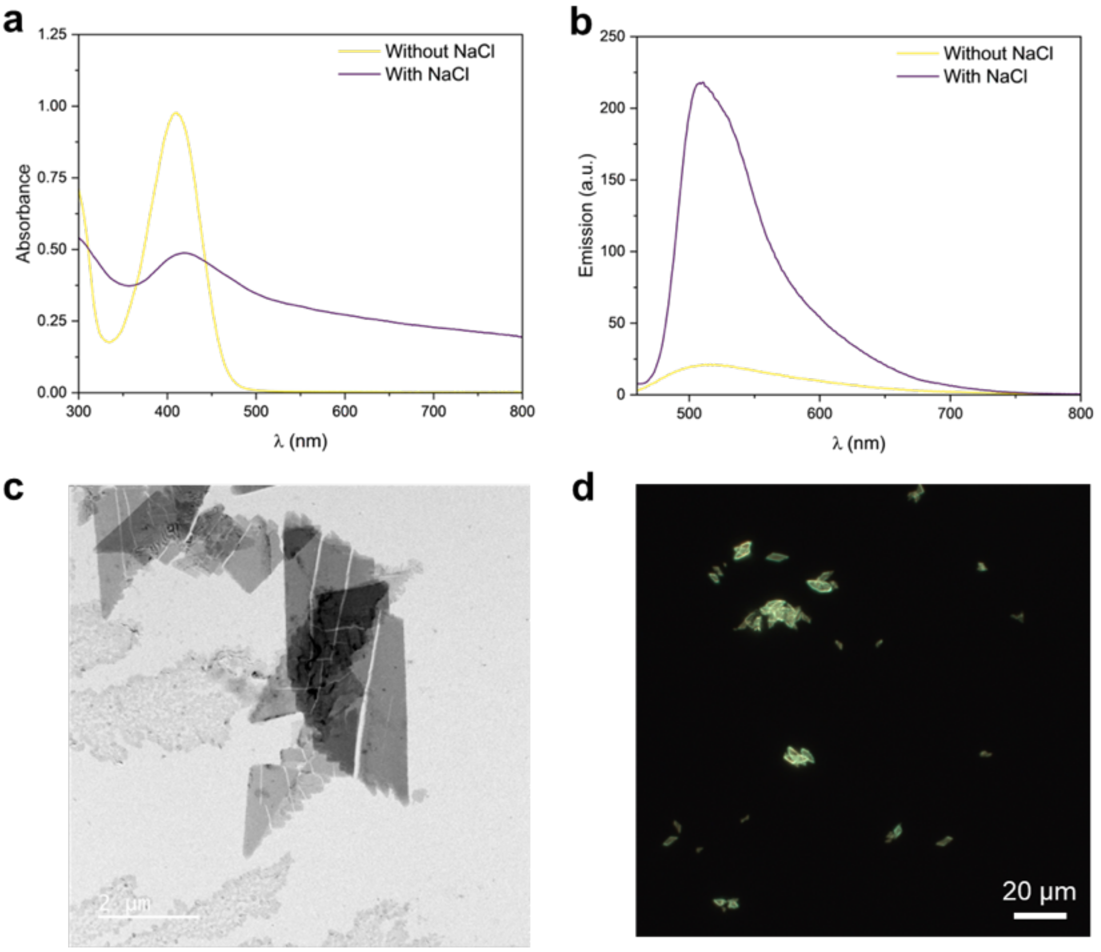

Supplementary Figure 44. Study of DSACl (0.05 mM) aggregation in presence of 1 M NaCl by monitoring a) absorption spectra, b) enhancement of fluorescence emission; and c) morphological analysis of DSACl 0.05 mM in presence of NaCl 1 M by TEM and d) widefield fluorescence microscopy.

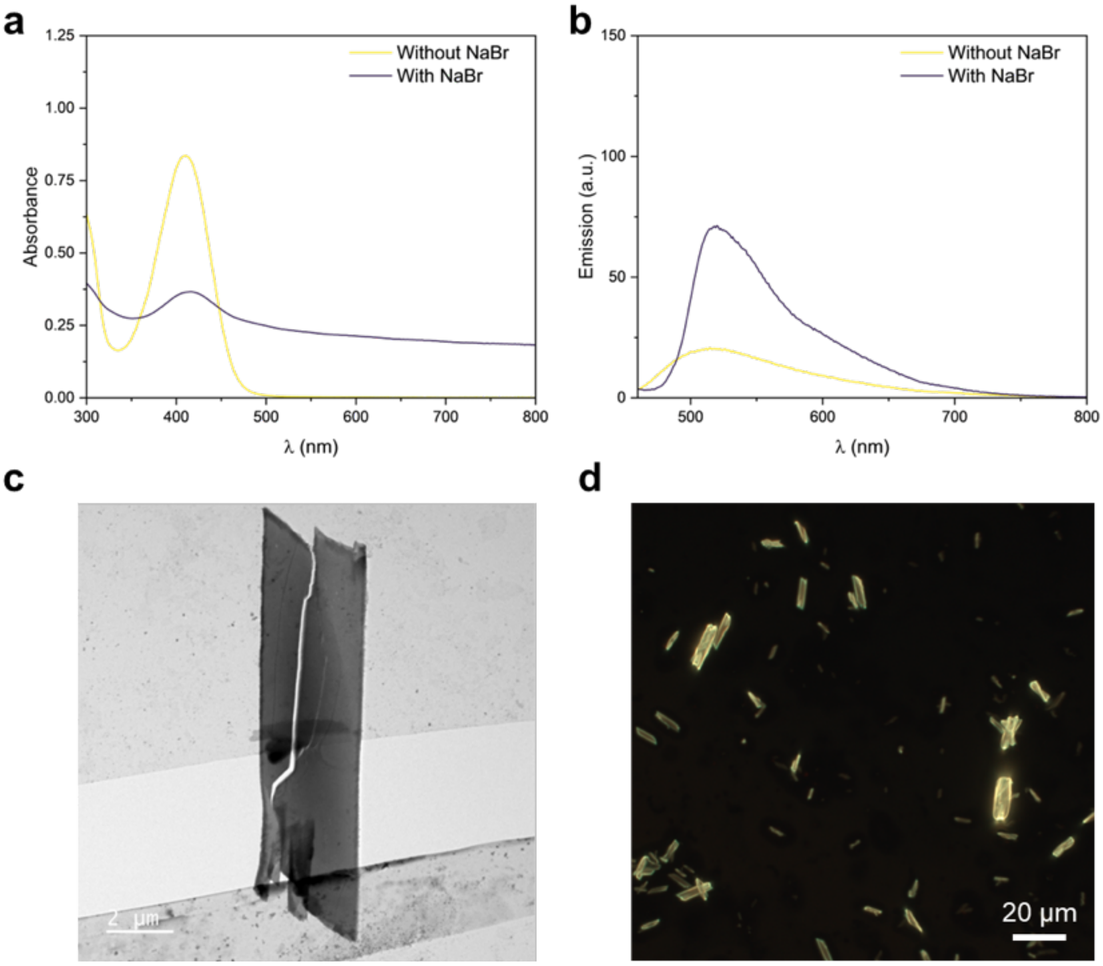

Supplementary Figure 45. Study of DSABr (0.05 mM) aggregation in the absence and presence of 0.03 M NaBr by monitoring a) absorption spectra, b) enhancement of fluorescence emission; and c) morphological analysis of DSABr 0.05 mM in presence of NaBr 0.03 M by TEM and d) widefield fluorescence microscopy.

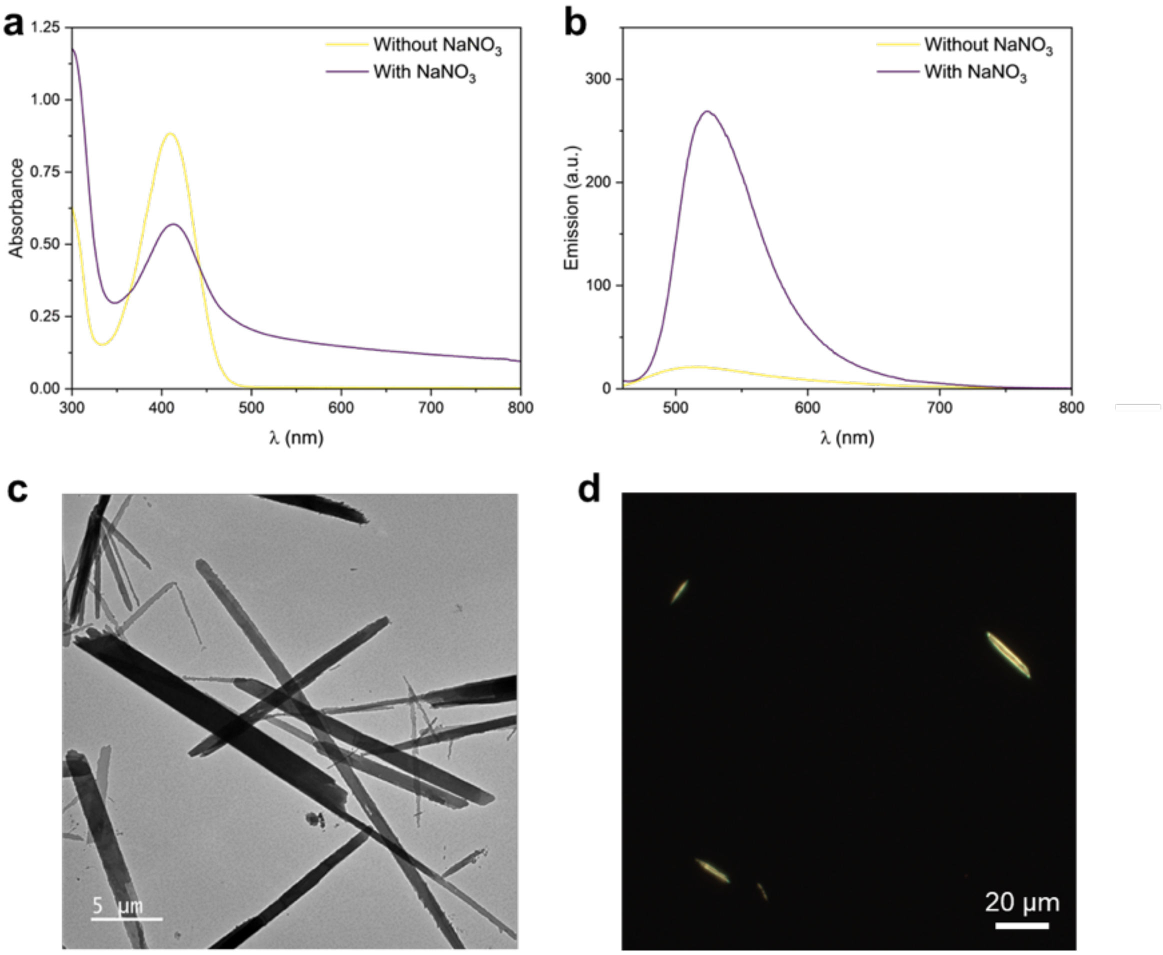

Supplementary Figure 46. Study of $DSANO_3$ (0.05 mM) aggregation in the absence and presence of 0.1 M $NaNO_3$ by monitoring a) absorption spectra, b) enhancement of fluorescence emission; and c) morphological analysis of $DSANO_3$ 0.05 mM in presence of $NaNO_3$ 0.1 M by TEM and d) widefield fluorescence microscopy.

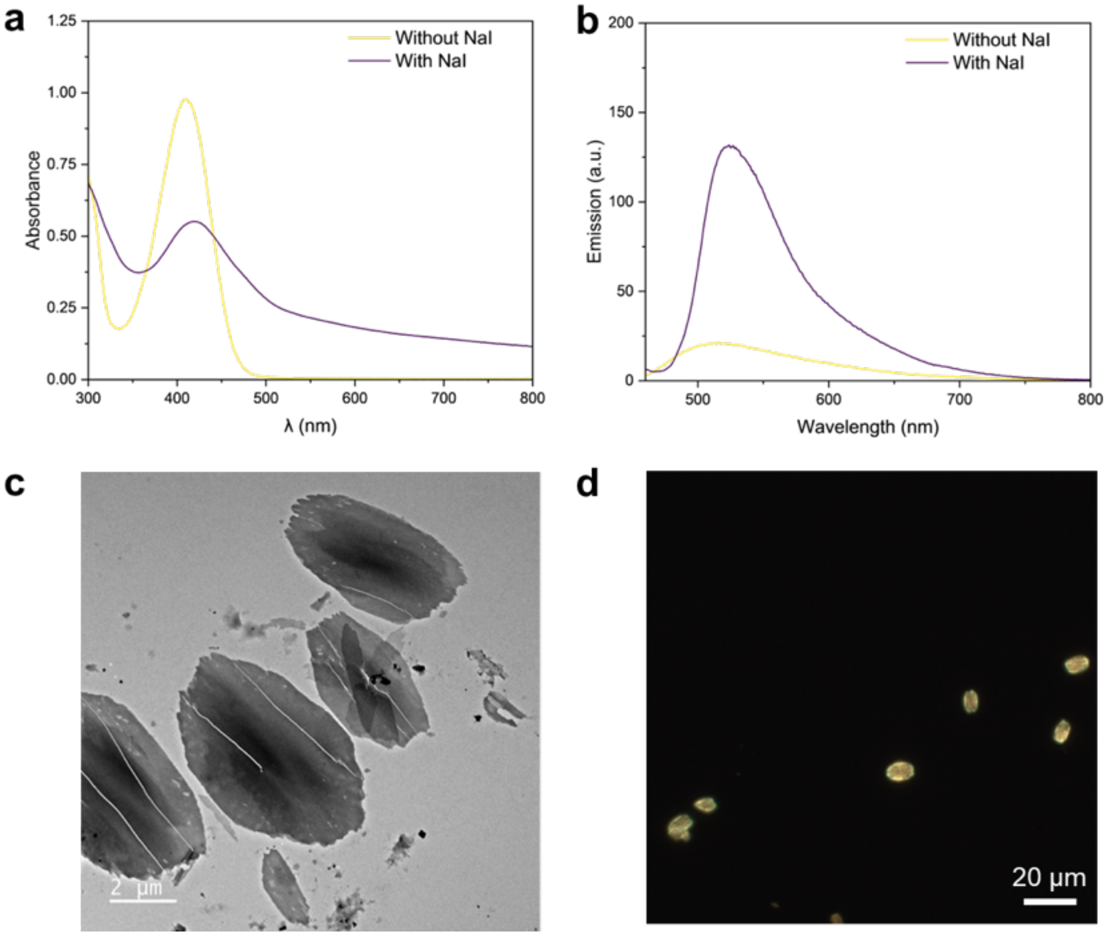

Supplementary Figure 47. Study of DSACl (0.05 mM) aggregation in the absence or presence of 3 mM NaI by monitoring a) absorption spectra, b) enhancement of fluorescence emission; and c) morphological analysis of DSACl 0.05 mM in presence of NaI 3 mM by TEM and d) widefield fluorescence microscopy.

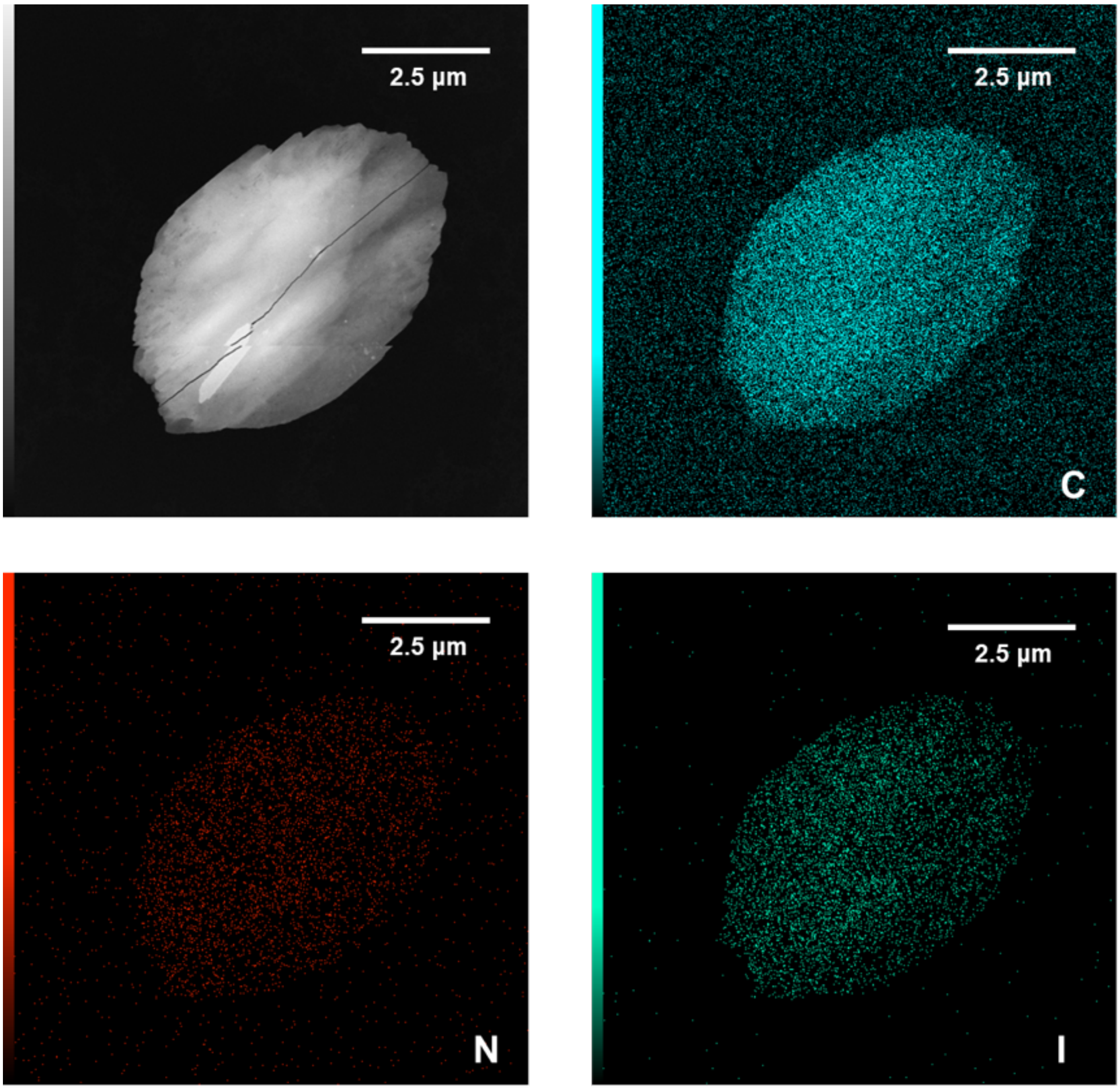

Supplementary Figure 48. TEM-EDX micrographs of 0.05 mM DSACl in the presence of 3 mM NaI in mQ-water showing the content of carbon (top right), nitrogen (bottom left), and iodide (bottom right).

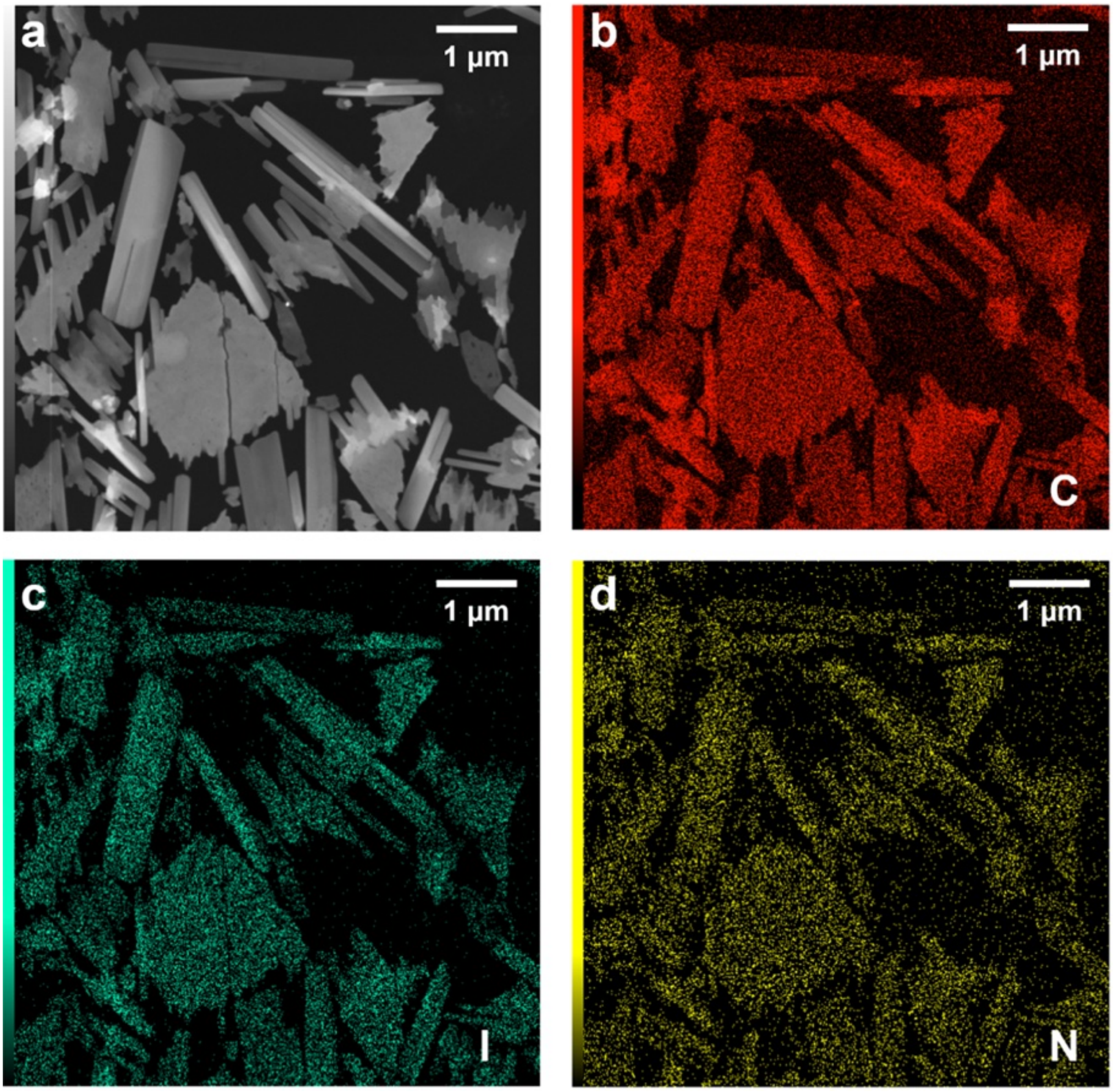

Supplementary Figure 49. TEM-EDX micrographs of 0.05 mM DSAI in the presence of 0.5 mM NaI in mQ-water showing the content of b) carbon, c) iodide, and d) nitrogen.

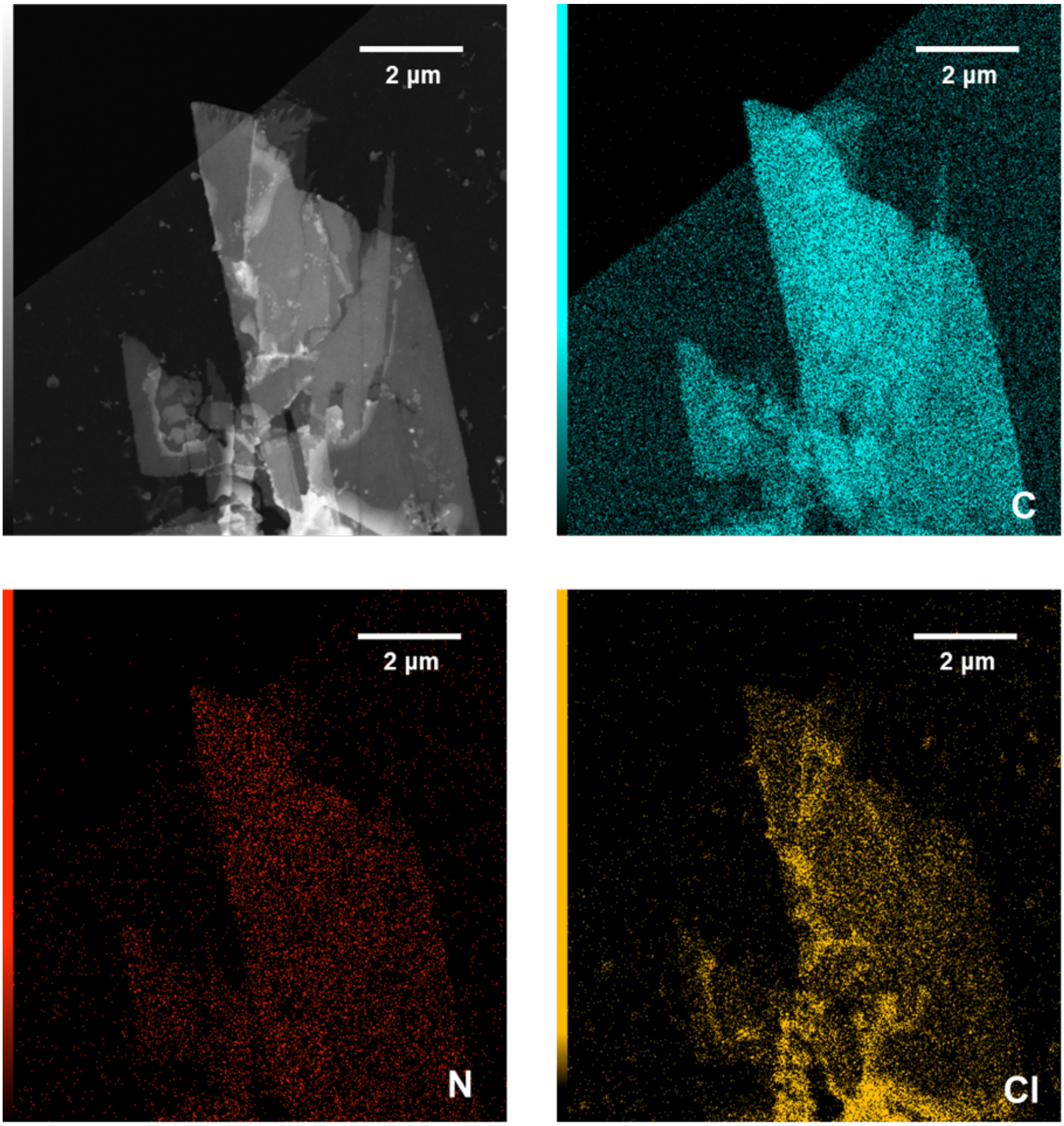

Supplementary Figure 50. TEM-EDX micrographs of 0.05 mM DSACl in the presence of 1 M NaCl in mQ-water showing the content of carbon (top right), nitrogen (bottom left), and chloride (bottom right).

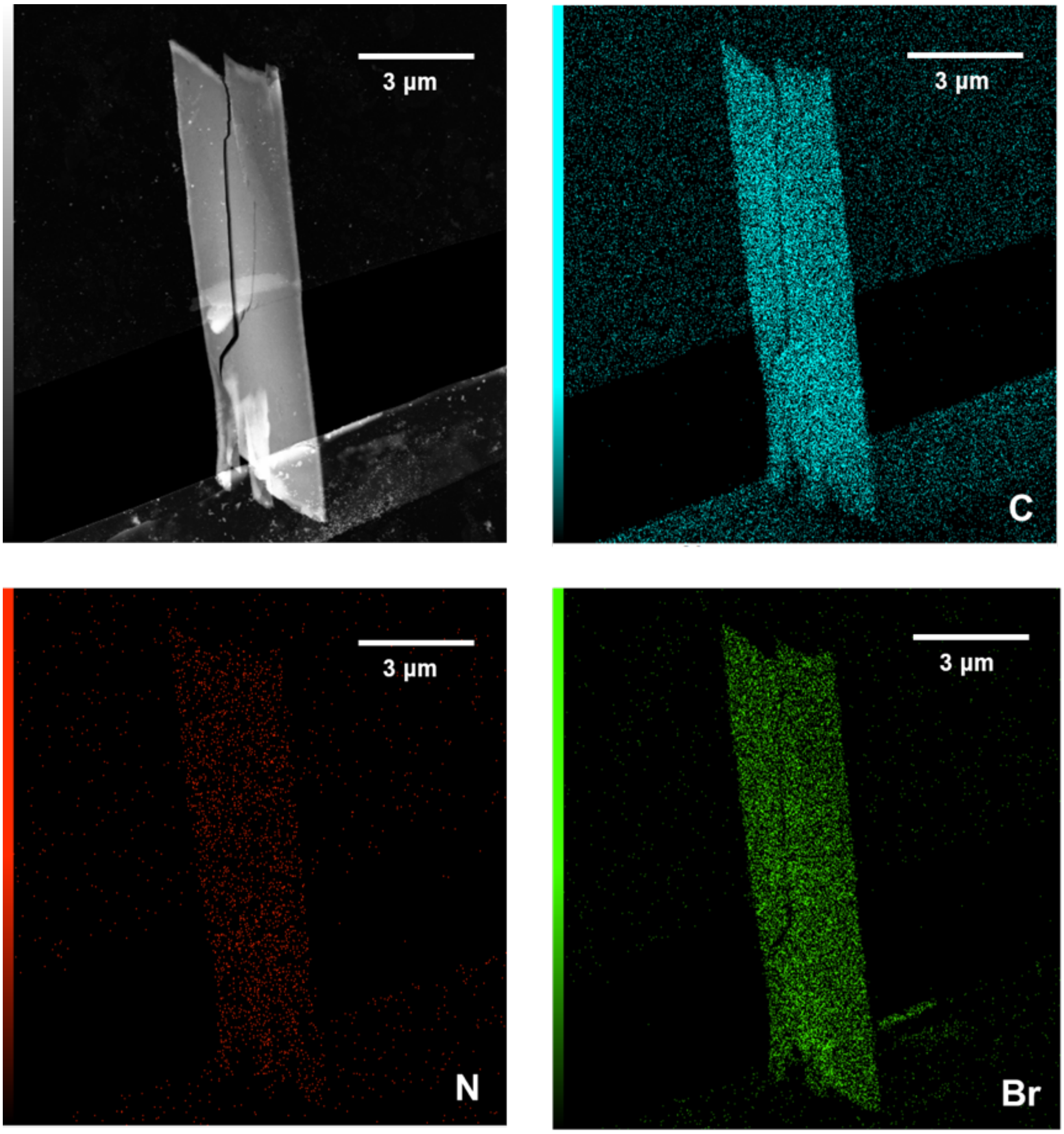

Supplementary Figure 51. TEM-EDX micrographs of 0.05 mM DSABr in the presence of 0.03 M NaBr in mQ-water showing the content of carbon (top right), nitrogen (bottom left), and bromide (bottom right).

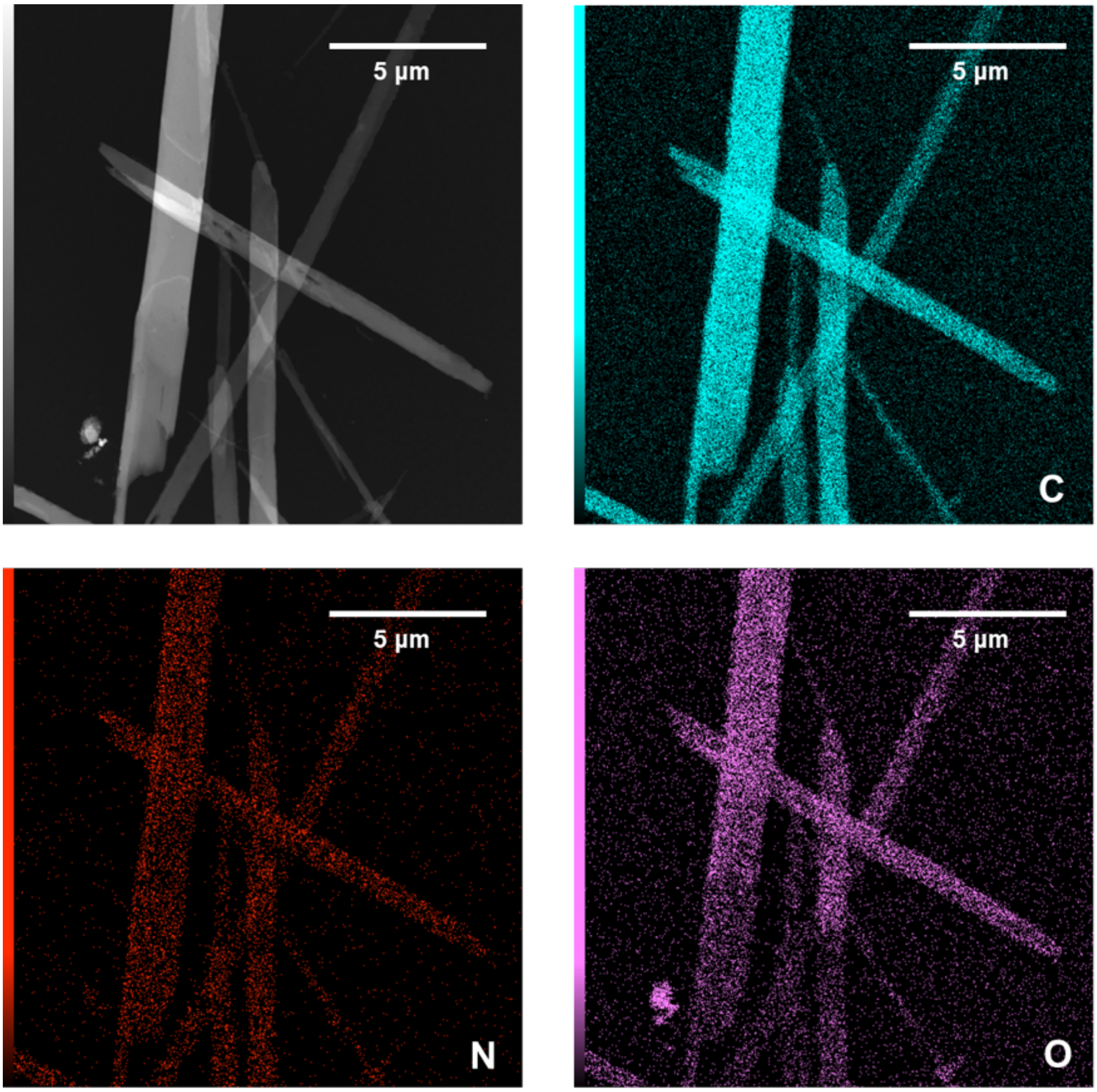

Supplementary Figure 52. TEM-EDX micrographs of 0.05 mM $DSANO_3$ in the presence of 0.1 M $NaNO_3$ in mQ-water showing the content of carbon (top right), nitrogen (bottom left), and oxygen (bottom right).

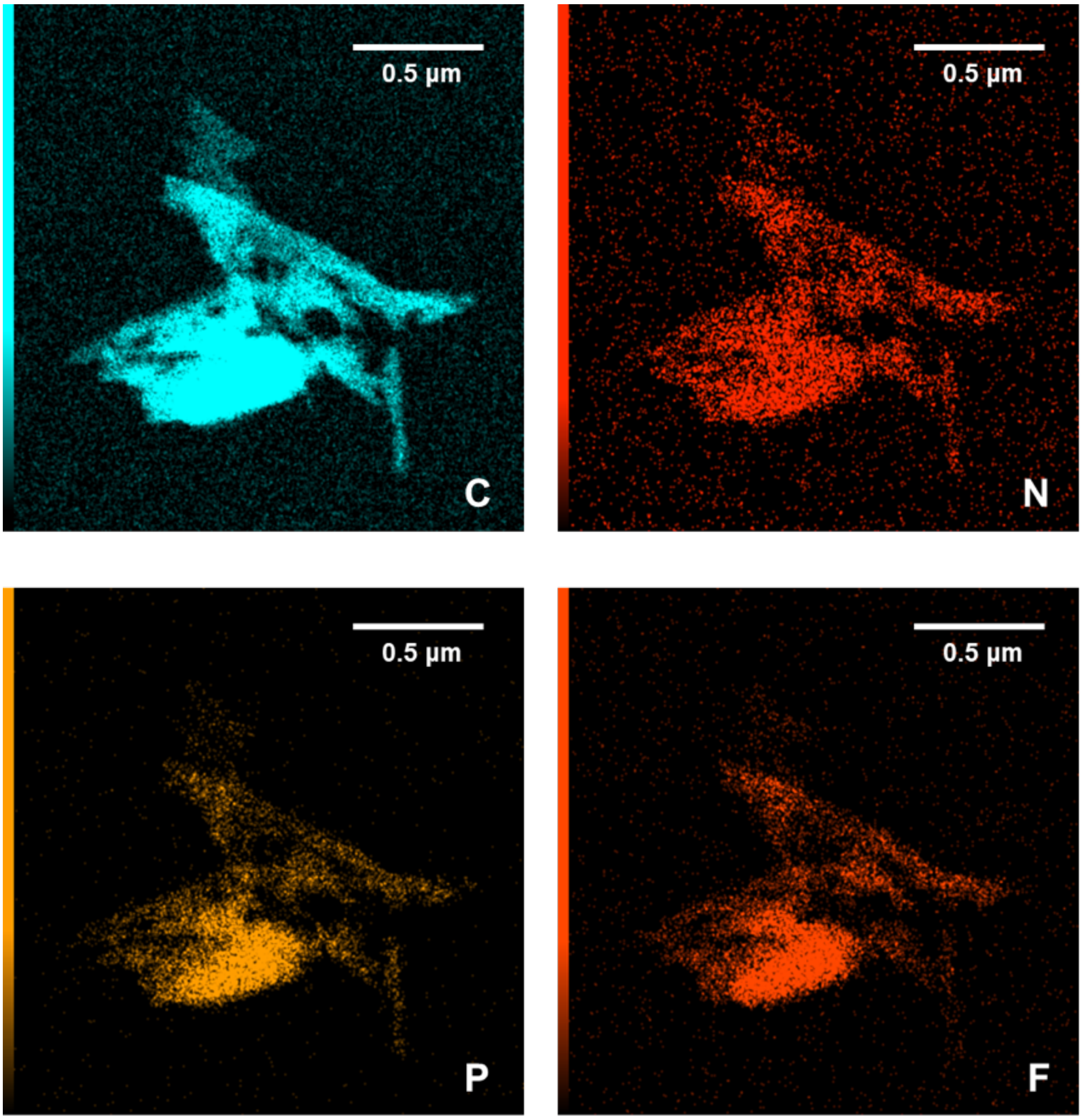

Supplementary Figure 53. TEM-EDX micrographs of 0.4 mg mL^{-1} DSAPF_6 in mQ-water nanoprecipitated from DMSO showing the content of carbon (top left), nitrogen (top right), phosphorous (bottom left) and fluorine (bottom right).

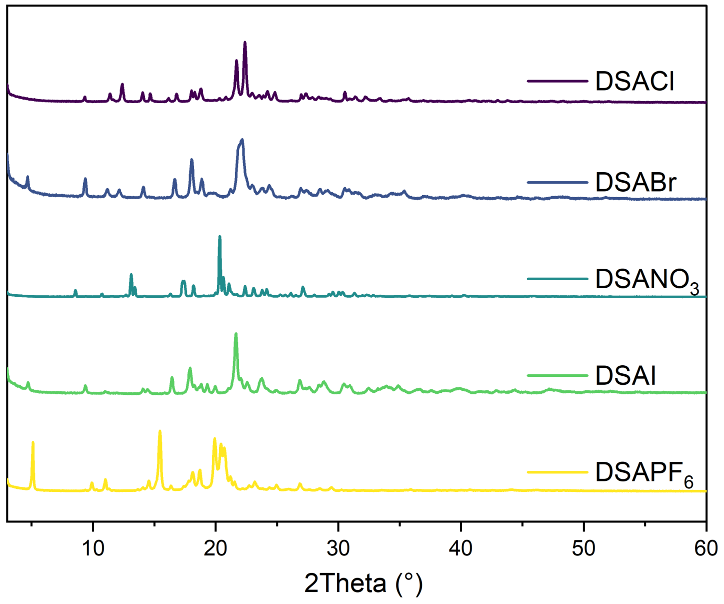

Supplementary Figure 54. PXRD diffraction patterns of DSACl (purple trace), DSABr (blue trace), $DSANO_3$ (light blue trace), DSAI (green trace), and $DSAPF_6$ (yellow trace).

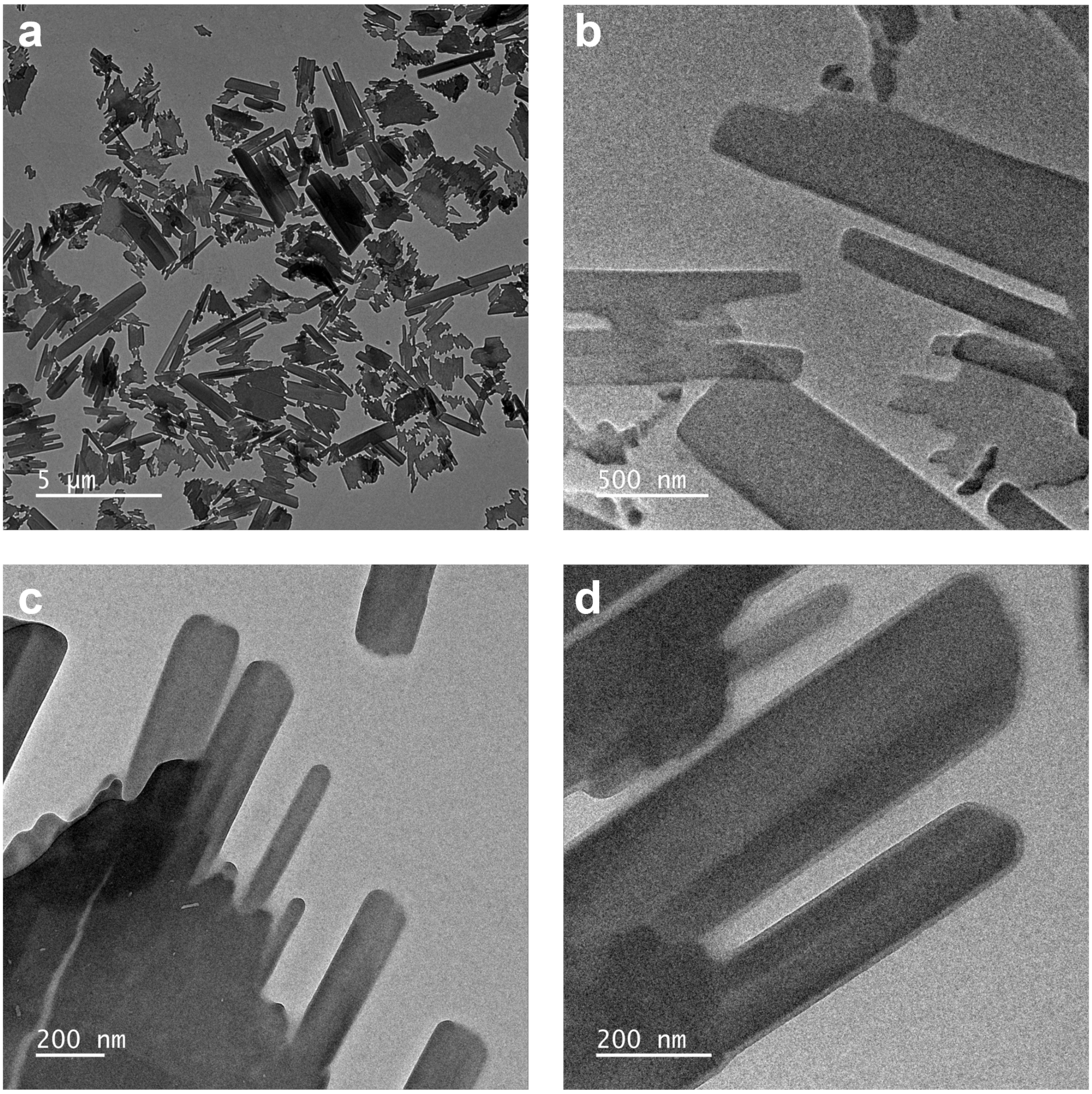

Supplementary Figure 55. TEM micrographs of 0.05 mM DSAI in the presence of 0.5 mM NaI in mQ-water. a) An ensemble of aggregates is observed. b-d) Flat elongated nanoribbons with defined and regular structure.

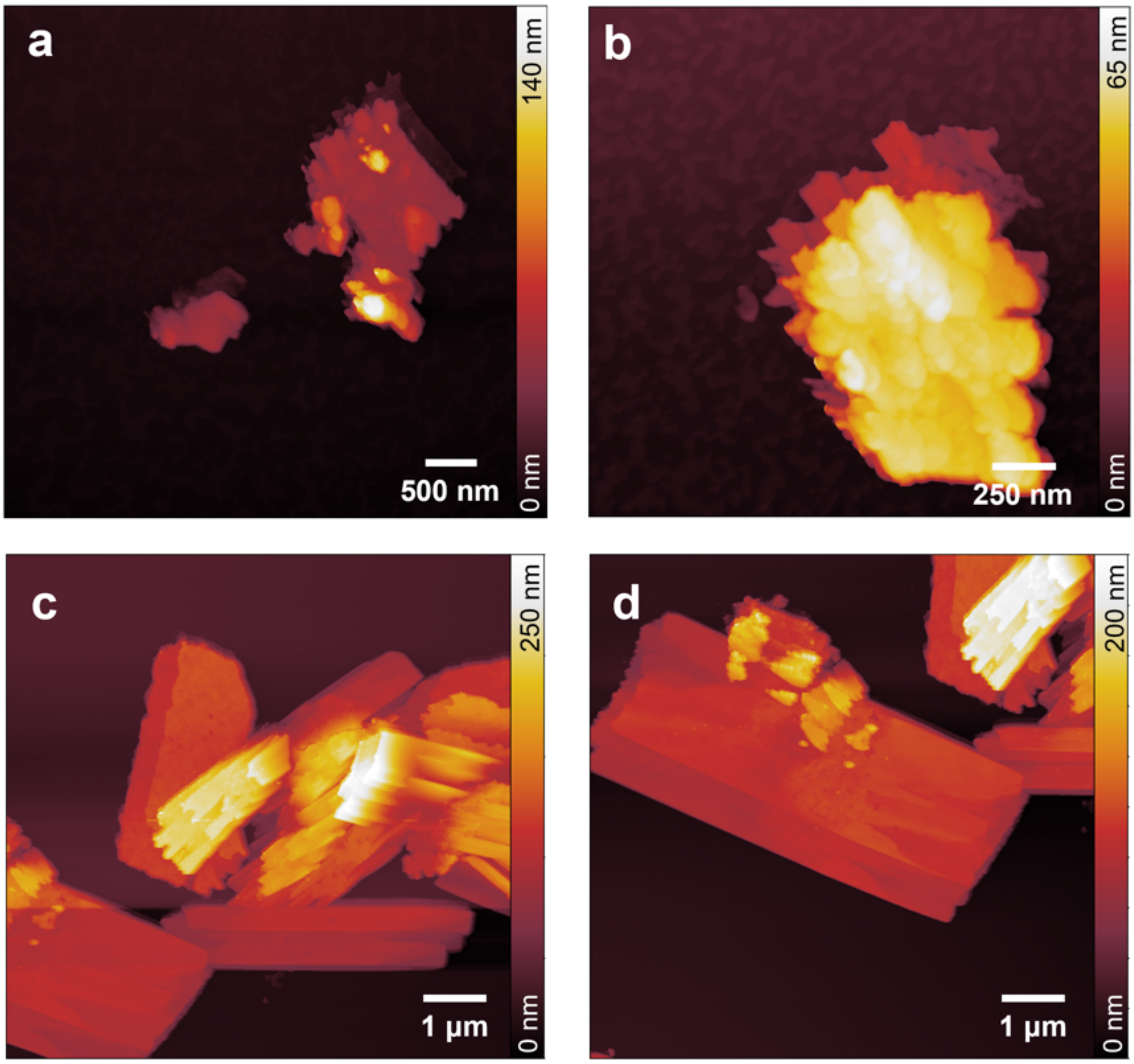

Supplementary Figure 56. AFM images of 0.05 mM DSAI in the presence of a,b) 0 mM, and c,d) 0.5 mM NaI in mQ-water. The presence of NaI leads to the formation of micrometric and ordered structures like those observed from SEM and TEM micrographs.

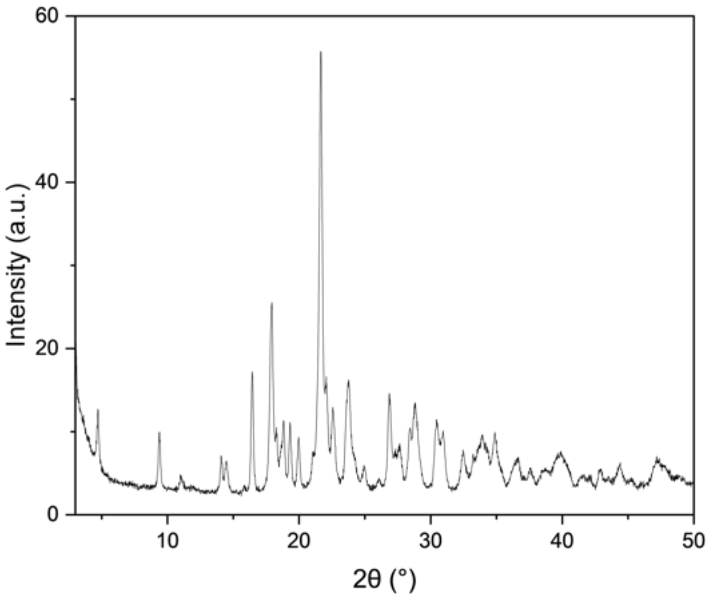

Supplementary Figure 57. PXRD diffraction pattern of 0.05 mM DSAI in presence of 5 mM NaI.

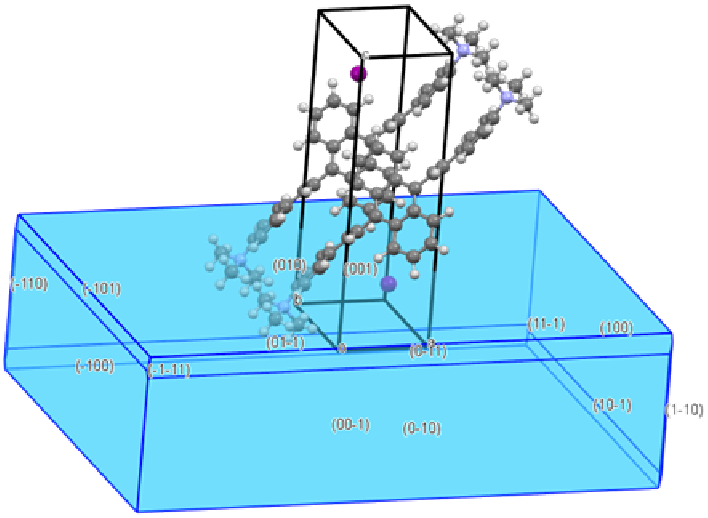

Supplementary Figure 58. Unit cell of DSAI crystal structure and simulated BFDH crystal morphology.

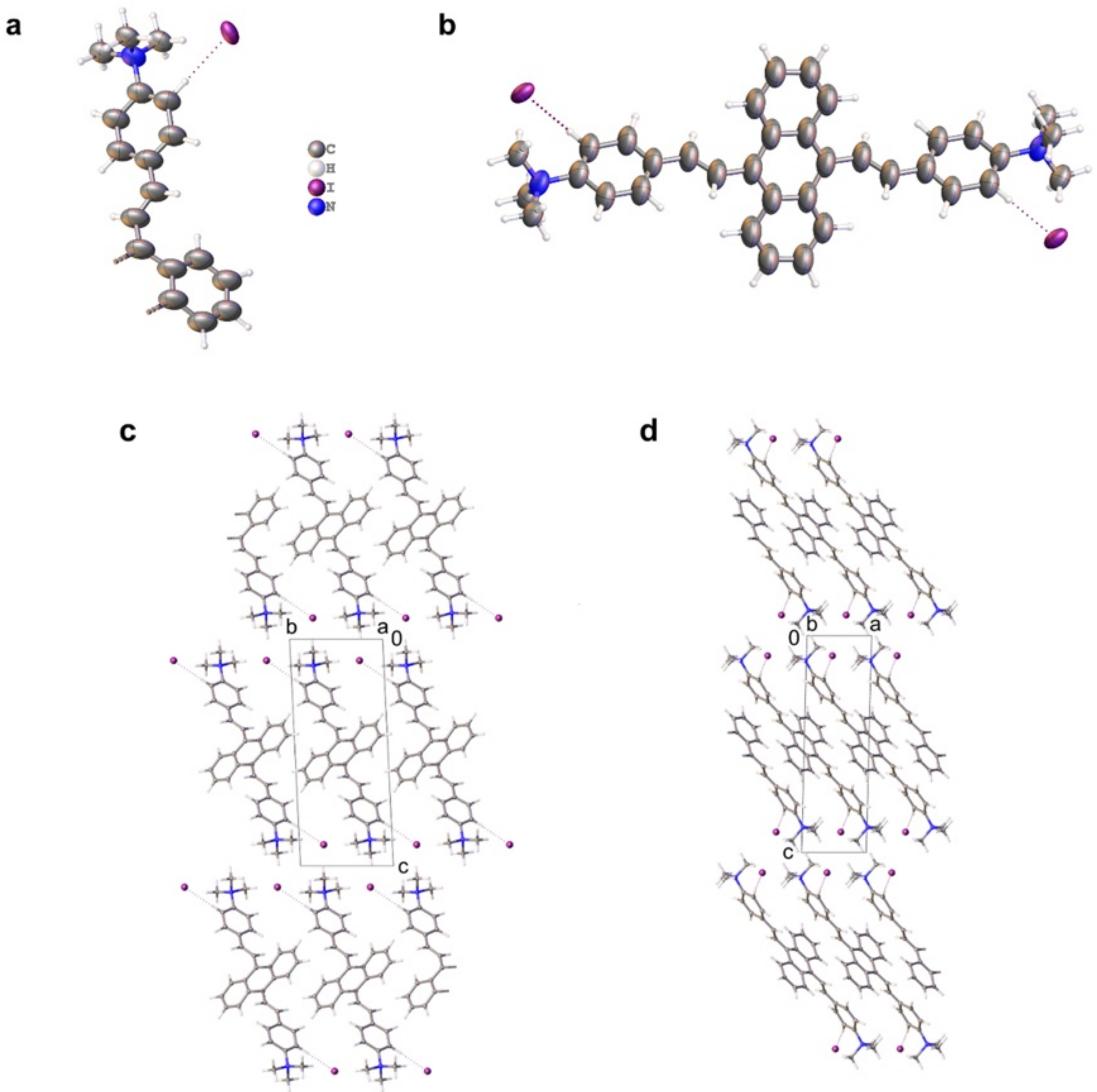

Supplementary Figure 59. a) The asymmetric unit of DSAI. b) Complete molecule. Displacement ellipsoids are drawn at the 50% probability level. c,d) Crystal packing viewed along two different crystallographic directions.

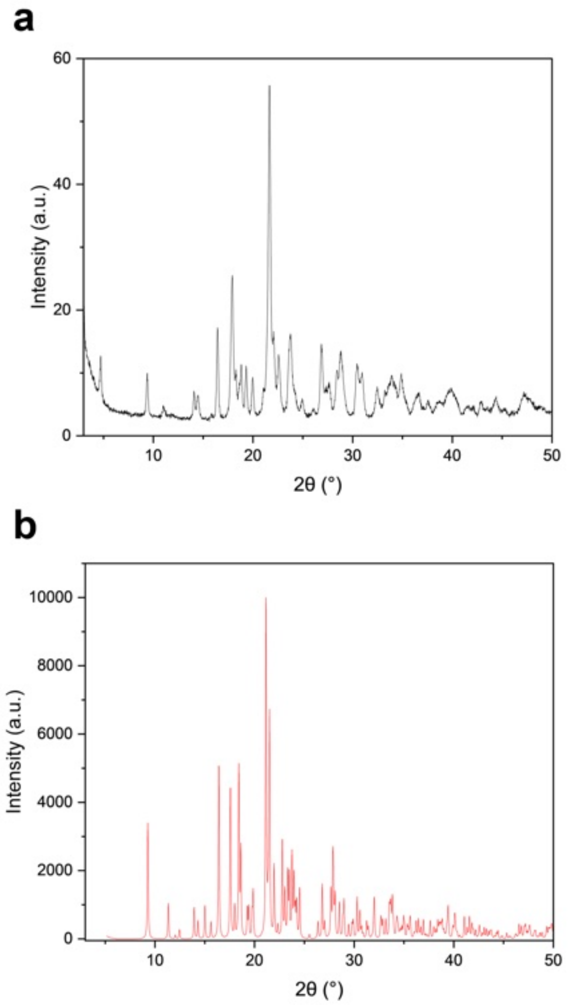

Supplementary Figure 60. Comparison between PXRD of 0.05 mM DSAI in presence of 5 mM NaI, a) experimental and b) calculated pattern from 3D ED.

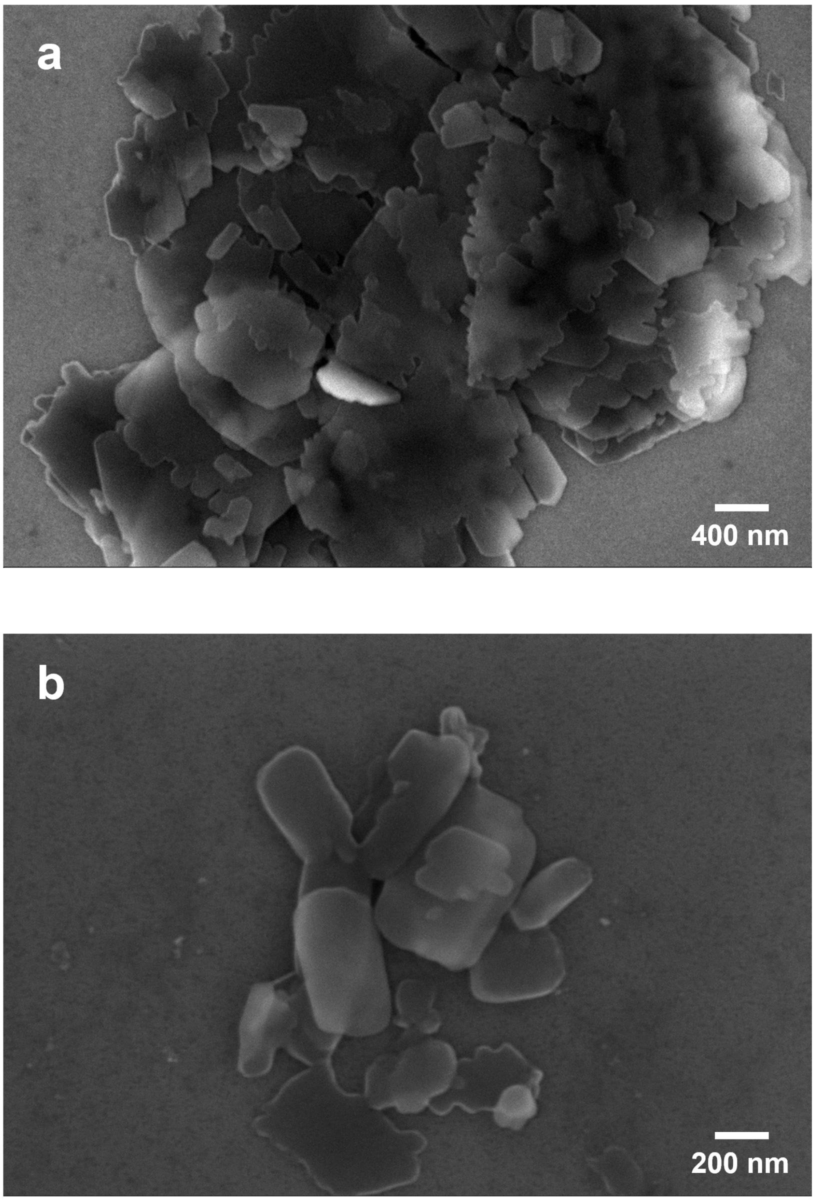

Supplementary Figure 61. SEM micrographs of 0.05 mM DSAI in the presence of 0.5 mM NaI in mQ-water. a) Ensemble of aggregates, and b) nanoribbons morphology.

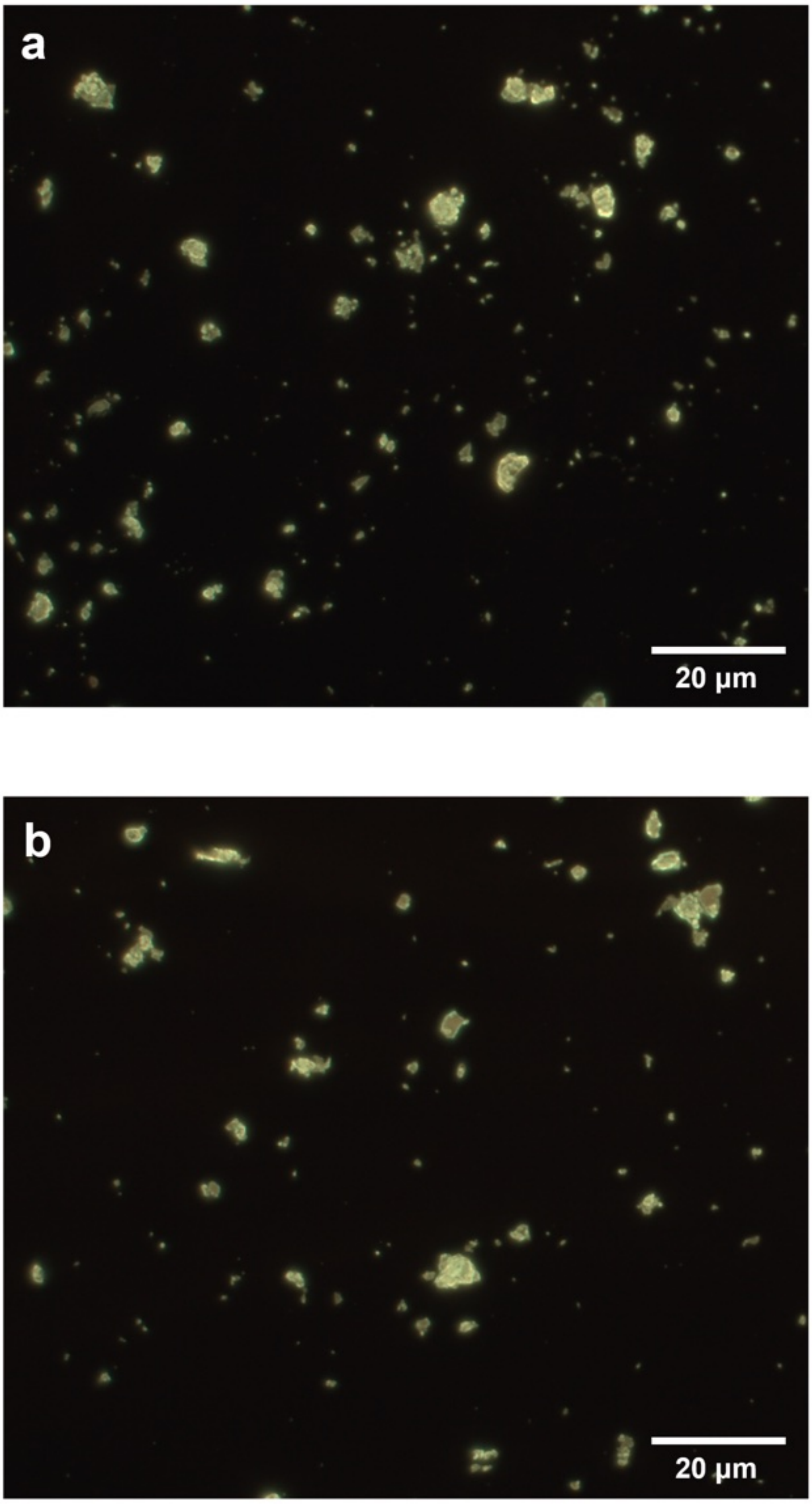

Supplementary Figure 62. a,b) Fluorescence microscopy images of 0.05 mM DSAI in the presence of 0.5 mM NaI in mQ-water. The aggregates are clearly visible due to their characteristic yellow-green emission.

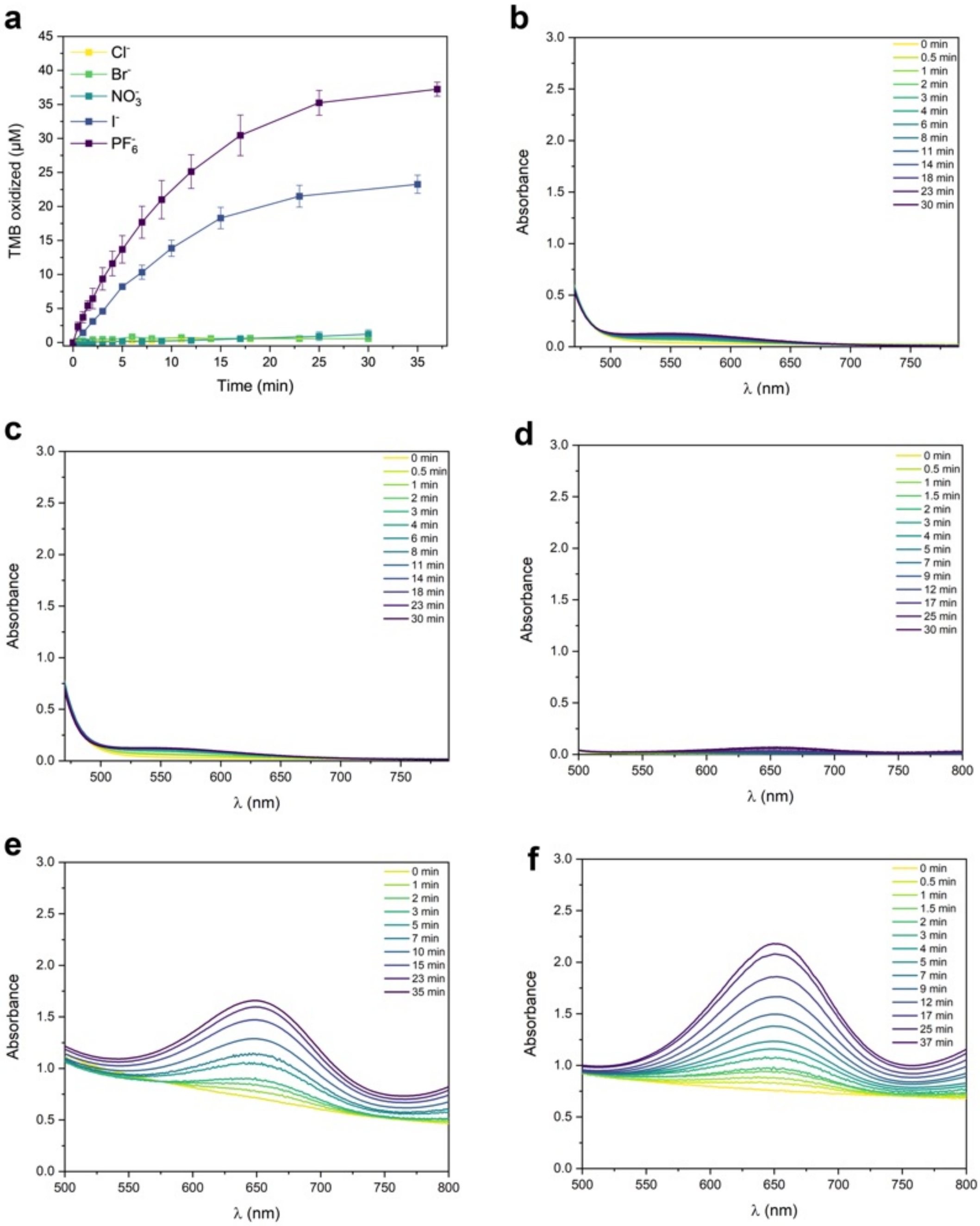

Supplementary Figure 63. a) Irradiation of DSAX (0.3 mM) in presence of TMB (134 μM) over time in acetate buffer (0.1 M, pH = 5) under white light (180 mW cm^{-2}) under air. Statistics are from 2 independent groups. b) UV-Vis traces using DSACl; c) UV-Vis traces using DSABr; d) UV-Vis traces using DSANO$_3$, e) UV-Vis traces using DSAI; f) UV-Vis traces using DSAPF$_6$.

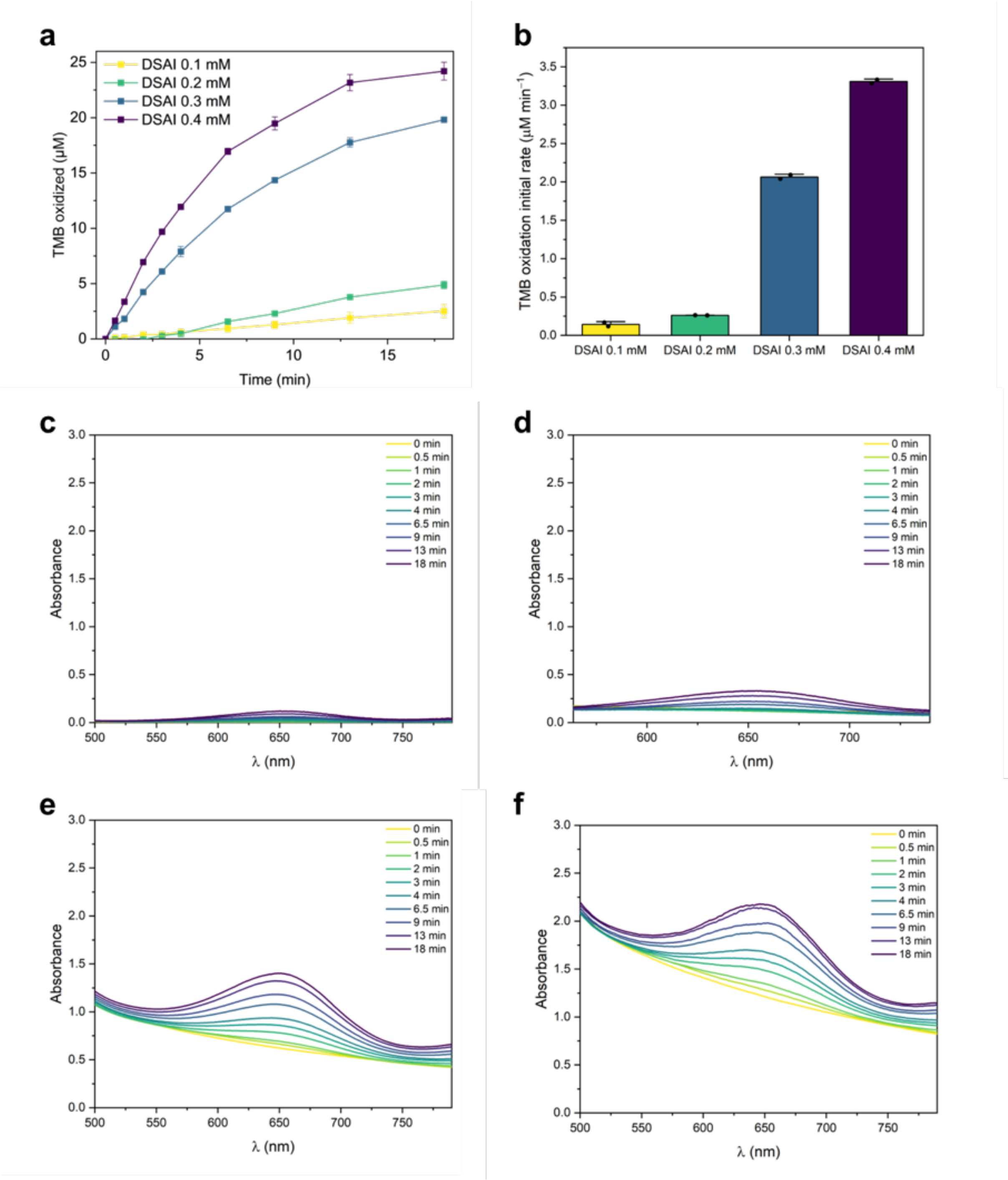

Supplementary Figure 64. a) Irradiation of DSAI (0.1–0.4 mM) in presence of TMB (134 μM) over time in acetate buffer (0.1 M, pH = 5) under white light (180 mW cm^{-2}) under air. b) TMB oxidation rate extracted from traces in panel a): 0.14 ± 0.04, 0.26 ± 0.00, 2.10 ± 0.04, 3.30 ± 0.03 μM min^{-1} (from left to right). Statistics are from 2 independent groups. c) UV-Vis traces using DSAI 0.1 mM; d) UV-Vis traces using DSAI 0.2 mM; e) UV-Vis traces using DSAI 0.3 mM; f) UV-Vis traces using DSAI 0.4 mM.

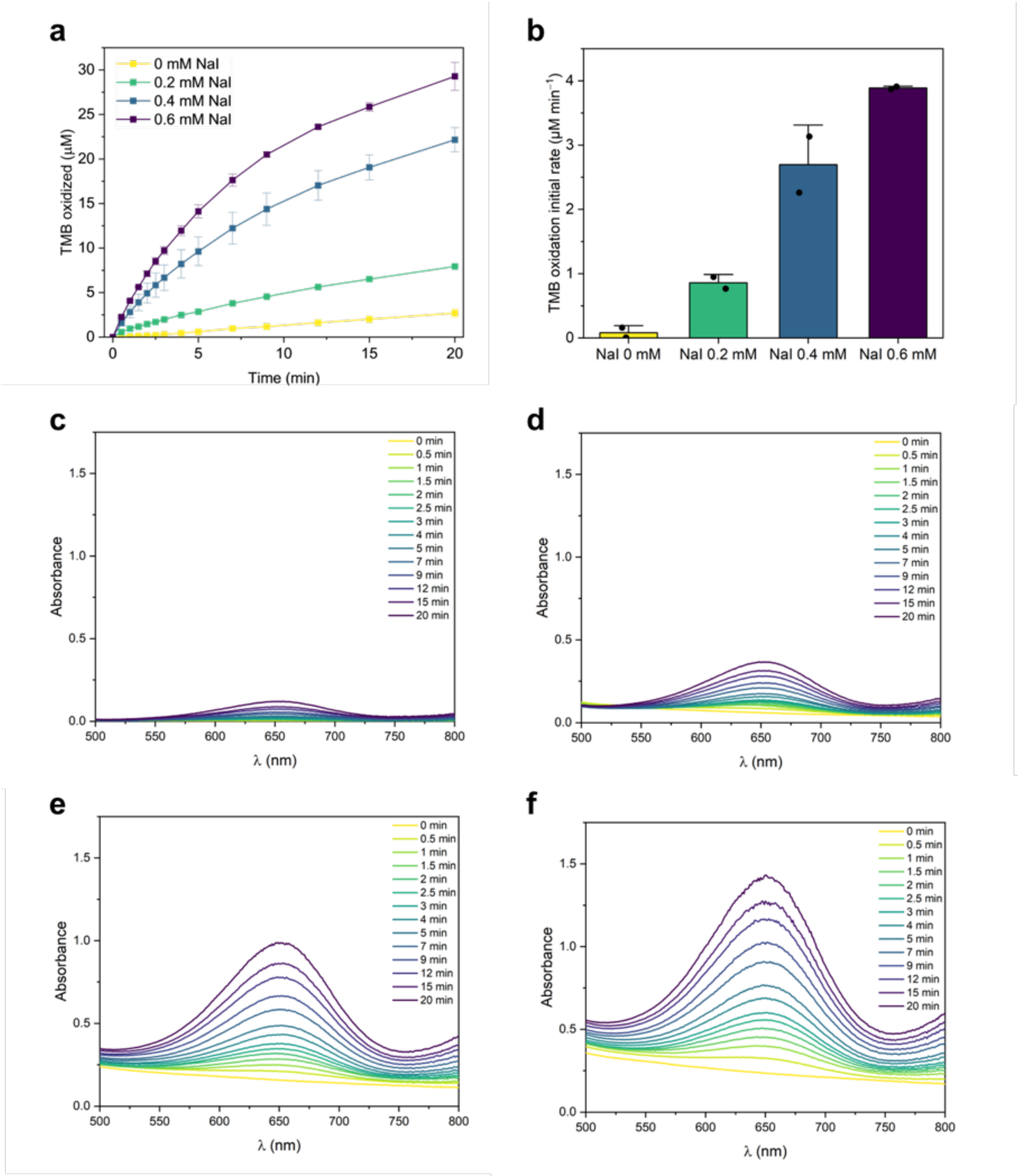

Supplementary Figure 65. a) Irradiation of DSAI (0.1 mM) aggregated with NaI (0.0–0.6 mM) in presence of TMB (134 µM) over time in acetate buffer (0.1 M, pH = 5) under white light (180 mW cm^{-2}) under air. b) TMB oxidation rate extracted from traces in panel a): 0.083 ± 0.11, 0.86 ± 0.13, 2.7 ± 0.6, 3.9 ± 0.02 µM min^{-1} (from left to right). Statistics are from 2 independent groups. c) UV-Vis traces using DSAI 0.1 mM with NaI 0.0 mM; d) UV-Vis traces using DSAI 0.1 mM with NaI 0.2 mM; e) UV-Vis traces using DSAI 0.1 mM with NaI 0.4 mM; f) UV-Vis traces using DSAI 0.1 mM with NaI 0.6 mM.

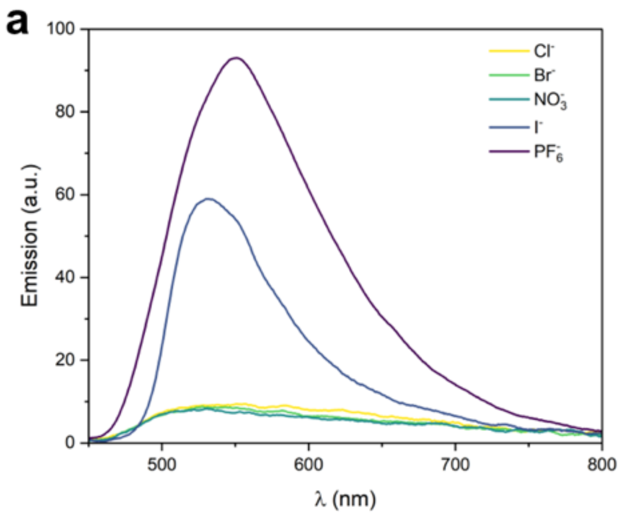

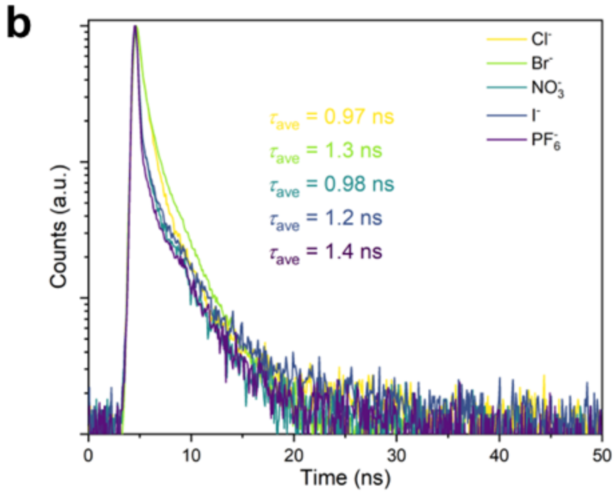

c

anions	τ_1	τ_2	τ_3
Cl^-	0.09	1.1	3.8
Br^-	0.09	1.3	4.9
NO_3^-	0.06	1.0	3.6
I^-	0.25	0.96	4.8
PF_6^-	0.13	1.2	4.1

Supplementary Figure 66. a) Emission spectra of DSAX (0.3 mM) recorded with an integrating sphere in acetate buffer (0.1 M, pH = 5) containing 3.2 % DMSO (*v*/*v*). Fluorescence quantum yields (Φ_{FL}) increase from ≤1.0% ± 0.1% (for Cl^-, Br^-, and NO_3^- salts) to 2.7% ± 0.5% and 8.7% ± 0.8% for DSAI and $DSAPF_6$, respectively. b) Time-correlated single-photon counting (TCSPC) decay traces of the same samples (λ_{exc} = 402.6 nm, λ_{em} = 520 nm). c) multiexponential fluorescence decay components.

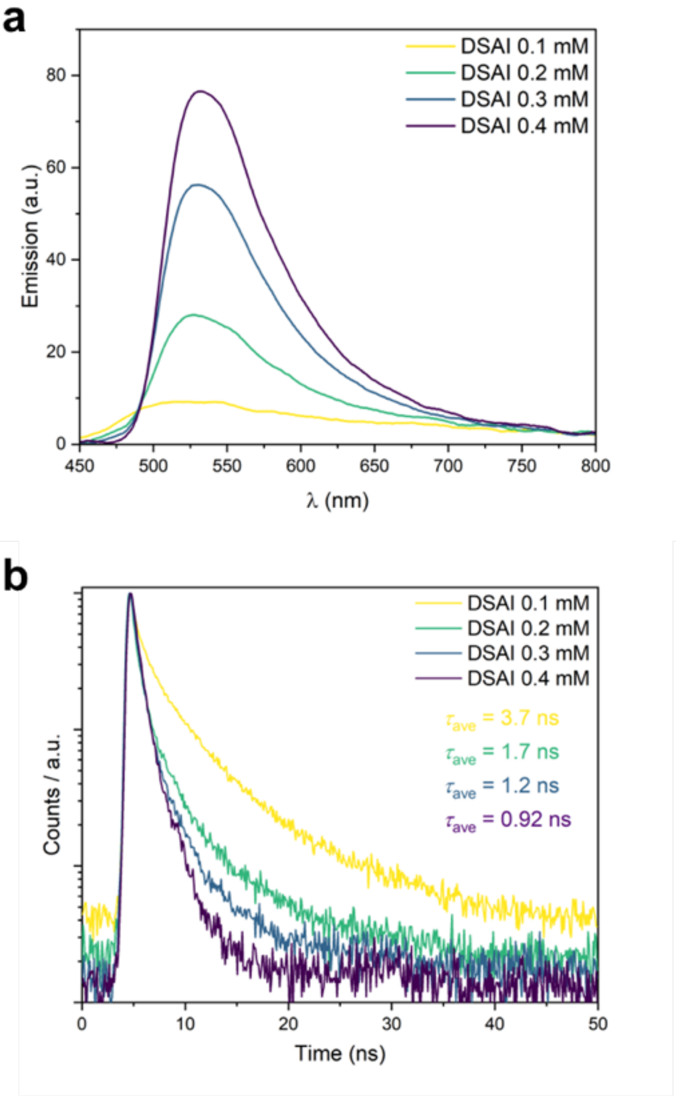

c

DSAI	τ_1	τ_2	τ_3
0.1 mM	0.25	1.8	6.4
0.2 mM	0.23	1.1	5.4
0.3 mM	0.25	0.96	4.8
0.4 mM	0.43	1.1	4.5

Supplementary Figure 67. a) Emission spectra registered with integrating sphere of different concentrations of DSAI in acetate buffer (0.1 M, pH = 5) containing 3.2 % DMSO (*v*/*v*). Fluorescence quantum yields (Φ_{FL}) from ≤1.0% ± 0.9% for 0.1 mM to 4.8% ± 0.4% for 0.4 mM. DSAI 0.2 and 0.3 mM showed Φ_{FL} of 2.0% ± 0.2% and 3.6% ± 0.4%, respectively. b) Time-correlated single-photon counting (TCSPC) measurements (λ_{exc} = 402.6 nm, λ_{em} = 520 nm) of same samples. c) multiexponential fluorescence decay components.

a

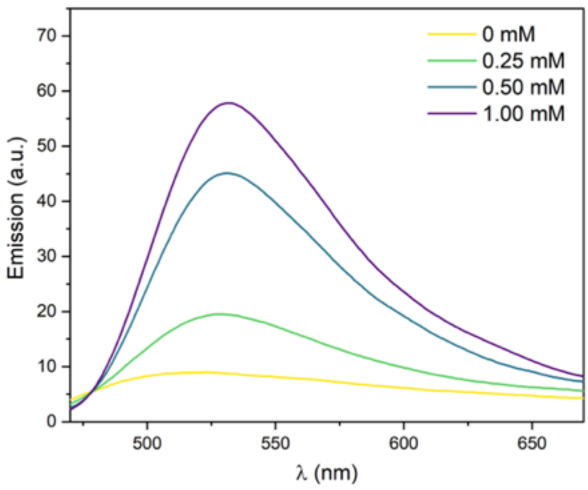

b

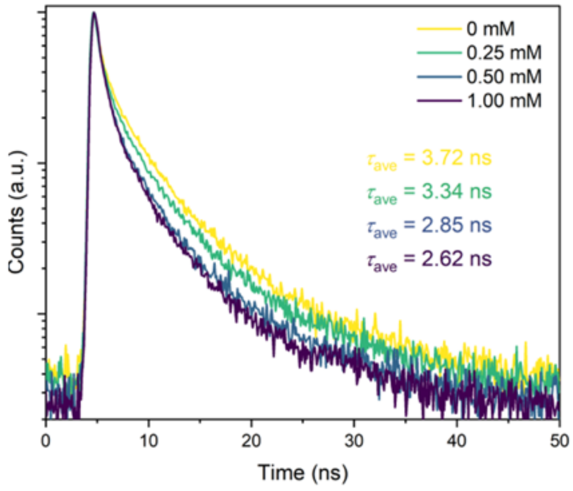

c

NaI	τ_1	τ_2	τ_3
0 mM	0.30	1.9	6.5
0.25 mM	0.28	1.8	6.4
0.5 mM	0.46	1.9	6.6
1.0 mM	0.44	1.8	6.3

Supplementary Figure 68. a) Emission spectra registered with integrating sphere of DSAI 0.1 mM in acetate buffer (0.1 M, pH = 5.0) containing 3.2 % DMSO (*v*/*v*) with different concentrations of NaI. Fluorescence quantum yields (Φ_{FL}) from 1% ± 0.1% for NaI 0.0 mM to 4.3% ± 0.3% for NaI 1.0 mM. DSAI 0.1 with NaI 0.25 and 0.4 mM showed Φ_{FL} of 2.3% ± 0.4% and 2.5% ± 0.3%, respectively. b) Time-correlated single-photon counting (TCSPC) measurements (λ_{exc} = 402.6 nm, λ_{em} = 520 nm) of same samples. c) multiexponential fluorescence decay components.

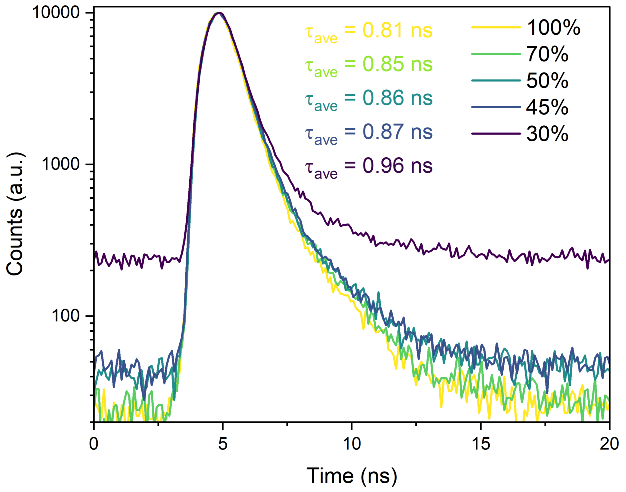

Supplementary Figure 69. Time-correlated single-photon counting (TCSPC) measurements (λ_{exc} = 402.6 nm, λ_{em} = 520 nm) of DSAI 0.1 mM with NaI 1 mM by tuning the intensity of the exciting laser.

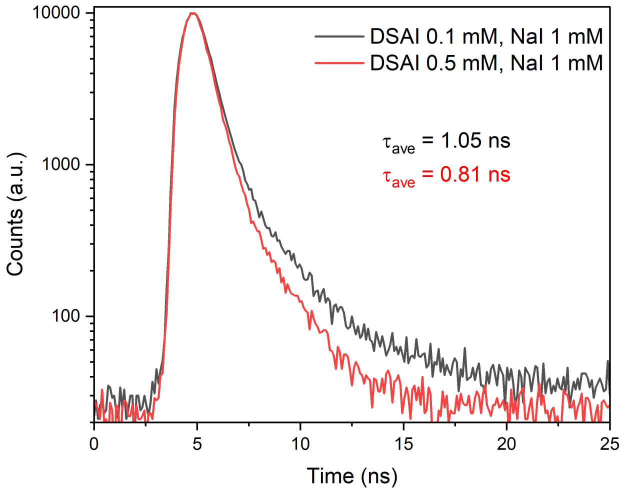

Supplementary Figure 70. Time-correlated single-photon counting (TCSPC) measurements (λ_{exc} = 402.6 nm, λ_{em} = 520 nm) of DSAI 0.1 mM or 0.5 mM with NaI 1 mM.

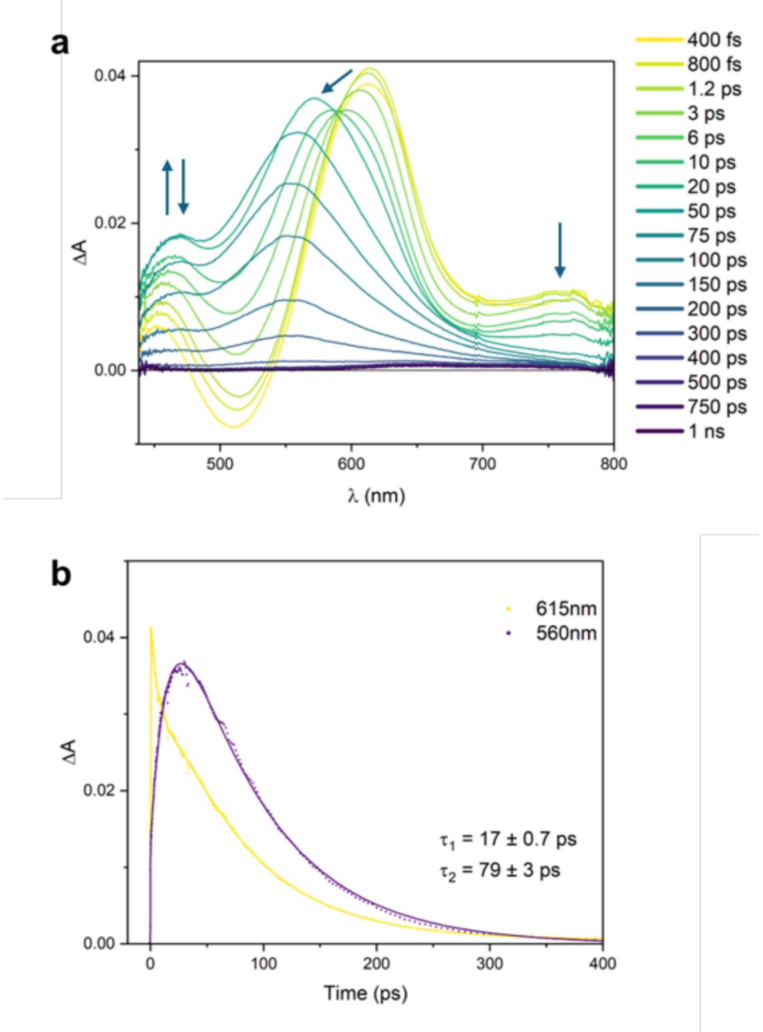

Supplementary Figure 71. a) Transient absorption spectra at different delays of an aqueous solution of DSAI 0.05 mM. The arrows indicate the spectral changes associated with the different processes. b) Transient absorption kinetic traces at significant wavelengths together with the corresponding fitting decays (solid lines). λ_{exc} = 420 nm. $A_{420\ nm}$ = 0.22, 0.2 cm optical path, 4 μJ per pulse.

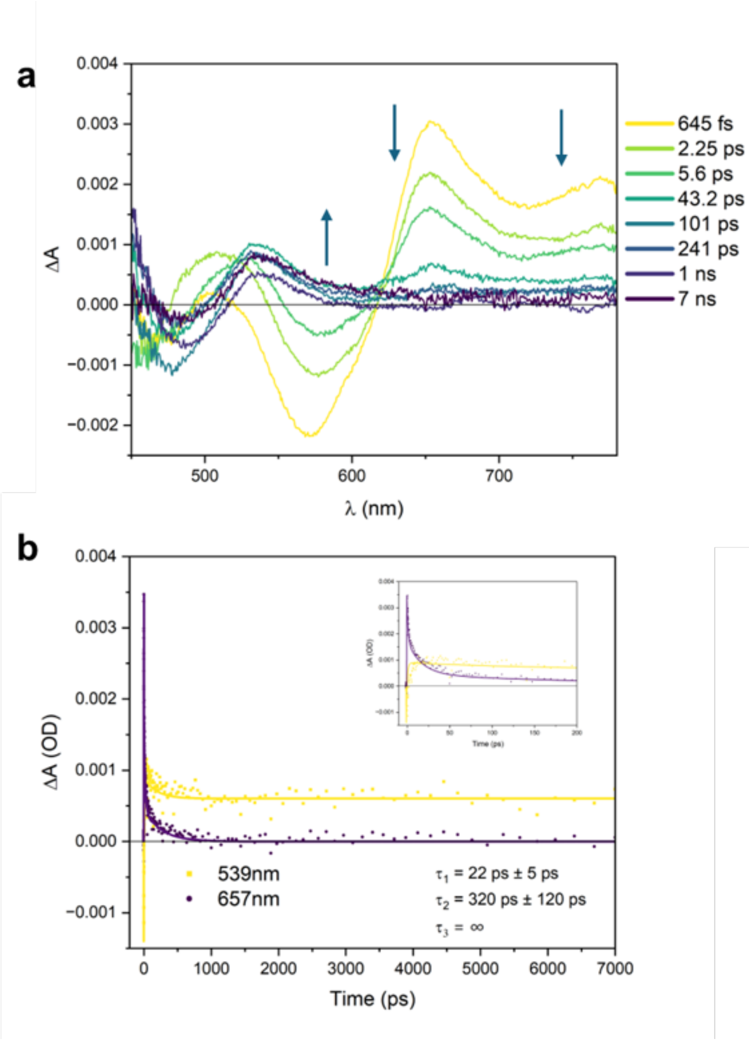

Supplementary Figure 72. a) Transient absorption spectra at different delays of an aqueous solution of aggregates of DSAI 0.05 mM and 2 mM of NaI. The arrows indicate the spectral changes associated with the different processes. b) Transient absorption kinetic traces at significant wavelengths together with the corresponding fitting decays (solid lines). λ_{exc} = 420 nm. $A_{420\ nm}$ = 0.22, 0.2 cm optical path, 4 μJ per pulse.

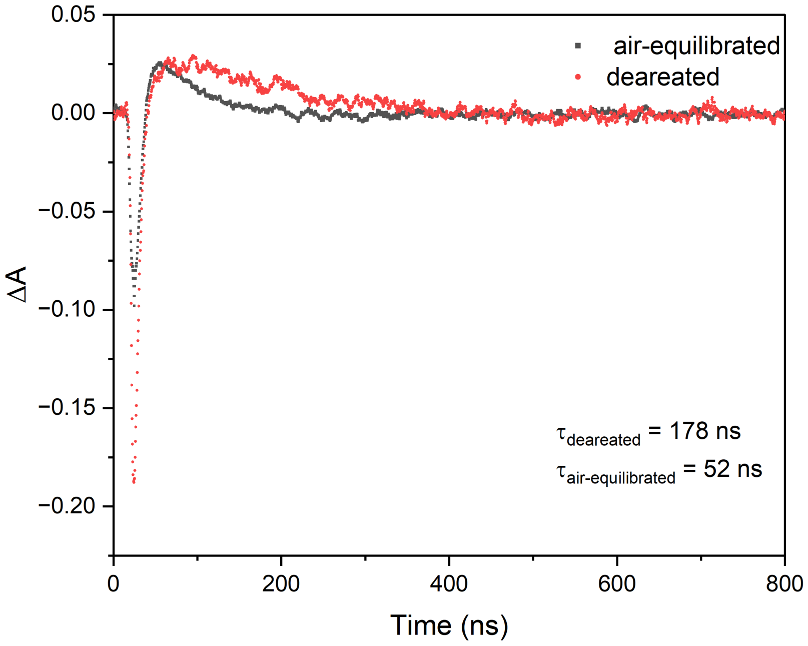

Supplementary Figure 73. Transient absorption kinetic traces at 420 nm of an aqueous solution of aggregates of DSAI 0.05 mM and 2 mM of NaI in air-equilibrated (black) and deareated (red) condition. λ_{exc-} = 355 nm. $A_{355\ nm}$ = 0.31, 1 cm optical path, 4.4 mJ per pulse.

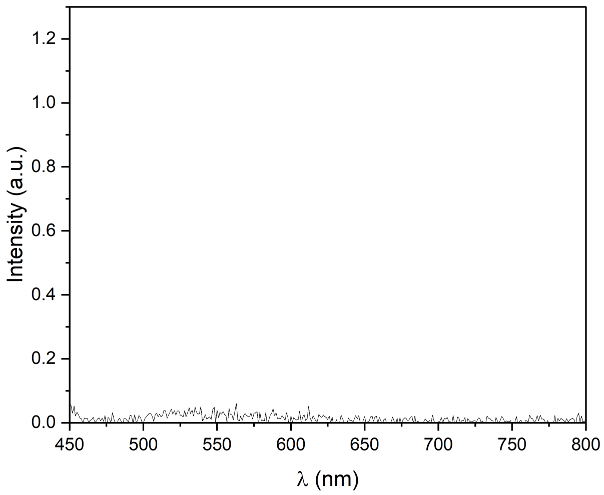

Supplementary Figure 74. Phosphorescence spectrum of DSAI powder measured at 77 K.

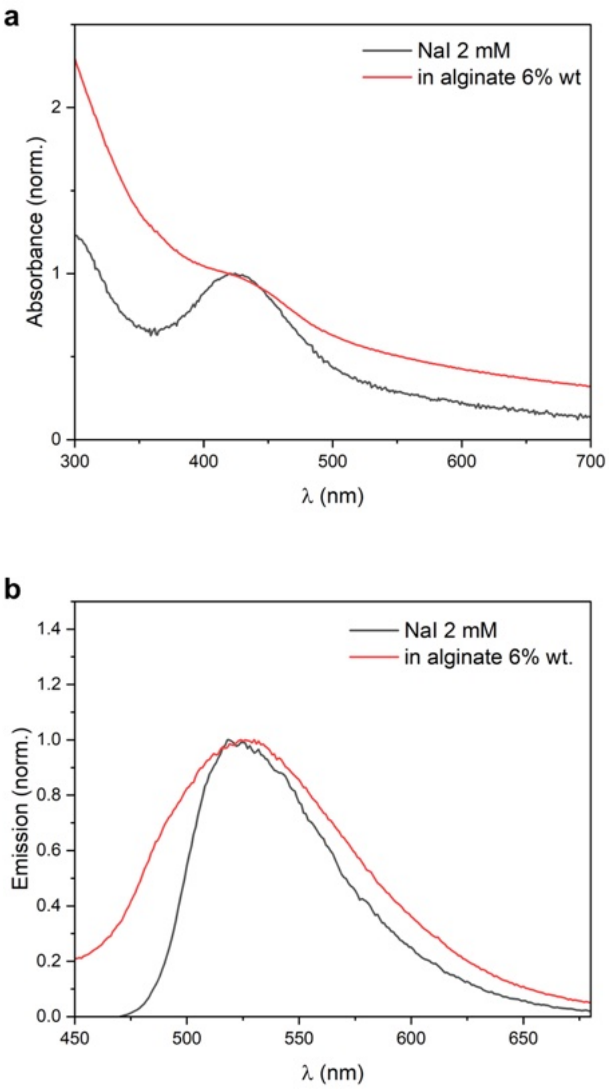

Supplementary Figure 75. Normalized a) absorption and b) emission spectra of DSAI 50 μM with NaI (2 mM, black trace) and sodium alginate (6% wt., red trace).

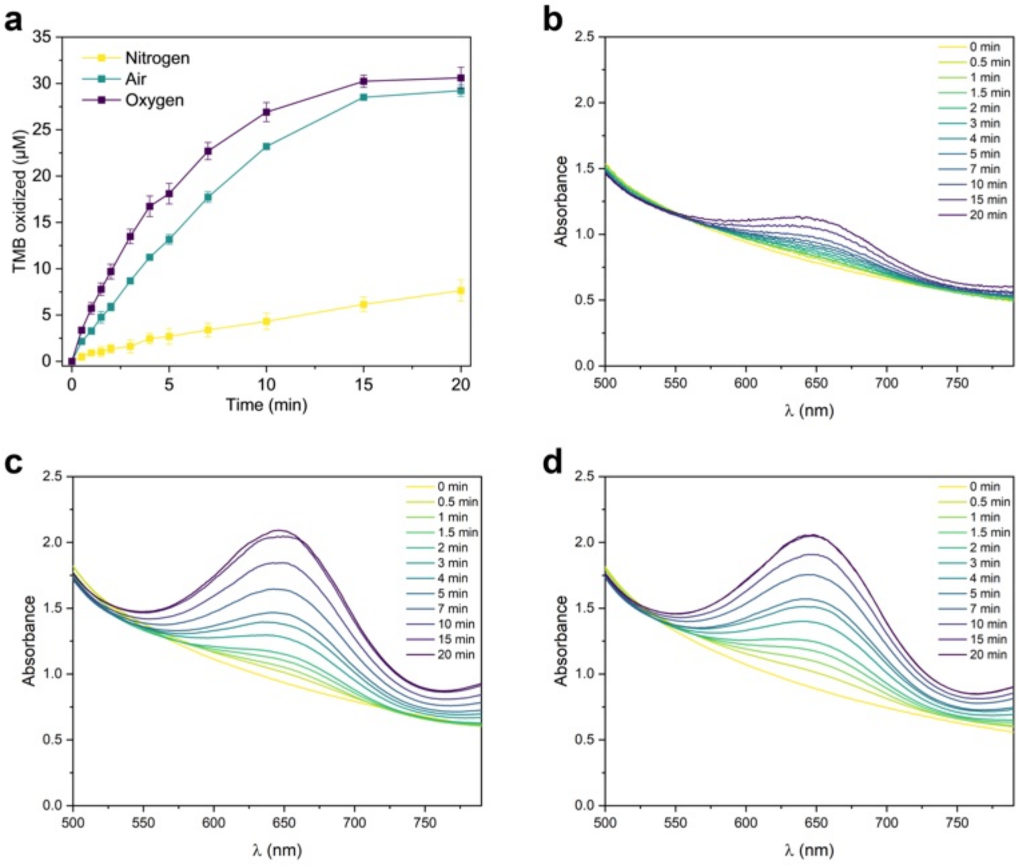

Supplementary Figure 76. a) Irradiation of DSAI (0.3 mM) in presence of TMB (134 μM) over time in acetate buffer (0.1 M, pH = 5) under white light (180 mW cm^{-2}) under different atmospheres. Statistics are from 2 independent groups. b) UV-Vis traces using DSAI 0.3 mM under nitrogen; c) UV-Vis traces using DSAI 0.3 mM under air; d) UV-Vis traces using DSAI 0.3 mM under oxygen.

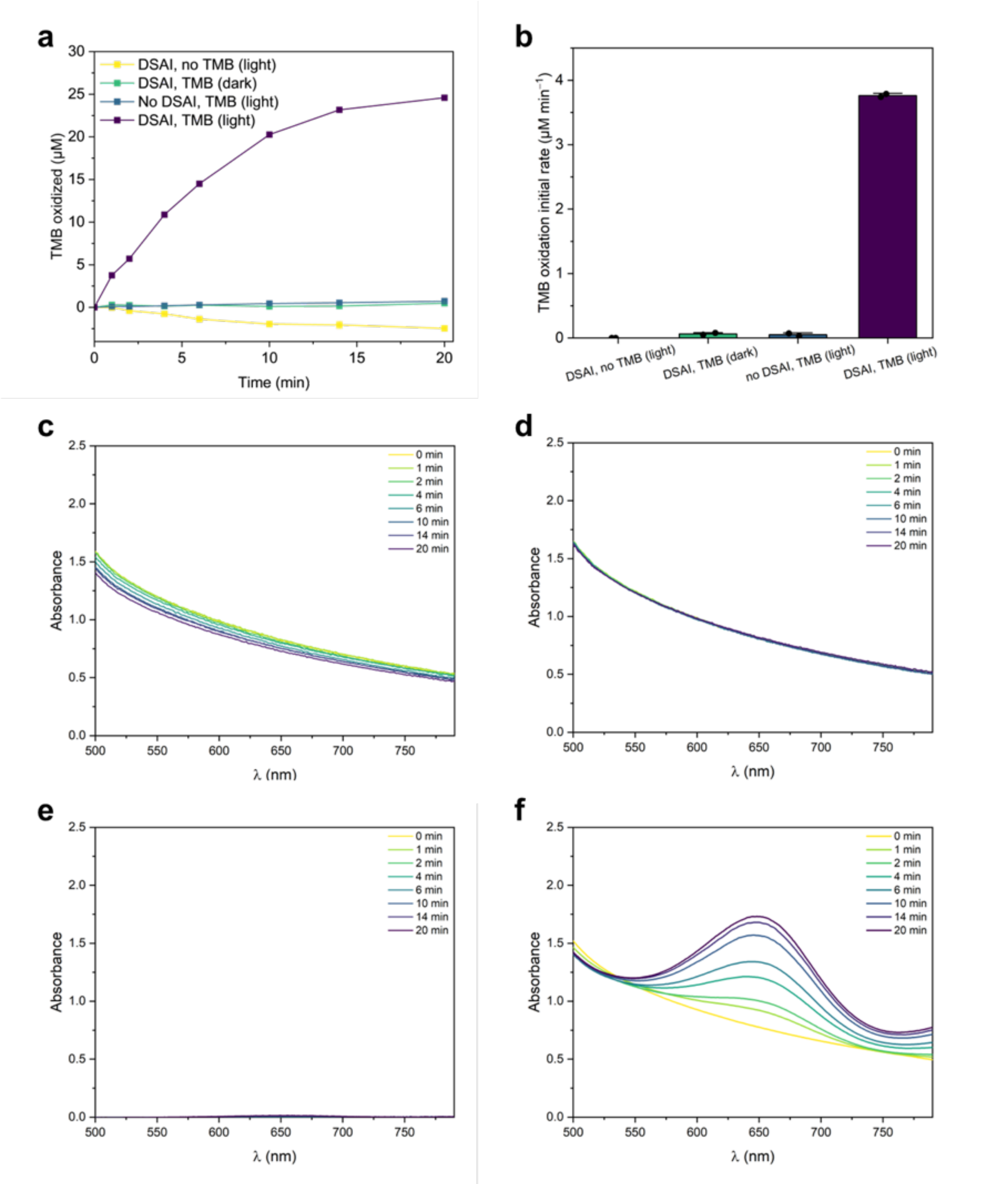

Supplementary Figure 77. a) Control experiments for irradiation of DSAI (0.3 mM) in presence/absence of TMB (134 μM) over time in acetate buffer (0.1 M, pH = 5) under white light (180 mW cm^{-2}) or dark under air. b) TMB oxidation rate extracted from traces in panel a): 0.00 ± 0.00, 0.064 ±0.02, 0.050 ± 0.03, 3.8 ± 0.03 μM min^{-1} (from left to right). Statistics are from 2 independent groups. c) UV-Vis traces in absence of TMB; d) UV-Vis traces under dark; e) UV-Vis traces without DSAI; f) UV-Vis traces of the positive control.

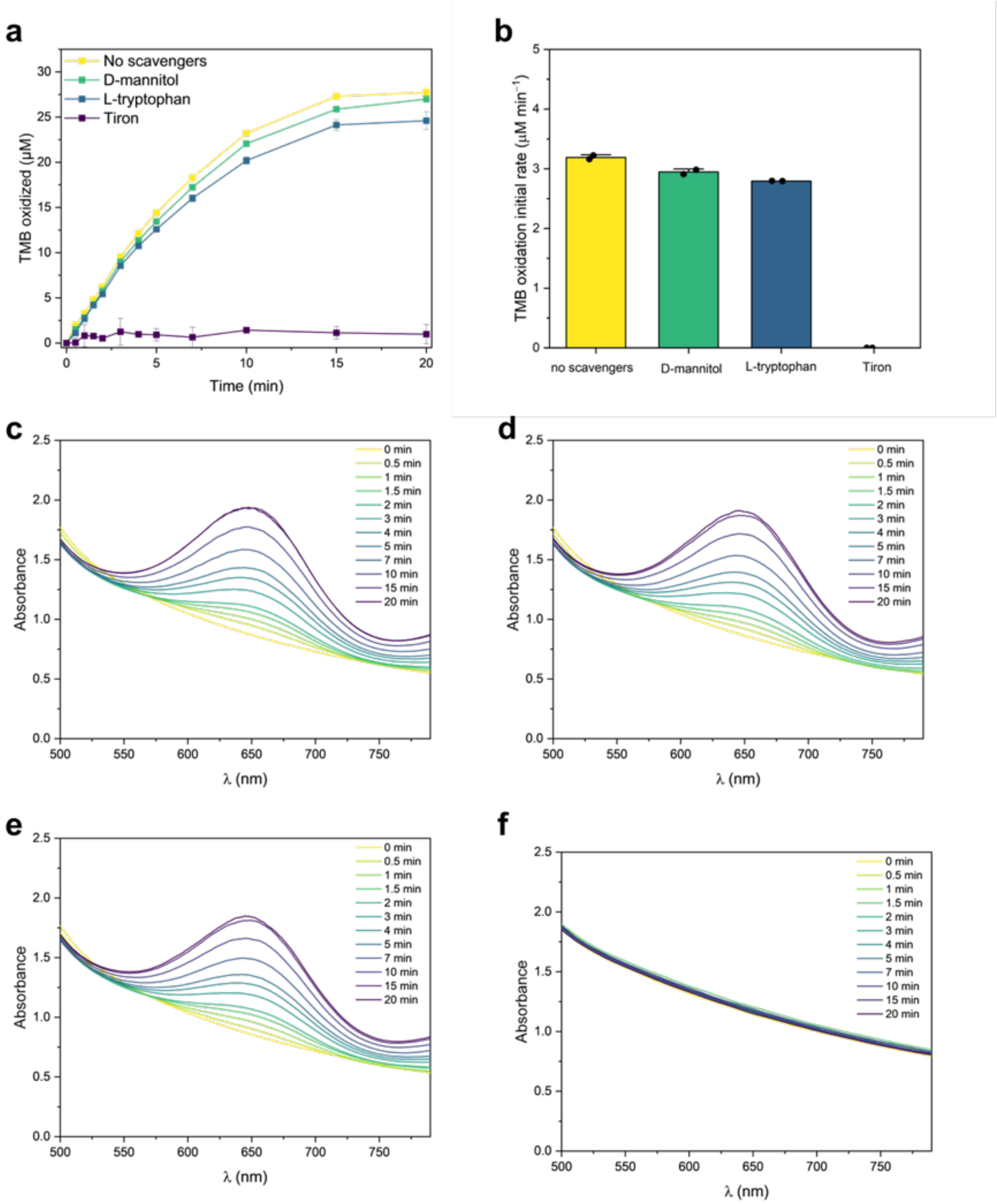

Supplementary Figure 78. a) Irradiation of DSAI (0.3 mM) in presence of TMB (134 μM) over time in acetate buffer (0.1 M, pH = 5) under white light (180 mW cm^{-2}) under air. b) TMB oxidation rate extracted from traces in panel a): 3.19 ± 0.04, 2.95 ± 0.05, 2.79 ± 0.00, 0.00 ± 0.00 μM min^{-1} (from left to right). Statistics are from 2 independent groups. c) UV-Vis traces without scavengers; d) UV-Vis traces using D-mannitol; e) UV-Vis traces using L-tryptophan; f) UV-Vis traces using Tiron.

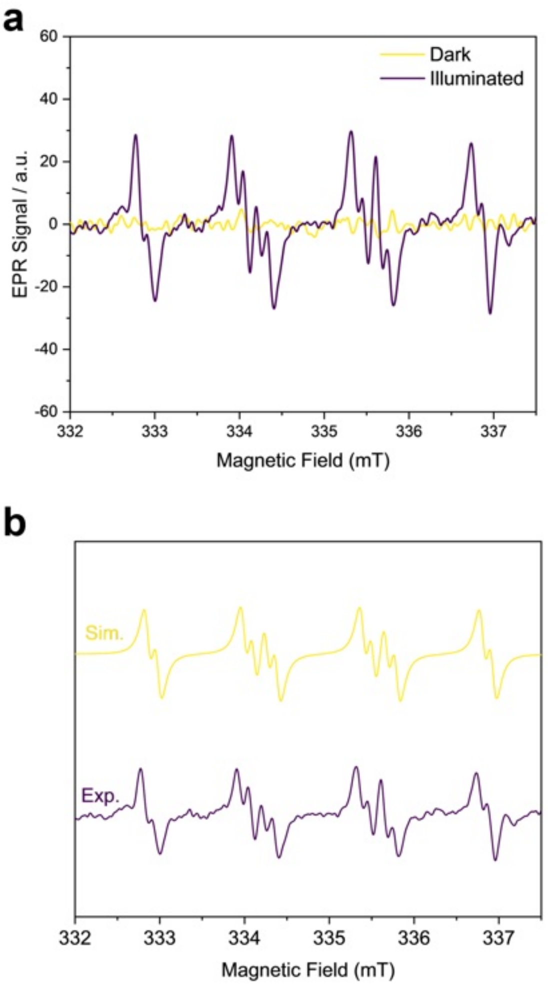

Supplementary Figure 79. a) EPR spectra before (yellow) and after (purple) irradiation of DSAI 0.5 mM with 50 mM DMPO in EDTA (0.1 M, pH = 6.0) under oxygen atmosphere. b) Comparison between experimental and simulated spectra of DMPO-OOH.

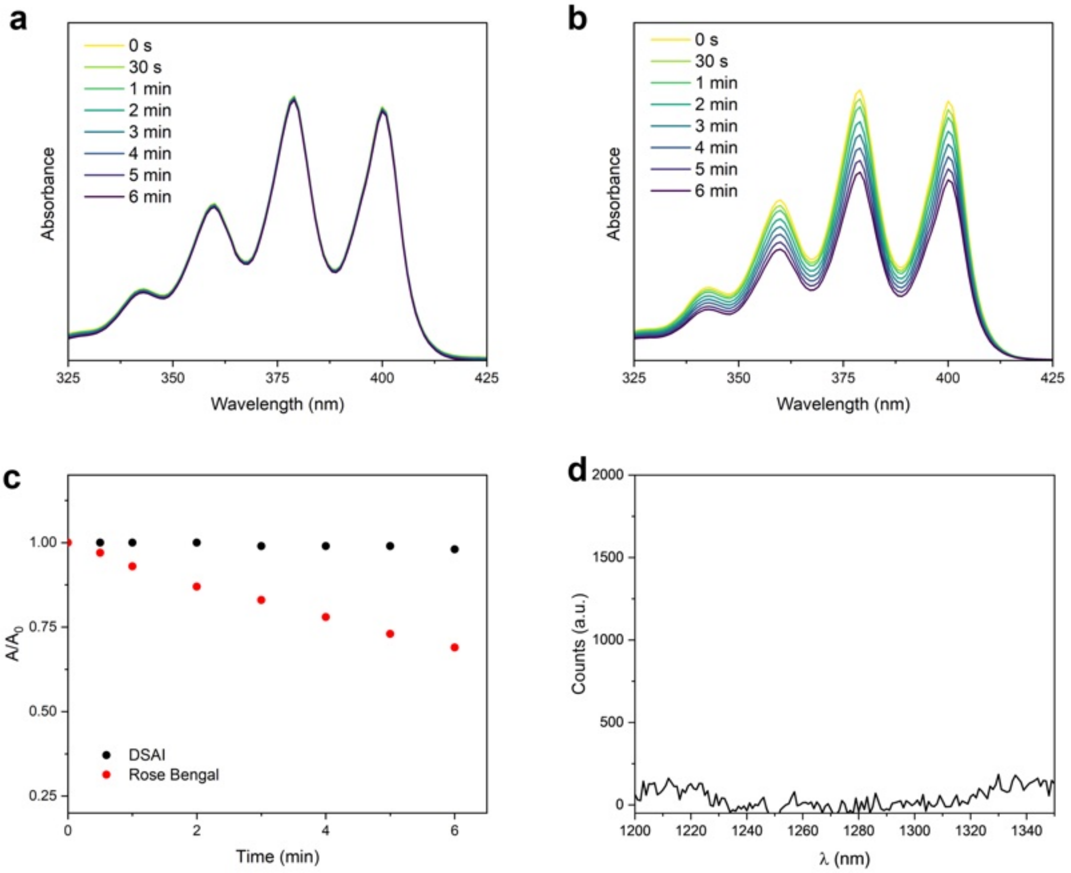

Supplementary Figure 80. Singlet oxygen sensitization evaluated using 9,10-anthracenedipropionic acid (ADPA) as an indicator. a) ADPA UV-Vis spectra evaluated from 0 to 6 minutes irradiation under white light 28 mW cm^{-2} in presence of DSAI 10 µM, NaI 15 mM, ADPA 110 µM; b) ADPA UV-Vis spectra evaluated from 0 to 6 minutes irradiation under white light 28 mW cm^{-2} in presence of Rose Bengal 10 µM and ADPA 110 µM; c) normalized absorption intensity of ADPA at 378 nm after photodecomposition by singlet oxygen upon white light irradiation. d) Singlet oxygen emission spectrum in presence of DSAI 0.1 mM and NaI 5 mM in D_2O.

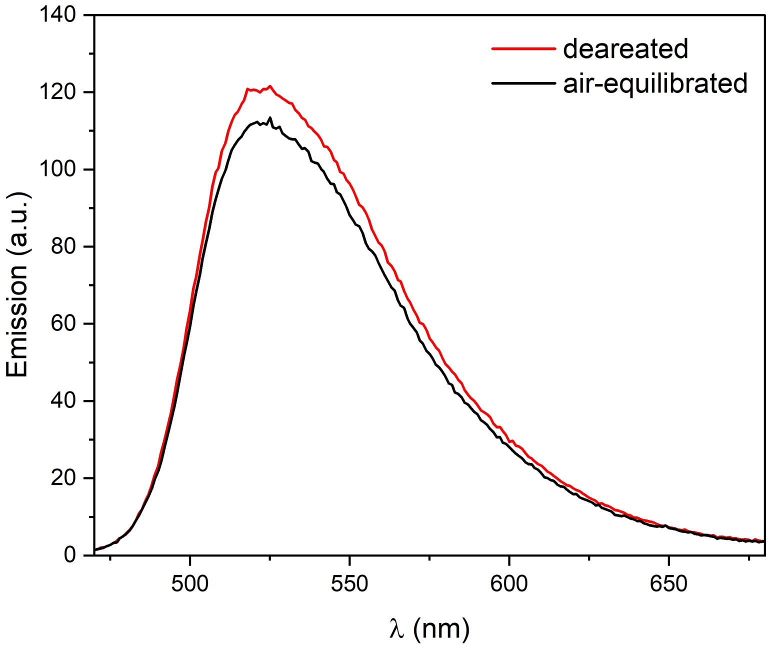

Supplementary Figure 81. Emission spectra of DSAI 0.1 mM with NaI 100 mM under different atmospheres (λ_{exc} = 355 nm, at isoabsorbance).

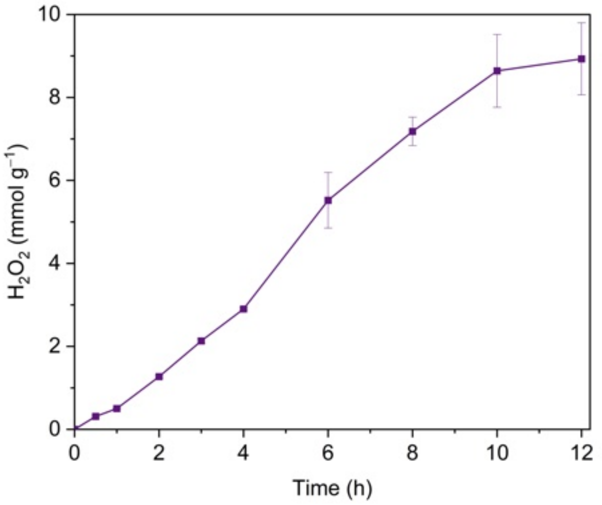

Supplementary Figure 82. Kinetic of H_2O_2 produced by DSACl 0.5 mM, with NaCl 0.9 M in MOPS (0.1 M, pH = 7.0) under 415 nm irradiation (140 mW cm^{-2}) over 12 hours. Statistics are from 3 independent groups.

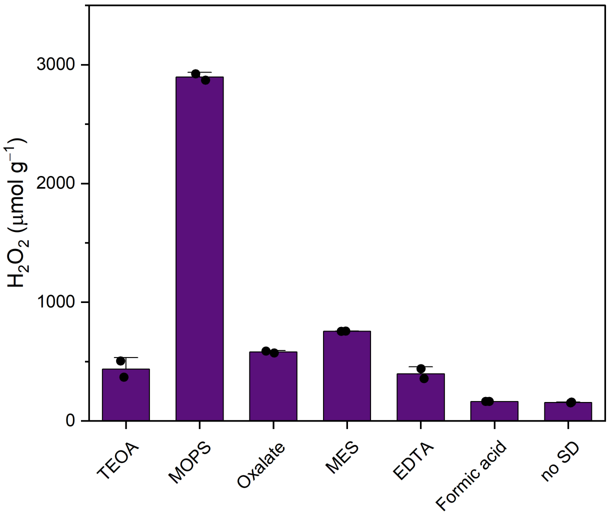

Supplementary Figure 83. H_2O_2 produced by DSACl 0.5 mM, with NaCl 0.9 M in different sacrificial agents under 415 nm irradiation (140 mW cm^{-2}) after 4 hours: 436 ± 97, 2897 ± 39, 580 ± 11, 756 ± 1, 397 ± 58, 163 ± 0 and 155 ± 5 µmol g^{-1}. Statistics are from 2 independent groups. The higher activity observed with MOPS is attributed to more facile aggregation in the presence of this buffer.

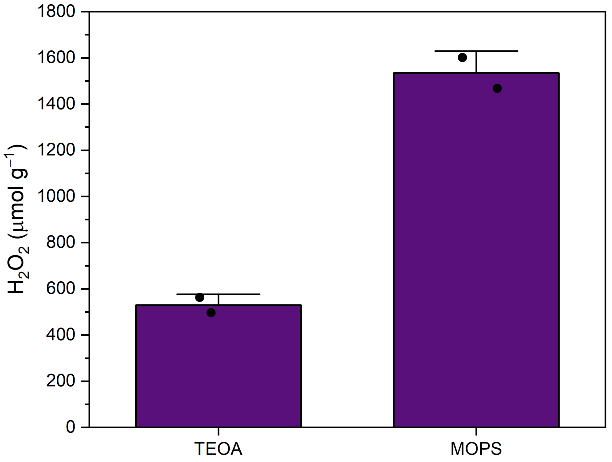

Supplementary Figure 84. H_2O_2 produced by $DSAPF_6$ 0.1 mM in TEOA (530 ± 47 μmol g^{-1}) or MOPS 0.1 M, pH = 7.0 (1535 ± 94 μmol g^{-1}) under white light irradiation (100 mW cm^{-2}). Statistics are from 2 independent groups.

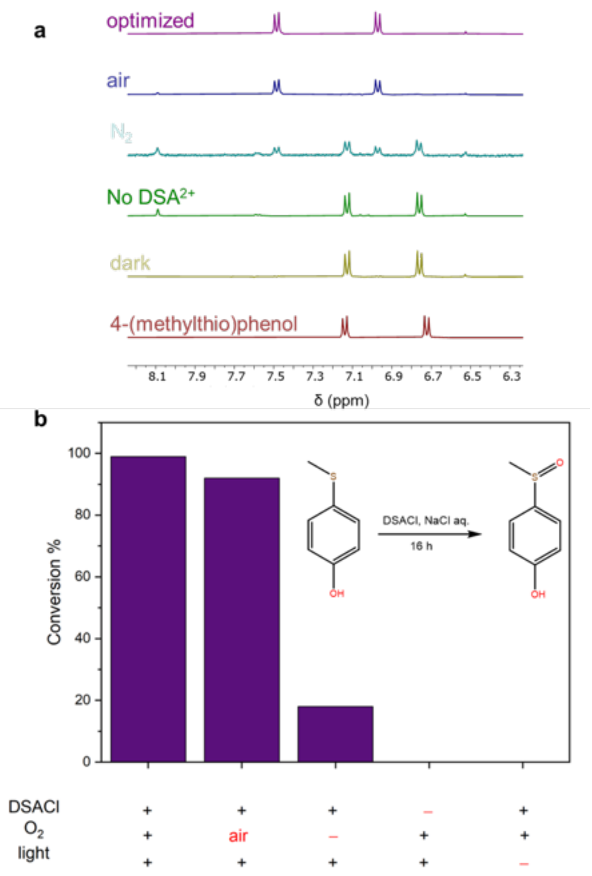

Supplementary Figure 85. Photocatalytic oxidation of 4-(methylthio)phenol to 4-(methylsulfinyl)phenol using DSACl aggregates in water. a) ^{1}H NMR traces, b) calculated conversion. Samples and controls were run with DSACl 1 mM in presence of NaCl 0.9 M in mQ-water, O_2 atmosphere for 16 hours under 415 nm irradiation (140 mW cm^{-2}). Product was quantified using NMR compared to internal standard 1,3,5-trimethoxybenzene in DMSO-d_6.

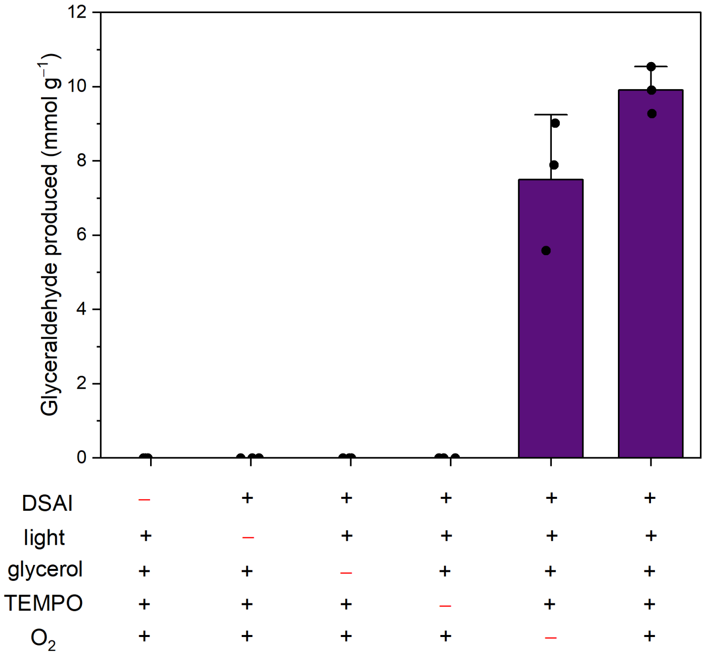

Supplementary Figure 86. Glycerol oxidation to glyceraldehyde (0.0 ± 0.0, 0.0 ± 0.0, 0.0 ± 0.0, 0.0 ± 0.0, 7.5 ± 1.7*,* 9.9 ± 0.9 mmol g^{-1}) performed by DSAI 0.1 mM in presence of NaI 25 mM, glycerol 40 mM and TEMPO 7.5 mM, under white light irradiation (100 mW cm^{-2}) for 16 hours. TEMPO is acting as both a redox-mediator and a catalyst for the oxidative transformation. Statistics are from 3 independent groups.

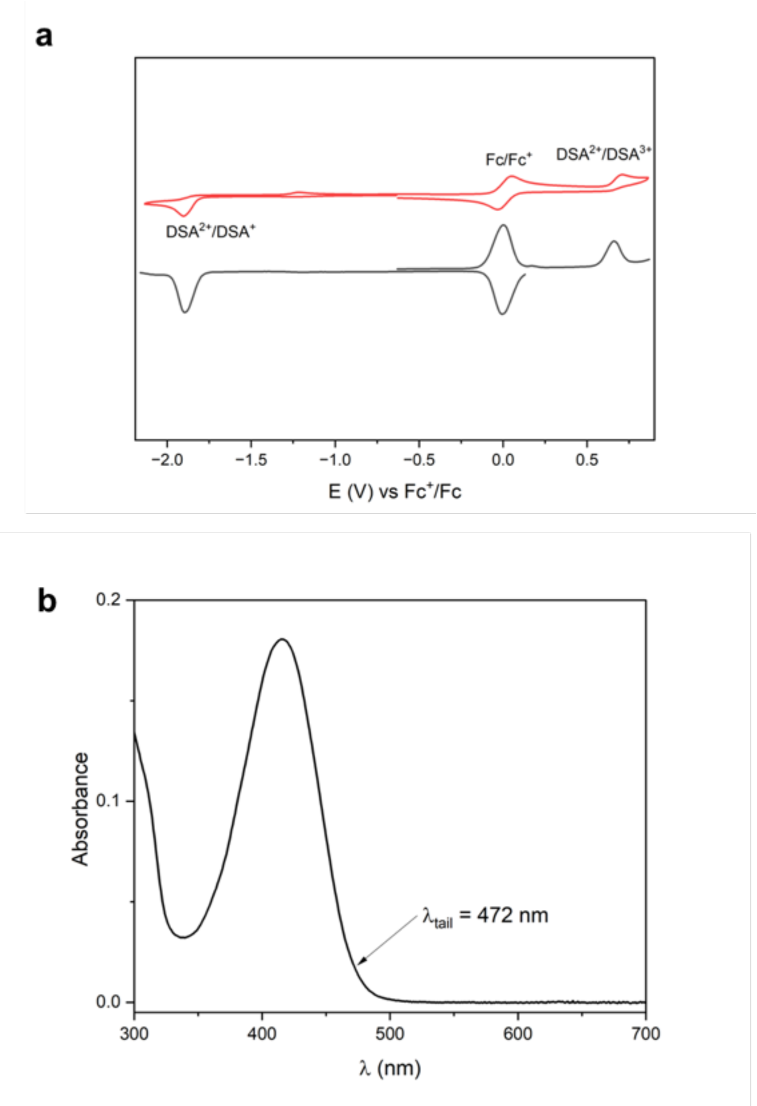

Supplementary Figure 87. a) Cyclic (red trace) and differential pulse voltammetry (black traces) of $DSAPF_6$ 1 mM in DMF, using $TBAPF_6$ 100 mM as electrolyte and Ferrocene 1 mM as internal standard. Scan rate: 100 mV s^{-1}. b) Absorption spectrum of $DSAPF_6$ 10 μM in DMF and the calculated long wavelength tail value.

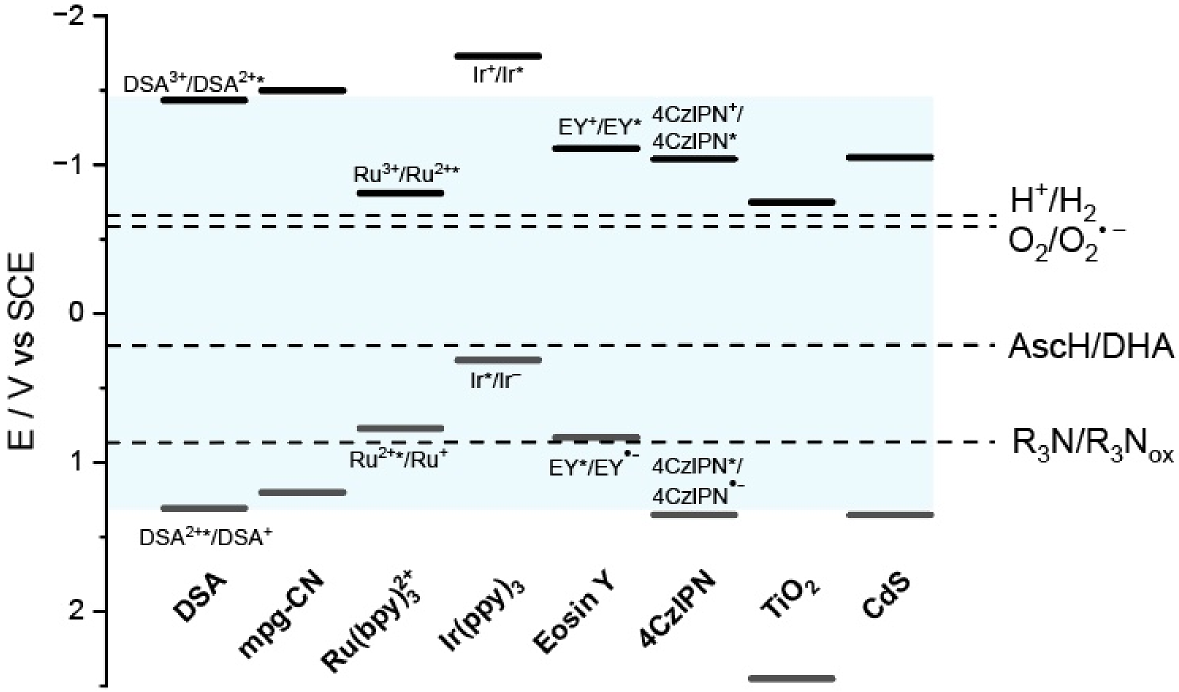

Supplementary Figure 88. Calculated reduction and oxidation excited state potentials for DSA^{2+} vs SCE, compared to thermodynamic potentials for H^+/H_2, $O_2/O_2^{\cdot -}$, ascorbic acid and amines and relative positions of excited state potentials of DSA^{2+} compared with commonly used photocatalysts. The blue background serves as guide for the eyes from comparing the excited state potential with other common photocatalysts. Scheme adapted from reference.[40]

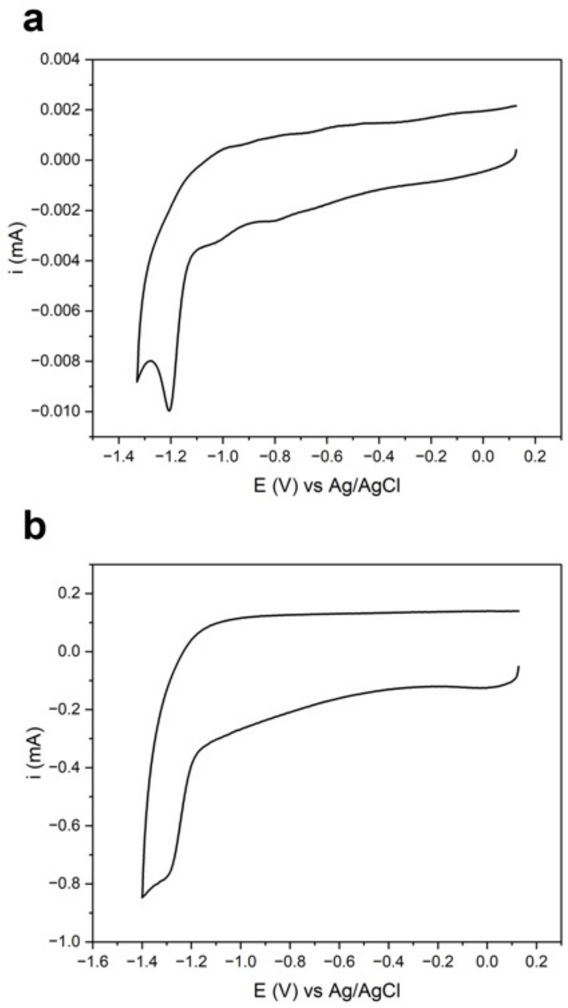

Supplementary Figure 89. Cyclic voltammetries of a) DSAI 0.25 mM in aqueous NaOAc 1 M and b) DSAI deposited as ink (with Vulcan C) in H_2O (KCl 1 M); scan rate 100 mV/s.

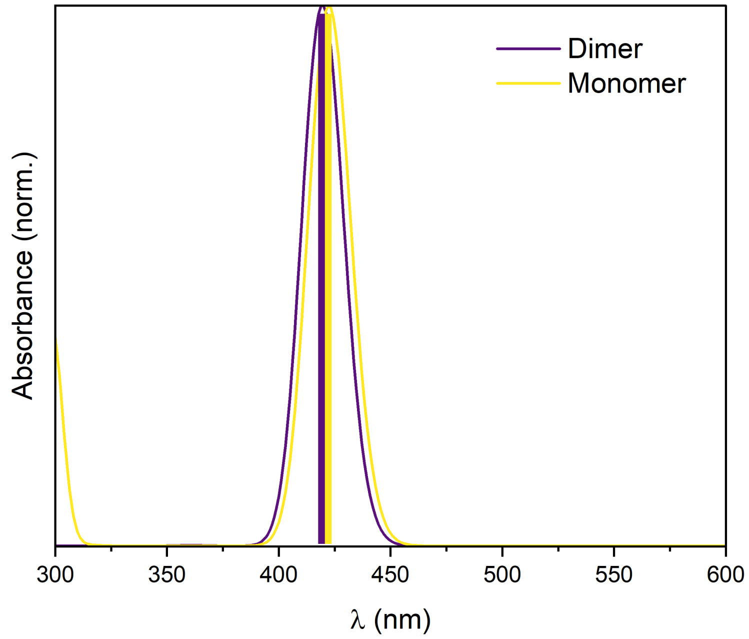

Supplementary Figure 90. Simulated UV-Vis (TD-DFT) spectra of DSAI as monomer (yellow trace) and dimer (purple trace). Calculations performed at the CAM-B3LYP/6-31G*/LanL2DZ level of theory.

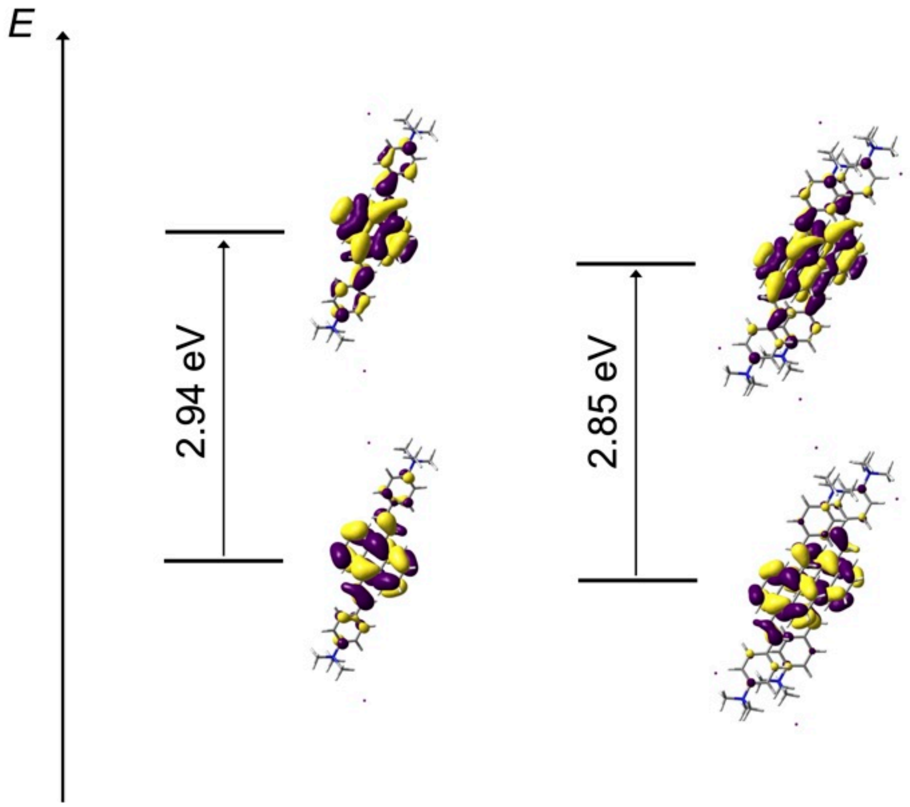

Supplementary Figure 91. Frontier molecular orbitals for DSAI as monomer and as dimer and HOMO-LUMO transition, calculated with TD-DFT.

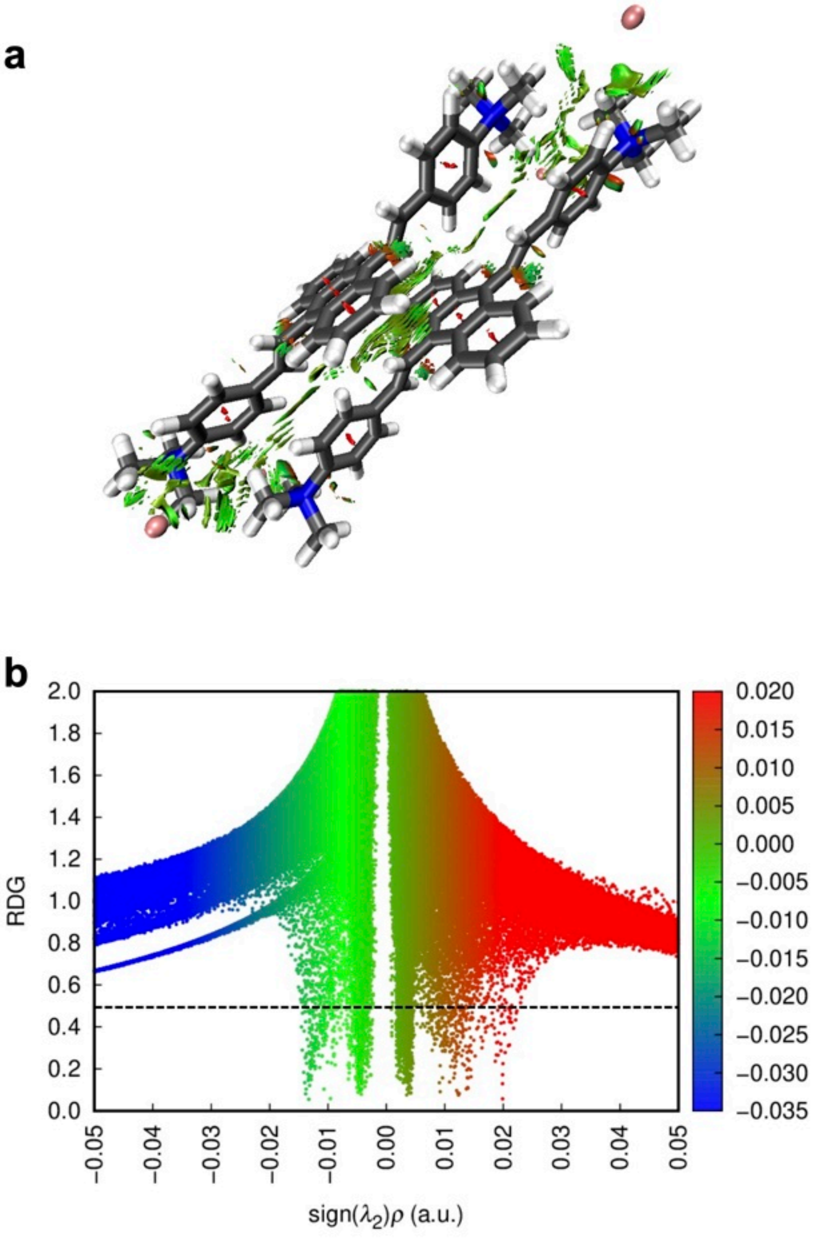

Supplementary Figure 92. a) Non-covalent interaction (NCI)-plot visualizing interactions as green surfaces; b) RDG scatter plot. Colors: blue represents strong attractive interactions, green indicates van der Waals interactions and red indicates repulsive/steric interactions.

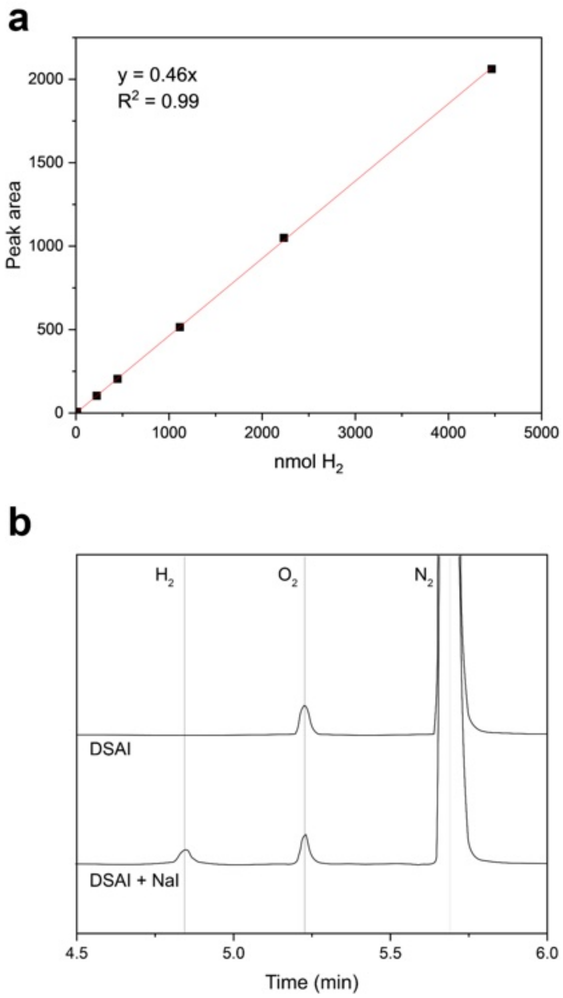

Supplementary Figure 93. a) Calibration curve for H_2 quantification with gas chromatography; b) chromatograms acquired after 4 hours irradiation of DSAI 0.1 mM in ascorbic acid (1.0 M, pH = 4.0) with PtNPs (8% mol) with (bottom) and without (top) NaI as aggregating agent

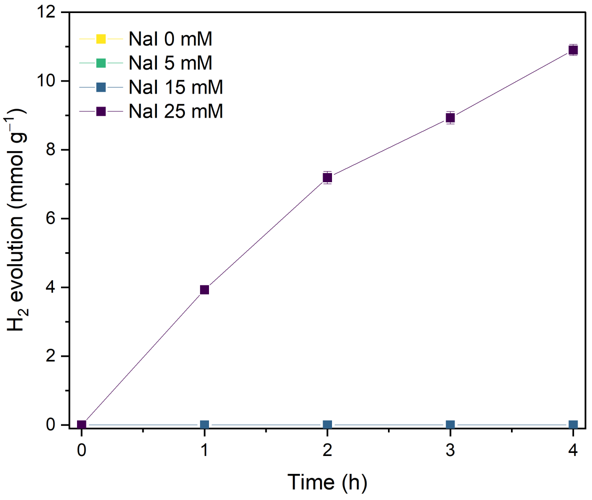

Supplementary Figure 94. Kinetic study of DSAI (0.1 mM) photosensitized H_2 evolution over 4 hours irradiation under white light (100 mW cm^{-2}), in ascorbic acid (1.0 M, pH = 4.0), PtNPs (8% mol) and different NaI concentrations. Statistics are from 3 independent groups.

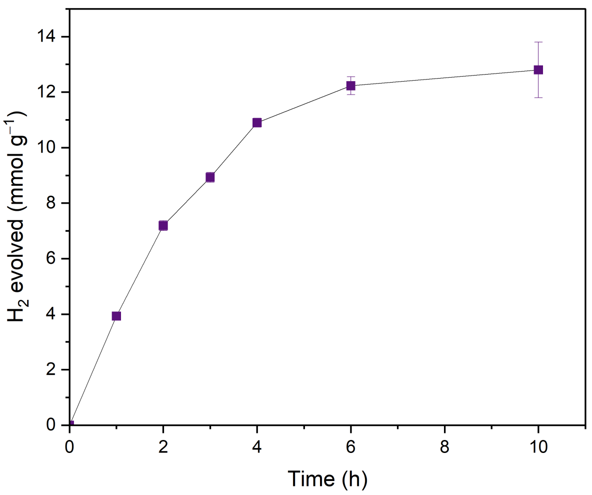

Supplementary Figure 95. Kinetic study of DSAI (0.1 mM) photosensitized H_2 evolution over 10 hours irradiation under white light (100 mW cm^{-2}), in ascorbic acid (1.0 M, pH = 4.0), PtNPs (8% mol), NaI 25 mM. Statistics are from 3 independent groups.

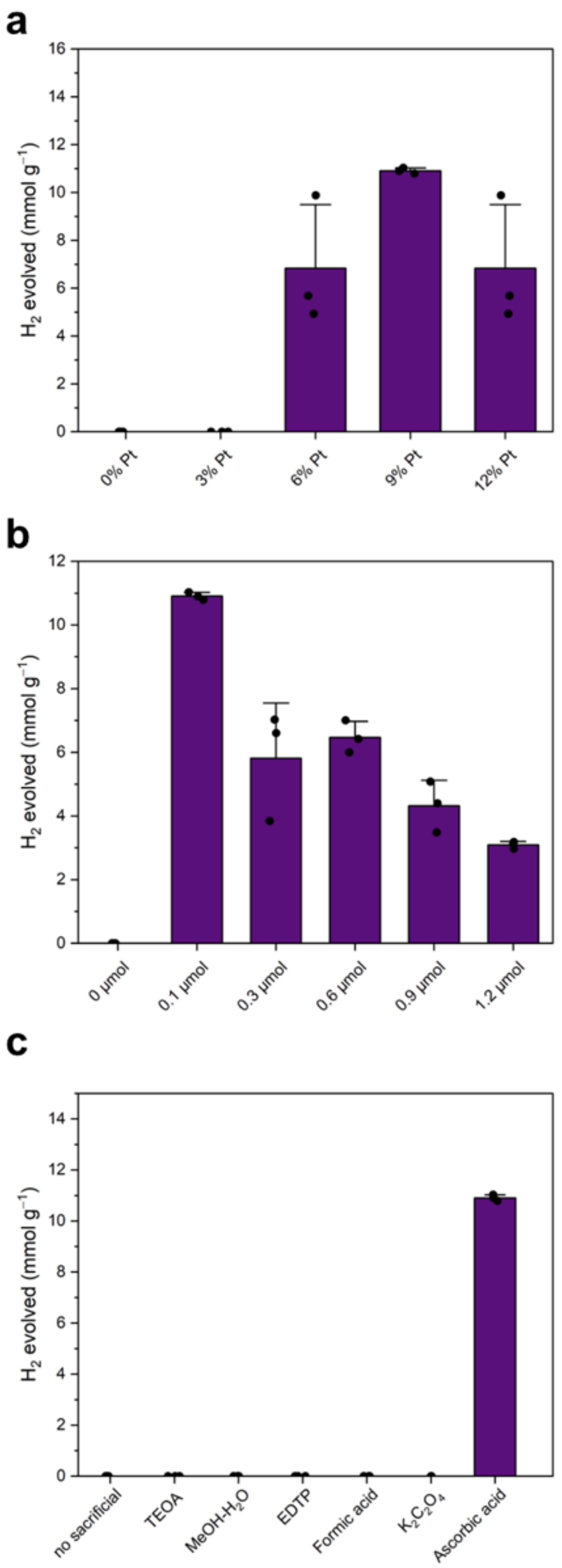

Supplementary Figure 96. a) Optimization of PtNPs molar ratio for DSAI photosensitized H_2 evolution (DSAI 0.1 mM in ascorbic acid 1.0 M, pH = 4.0, 4 hours under white light 100 mW cm^{-2}): 0.00 ± 0.00, 0.00 ± 0.00, 6.83 ± 2.67, 10.9 ± 0.1, 6.36 ± 3.25 mmol g^{-1}; b) optimization of DSAI concentration for H_2 evolution (ascorbic acid 1.0 M, pH = 4.0, PtNPs 9% mol, 4 hours under white light 100 mW cm^{-2}): 0.00 ± 0.00, 10.9 ± 0.1, 5.82 ± 1.72, 6.40 ± 1.0, 4.32 ± 0.80, 3.09 ± 0.11 mmol g^{-1}; c) different sacrificial agent tested for DSAI photosensitized H_2 evolution (DSAI 0.1 mM, PtNPs 9% mol, 4 hours under white light 100 mW cm^{-2}): 0.00 ± 0.00, 0.00 ± 0.00, 0.00 ± 0.00, 0.00 ± 0.00, 0.00 ± 0.00, 0.00 ± 0.00, 10.9 ± 0.1 mmol g^{-1}. Statistics are from 3 independent groups.

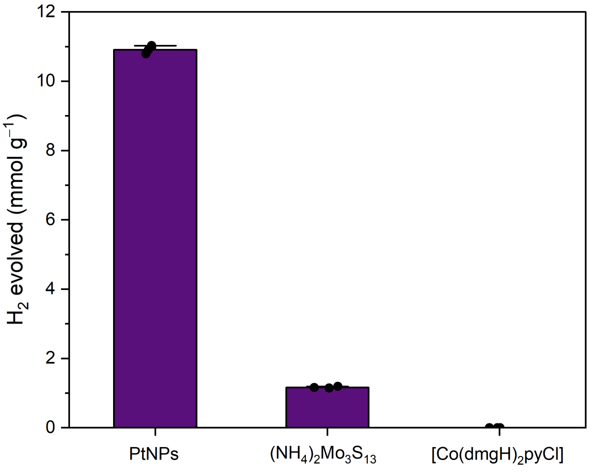

Supplementary Figure 97. Photosensitized H_2 evolution (DSAI 0.1 mM in ascorbic acid 1.0 M, pH = 4.0, NaI 25 mM), 4 hours under white light (100 mW cm^{-2}) with different co-catalyst 8% mol (PtNPs, $(NH_4)_2Mo_3S_{13}$, [Co(dmgH)$_2$pyCl]). H_2 evolved (mmol g^{-1}) is 10.9 ± 0.1 with PtNPs, 1.17 ± 0.03 with $(NH_4)_2Mo_3S_{13}$ and 0.00 ± 0.00 with [Co(dmgH)$_2$pyCl]. Statistics are from 3 independent groups.

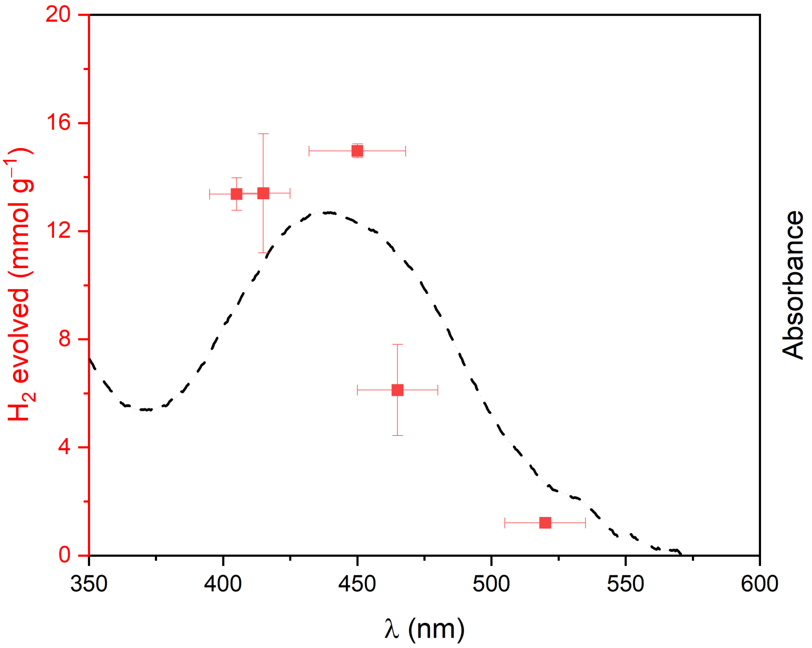

Supplementary Figure 98. H_2 evolution produced by DSAI 0.1 mM in ascorbic acid (1.0 M, pH = 4.0) in presence of NaI 25 mM and PtNPs (8% mol) under 4 hours irradiation at different wavelengths at 26 mW cm^{-2} and DSAI absorption spectrum in the same conditions. Statistics are from 2 independent groups.

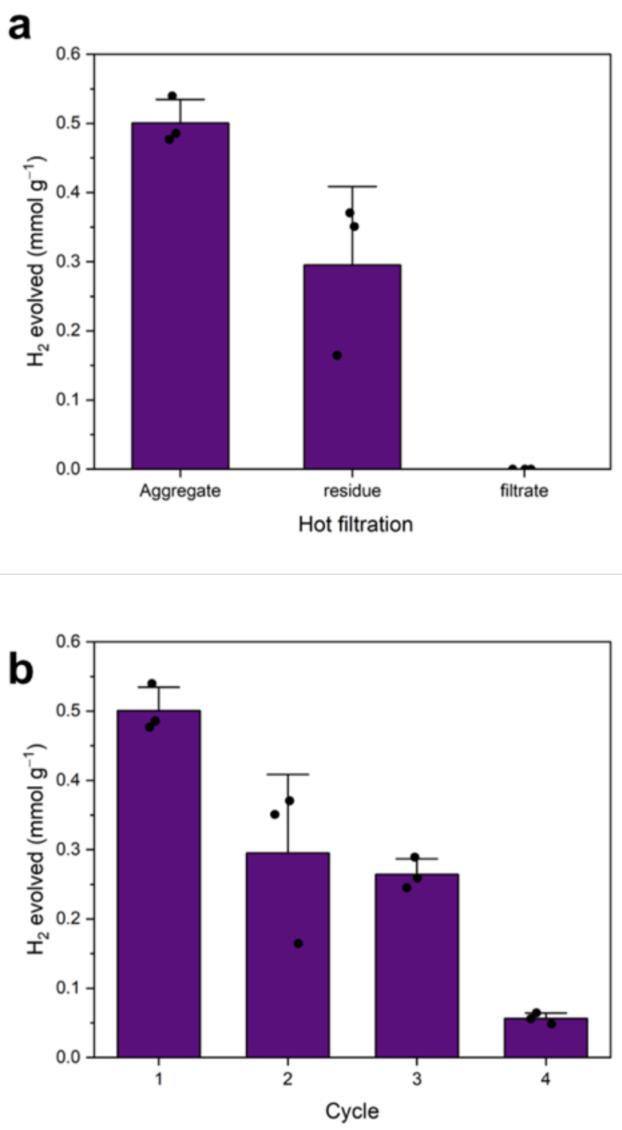

Supplementary Figure 99. Recycling tests for H_2 evolution: DSAI 0.65 mM, ascorbic acid 1.0 M, pH = 4.0, PtNPs 8% mol, NaI 16.25 mM, white light 100 mW cm^{-2} for 4 hours each cycle; samples were filtered using a polycarbonate membrane filter (0.1 μm pore size) after each cycle. Fresh ascorbic acid and PtNPs were added in each cycle. a) Cycle 1 compared to the catalytic activity of the residue and the filtrate (0.50 ± 0.03, 0.30 ± 0.11, 0.00 ± 0.00 mmol g^{-1}); b) different activities of the four catalytic cycles (0.50 ± 0.03, 0.30 ± 0.11, 0.26 ± 0.02, 0.06 ± 0.01 mmol g^{-1}). Statistics are from 3 independent groups.

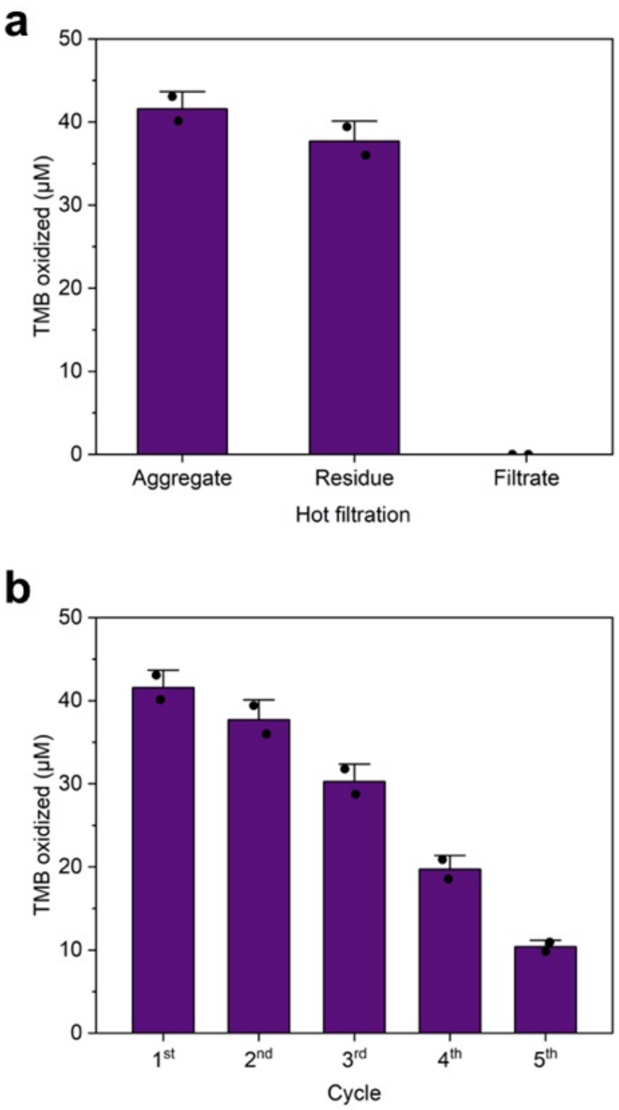

Supplementary Figure 100. a) Hot filtration (42 ± 2, 38 ± 2, 0.0 ± 0.0 μM) and b) recycling tests (42 ± 2, 38 ± 2, 30 ± 2, 20 ± 2, 10 ± 1 μM) for light-driven TMB oxidation using $DSAPF_6$ (0.24 mg mL^{-1}) nanoprecipitated from DMSO in presence of TMB (134 μM) in acetate buffer (0.1 M, pH = 5) under white light (180 mW cm^{-2}) under air. 15 min each cycle; samples were filtered using a polycarbonate membrane filter (0.1 μm pore size). After each cycle. fresh acetate buffer and TMB were added. Statistics are from 2 independent groups.

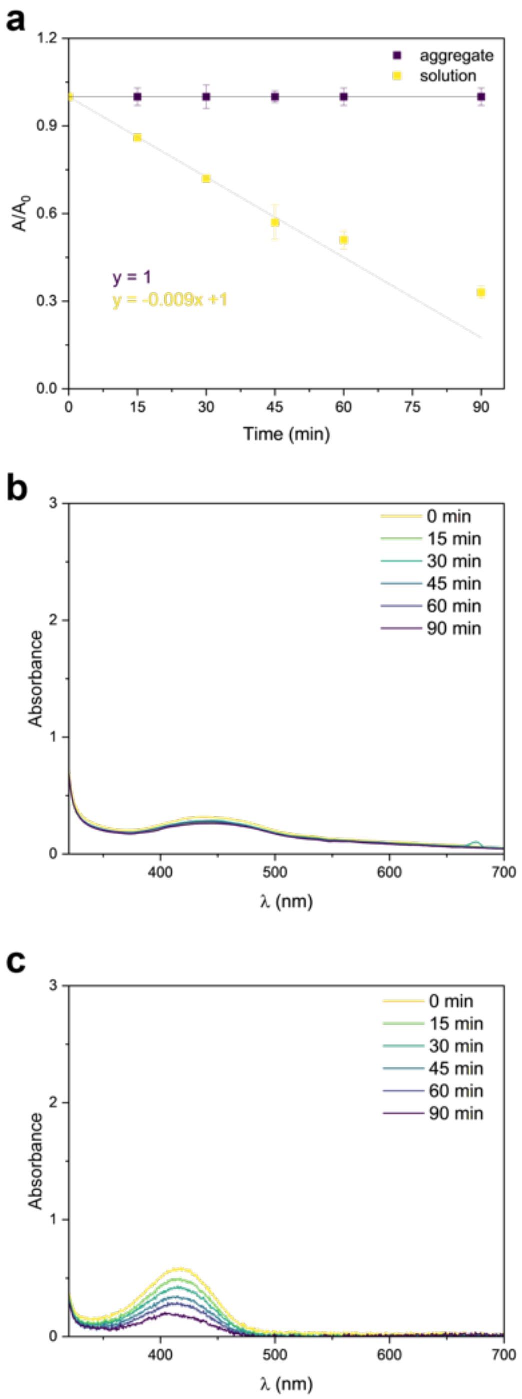

Supplementary Figure 101. a) Absorbance over time by irradiation (white light, 100 mW cm^{-2}) of DSAI solution or DSAI nanostructures in ascorbic acid; b) absorption spectra variation of DSAI nanostructure in ascorbic acid, and c) absorption spectra variation of DSAI solution in ascorbic acid. Statistics are from 2 independent groups.

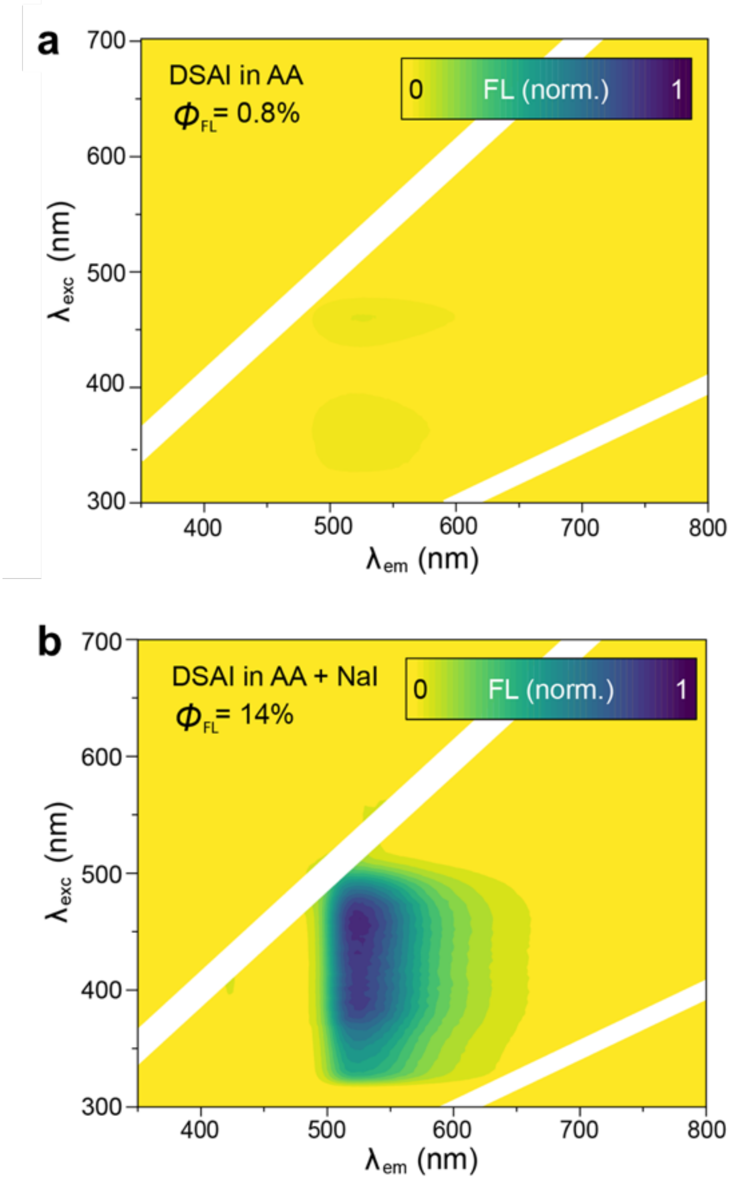

Supplementary Figure 102. a) excitation-emission map (EEM) and fluorescence quantum yield for DSAI solution 0.1 mM in ascorbic acid (1.0 M, pH = 4.0); b) excitation-emission map (EEM) and fluorescence quantum yield for DSAI nanostructures (DSAI 0.1 mM, NaI 25 mM) in ascorbic acid (1.0 M, pH = 4.0).

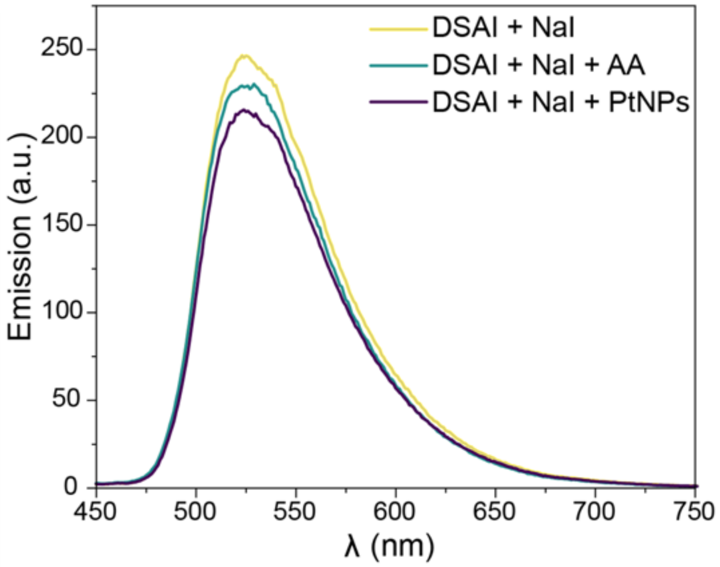

Supplementary Figure 103. Fluorescence spectra (λ_{exc} = 395 nm, at isoabsorbance) of DSAI nanostructures (0.1 mM, NaI 25 mM) in absence or presence of sacrificial agent (AA; 2 mM) and co-catalyst (PtNPs, 8% mol).

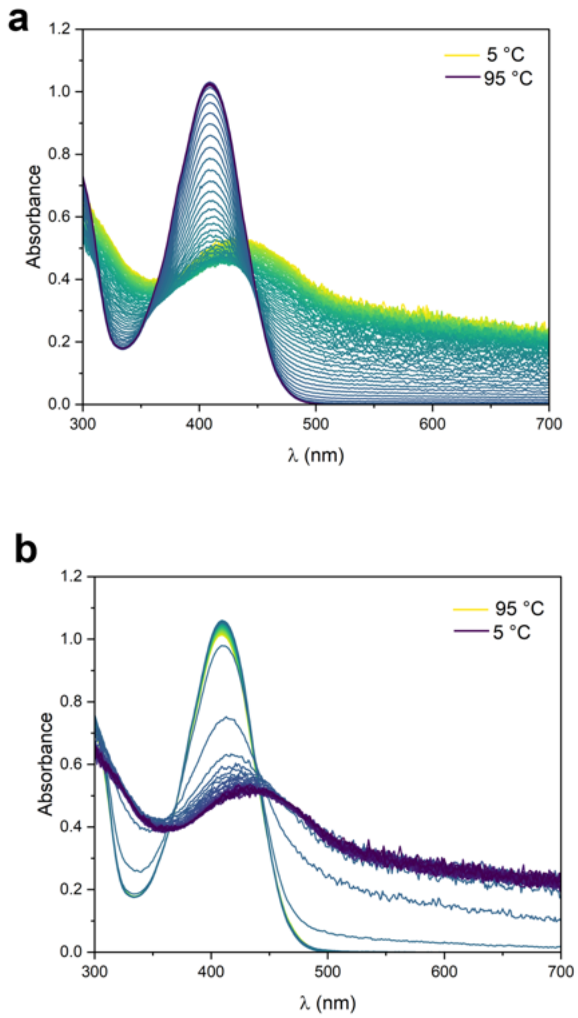

Supplementary Figure 104. a) Absorption spectra of heating from 5 °C to 95 °C and b) cooling from 95 °C to 5 °C with a rate of 1.0 K min^{-1} of DSAI 50 μM with NaI 5.0 mM in mQ-water.

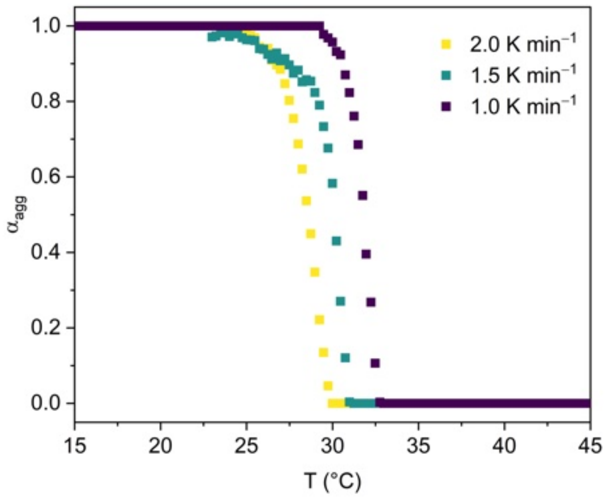

Supplementary Figure 105. Aggregation degree *vs* temperature: cooling ramp from 95 °C to 5 °C for DSAI 50 µM, with NaI 5.0 mM in mQ-water acquired with different scan rates.

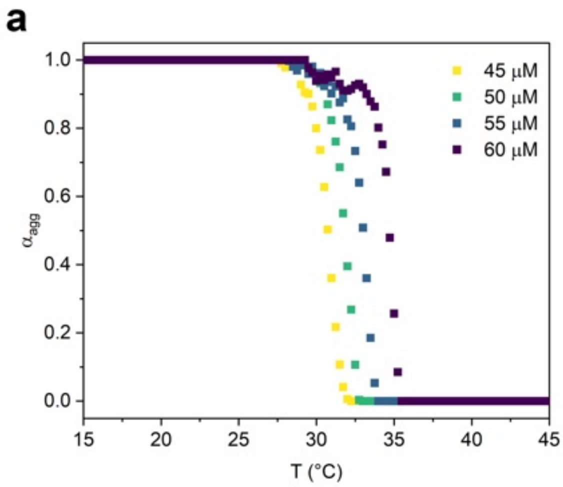

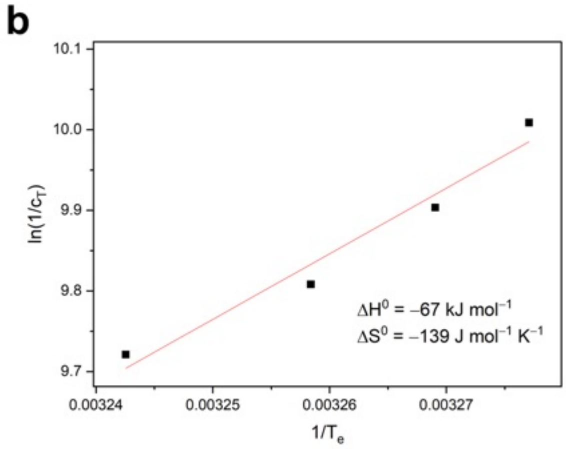

Supplementary Figure 106. a) Aggregation degree *vs* temperature: cooling ramp from 95 °C to 5 °C for different concentrations of DSAI with NaI 5.0 mM in mQ-water acquired with a rate of 1.0 K min^{-1}. b) Van't Hoff fitting of the supramolecular aggregation of DSAI.

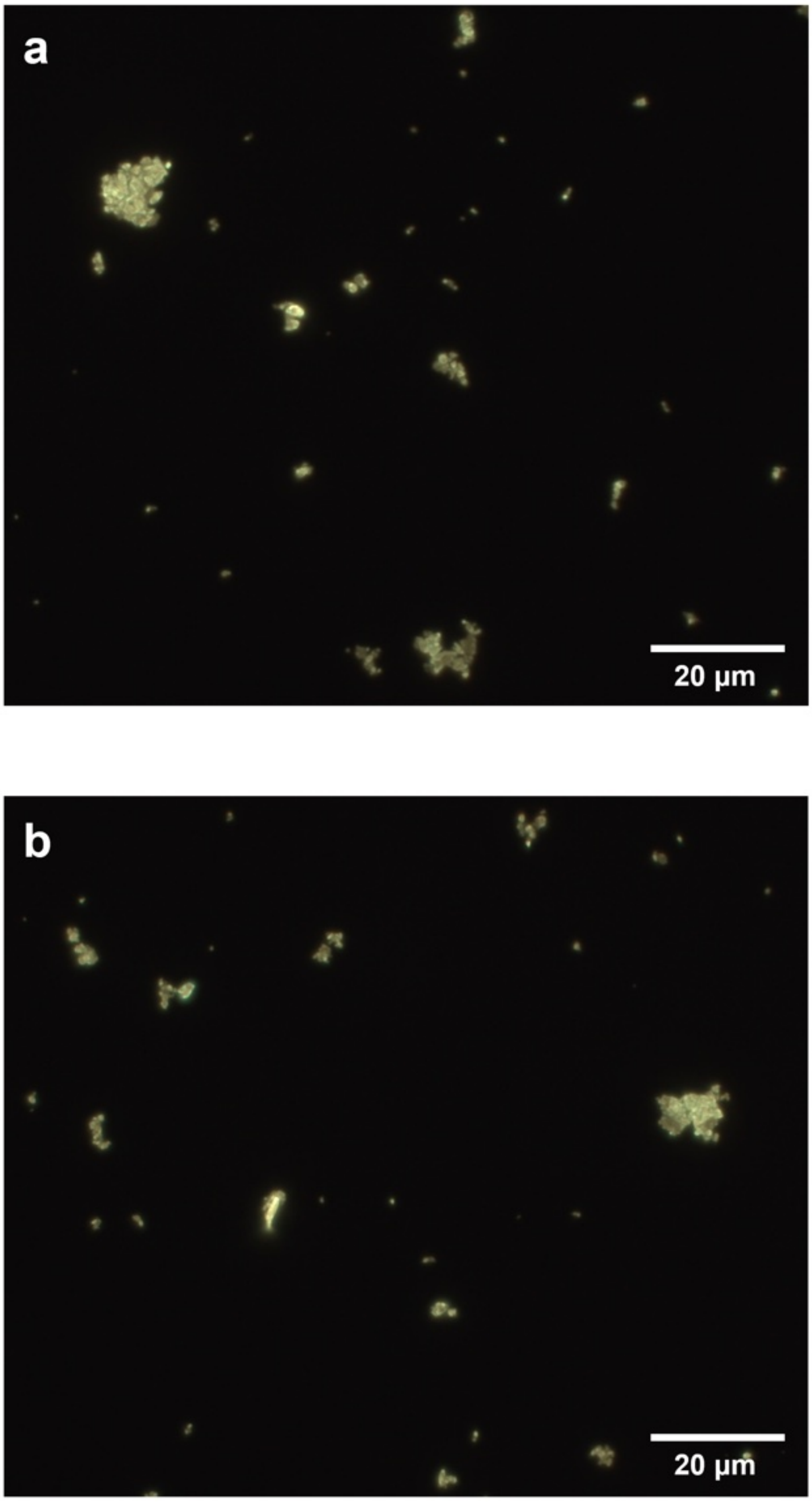

Supplementary Figure 107. a,b) Fluorescence microscopy images of the kinetically trapped aggregate (DSAI 50 µM, NaI 5.0 mM). The kinetic aggregates are visible due to their characteristic fluorescence emission.

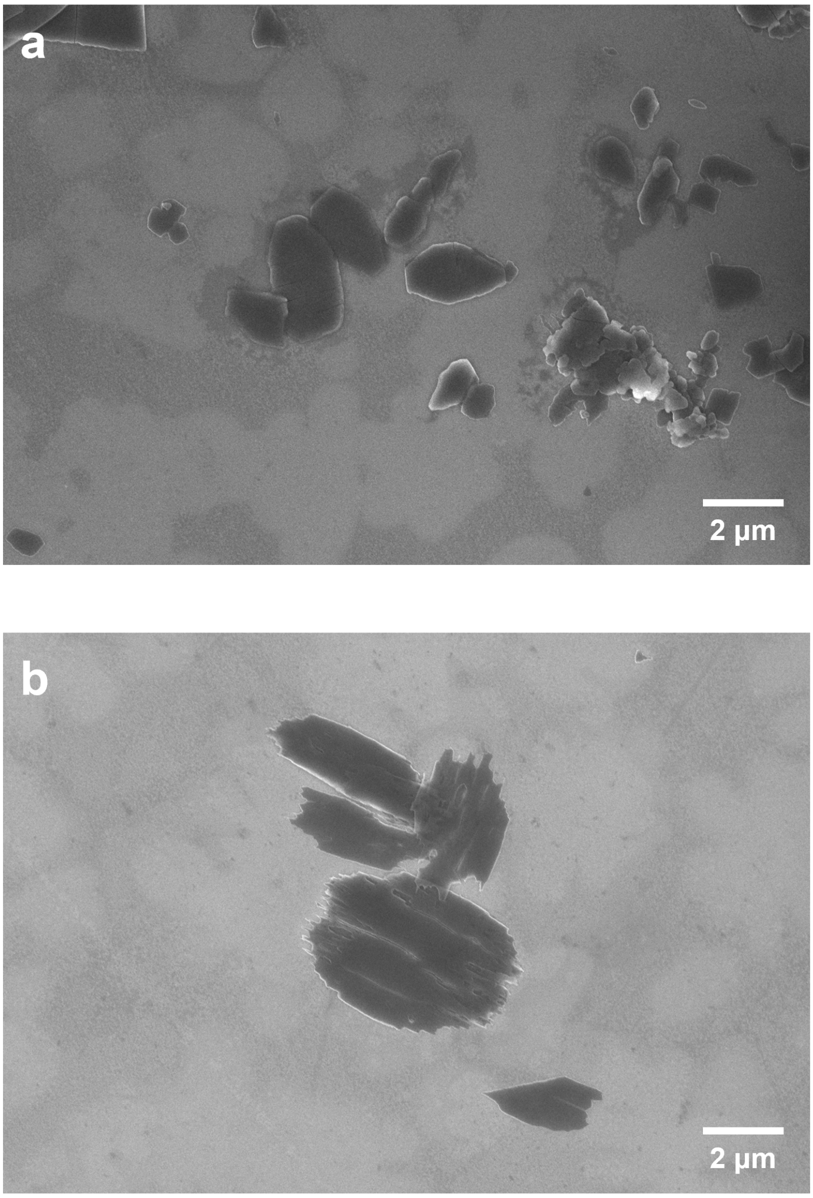

Supplementary Figure 108. a,b) SEM images of the kinetically trapped aggregate (DSAI 50 μM, NaI 5.0 mM).

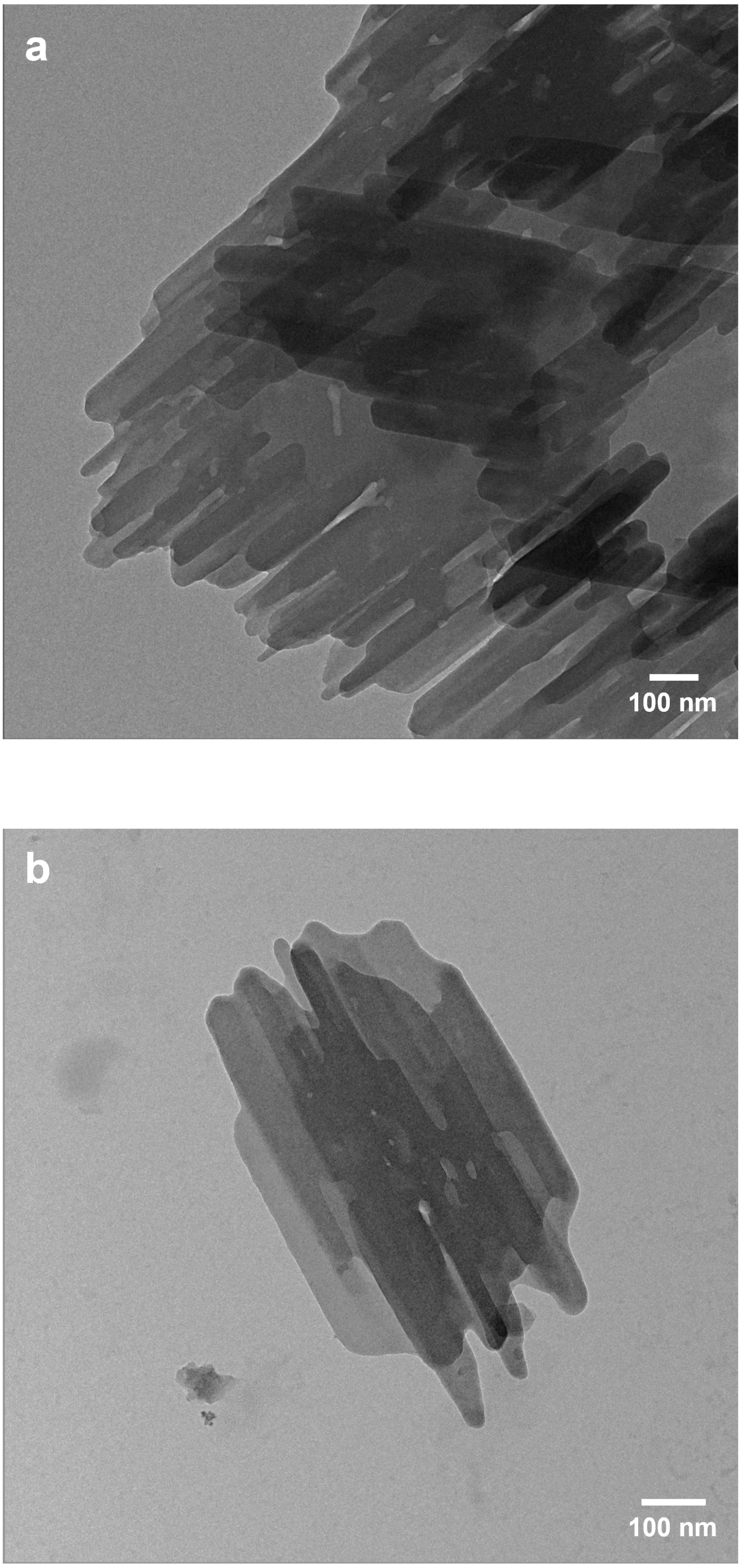

Supplementary Figure 109. a,b) TEM micrographs of the kinetically trapped aggregate (DSAI 50 µM, NaI 5.0 mM).

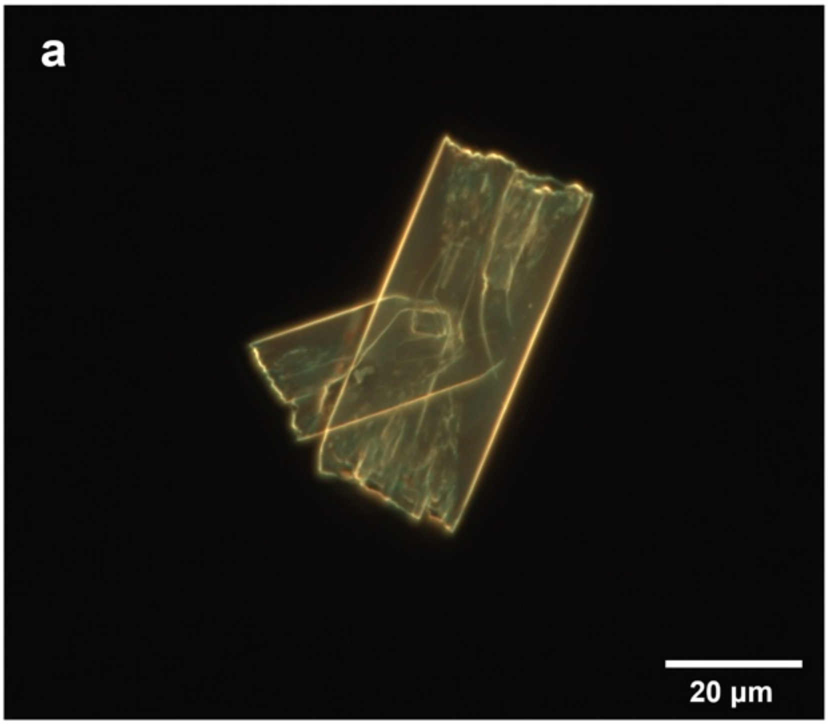

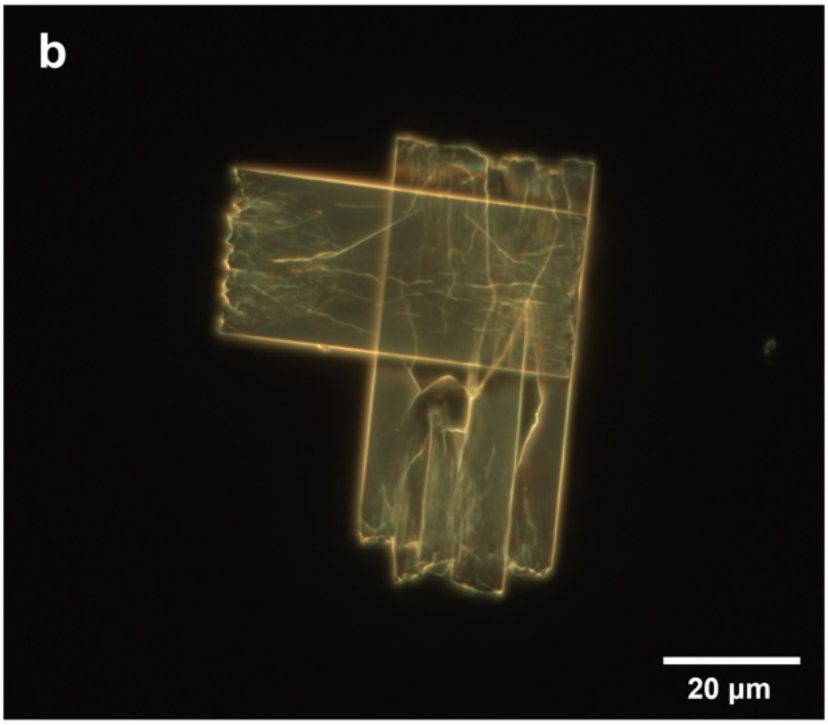

Supplementary Figure 110. a,b) Fluorescence microscopy images of the thermodynamic aggregates (DSAI 50 µM, NaI 5.0 mM).

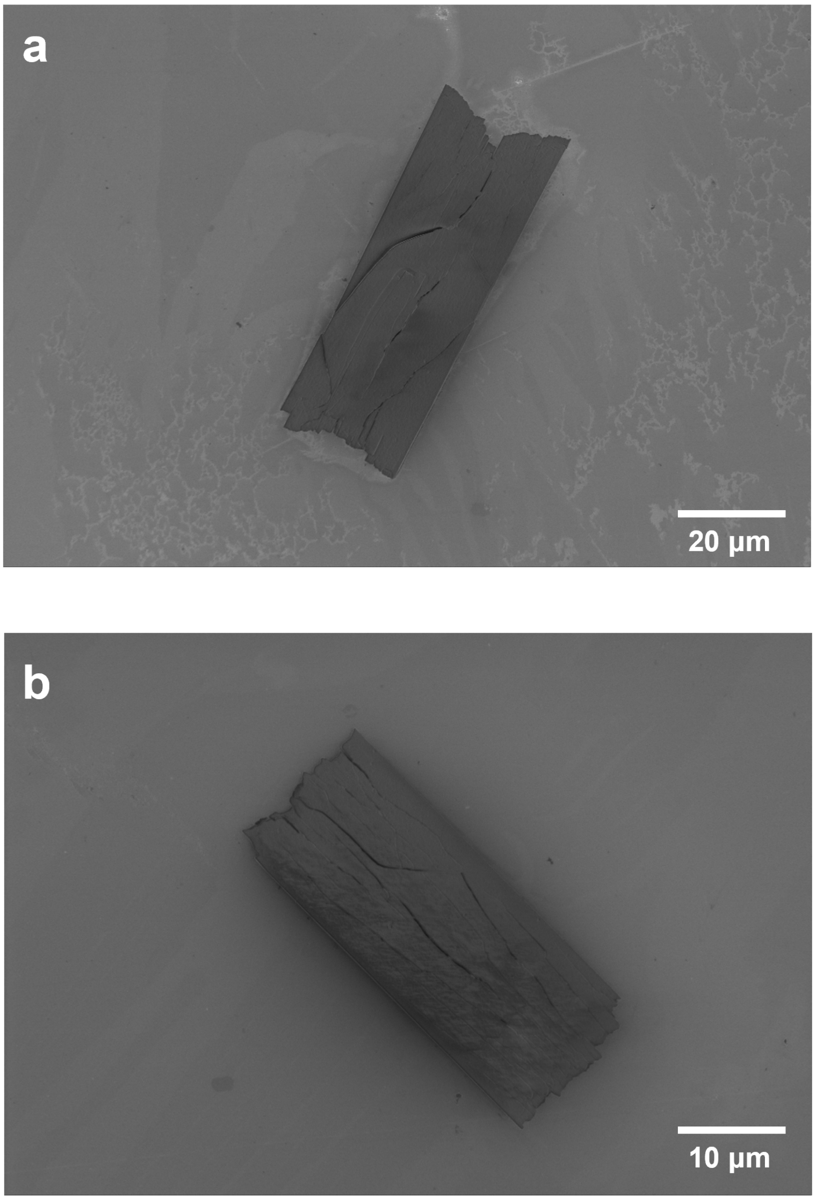

Supplementary Figure 111. a,b) SEM images of the thermodynamic aggregates (DSAI 50 µM, NaI 5.0 mM).

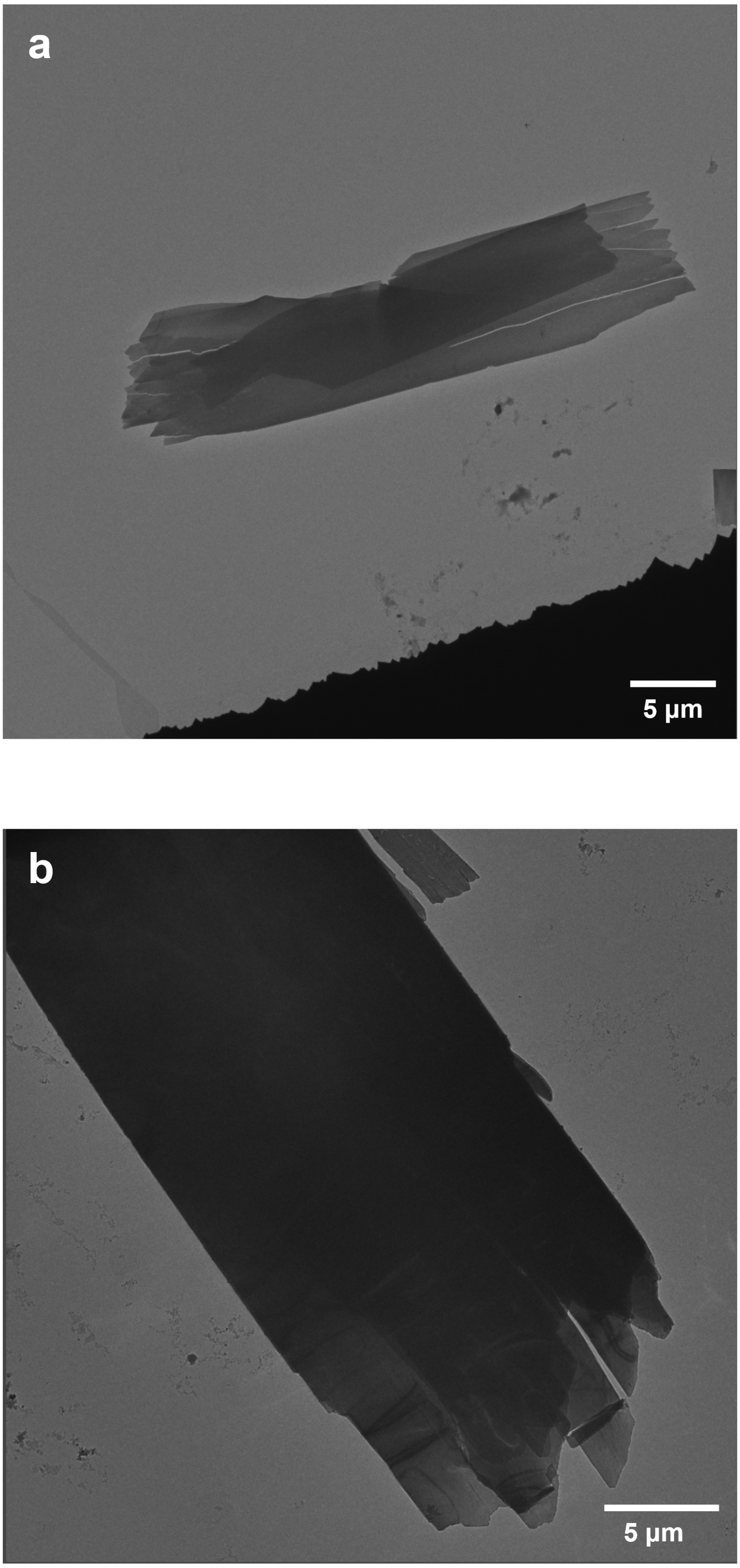

Supplementary Figure 112. a,b) TEM micrographs of the thermodynamic aggregates (DSAI 50 μM, NaI 5.0 mM).

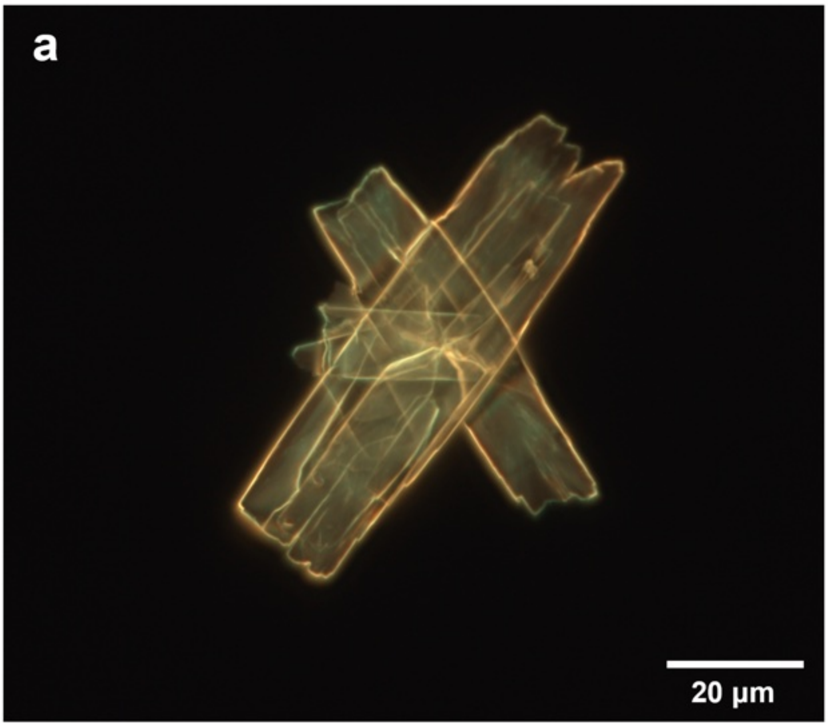

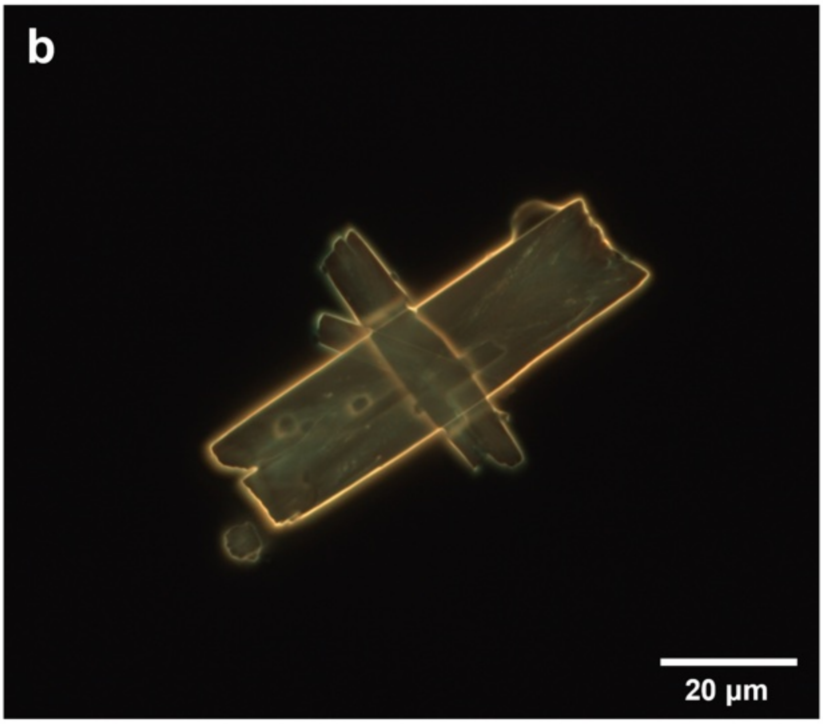

Supplementary Figure 113. a,b) Fluorescence microscopy images of the seeded-growth aggregates (DSAI 50 µM, NaI 5.0 mM).

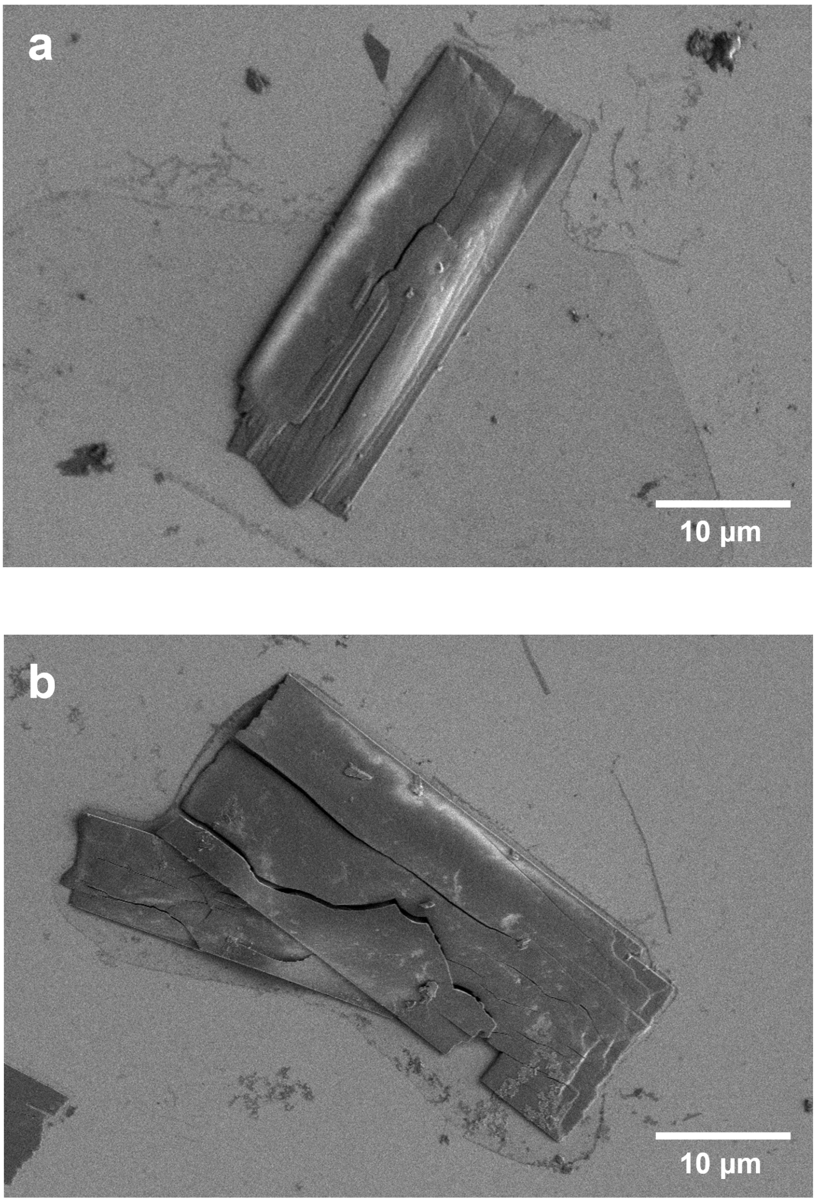

Supplementary Figure 114. a,b) SEM images of the seeded-growth aggregates (DSAI 50 µM, NaI 5.0 mM).

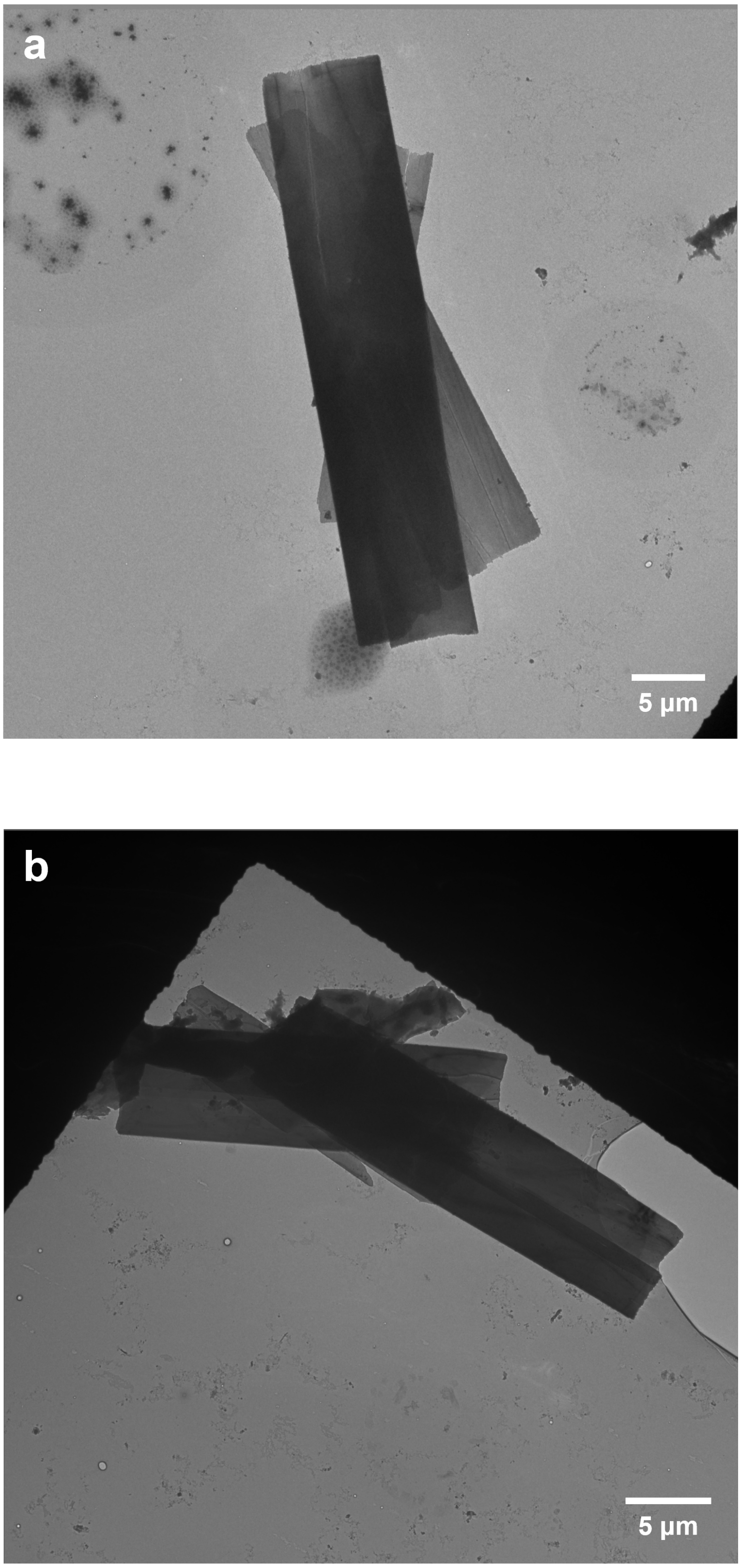

Supplementary Figure 115. a,b) TEM micrographs of the seeded-growth aggregates (DSAI 50 µM, NaI 5.0 mM).

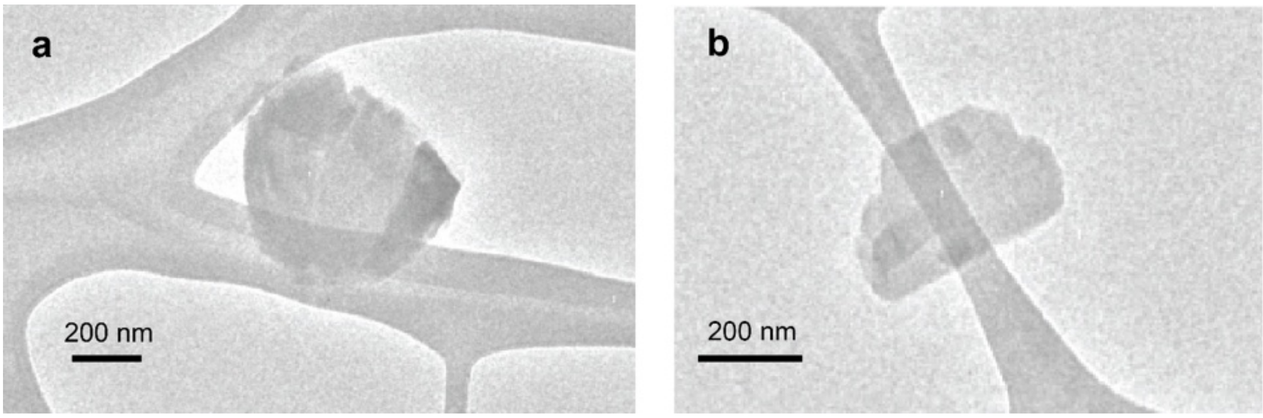

Supplementary Figure 116. Representative nanocrystals of a) kinetic aggregates and b) thermodynamic aggregates of DSAI used for 3D ED experiments.

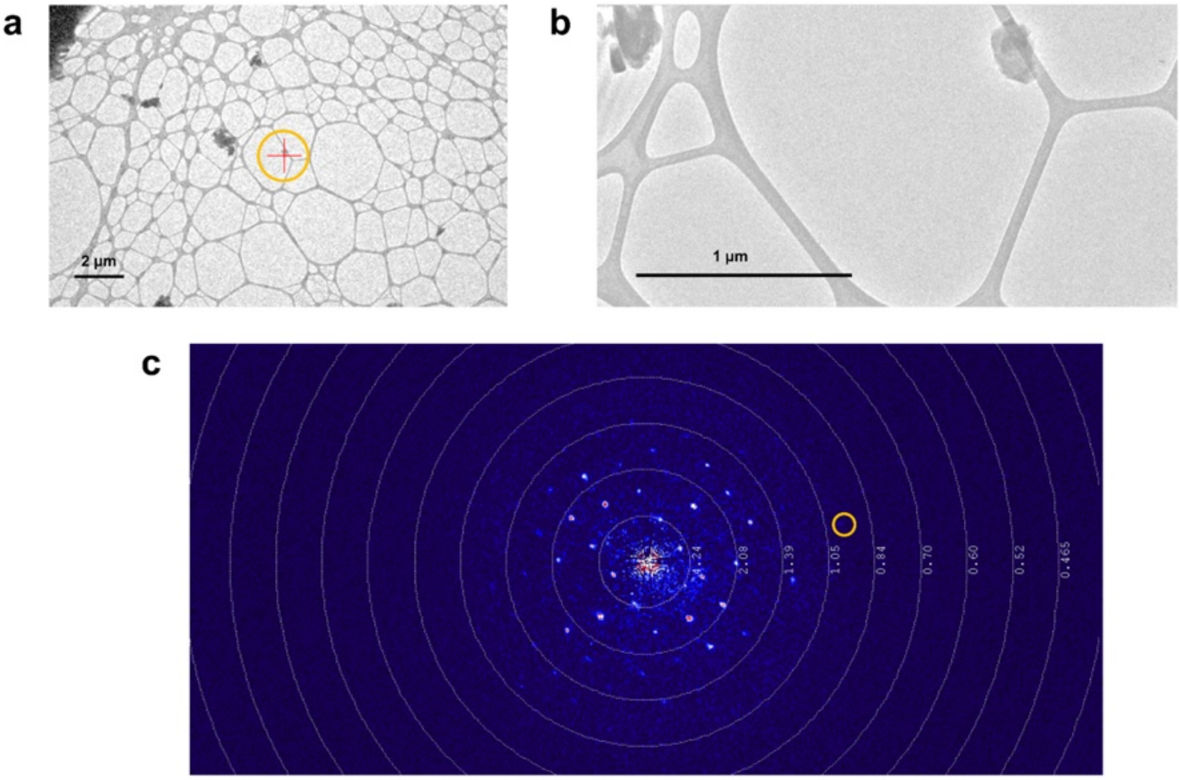

Supplementary Figure 117. a,b) Grain snapshot of a kinetic aggregate DSAI nanocrystal. c) Diffraction pattern showing diffraction up to approximately 0.90 Å resolution.

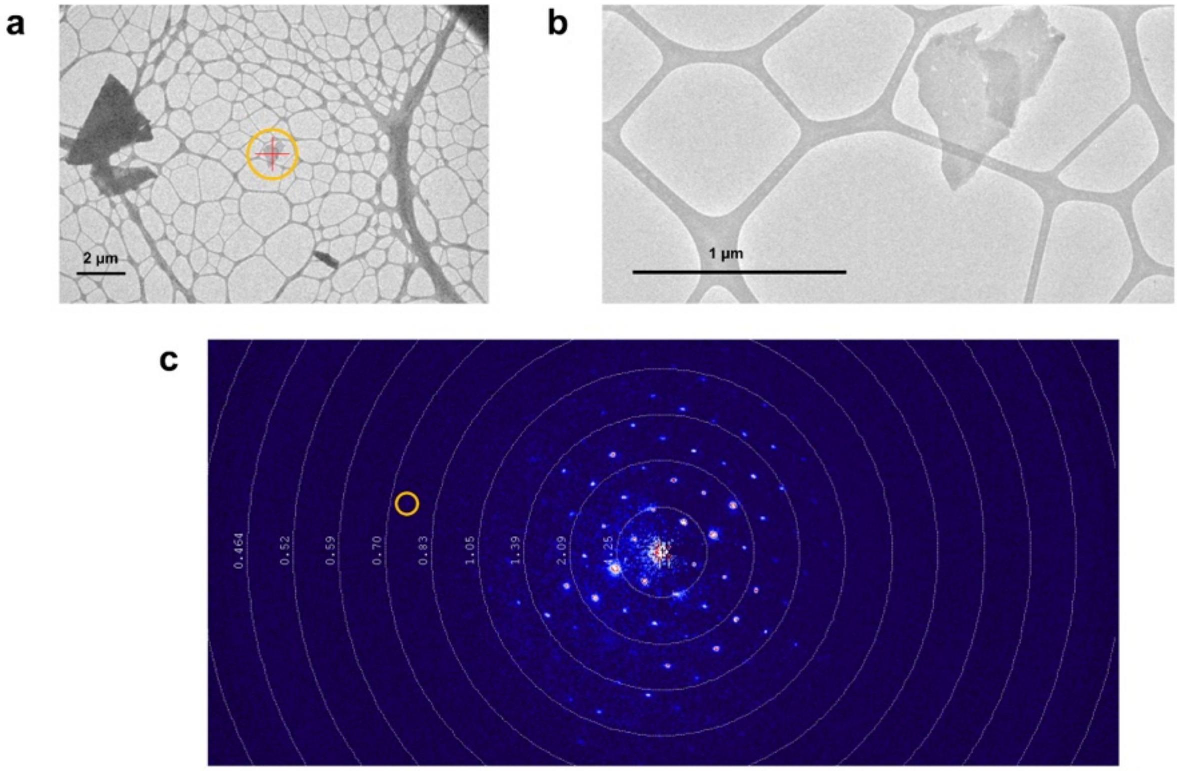

Supplementary Figure 118. a,b) Grain snapshot of a thermodynamic aggregated DSAI nanocrystal. c) Diffraction pattern showing diffraction beyond 0.80 Å resolution.

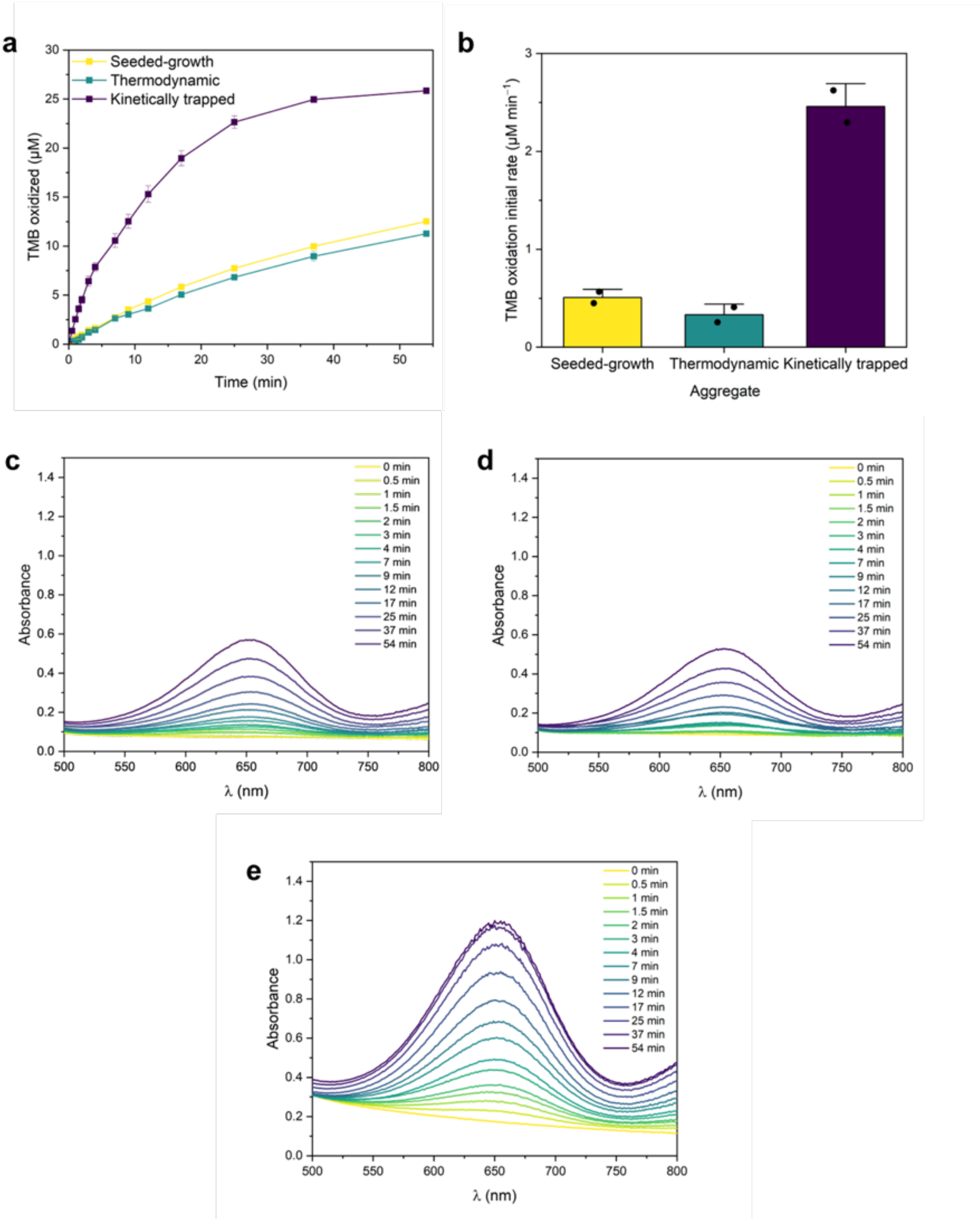

Supplementary Figure 119. a) Irradiation of various DSAI aggregated states (0.1 mM) with NaI (0.6 mM) in presence of TMB (134 μM) over time in acetate buffer (0.1 M, pH = 5) under white light (180 mW cm^{-2}) under air. b) TMB oxidation rate extracted from traces in panel a): 0.51 ± 0.08, 0.33 ± 0.11, 2.5 ± 0.2 μM min^{-1} (from left to right). Statistics are from 2 independent groups. c) UV-Vis traces using DSA seeded-growth aggregate; d) UV-Vis traces using DSAI thermodynamic aggregate; d) UV-Vis traces using DSAI kinetically trapped aggregate.

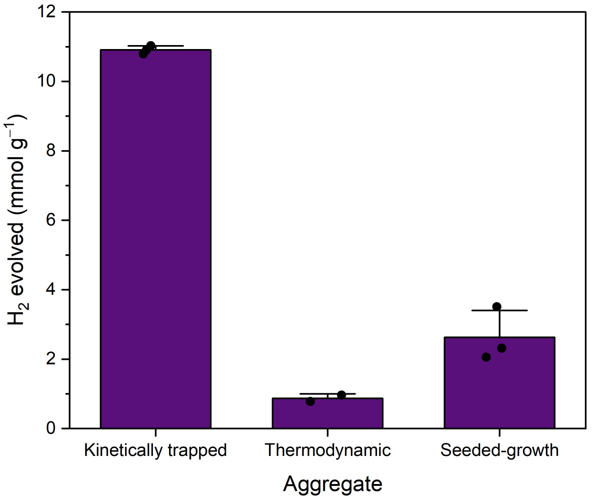

Supplementary Figure 120. H_2 evolution (10.9 ± 0.1, 0.87 ± 0.13, 2.6 ± 0.8 mmol g^{-1}) by irradiation of various DSAI aggregated states (0.1 mM, NaI 25 mM) with white light (LED, 100 mW cm^{-2}) in presence of ascorbic acid (1.0 M, pH = 4.0) and co-catalyst (PtNPs, 8% mol). Statistics are from 2 or 3 independent groups.

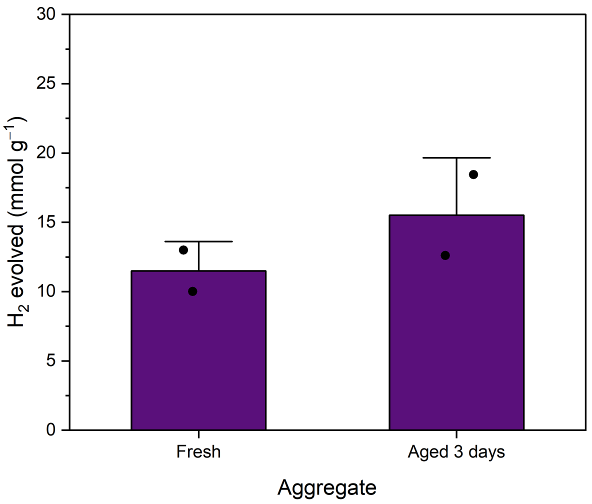

Supplementary Figure 121. H_2 evolved by fresh (12 ± 2 mmol g^{-1}) and aged three days(15 ± 4 mmol g^{-1}) kinetically trapped aggregate (DSAI 0.1 mM, NaI 25 mM, ascorbic acid 1.0 M, pH = 4.0, PtNPs 8% mol) after 4h irradiation under white light (100 mW cm^{-2}). Statistics are from 2 independent groups.

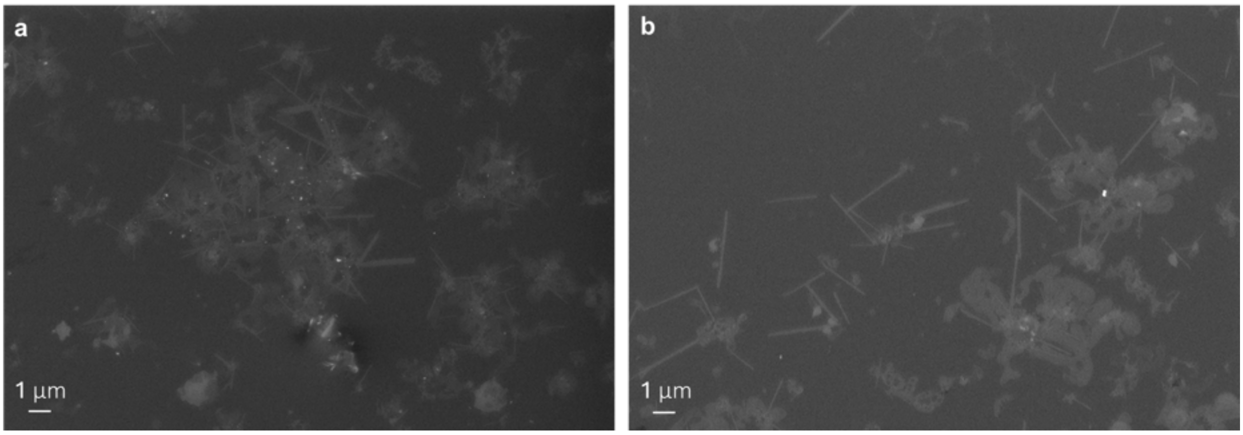

Supplementary Figure 122. SEM images of DSAI 0.1 mM in ascorbic acid (1.0 M, pH = 4.0) in presence of NaI (25 mM) and PtNPs (8% mol) aggregates a) pre- and b) post- photocatalytic H_2 evolution. These images show that the morphology is strictly retained in these kinetically trapped aggregates.

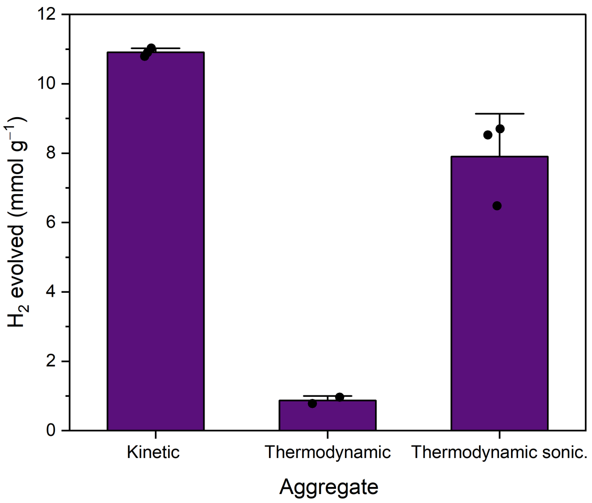

Supplementary Figure 123. H_2 evolution (10.9 ± 0.1, 0.87 ± 0.13, 7.9 ± 1.4 mmol g^{-1}) by irradiation of various DSAI aggregated states (0.1 mM) with white light (LED, 100 mW cm^{-2}) in presence of ascorbic acid (1.0 M, pH = 4.0) and co-catalyst (PtNPs, 8% mol). Statistics are from 2 or 3 independent groups.

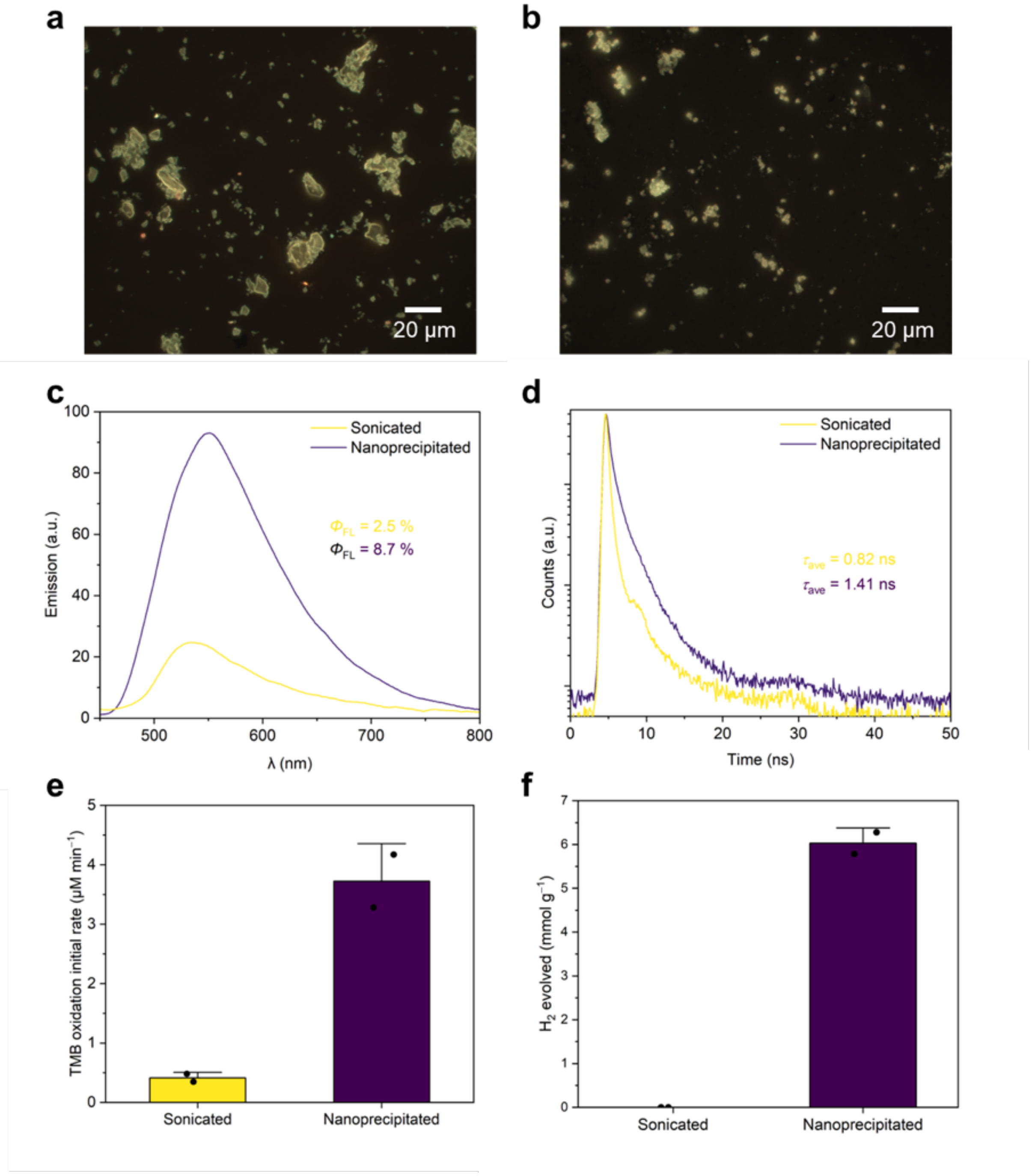

Supplementary Figure 124. Widefield fluorescence images of DSAPF6 0.4 mg mL^{-1} a) sonicated in mQ-water and b) nanoprecipitated from DMSO in mQ-water. c) Fluorescence emission spectra and fluorescence quantum yields (Φ_{FL}) of DSAPF6 0.4 mg mL^{-1} sonicated *vs* nanoprecipitated in acetate buffer (0.1 M, pH 0 5) containing 3.2% DMSO (*v/v*). d) Time-correlated single-photon counting (TCSPC) decay traces of the same samples. e) TMB oxidation rate (0.41 ± 0.09, 3.7 ± 0.6 µM min^{-1}) of same samples under white light (180 mW cm^{-2}). f) H_2 evolution produced by DSAPF6 0.4 mg mL^{-1} in ascorbic acid (1.0 M, pH = 4.0) sonicated (0.0 ± 0.0 mmol g^{-1}) *vs* nanoprecipitated from DMSO (6.0 ± 0.3 mmol g^{-1}), in presence of PtNPs (8% mol) under 4 hours white light irradiation (100 mW cm^{-2}). Statistics are from 2 independent groups.

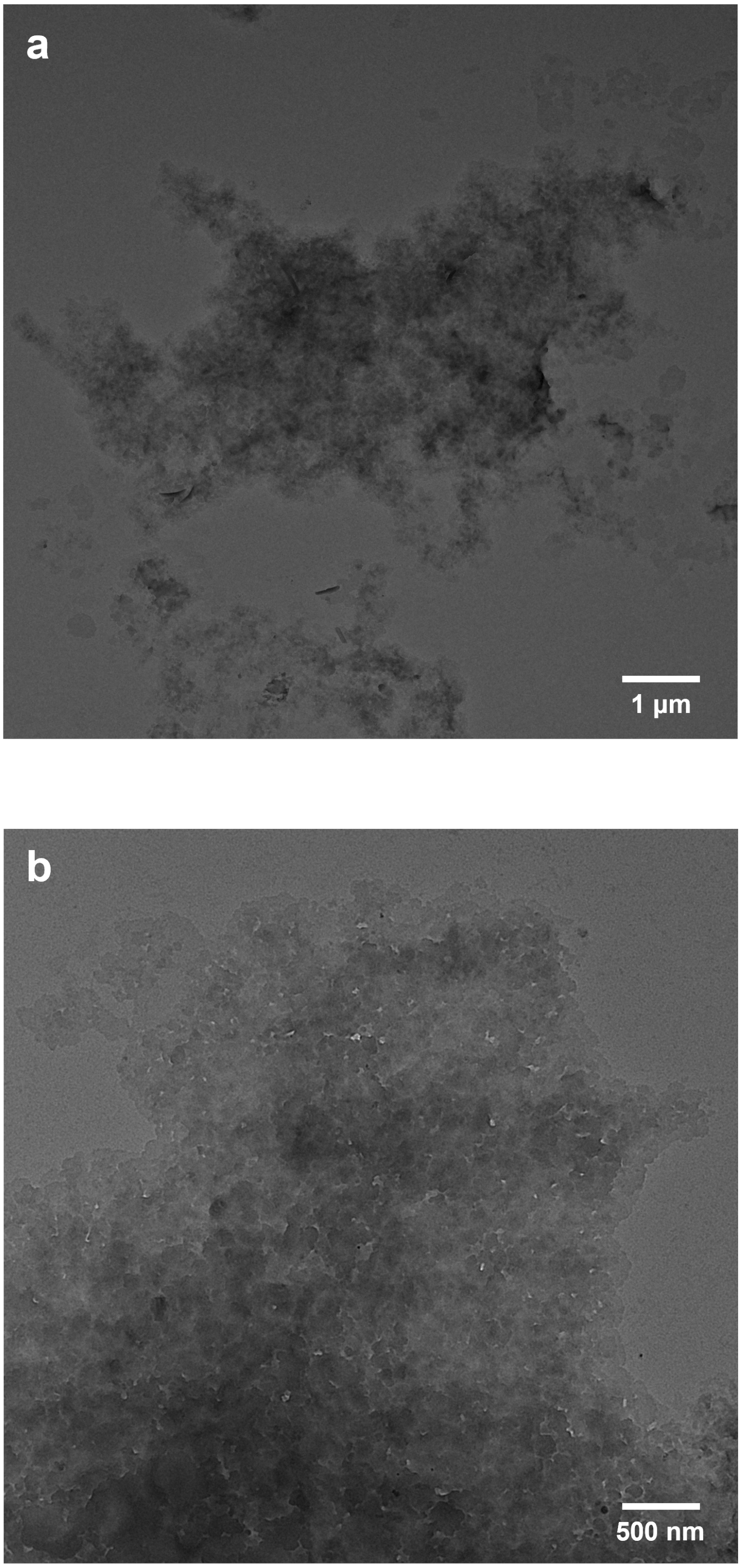

Supplementary Figure 125. a,b) TEM micrographs of TBATPE 0.1 mM in ascorbic acid 5 mM (pH = 4.0).

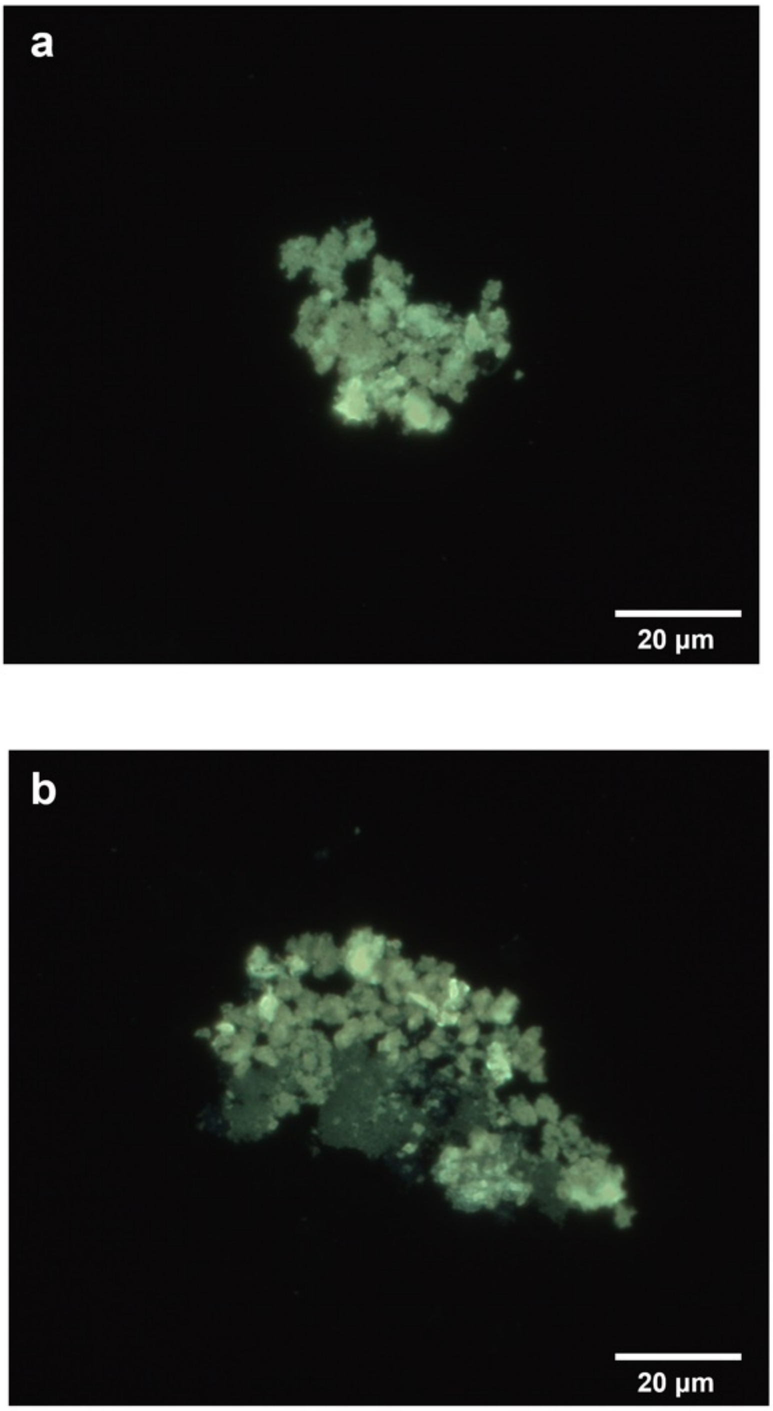

Supplementary Figure 126. a,b) Fluorescence microscopy micrographs of TBATPE 0.1 mM in ascorbic acid 5 mM (pH = 4.0).

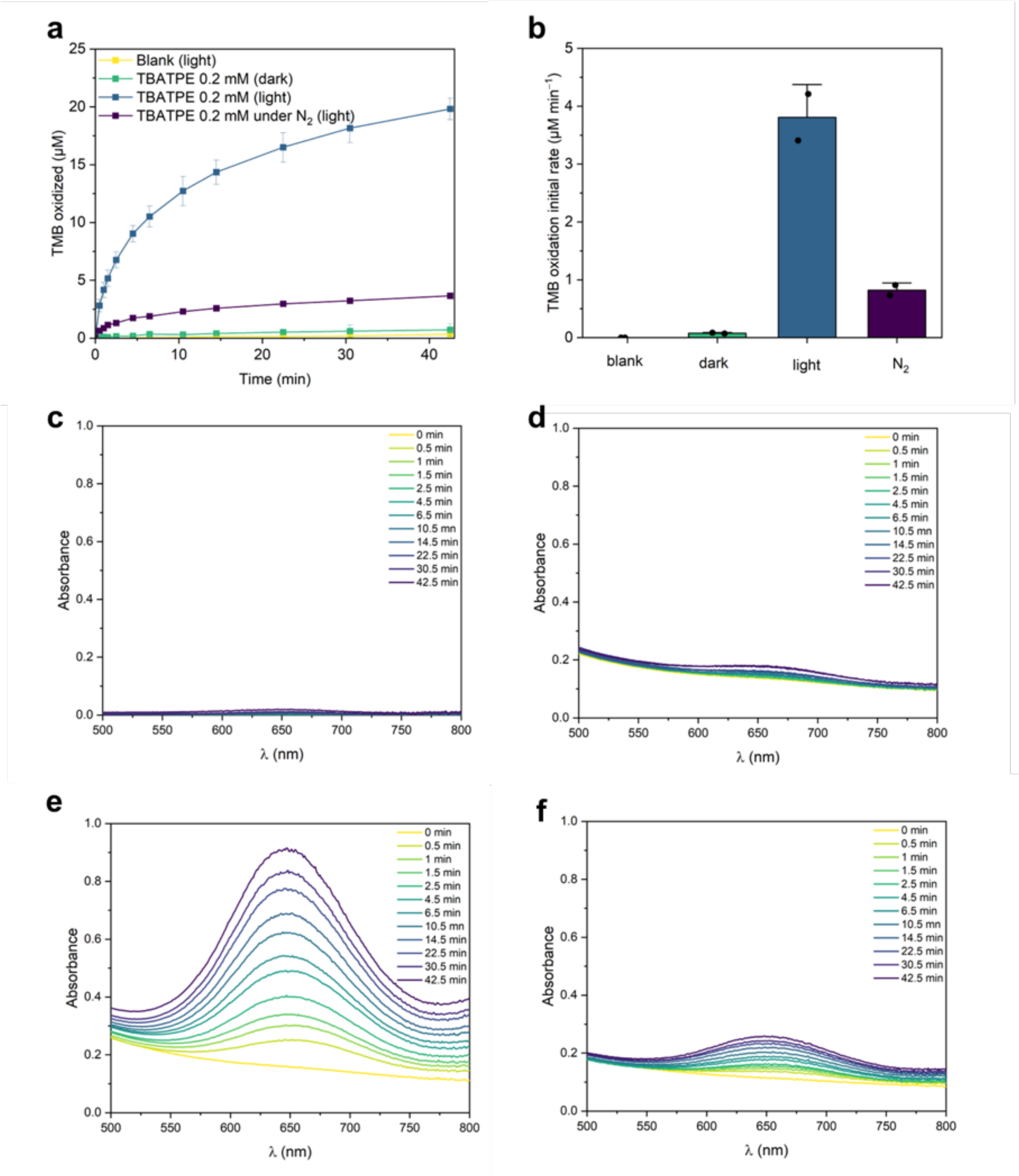

Supplementary Figure 127. a) Irradiation of TBATPE (0.2 mM) aggregated with acetate buffer (60 mM, pH = 5.0) in presence of TMB (134 μM) over time under white light (180 mW cm^{-2}) or dark under different atmospheres. b) TMB oxidation rate extracted from traces in panel a): 0.0 ± 0.0, 0.08 ± 0.01, 3.8 ± 0.6, 0.82 ± 0.12 μM min^{-1} (from left to right). Statistics are from 2 independent groups. c) UV-Vis traces without TBATPE under air; d) UV-Vis traces under dark under air; e) UV-Vis traces of the positive control; f) UV-Vis traces under nitrogen atmosphere.

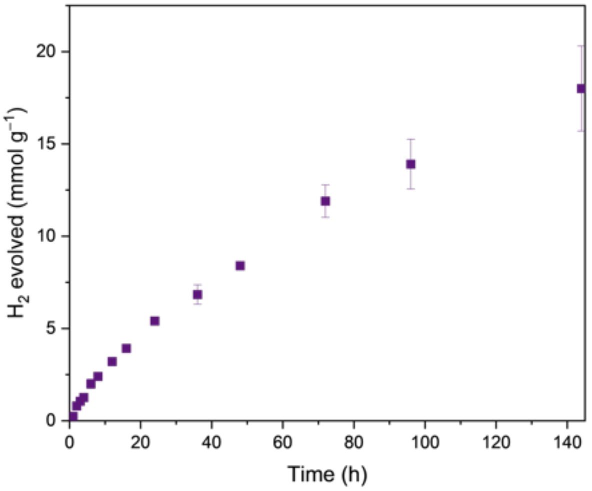

Supplementary Figure 128. Kinetic run of H_2 evolution photosensitized by TBATPE (0.1 mM) in ascorbic acid (0.8 M, pH = 4.0), PtNPs 8% mol, under 415 nm irradiation (140 mW cm^{-2}). Statistics are from 3 independent groups.

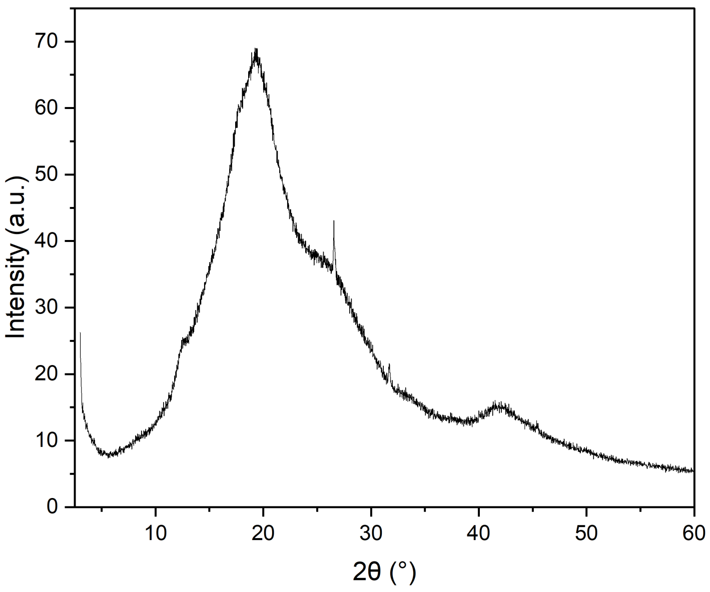

Supplementary Figure 129. PXRD diffraction pattern of TBATPE from synthesis**.**

5. Supplementary Tables

Supplementary Table 1. Data collection parameter overview.

Dataset (grain)	Scan range [°]	Scan width [°]	Number of frames	Exposure time/frame [s]	Total exposure time [s]	Dose rate [$e^-/(Å^2 \cdot s)$]	Dose [$e^-/(Å^2)$]
**10286**	-65 to +60	0.50	250	0.50	125	1.26E-02	1.57
**10293**	-70 to +75	0.50	290	0.25	72	1.26E-02	0.91
**10294**	-70 to +73	0.50	286	0.25	71	1.26E-02	0.90
**10296**	-65 to +60	0.50	250	0.25	62	1.27E-02	0.79

Supplementary Table 2. Data quality statistics overview for all single data collections and the final merged dataset of DSAI. They were all processed up to a resolution of 0.83 Å. Point group symmetry: *P*-1.

Dataset (grain)	Data	Compl. [%]	Redund.	$\langle F^2 \rangle$	$\langle F^2/\sigma(F^2) \rangle$	R_{int}	R_{pim}	R_{rim}	CC1/2
**10286**	4113	67.9	2.0	16405.48	5.58	0.058	0.058	0.085	1.000
**10293**	4805	78.9	2.0	66275.66	9.40	0.033	0.033	0.047	0.999
**10294**	4628	76.1	2.0	142933.23	13.09	0.023	0.023	0.033	1.000
**10296**	4109	68.0	2.0	46324.09	5.65	0.060	0.060	0.085	0.993
**merged**	16397	98.0	5.4	40758.52	5.88	0.197	0.088	0.213	0.955

Supplementary Table 3. 3D ED crystal data and structure refinement for DSAI.

Compound name	DSAI
CCDC number	2485494
Chemical formula	$C_{36}H_{38}I_2N_2$
Formula weight	752.51 $g \cdot mol^{-1}$
Crystal description	Yellow plate
Crystal system	Triclinic
Space group	*P*–1
a (Å)	5.6711(6)
b (Å)	7.8068(8)
c (Å)	19.074(2)
α (°)	92.743(10)
β (°)	91.389(10)
γ (°)	90.402(9)
Volume ($Å^3$)	843.22(16)
Z, Z′	1, 0.5
Temperature (K)	298
m(transmission electron microscope)	0
Reflections collected	16 397
Independent reflections	3 035
Observed reflections [$I_0 > 2\sigma(I_0)$]	2 051
Parameters refined	185
Restraints	265
Goodness-of-fit on F^2	2.663
R_1, wR_2 [$I_0 > 2\sigma(I_0)$]	0.2332, 0.5190
R_1, wR_2 (all data)	0.2674, 0.5331
Largest diff. peak/hole ($e \cdot Å^{-3}$)	+0.392 / –0.234

Supplementary Table 4. Cartesian coordinates of optimized compound DSAI monomer with the r^2SCAN-3c composite method (water, SMD method).

N	-2.7117747727	9.6803589799	2.3796296833
C	-1.8310247927	9.4149327747	3.5595634916
C	-0.8391729137	10.3434732578	3.8590144549
H	-0.7024080963	11.2423313672	3.2649095302
C	-0.0015390907	10.1209205575	4.9403325321
H	0.7715594781	10.8500051503	5.1672230442
C	-0.1251696986	8.9730694344	5.7392594575
C	-1.1451934999	8.0649517194	5.4226827907
H	-1.2998254505	7.1774093433	6.0276763917
C	-1.9902427002	8.2761770440	4.3411330583
H	-2.7615713720	7.5444654937	4.1395475041
C	-1.8683739792	9.7388963450	1.1266360493
H	-1.1590725821	10.5634334995	1.2055329549
H	-2.5376017265	9.9032743081	0.2798193074
H	-1.3432993906	8.7876032247	1.0246391683
C	1.9288634867	7.4912955343	8.6546614431
C	2.8083020263	5.4708124704	7.5331479635
H	2.1076430932	5.6205812055	6.7190070623
C	1.0493762575	9.4714221889	9.8778719604
H	0.2773255205	9.5972387614	9.1284590209
C	-3.7557879462	8.6170827273	2.1783005280
H	-3.2661150031	7.6593564925	1.9963846336
H	-4.3421250484	8.9034179141	1.3038316159
H	-4.3997491400	8.5718128225	3.0574543625
C	0.8047959815	8.7871697333	6.8487478714
H	1.4306362353	9.6494192599	7.0768938419
C	-3.4277138320	10.9989253606	2.5632170094
H	-4.0132282157	10.9437644332	3.4825321267
H	-4.0786221504	11.1490538462	1.6996273506
H	-2.6961427518	11.8054621819	2.6186459918
C	1.9783803762	8.3968261005	9.7429495016
C	2.8101246005	6.3830978063	8.6297666919
C	0.9753582820	7.6542468886	7.5529159348
H	0.4107300637	6.7661762392	7.2724138892
C	1.0929850759	10.3281931594	10.9438090059
H	0.3659389291	11.1313868826	11.0221128638
C	3.6880048797	4.4249268786	7.4695852477
H	3.6636622245	3.7450677109	6.6227666874
N	8.4605082390	4.9451889418	17.0637509064
C	7.5827815552	5.1969952478	15.8792683892
C	6.5923642539	4.2650196155	15.5841614228
H	6.4563829161	3.3727609644	16.1849562597
C	5.7571526709	4.4776439541	14.4989165265
H	4.9852033900	3.7464658078	14.2758078935
C	5.8814875857	5.6191785483	13.6912230591
C	6.9009267738	6.5298366152	14.0016935680
H	7.0570414869	7.4117515231	13.3888142595
C	7.7431659194	6.3283373268	15.0871027026
H	8.5138983609	7.0619292143	15.2839136484
C	7.6156240854	4.8971191373	18.3157133904
H	6.9023240485	4.0771096274	18.2402879838
H	8.2829355772	4.7359477124	19.1644704322
H	7.0930975641	5.8504756895	18.4115184027
C	3.8228610596	7.0895129384	10.7733274953
C	2.9423299462	9.1091870558	11.8955931850
H	3.6430058118	8.9595108486	12.7097348402
C	4.7012033678	5.1083734704	9.5509707748
H	5.4732822800	4.9824617506	10.3003255560
C	9.5019994479	6.0123938260	17.2578801590
H	9.0104581593	6.9713851995	17.4271448829
H	10.0850249272	5.7363021726	18.1377636576
H	10.1497712658	6.0488172816	16.3812303592
C	4.9515411853	5.7976746618	12.5804450507
H	4.3280489753	4.9330001618	12.3550504908
C	9.1833887853	3.6257635939	16.8945802365
H	9.7729849831	3.6768835981	15.9780038680
H	9.8294817034	3.4859632759	17.7629766530
H	8.4554788226	2.8177467908	16.8388430720
C	3.7727567540	6.1834651034	9.6855260941
C	2.9409708107	8.1972245388	10.7986845247
C	4.7776378227	6.9288308362	11.8742426533
H	5.3403469290	7.8185649829	12.1534437329
C	4.6570444292	4.2512096774	8.4853608174
H	5.3836501375	3.4475915945	8.4073386073
C	2.0621075927	10.1546092892	11.9595141606
H	2.0861359044	10.8341992992	12.8065625068
I	-0.1764650693	13.7825988564	1.4570757282
I	10.9981671842	0.2146201730	16.5071000258

Supplementary Table 5. Cartesian coordinates of optimized compound DSAI dimer with the r²SCAN-3c composite method (water, SMD method).

N	2.5573956259	9.5962444804	1.8324923661
C	3.3254573103	9.4306667520	3.1047992540
C	4.2976228628	10.3777508143	3.4132422398
H	4.4762536580	11.2330361117	2.7712378591
C	5.0659365669	10.2208941979	4.5558650871
H	5.8282125126	10.9601156777	4.7858650778
C	4.8970693474	9.1150467924	5.4040778182
C	3.8890875098	8.1955867572	5.0847297366
H	3.6962455644	7.3471766206	5.7338572411
C	3.1087724793	8.3444481321	3.9457240633
H	2.3423933939	7.6061756972	3.7458631878
C	3.5038849356	9.4479856668	0.6634535404
H	4.2659015136	10.2252086881	0.7210547653
H	2.9239734308	9.5545626580	-0.2555591667
H	3.9609653172	8.4583023099	0.7184537546
C	6.9128614314	7.6740365039	8.3628051556
C	7.7210829434	5.6138230066	7.2593737332
H	7.0129422474	5.7688656060	6.4529665707
C	6.1004826070	9.6882238460	9.5727162757
H	5.3113923990	9.8144962265	8.8409840136
C	1.4673417327	8.5695645442	1.6740291992
H	1.9135212623	7.5760469952	1.6319857726
H	0.9591465216	8.7838687783	0.7332009554
H	0.7669624962	8.6525397526	2.5075365761
C	5.7904903569	8.9629525110	6.5480514040
H	6.4158675503	9.8276044096	6.7678164869
C	1.9121695455	10.9629015930	1.7810704491
H	1.2909167275	11.0779532151	2.6688932765
H	1.3103731552	11.0134781734	0.8724499574
H	2.6842539017	11.7309236796	1.7547354477
C	7.0049433453	8.5942853751	9.4352223695
C	7.7660502721	6.5437783714	8.3401494638
C	5.9503374422	7.8392199022	7.2703742101
H	5.3832563635	6.9505199731	6.9952831692
C	6.1794606578	10.5557070979	10.6281569178
H	5.4653770490	11.3698664239	10.7133842312
C	8.5619117974	4.5364402041	7.2045552549
H	8.5014297795	3.8418399026	6.3715473971
N	13.7047544979	4.8415455591	16.4827876923
C	12.7643302296	5.1717661609	15.3664671337
C	11.8557752009	4.2002299183	14.9618607320
H	11.8287950374	3.2185259131	15.4255135240
C	10.9497299028	4.4893909140	13.9535307262
H	10.2416720520	3.7249830111	13.6448013730
C	10.9299799639	5.7433442260	13.3236730491
C	11.8500411082	6.7067371731	13.7610956085

H	11.8711031591	7.6948427326	13.3125877709
C	12.7591903306	6.4299912570	14.7733479482
H	13.4482854299	7.2084726529	15.0728082617
C	12.9114919889	4.6990502635	17.7617351784
H	12.1898583112	3.8882794908	17.6466253668
H	13.6112335574	4.4684620861	18.5678556163
H	12.4051472085	5.6483570170	17.9504561004
C	8.8319287137	7.2563696636	10.4553976931
C	8.0251775571	9.3147211255	11.5593163161
H	8.7395245147	9.1604127713	12.3612170326
C	9.6248227106	5.2221741906	9.2596945190
H	10.4036853182	5.0836553247	9.9994672440
C	14.7459316248	5.9058252476	16.7001564588
H	14.2581475878	6.8347918608	16.9997121005
H	15.3977675349	5.5598591456	17.5038067830
H	15.3197184386	6.0372181855	15.7814373509
C	9.9644547692	5.9646091755	12.2529647038
H	9.3087076268	5.1189830813	12.0472026896
C	14.4296878391	3.5479000133	16.1927901780
H	14.9582020792	3.6579666839	15.2442257323
H	15.1342825030	3.3699585742	17.0072405802
H	13.7138057058	2.7277419251	16.1444736220
C	8.7348135444	6.3308603456	9.3869028053
C	7.9825572328	8.3884744825	10.4753901232
C	9.8106842885	7.0901892927	11.5349949997
H	10.4178419273	7.9628490718	11.7709426023
C	9.5366799395	4.3468729961	8.2119508695
H	10.2336996834	3.5168640596	8.1404341631
C	7.1638550051	10.3759817943	11.6281115275
H	7.2153730679	11.0642495831	12.4669084672
N	-2.8325834695	9.7911294895	2.5065788367
C	-1.8926611233	9.4615205320	3.6235692210
C	-0.9829129358	10.4326905801	4.0264269723
H	-0.9546731114	11.4133981471	3.5607755504
C	-0.0769989915	10.1440994285	5.0349851043
H	0.6318603997	10.9082333333	5.3425405848
C	-0.0587176422	8.8911909783	5.6670163331
C	-0.9801354419	7.9282503580	5.2314882282
H	-1.0022966513	6.9409173545	5.6817706864
C	-1.8891256450	8.2043327244	4.2189265906
H	-2.5793508917	7.4262629702	3.9210113916
C	-2.0387828108	9.9333236876	1.2279128108
H	-1.3174831483	10.7444169018	1.3426488098
H	-2.7381973805	10.1633245545	0.4213256763
H	-1.5322497932	8.9839726134	1.0398251003
C	2.0397733342	7.3793671586	8.5356317679

C	2.8465676801	5.3210820068	7.4316963874
H	2.1314394592	5.4752230934	6.6304447551
C	1.2468892882	9.4136274304	9.7311289718
H	0.4681090665	9.5521287566	8.9912669149
C	-3.8732246547	8.7264528907	2.2887875165
H	-3.3844581202	7.7974400365	1.9907101837
H	-4.5241442324	9.0714648946	1.4839104434
H	-4.4484149647	8.5957553822	3.2067264271
C	0.9069130679	8.6705064195	6.7377369660
H	1.5626156769	9.5163523160	6.9429483155
C	-3.5579353545	11.0846789075	2.7960255777
H	-4.0866873211	10.9742696981	3.7443840473
H	-4.2623824352	11.2622917669	1.9813960083
H	-2.8423223084	11.9050786178	2.8446105198
C	2.1368630062	8.3049598895	9.6040922547
C	2.8894052844	6.2474931293	8.5155655936
C	1.0609166514	7.5452153246	7.4561063095
H	0.4536847341	6.6724470473	7.2206519793
C	1.3350119401	10.2890409679	10.7787850078
H	0.6380236954	11.1190656639	10.8500788941
C	3.7077116085	4.2597385013	7.3629190468
H	3.6559874092	3.5710501662	6.5244888301
N	8.3170405980	5.0430846794	17.1577836506
C	7.5486171997	5.2078477028	15.8856768296
C	6.5764970046	4.2604431419	15.5779903895
H	6.3982811818	3.4053116813	16.2203503903
C	5.8077986116	4.4166352851	14.4355444967
H	5.0456864863	3.6770609507	14.2061375304
C	5.9758567207	5.5223781899	13.5870318250
C	6.9837212797	6.4422404837	13.9057109284
H	7.1757185201	7.2907121023	13.2564282135
C	7.7646677165	6.2938468785	15.0442934851

H	8.5310474691	7.0322566123	15.2436861494
C	7.3706630989	5.1905295788	18.3270455463
H	6.6097386116	4.4121996832	18.2700378437
H	7.9511473827	5.0852137567	19.2458031197
H	6.9121611486	6.1795274170	18.2717181028
C	3.9590230313	6.9619057809	10.6280985747
C	3.1504731761	9.0220155555	11.7315090613
H	3.8585746416	8.8670547961	12.5379988691
C	4.7714171927	4.9476134930	9.4182844051
H	5.5603111513	4.8211146056	10.1502026399
C	9.4063241919	6.0706230108	17.3159694236
H	8.9592985310	7.0637606779	17.3583781289
H	9.9153236641	5.8566242358	18.2564217499
H	10.1063243415	5.9885088148	16.4820689037
C	5.0819811132	5.6739120472	12.4433274775
H	4.4566396182	4.8089670833	12.2242264956
C	8.9636082768	3.6770260800	17.2091565963
H	9.5856366871	3.5628616882	16.3217624476
H	9.5648888817	3.6263771517	18.1181114085
H	8.1921947717	2.9082944215	17.2346709186
C	3.8670284714	6.0416670959	9.5557151832
C	3.1056360809	8.0920171234	10.6508206790
C	4.9218483746	6.7971750947	11.7203406385
H	5.4888616767	7.6861888326	11.9945637938
C	4.6922986239	4.0801223261	8.3628813848
H	5.4062477112	3.2658475044	8.2778170451
C	2.3097067364	10.0994258258	11.7862836117
H	2.3702582384	10.7941037466	12.6191948155
I	-0.9889867546	5.5836777011	1.2680302661
I	-0.4102793985	14.0845904980	1.8689446162
I	11.2777210507	0.5528063691	17.1240863120
I	11.8630822105	9.0517921606	17.7264716831

Supplementary Table 6. First 6 electronic transitions calculated for DSAI monomer using CAM-B3LYP/6-31G*/LanL2DZ level of theory. Contributions smaller than 10% are not reported.

No.	Energy (cm^{-1})	λ (nm)	Osc. Strength	Symmetry	Major contribs
1	23693	422	0.8852	Singlet-A	HOMO→LUMO (97%)
2	31730	315	0.0013	Singlet-A	H-9→LUMO (42%), HOMO→L+3 (43%)
3	33540	298	0.3541	Singlet-A	HOMO→L+2 (87%)
4	33722	297	0.0062	Singlet-A	HOMO→L+1 (83%)
5	36739	272	0.0745	Singlet-A	H-8→LUMO (12%) H-7→LUMO (71%)
6	36795	272	0.3273	Singlet-A	H-8→LUMO (71%), H-7→LUMO (11%)

Supplementary Table 7. First 6 electronic transitions calculated for DSAI dimer using CAM-B3LYP/6-31G*/LanL2DZ level of theory. Contributions smaller than 10% are not reported.

No.	Energy (cm^{-1})	λ (nm)	Osc. Strength	Symmetry	Major contribs
1	22963	435	0.0	Singlet-A	H-1→L+1 (29%), HOMO→LUMO (67%)
2	23859	419	1.6165	Singlet-A	H-1→LUMO (48%), HOMO→L+1 (49%)
3	27749	360	0.0018	Singlet-A	H-1→LUMO (47%), HOMO→L+1 (46%)
4	28087	356	0.0	Singlet-A	H-1→L+1 (67%), HOMO→LUMO (29%)
5	31632	316	0.0	Singlet-A	H-19→L+1 (12%), H-18→LUMO (16%), H-1→L+7 (20%), HOMO→L+6 (24%)
6	31773	314	0.0003	Singlet-A	H-19→LUMO (13%), H-18→L+1 (13%), H-1→L+6 (19%), HOMO→L+7 (22%)

6. References

1. Jonkheijm, P., Van Der Schoot, P., Schenning, A. P. H. J. & Meijer, E. W. Probing the Solvent-Assisted Nucleation Pathway in Chemical Self-Assembly. *Science* **313**, 80–83 (2006).
2. Ogi, S., Stepanenko, V., Sugiyasu, K., Takeuchi, M. & Würthner, F. Mechanism of Self-Assembly Process and Seeded Supramolecular Polymerization of Perylene Bisimide Organogelator. *J. Am. Chem. Soc.* **137**, 3300–3307 (2015).
3. Wehner, M. & Würthner, F. Supramolecular polymerization through kinetic pathway control and living chain growth. *Nat. Rev. Chem.* **4**, 38–53 (2019).
4. Schindelin, J. *et al.* Fiji: an open-source platform for biological-image analysis. *Nat. Methods* **9**, 676–682 (2012).
5. Nečas, D. & Klapetek, P. Gwyddion: an open-source software for SPM data analysis. *Open Phys.* **10**, 181–188 (2012).
6. Ito, S. *et al.* Structure determination of small molecule compounds by an electron diffractometer for 3D ED/MicroED. *CrystEngComm* **23**, 8622–8630 (2021).
7. Truong, K.-N. *et al.* Making the Most of 3D Electron Diffraction: Best Practices to Handle a New Tool. *Symmetry* **15**, 1555 (2023).
8. Rigaku Oxford Diffraction. CrysAlisPRO v. 1.171.44.120a. Rigaku Corporation (2023).
9. Sheldrick, G. M. *SHELXT* – Integrated space-group and crystal-structure determination. *Acta Crystallogr. A* **71**, 3–8 (2015).
10. Sheldrick, G. M. Crystal structure refinement with *SHELXL*. *Acta Crystallogr. C* **71**, 3–8 (2015).
11. Rigaku Corporation. AutoChem 7 software system in conjunction with OLEX2 v. 1.5-ac7-018. Rigaku Oxford Diffraction (2023).

12. Dolomanov, O. V., Bourhis, L. J., Gildea, R. J., Howard, J. A. K. & Puschmann, H. *OLEX2*: a complete structure solution, refinement and analysis program. *J. Appl. Crystallogr.* **42**, 339–341 (2009).
13. Hirshfeld, F. L. *Acta Cryst.* **A32**, 239–244 (1976).
14. Thorn, A., Dittrich, B. & Sheldrick, G. M. Enhanced rigid-bond restraints. *Acta Crystallogr. A* **68**, 448–451 (2012).
15. Zhao, H.-M. *et al.* Understanding Ground- and Excited-State Properties of Perylene Tetracarboxylic Acid Bisimide Crystals by Means of Quantum Chemical Computations. *J. Am. Chem. Soc.* **131**, 15660–15668 (2009).
16. Engels, B. & Engel, V. The dimer-approach to characterize opto-electronic properties of and exciton trapping and diffusion in organic semiconductor aggregates and crystals. *Phys. Chem. Chem. Phys.* **19**, 12604–12619 (2017).
17. Draper, E. R. *et al.* pH-Directed Aggregation to Control Photoconductivity in Self-Assembled Perylene Bisimides. *Chem* **2**, 716–731 (2017).
18. Grimme, S., Hansen, A., Ehlert, S. & Mewes, J.-M. r2SCAN-3c: A "Swiss army knife" composite electronic-structure method. *J. Chem. Phys.* **154**, 064103 (2021).
19. Bursch, M., Mewes, J., Hansen, A. & Grimme, S. Best-Practice DFT Protocols for Basic Molecular Computational Chemistry**. *Angew. Chem. Int. Ed.* **61**, e202205735 (2022).
20. Neese, F. Software update: The ORCA program system—Version 5.0. *WIREs Comput. Mol. Sci.* **12**, e1606 (2022).
21. Yanai, T., Tew, D. P. & Handy, N. C. A new hybrid exchange–correlation functional using the Coulomb-attenuating method (CAM-B3LYP). *Chem. Phys. Lett.* **393**, 51–57 (2004).

22. Petersson, G. A. *et al.* A complete basis set model chemistry. I. The total energies of closed-shell atoms and hydrides of the first-row elements. *J. Chem. Phys.* **89**, 2193–2218 (1988).

23. Hay, P. J. & Wadt, W. R. *Ab initio* effective core potentials for molecular calculations. Potentials for K to Au including the outermost core orbitals. *J. Chem. Phys.* **82**, 299–310 (1985).

24. Frisch, M. J. *et al.* Guassian 16, Revision B.01. Gaussian, Inc. (2016).

25. Dennington, R., Keith, T. A.; & Millam, J. M. Gaussview 5. Semichem Inc., Shawnee Mission KS (2016).

26. O'boyle, N. M., Tenderholt, A. L. & Langner, K. M. cclib: A library for package-independent computational chemistry algorithms. *J. Comput. Chem.* **29**, 839–845 (2008).

27. Johnson, E. R. *et al.* Revealing Noncovalent Interactions. *J. Am. Chem. Soc.* **132**, 6498–6506 (2010).

28. Lu, T. A comprehensive electron wavefunction analysis toolbox for chemists, Multiwfn. *The Journal of Chemical Physics* **161**, 082503 (2024).

29. Lu, T. & Chen, F. Multiwfn: A multifunctional wavefunction analyzer. *J. Comput. Chem.* **33**, 580–592 (2012).

30. Arcudi, F., Đorđević, L., Schweitzer, N., Stupp, S. I. & Weiss, E. A. Selective visible-light photocatalysis of acetylene to ethylene using a cobalt molecular catalyst and water as a proton source. *Nat. Chem.* **14**, 1007–1012 (2022).

31. Josephy, P. D., Eling, T. & Mason, R. P. The horseradish peroxidase-catalyzed oxidation of 3,5,3',5'-tetramethylbenzidine. Free radical and charge-transfer complex intermediates. *J. Biol. Chem.* **257**, 3669–3675 (1982).

32. Jakešová, M. *et al.* Hydrogen-Bonded Organic Semiconductors as Stable Photoelectrocatalysts for Efficient Hydrogen Peroxide Photosynthesis. *Adv. Funct. Mater.* **26**, 5248–5254 (2016).
33. Buzzetti, L., Crisenza, G. E. M. & Melchiorre, P. Mechanistic Studies in Photocatalysis. *Angew Chem Int Ed* **58**, 3730–3747 (2019).
34. Zhao, W. *et al.* Nanoscale covalent organic frameworks for enhanced photocatalytic hydrogen production. *Nat Commun* **15**, 6482 (2024).
35. Masnovi, J. M., Seddon, E. A. & Kochi, J. K. Electron transfer from anthracenes. Comparison of photoionization, charge-transfer excitation and electrochemical oxidation. *Can. J. Chem.* **62**, 2552–2559 (1984).
36. Zhang, H. C., Guo, E. Q., Zhang, Y. L., Ren, P. H. & Yang, W. J. Donor−Acceptor-Substituted Anthracene-Centered Cruciforms: Synthesis, Enhanced Two-Photon Absorptions, and Spatially Separated Frontier Molecular Orbitals. *Chem. Mater.* **21**, 5125–5135 (2009).
37. Zhou, Y. *et al.* A cationic on–off fluorescent sensor with AIE properties for heparin and protamine detection. *New J. Chem.* **45**, 16537–16542 (2021).
38. Biesen, L., Krenzer, J., Nirmalananthan-Budau, N., Resch-Genger, U. & Müller, T. J. J. Asymmetrically bridged aroyl-*S,N*-ketene acetal-based multichromophores with aggregation-induced tunable emission. *Chem. Sci.* **13**, 5374–5381 (2022).
39. Wei, Z. *et al.* Rigidifying Fluorescent Linkers by Metal–Organic Framework Formation for Fluorescence Blue Shift and Quantum Yield Enhancement. *J. Am. Chem. Soc.* **136**, 8269–8276 (2014).
40. Ghosh, I. *et al.* Organic semiconductor photocatalyst can bifunctionalize arenes and heteroarenes. *Science* **365**, 360–366 (2019).
