# Supplementary material for: Supramolecular dye polymers for aggregation-induced photocatalysis
Source: Nat Chem. 2026 May 18;18(8):1372–82. doi: 10.1038/s41557-026-02151-4 (PMC13423815; doi:10.1038/s41557-026-02151-4)
Supplement: Supplementary file 2 — Peer Review File [file 41557_2026_2151_MOESM2_ESM.pdf]

nature portfolio

Peer Review File

Supramolecular dye polymers for aggregation-induced photocatalysis

Corresponding Author: Professor Luka Đorđević

This file contains all reviewer reports in order by version, followed by all author rebuttals in order by version.

Version 1:

Reviewer comments:

Reviewer #1

(Comments for the Author)
This resesarch investigates organic photocatalysis for the formation of hydrogen and hydrogen peroxide. Much thorough work has been done, and it is an excellent body of research of very high quality.

Generally, the work is well performed and reported, but overall, in my view, the work is similar conceptually to previous work in other systems, and while the effects that are observed are of interest to the scientific community, they are not as novel as they are made out to be. More careful mention of previous work should have been made to put the research in proper context.

There are many works that show enhancement of reactive oxygen species by immobilization of chromophores leading to reduced intramolecular motion and deactivation (to cite just two that came up in a simple search: DOI: 10.1007/s43630-023-00502-5 and DOI: 10.1016/j.xcrp.2020.100030. In the specific case here, the cited reference 24 coincides with the conceptual approach of this work (the term aggregation-induced photocatalysis was used in the paper), and therefore, in my opinion, the originality and significance are overstated. It is already known that "photocatalytic activity emerges from localized, individualized singlet excited states".

The authors state that " not only aggregation, but also the pathway to crystalline assemblies dictate the resulting optoelectronic properties and photocatalytic outcomes". Yet, before that, they acknowledge that the crystal structures of the aggregates are identical, and it is domain size and general dimensions of the crystals that differ. The catalytic effects are given on a mole basis, but, obviously, the surface area of the systems is very different. It is well known that kinetically-grown materials are smaller and less crystallographically ordered than more thermodynamically-grown materials. Rather than kinetic or thermodynamic aggregates, it appears that it is simply the dimensions of the aggregate that are dominant.

The effect of counter-ion is nice, but is surely a result of changes in the crystal structure of the materials.

The paper is very well presented, but contains a lot of jargon that doesn't help understanding and might even be wrong, for example "hierarchical nanocrystals". Where is the hierarchy in the system that is presented here? The molecules are dissolved or assembled in nanocrystals as far as I could tell. Another case is " the molecular packing elucidated through electron diffraction and morphology confirmed across multiple length scales.", that sounds very grand, but in fact the molecular packing is not considered over multiple legth scales, it is simply (unless I misunderstood) the molecular packing. Disassembly of the supramolecular polymer simply means solubilization.

Reviewer #2

(Comments for the Author)
This manuscript prepared by L. Đorđević and co-workers introduces the pioneering concept of Aggregation-Induced Photocatalysis (AIP), a mechanism that fundamentally diverges from established paradigms. Rather than relying on long-range exciton delocalization or charge separation, the authors demonstrate that supramolecular aggregation enhances

photocatalytic efficiency through rigidification of individual monomers. This restriction of intramolecular motion suppresses non-radiative decay, thereby stabilizing localized singlet excited states and increasing their population for redox reactions. Using an amphiphilic distyrylanthracene (DSA^{2+}) derivative, the study convincingly shows that aggregation—triggered by concentration, ionic strength, or counterion exchange—simultaneously enhances fluorescence and drives efficient light-driven production of H_2O_2 and H_2. The work is exceptionally comprehensive, integrating photophysical (steady-state and time-resolved fluorescence), electrochemical, morphological (TEM, AFM, SEM), structural (3D ED), and catalytic analyses, alongside detailed mechanistic investigations (EPR, scavenger assays). The rigorous experimental design, supported by meticulous controls and a logically structured narrative, provides a compelling case for the proposed AIP mechanism. This study is poised to inspire new research directions in controlling aggregation pathways and leveraging AIE effects for photocatalysis. I recommend publication in Nature Chemistry after minor revisions addressing the following points:

1. While strong evidence against triplet-state involvement is provided, have direct experiments such as singlet oxygen sensitization or ultrafast transient absorption spectroscopy been performed to positively confirm the singlet state as the direct electron donor and exclude any ultrafast intersystem crossing to a short-lived triplet?
2. The correlation between increased fluorescence quantum yield and catalytic activity is clear. Could a more quantitative analysis be provided—e.g., does the catalytic rate scale linearly with the calculated excited-state population across different aggregation conditions (counterion, concentration, salt)?
3. The superior performance of kinetically trapped aggregates is attributed to higher excited-state availability from stacking faults. Could their smaller size and higher surface-to-volume ratio—and thus improved mass transport and substrate accessibility—also contribute significantly to the enhanced reaction rates?
4. Have the long-term (e.g., multi-day) photostability and recyclability of kinetic aggregates been assessed? Is there a risk of restructuring into less active thermodynamic forms during prolonged use?
5. The generalization of AIP to a TPE derivative is compelling. What specific molecular features are deemed essential for AIP? Would a fully rigid, planar chromophore—lacking conformational flexibility—still benefit from this strategy?
6. Beyond initiating aggregation via the reverse Hofmeister effect, do counterions (e.g., I^-, PF_6^-) remain associated within the aggregate and influence the excited-state electronic structure or facilitate electron transfer?
7. Was wavelength-dependent reactivity observed? Such data could indicate whether catalytic activity is associated with specific vibronic transitions or aggregate species with distinct energies.
8. With an apparent quantum yield of 0.2% for H_2 evolution, what is identified as the primary efficiency-limiting factor—e.g., intrinsic electron transfer rate, back-electron transfer, sacrificial donor regeneration, or the co-catalyst interface?
9. Several mass spectra in the Supporting Information lack analysis, and some NMR spectra (e.g., Fig. S11, S24, S26) show impurity peaks. Purified spectra may be provided. Additionally, why does the NMR spectrum in Fig. S16 show two singlets? Could this indicate residual starting material?
10. Some recently published related papers may be of reference value, e.g., 10.1002/agt2.364, 10.1038/s41467-024-55060-w, 10.1007/s11426-025-2780-x, 10.1002/agt2.676.

Reviewer #3

(Comments for the Author)
This manuscript presents an interesting study on aggregation-induced photocatalysis (AIP), in which supramolecular dye polymerization rigidifies chromophores to stabilize localized singlet excited states and thereby enhances light-driven reactivity. The work integrates the molecular design with comprehensive spectroscopic, microscopic, and theoretical analyses, providing a new mechanistic perspective that contrasts exciton delocalization with excited-state confinement. While this manuscript is well organized, but the current data is not strong enough, and thus the authors are highly recommended to conduct more in-depth data collection and analysis. For example, more in-depth kinetic analysis, detailed energy-level mapping, and rigorous comparison with established photocatalytic benchmarks are needed to validate the universality and practical competitiveness of the reported AIP effect.
The authors should specifically address the following points:

1. In Figure 1b, the authors actually used PtNPs as a cocatalyst in the hydrogen evolution experiment, but this was not indicated in the figure, which may cause misunderstanding. It is recommended that the authors redesign the figure. Could the authors determine and discuss the excited-state redox potentials of the aggregated dye systems (both oxidation and reduction potentials) and compare them with the thermodynamic potentials for H^+/H_2 and O_2/H_2O_2 (or O_2/H_2O) couples? This comparison would clarify whether the observed photocatalytic reactions are energetically feasible and further substantiate the proposed electron-transfer mechanism.
2. The manuscript describes that aggregation increases fluorescence lifetime and simultaneously enhances photocatalytic activity; however, in some cases (e.g., higher DSAI concentration or increased NaI), the fluorescence lifetime decreases while activity rises. Could the authors clarify this apparent inconsistency? It raises questions about whether the observed enhancement truly originates from “excited-state stabilization” or from other factors such as intermolecular charge transfer or scattering effects in the aggregates.
3. The authors emphasize that the iodide salt (DSAI) is the only sample exhibiting concentration-dependent aggregation and emission behavior. The reviewer would like to know why the addition of extra NaI leads to further aggregation of DSAI. Could the authors explain the underlying mechanism? Similarly, for compounds such as DSABr that hardly aggregate, would the addition of excess NaBr induce aggregation?
4. Compared with DSAI, DSAPF6 exhibits better aggregation and superior photocatalytic performance without the need for additional NaI. However, the authors mainly focus on DSAI in this work. Could they explain the reason for this choice?
5. The authors measured the reduction potentials of the monomer and the aggregates; however, according to the cyclic voltammetry results (Figure S65), DSAI shows poor redox reversibility both in solution and in the solid state. How do the authors explain its excellent photocatalytic performance?
6. The authors claim that DSAI exhibits nearly identical redox and photoexcited-state energy levels between the monomeric

and aggregated states. This characteristic is rather uncommon—are there any known compounds with similar behavior?
7. Could the authors perform femtosecond/picosecond transient absorption or time-resolved fluorescence measurements to directly track the excited-state dynamics of the monomeric and aggregated species? Such data would be essential to substantiate the proposed mechanism of localized singlet-state stabilization and suppressed non-radiative decay.
8. Could the authors evaluate the generality of the photocatalytic behavior by testing additional substrates or redox reactions beyond H2 and H2O2 generation (e.g., organic transformations or alternative electron acceptors/donors)? Demonstrating catalytic versatility would strengthen the claim that aggregation-induced photocatalysis represents a broadly applicable design principle rather than a system-specific phenomenon.

Reviewer #4

(Comments for the Author)

1. Summary of the key results
Dorđević et al. present an interesting study on photocatalysis triggered by the aggregation of certain supramolecular dye polymers.The authors propose that the photocatalytic performance induced by aggregation is not due to the well-established electronic coupling or altered redox potentials of dyes within the aggregates. Instead, they suggest that it is attributable to localized emissive singlet excited states, facilitated by the rigidification of the dye framework, imposed by packing forces.

2. Originality and significance: if not novel, please include reference
The novelty of this work lies in its provision of new insights into aggregation-induced photocatalysis phenomena, suggesting a clear departure from the commonly established supramolecular photocatalysis via exciton delocalization and long-range charge separation. The authors conducted a thorough investigation into the relevant processes and mechanisms, which holds significant promise for advancing the field of supramolecular photocatalysis.

3. Data & methodology: validity of approach, quality of data, quality of presentation
High

4. Appropriate use of statistics and treatment of uncertainties
Yes

5. Conclusions: robustness, validity, reliability
In part. There are key areas where further improvement and exploration are warranted (see bellow; 6. Suggested improvements).

6. Suggested improvements: experiments, data for possible revision

a) (page 11; last paragraph): The time-correlated single photon counting (TCSPC) experiments (see also Fig. S56-S58) appear to be quite confusing and contradictory to the proposed hypothesis. While aggregation of the DSAI dye caused by charge-diffuse anions extended (albeit slightly) the singlet-state average lifetime (τavg), the opposite trend was observed when aggregation was triggered by increasing either the concentration of DSAI or ionic strength (addition of NaI). One would expect that stabilization of the excited-state by rigidification (i.e., suppression of non-radiative decay) will invariably result in an increase of τavg. As demonstrated by the clear visual indication (see Fig. S56-S57), a rapidly decaying component in proximity to the pulse profile manifests upon aggregation. This may suggest the existence of an additional reaction channel (e.g., charge delocalization) that depopulates rapidly the excited state, thereby challenging the conclusion that the system operates through localized singlet excited states stabilized by rigidification. It is imperative for the authors to provide a comprehensive analysis of the multiexponential fluorescence decay curves (contribution analysis of all lifetimes to the τavg).

b) (Page 24 conclusion part): The primary conclusion of this study is that the observed aggregation-induced photocatalysis is based on localized excited-state reactivity, rather than extended electronic coupling between the arranged dyes. A thorough examination, however, of the crystal structure of DSAI aggregates discloses a centre-to-centre distance between dye rings of 5.67 Å with a slip angle of θslip = 44.6(0). This molecular arrangement exhibits characteristics consistent with slip-stacked packing featuring mixed electronic states, in which J-type exciton coupling predominates - resulting in increased emission. Therefore, the possibility of mixed electronic states involving both coulombic and charge transfer coupling cannot be ruled out. To ensure the validity of the primary conclusion of this study, it is essential to investigate whether DSAI's photoinduced radical signatures can be directly detected using Transient Absorption Spectroscopy, as these signatures may contribute to or be responsible for triggering the photocatalytic reactions presented.

c) The authors posit that aggregation has no effect on excited-state energies or redox potentials, and that dyes behave independently of each other (i.e. there is no coupling), operating through localized singlet excited states stabilized by rigidification. In other words, the only beneficial function of aggregation is the rigidification of the dye framework, imposed by the packing forces. If this is true, it may be possible to hypothesize that a synthetic modification that renders the monomer (e.g., DSAI) more planar would maintain its aggregation-induced photocatalytic performance without requiring aggregation. In my opinion, this question doesn’t need to be fully explained at this moment, but it deserves to be discussed.

d) Page 1 last paragraph: Remove “that” from “confirming that the generality…”

e) Figure 5d caption: Add “in the presence of co-catalyst PtNPs”.

7. References: appropriate credit to previous work?
Yes

8. Clarity and context: lucidity of abstract/summary, appropriateness of abstract, introduction and conclusions
Yes

Version 2:

Reviewer comments:

Reviewer #1

(Comments for the Author)
After reading thoroughly the thorough and extensive replies to the referees and the modified manuscript, the paper states more clearly the novelty, and has provided additional evidence to allay doubt concerning certain aspects of the work. The research seems robust, valid and reliable.
All criticisms to the original submission have been addressed.

Reviewer #2

(Comments for the Author)
The authors have adequately revised their manuscript according to my previous comments and suggestions. The quality of the manuscript has been improved after the revision. The revised manuscript is recommended for publication in its present form.

Reviewer #3

(Comments for the Author)
The authors have made a substantial effort to address the concerns raised in the previous review and have significantly expanded the experimental and mechanistic scope of the manuscript. In particular, the inclusion of excited-state redox energetics, transient absorption spectroscopy (TAS), wavelength-dependent photocatalysis, and additional aggregation-control experiments has strengthened the mechanistic framework of aggregation-induced photocatalysis (AIP).
Nevertheless, while many of the reviewer’s questions are now addressed at a qualitative level, several key points require further refinement to confirm the mechanistic robustness and conceptual consolidation of the work.
I therefore recommend minor revision before the manuscript can be considered for publication in Nature Chemistry:
1. While the authors now attribute the apparent decoupling between fluorescence lifetime and photocatalytic activity to singlet–singlet annihilation and triplet-state participation, the mechanistic picture remains largely qualitative. It would be important to clarify, at least semi-quantitatively, which excited state (singlet vs triplet) dominates the productive electron-transfer step under catalytic conditions, and how this correlates with the observed reaction rates.
2. The authors justified the focus on DSAI due to its concentration-dependent assembly. However, to establish AIP as a general design principle, it is important to clarify if the mechanistic conclusions derived from salt-induced DSAI aggregates (kinetic traps) are fully transferable to spontaneously aggregating systems like DSAPF6. A brief comparative discussion on whether the "excited-state confinement" mechanism remains consistent across different counterion-induced packing motifs would be beneficial.
3. The contrast between the poor redox reversibility in cyclic voltammetry and the high photocatalytic activity remains a point of curiosity. While the authors argue that sacrificial donors "save" the oxidized chromophore, this suggests the system's robustness is highly dependent on donor kinetics. The authors should discuss whether the structural reorganization or degradation pathways inferred from the CV results could limit the catalyst's long-term recyclability in more demanding or donor-limited environments.

Reviewer #4

(Comments for the Author)
In the revised manuscript, the authors provide thorough and convincing responses to the initial comments raised. I am therefore satisfied that the work meets the publication standards of Nature Chemistry in its current form.

Version 3:

Reviewer comments:

Reviewer #3

(Comments for the Author)
I think that the authors have adequately addressed the comments made by the reviewers in the revised version of the manuscript. Therefore, I have no further comments.

Open Access This Peer Review File is licensed under a Creative Commons Attribution 4.0 International License, which permits use, sharing, adaptation, distribution and reproduction in any medium or format, as long as you give appropriate credit to the original author(s) and the source, provide a link to the Creative Commons license, and indicate if changes were made.
In cases where reviewers are anonymous, credit should be given to 'Anonymous Referee' and the source.
The images or other third party material in this Peer Review File are included in the article's Creative Commons license, unless indicated otherwise in a credit line to the material. If material is not included in the article's Creative Commons license and your intended use is not permitted by statutory regulation or exceeds the permitted use, you will need to obtain permission directly from the copyright holder.
To view a copy of this license, visit https://creativecommons.org/licenses/by/4.0/

Re: NCHEM-25093064B
Point-by-point response to the reviewer's comments

Reviewer #1

This research investigates organic photocatalysis for the formation of hydrogen and hydrogen peroxide. Much thorough work has been done, and it is an excellent body of research of very high quality.
Generally, the work is well performed and reported, but overall, in my view, the work is similar conceptually to previous work in other systems, and while the effects that are observed are of interest to the scientific community, they are not as novel as they are made out to be. More careful mention of previous work should have been made to put the research in proper context.

Answer: We thank the reviewer for commenting positively on the amount and quality of our work, while raising potential concerns on this manuscript. We take them very seriously, but we nevertheless feel that there might be some misunderstandings regarding the significance of this work. Importantly, we acknowledged in our original manuscript that certain organic molecules as aggregates exhibit photocatalytic properties; however, it's crucial to note that our underlying mechanism differs from previous works, and it is on the novelty and generality that we want to place our emphasis. In the following, we provide concrete responses to the comments and suggestions point-by-point.

Comment: *1. There are many works that show enhancement of reactive oxygen species by immobilization of chromophores leading to reduced intramolecular motion and deactivation (to cite just two that came up in a simple search: DOI: 10.1007/s43630-023-00502-5 and DOI: 10.1016/j.xcrp.2020.100030. In the specific case here, the cited reference 24 coincides with the conceptual approach of this work (the term aggregation-induced photocatalysis was used in the paper), and therefore, in my opinion, the originality and significance are overstated. It is already known that "photocatalytic activity emerges from localized, individualized singlet excited states".*

Answer: We thank the referee for their comment and for the chance to highlight the novelty of our manuscript. As the referee correctly points out, the term "aggregation-induced photocatalytic activity" has been used before, and we have indeed cited this work in our original manuscript. Kida and colleagues (reference 24 in the original manuscript, reference 26 in the revised version) reported that a self-assembled rhodamine (carrying C18 alkyl chain, RhB-C18) undergoes aggregation-caused quenching (ACQ) and is more active toward photocatalytic H_2 evolution when compared to the non assembled derivative (RhB-C2, carrying a C2 alkyl chain). **ACQ, rather than AIE**, was attributed to the increased photocatalytic activity and the authors hypothesized that **excited state quenching is due to charge separation** and more efficient **charge migration**. In our work, we report the opposite and correlate aggregation-induced emission with aggregation-induced photocatalysis. We therefore put forward that the term of "aggregation-induced photocatalysis (AIP)" is better suited. The work by Kida et al. is suitably cited together with other dyes that exhibit aggregation-induced quenching and photocatalysis (in the paragraph starting with "While aggregation is often detrimental to photocatalysis, it can also be beneficial for some organic dyes.[12,24-27]").
The other articles mentioned by the referee, while interesting, do not make a point of correlating increased excited state availability with photocatalysis. Specifically, Amara and co-workers[1] state that the immobilization of cationic photosensitizers on silica nanoparticles decreases the fluorescence emission of the former. It was hypothesized that the enhanced $^1O_2$-mediated photooxidation is due to the proximity between polar substrate and the surface of silica particles. Garcìa and co-workers[2] disclose an anionic porphyrin (TCPP) incorporated within the nanofibers of a gelator for enhanced $^1O_2$ generation. They report an increase in porphyrin fluorescence emission

due to a reduction in the degree of aggregation in the gel, however we note that the absorbances at the excitation point are different in solution and gel states and the emission quantum yields are not reported.
To summarize, the conceptual approach of our work differs from previous ones, as it is specifically focused on aggregation-induced photocatalysis rather than effects (i.e. charge delocalization and exciton transport) of aggregation-caused quenching contributing to photocatalysis. Importantly, we also observe the same phenomenon for the TPE molecule, structurally different from the DSA chromophore. The significance and novelty of this work therefore rely on demonstrating a supramolecular strategy to promote photocatalysis through localized excited states.

Revision: We revised our manuscript accordingly in the Introduction section. In the third paragraph, in the discussion about self-assembled chromophore for photocatalysis, we added the following: "In these photocatalytic systems, an aggregation-caused quenching (ACQ) phenomenon is observed, but is not the only requisite for photocatalysis. Rather, the organic nanostructures were shown to drive key reactions in artificial photosynthesis, including H_2 evolution and H_2O_2 production, when emission quenching leads to exciton migration and charge separation along the crystalline domains."
We also added the reference suggested by the referee in the second paragraph: "As a result, the excited-state is typically deactivated quickly in the aggregated state. Strategies to overcome this and exploit molecules in photocatalysis include preparation of metallacages,[16] macrocycles,[17] using surfactants,[18] formation of inclusion complexes,[19] and co-assembling dyes with insulating (macro)molecules.[20-23]"
Finally, we modified last paragraph of the introduction to highlight the novelty of our work: "Unlike other self-assembled crystalline photocatalysts, where aggregation-caused quenching enables charge delocalization and exciton transport, our system operates through localized excited states, stabilized by rigidification."

Comment: *2. The authors state that " not only aggregation, but also the pathway to crystalline assemblies dictate the resulting optoelectronic properties and photocatalytic outcomes". Yet, before that, they acknowledge that the crystal structures of the aggregates are identical, and it is domain size and general dimensions of the crystals that differ. The catalytic effects are given on a mole basis, but, obviously, the surface area of the systems is very different. It is well known that kinetically-grown materials are smaller and less crystallographically ordered than more thermodynamically-grown materials. Rather than kinetic or thermodynamic aggregates, it appears that it is simply the dimensions of the aggregate that are dominant.*

Answer: We thank the referee for their insightful comment. We performed further experiments to evaluate the impact of crystals size on photocatalysis. In one case we used $DSAPF_6$, in which two different procedures could be easily exploited to prepare crystals with different sizes due to its complete insolubility in water, and in the other case we used DSAI, in which annealing could be exploited to prepare crystals with tunable sizes.
First, we prepared $DSAPF_6$ crystals by two different methods: sonication or nanoprecipitation. Smaller crystals (from nanoprecipitation compared to sonication) lead to improved photocatalytic activity (both for TMB oxidation and for H_2 evolution). However, also the photophysical properties differ (longer lifetimes, higher fluorescence quantum yield, and higher photocatalytic performance toward both TMB oxidation and H_2 evolution for the nanoprecipitated crystals) (Fig. S124 for details).
Second, DSAI was annealed and the samples were stirred at different speeds (in presence of stir bars) during the slow-cooling phase to obtain crystalline samples with different sizes. The different sizes of the obtained crystals (at 800, 400, 200 or 0 rpm) were imaged by fluorescence microscopy (Figure R1a-d). These crystals were then used for photocatalytic H_2 evolution, which showed a clear trend with smaller sizes leading to higher photocatalytic activity (Figure R1e). While size plays an

important role, we also note that aggregates with different dimensions also possess different photophysical properties. For example, in the original manuscript we reported that kinetically trapped aggregates were smaller than their thermodynamic counterparts, but also their excited state availability differed (6% versus 14%, respectively).
In summary, while the referee is correct in pointing out that crystal size is important in photocatalytic activity, we show here that it is not the only contributing factor nor the dominant one, which makes appropriate to report the catalytic effect on a mole basis. Importantly, we note that in crystalline organic materials reported so far[4–6] more extended aggregates lead to better charge separation and improved photocatalytic activity. We are therefore excited to see how controlled nanostructure growth might be used to tune the photophysical and photocatalytic activities of self-assembled nanocrystals, although a detailed investigation of this lies outside the scope of the present manuscript. Nevertheless, the interplay between size and excited-state dynamics demonstrated here offers various design possibilities to exploit our new conceptual approach.

Revision: We have revised our manuscript by attributing the reasons for the difference in activity between kinetically trapped and thermodynamic aggregates to size and excited-state availability, by removing "changing stacking fault disorder, defects". We also added these lines to the main text: "To further examine how crystal size influences photocatalysis, $DSAPF_6$ aggregates were prepared using either sonication or nanoprecipitation. As observed above, the smaller crystals (generated by nanoprecipitation) displayed enhanced photocatalytic activity. However, they also showed distinct photophysical behavior, including greater excited-state availability (Supplementary Fig. S124), indicating a complex interplay between size and excited-state dynamics that governs the photocatalytic performance."

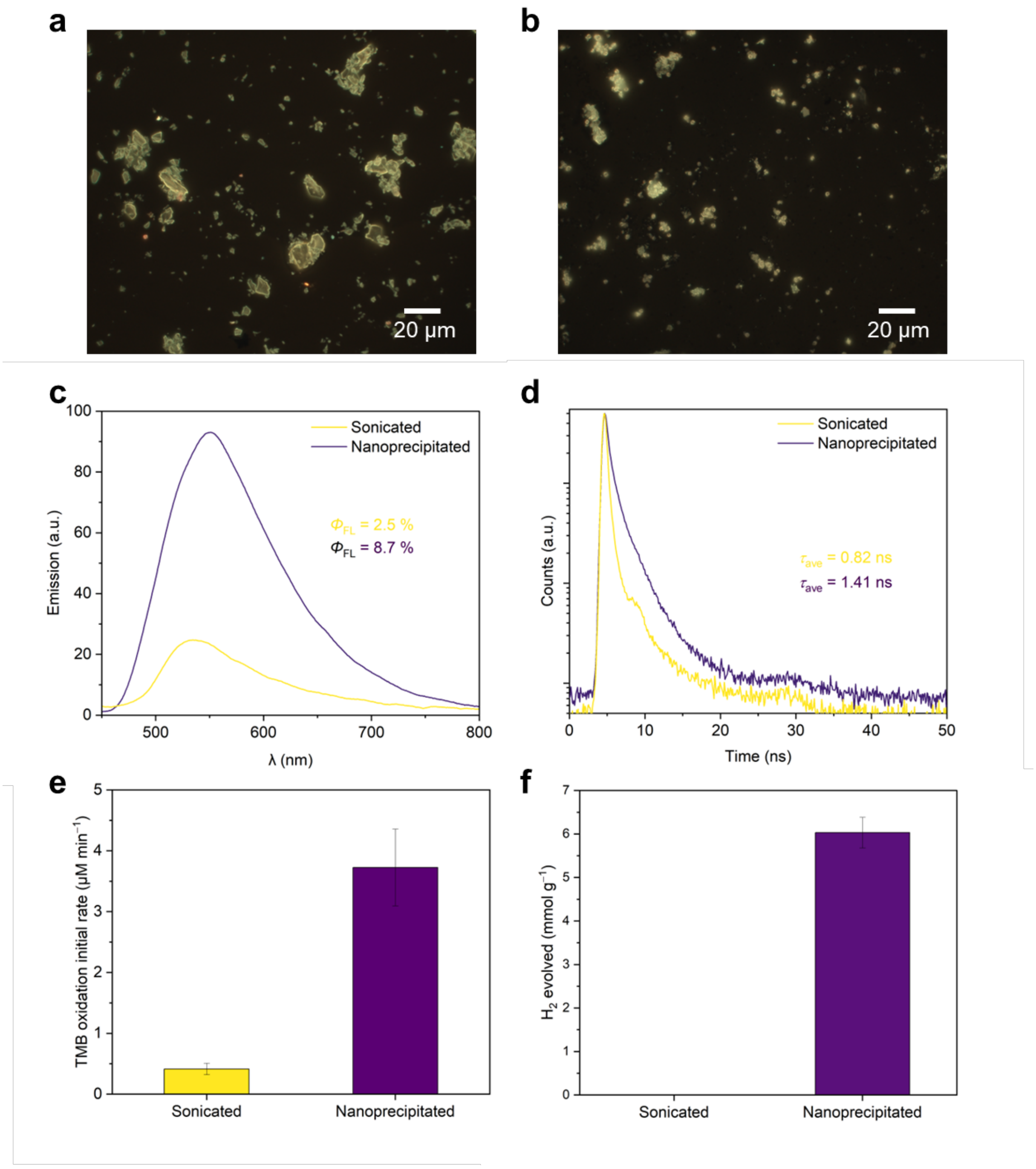

Figure S124. Widefield fluorescence images of $DSAPF_6$ 0.4 mg mL^{-1}. a) sonicated in mQ-water and b) nanoprecipitated from DMSO in mQ-water. c) Fluorescence emission spectra and fluorescence quantum yields (Φ_{FL}) of $DSAPF_6$ 0.4 mg mL^{-1}sonicated *vs* nanoprecipitated in acetate buffer (0.1 M, pH = 5.0) containing 3.2% DMSO (*v/v*). d) Time-correlated single-photon counting (TCSPC) decay traces of the same samples. e) TMB oxidation rate (0.41, 3.7 μM min^{-1}) of same samples under white light (180 mW cm^{-2}). f) H_2 evolution produced by $DSAPF_6$ 0.4 mg mL^{-1} in ascorbic acid (1.0 M, pH = 4.0) sonicated *vs* nanoprecipitated from DMSO, in presence of PtNPs (8% mol) under 4 hours white light irradiation (100 mW cm^{-2}).

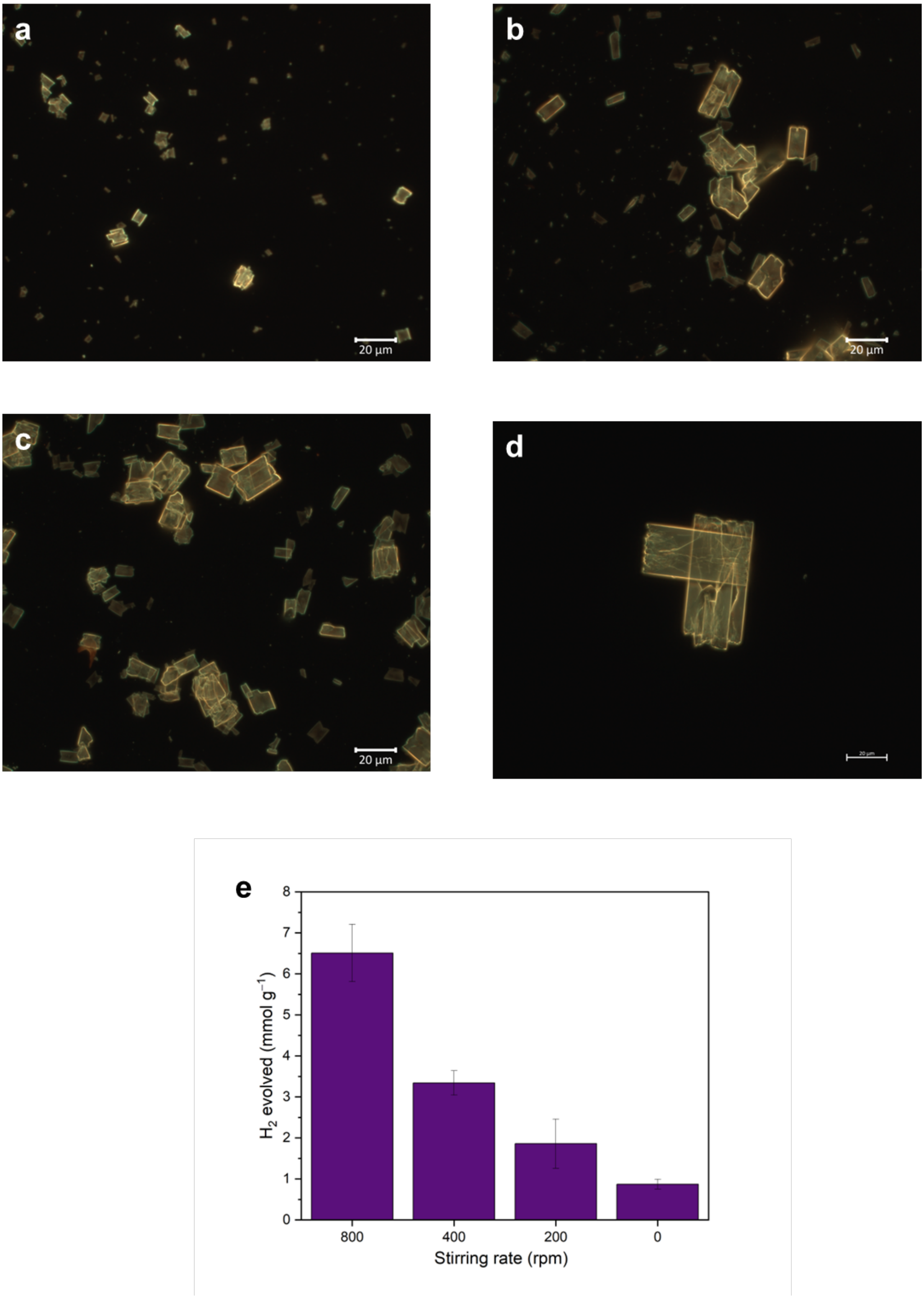

Figure R1. a) Widefield fluorescence image of DSAI 0.1 mM annealed and slowly cooled in presence of a stirring bar at 800 rpm, b) 400 rpm, c) 200 rpm and 0 rpm; e) H_2 evolution photosensitized by DSAI thermodynamic crystals obtained under different stirring conditions (800, 400, 200, and 0 rpm).

Comment: *3. The effect of counter-ion is nice, but is surely a result of changes in the crystal structure of the materials.*

Answer: We thank the reviewer for this comment. We agree that different counterions are indeed expected to affect to different extent (depending on their size, shape, and charge density) the resulting crystal structures, the associated molecular packing, the network and strength of intermolecular interactions and, hence, the physico-chemical properties of the final crystalline materials. However, as suggested also by experiments reported above, it is relevant how the anions influence the crystals size, which, in turn, determines different photophysical and photocatalytic properties.

To assess the structural effects of the different counterions on the final crystal structures in our work, we compared PXRD patterns of all the salts analyzed, confirming that different anions impact the resulting crystalline structures (Fig. S54 in the revised manuscript). As expected, the halides display closely related diffraction features, while salts based on PF_6^- and NO_3^- have markedly different diffraction patterns.

Moreover, it is crucial to note that in our molecular compounds, the role of the different anions goes well beyond merely inducing (expected) changes in the crystal structure. Indeed, even if DSACl, DSABr and DSAI show similar solid state structures, they lead to very different behaviors when the solid is added to a water solution. In particular, Br^- and Cl^- help with the solubilization of the monomer while I^- does not. Our interest was indeed mainly on how the aggregation is dependent on charge-screening, which ultimately lead to different emission properties (Fig. 2a, Supporting Figs. 33-34) and different photocatalytic activities. The choice of I^- was not guided by structural considerations, but rather by its ability (unique in the series of anions investigated) to modulate the aggregation pathways, and the associated aggregation-induced rigidification, through specific concentration-dependent ion effects, see Figures 2 and 3 in the main text and relative discussion.

Revision: In response to this comment, we have revised the main text in the following way. We have modified the following sentence to better explain the choice of using the DSAI to explore the assembly-photocatalysis landscape: "Among the DSAX salts, only the iodide form (DSAI) exhibited concentration-dependent aggregation and emission behavior, appearing weakly emissive in dilute solutions and forming progressively fluorescent yellow suspensions at higher concentrations (Fig. 2a and Supplementary Figs. S35-S39)".

Then, to better clarify the points above, we have modified the following sentence in the main text: "We further investigated the polymerization behavior of DSAI, which can exist either as molecularly dissolved species or as aggregates depending on concentration."

Importantly, we have also added the following section to the manuscript, in which we discuss and clarify the effect of the nature of the counteranion on the aggregates: "Increasing the ionic strength of the solution through salt addition screens the positive charges on the molecules, thereby reducing electrostatic repulsion and promoting hydrophobic collapse, ultimately leading to aggregation. Consistent with this mechanism, aggregation of DSAX (X = Cl^-, Br^-, NO_3^-, and I^-) can be induced by addition of the corresponding sodium salts (NaX), which increases charge screening and leads to formation of aggregates (Supplementary Figs. S44-S46). Importantly, aggregation can be induced by adding inorganic salts with counteranions different from those originally present with the dye, enabling solvated molecules to aggregate. For example, DSACl undergoes aggregation upon addition of NaI (Supplementary Figs. S47-S48), demonstrating that both ionic strength and counteranion identity contribute to aggregation. Supporting this interpretation, we observed that counteranions are not merely dissociated but remain localized within the aggregates, confirming they are embedded within them and contribute to the overall aggregation (Supplementary Figs. S48-S54)."

In the Supporting Information we added Supplementary Figure S54:

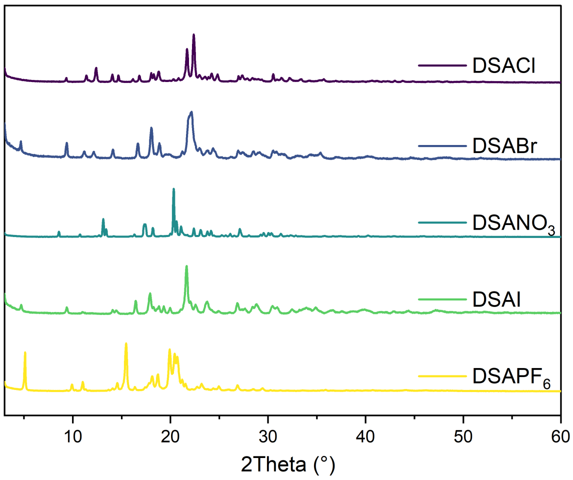

Figure S54. PXRD diffraction patterns of DSACl (purple trace), DSABr (blue trace), $DSANO_3$ (light blue trace), DSAI (green trace), and $DSAPF_6$ (yellow trace).

Comment: *4. The paper is very well presented, but contains a lot of jargon that doesn't help understanding and might even be wrong, for example "hierarchical nanocrystals". Where is the hierarchy in the system that is presented here? The molecules are dissolved or assembled in nanocrystals as far as I could tell. Another case is " the molecular packing elucidated through electron diffraction and morphology confirmed across multiple length scales.", that sounds very grand, but in fact the molecular packing is not considered over multiple length scales, it is simply (unless I misunderstood) the molecular packing. Disassembly of the supramolecular polymer simply means solubilization.*

Answer: We thank the reviewer for the suggestions. While our intention was to use a language commonly used in the field of supramolecular chemistry, we agree that it is helpful to make the manuscript more accessible to a general chemistry audience. In the revised manuscript, we refer to our crystalline system only as nanocrystals (rather than hierarchical nanocrystals). Furthermore, we make a point that "across multiple length scales" refers to a full characterization of the structure, which was confirmed and characterized from nano to bulk scale, by using different techniques (TEM, SEM, AFM, widefield fluorescence microscopy, 3D electron diffraction). We also agree that disassembly means solubilization, thus we modified the main text accordingly.

Revision: In the main text, we removed the term "hierarchical" and substituted the term "disassembly" of supramolecular polymer with "solubilization" or "dissolution". Regarding the "multiple length scale" we clarified in following way: "We next characterized the nanostructures formed upon DSAI self-assembly using complementary techniques spanning from the nanoscale to the bulk." and "Supramolecular aggregation of DSA^{2+} in water leads to formation of nanocrystals, whose molecular packing was elucidated through electron diffraction and whose morphology was confirmed using multiple microscopy techniques."

Reviewer #2

This manuscript prepared by L. Đorđević and co-workers introduces the pioneering concept of Aggregation-Induced Photocatalysis (AIP), a mechanism that fundamentally diverges from established paradigms. Rather than relying on long-range exciton delocalization or charge separation, the authors demonstrate that supramolecular aggregation enhances photocatalytic efficiency through rigidification of individual monomers. This restriction of intramolecular motion suppresses non-radiative decay, thereby stabilizing localized singlet excited states and increasing their population for redox reactions. Using an amphiphilic distyrylanthracene (DSA2+) derivative, the study convincingly shows that aggregation—triggered by concentration, ionic strength, or counterion exchange—simultaneously enhances fluorescence and drives efficient light-driven production of H2O2 and H2. The work is exceptionally comprehensive, integrating photophysical (steady-state and time-resolved fluorescence), electrochemical, morphological (TEM, AFM, SEM), structural (3D ED), and catalytic analyses, alongside detailed mechanistic investigations (EPR, scavenger assays). The rigorous experimental design, supported by meticulous controls and a logically structured narrative, provides a compelling case for the proposed AIP mechanism. This study is poised to inspire new research directions in controlling aggregation pathways and leveraging AIE effects for photocatalysis. I recommend publication in Nature Chemistry after minor revisions addressing the following points:

Answer: We are grateful for the reviewer's support and for the positive evaluation of the manuscript. We sincerely thank the reviewer for their constructive comments and suggestions, which have helped us to improve the quality and clarity of the manuscript. Below, we provide point-by-point responses to each comment.

Comment: *1. While strong evidence against triplet-state involvement is provided, have direct experiments such as singlet oxygen sensitization or ultrafast transient absorption spectroscopy been performed to positively confirm the singlet state as the direct electron donor and exclude any ultrafast intersystem crossing to a short-lived triplet?*

Answer: We thank the referee for this insightful comment that prompted us to perform and update the manuscript with direct experiments. In brief, singlet oxygen measurements exclude its involvement, while femtosecond and nanosecond transient absorption (fs-TAS and ns-TAS, respectively) revealed that both localized singlet and triplet excited state of the aggregates are involved in electron transfer to acceptor molecules.

First, we examined the 9,10-anthracenedipropionic acid (ADPA) degradation in presence of DSAI aggregates and molecular oxygen, and we compared the obtained data with $^1O_2$ sensitization operated by Rose Bengal at the same concentration. We did not observe any ADPA degradation with DSAI, while a pronounced decrease in the ADPA absorbance is observed for the samples in presence of Rose Bengal (Fig. S80a-c in the revised manuscript). This strongly suggests that singlet oxygen sensitization is not achieved with DSAI. Furthermore, we did not observe any singlet oxygen emission when a sample of DSAI aggregated in water was irradiated in the presence of oxygen (Fig. S80d in the revised manuscript).

We also investigated the transient absorption spectroscopy of the DSAI both as solution and as aggregate (upon addition of NaI) by fs- and ns-pulsed excitation. We observed that, in solution, the recovery of the ground state occurs with a decay time of 79 ps without formation of a long-lived triplet excited state, while in the case of the aggregates, the formation of a longer-lived triplet excited state is observed with a decay time of 178 ns. The photocatalytic activity of this state has been demonstrated upon the addition of two quenchers, O_2 or methyl viologen. In both cases, in addition to the quenching observed in singlet emission, it was demonstrated that the triplet excited state is also active in photocatalysis. Thus, our new set of experiments confirm that aggregation leads to

localized – singlet and triplet – excited state availability, as also suggested by emission and TAS features of DSAI aggregates that mirrors those reported in literature for distyrylanthracene monomer in a rigid environment, where its intramolecular motion is restricted.[7]

Revision: We updated the main text and Figure 5 (specifically 5g−i):

"In all cases, the fluorescence decays show lifetimes in the nanosecond range, pointing to formation of singlet excited states. This prompted us to further probe the excited states of DSAI solution and aggregates through femtosecond and nanosecond transient absorption (fs-TAS and ns-TAS, respectively) experiments. The monomeric chromophore in solution displayed a band peaked at 620 nm that shifts at 545 nm and then disappeared with lifetimes of 17 ps and 79 ps, respectively, attributed to fast isomerization of the styryl moieties of the DSAI (Supplementary Fig. S71). These results are in agreement with fs-TAS spectra reported for unsubstituted 9,10-distyrylanthracene and derivatives.[54] For DSAI aggregates, instead, fs-TAS showed significative differences: no shift of the excited state absorption (ESA) bands were observed and a kinetic analysis shows a fast component of 21 ps, similar to that previously observed for the monomeric species, but the second component is slower with 320 ps lifetime, as expected by the rigidification of the structure that slows down decays related to photoisomerization pathways and enhances excited state stabilization (Supplementary Fig. S72). At the end of the time range of measurement (7 ns) an ESA band peaked at 539 nm persisted, compatible with the formation of the lowest triplet excited state, whose presence was confirmed in the ns-TAS experiment (Supplementary Fig. S73), although no phosphorescence was observed in solid state measurements (at 77 K, Supplementary Fig. S74). Importantly, the emission and transient absorption spectra of DSAI aggregates mirror those reported for the monomeric dye in rigid environments (Supplementary Fig. S75),[54] further supporting the formation of localized excited states – both singlet and triplet – in the aggregated state. These results show that aggregation enhances photocatalysis by increasing the population of localized excited states. The fact that three distinct aggregation triggers – counterion identity, concentration, and ionic strength – produce parallel trends in both emission and ROS generation supports the observation in which aggregation restricts intramolecular motion and increases the probability of productive excited-state processes."

[…] Additionally, singlet oxygen emission and anthracene-9,10-dipropionic acid photodegradation experiments ruled out singlet oxygen sensitization (Supplementary Fig. S80). Both singlet and triplet excited states of aggregates are involved in electron transfer to O_2 and superoxide formation, as evidenced by quenching of fluorescence emission (Supplementary Fig. S81) and quenching of the triplet state observed by ns-TAS (Supplementary Fig. S73).

[…] Fluorescence experiments revealed that salt-induced aggregation leads to a marked increase in emission with Φ_{FL} going from 0.8% to 14% (Fig. 5g and Supplementary Fig. S102 for excitation-emission maps). Quenching experiments (Fig. 5h,i and Supplementary Fig. S103)[53] showed that the excited states of the aggregated DSAI are quenched in the presence increasing concentration of MV^{2+}, observed both for the singlet (k_{SV}^{S} = 222 M^{-1} from fluorescence titration) and the triplet state (k_{SV}^{T} = 32 M^{-1} from ns-TAS titration), consistent with the involvement in photocatalysis of both localized excited states.

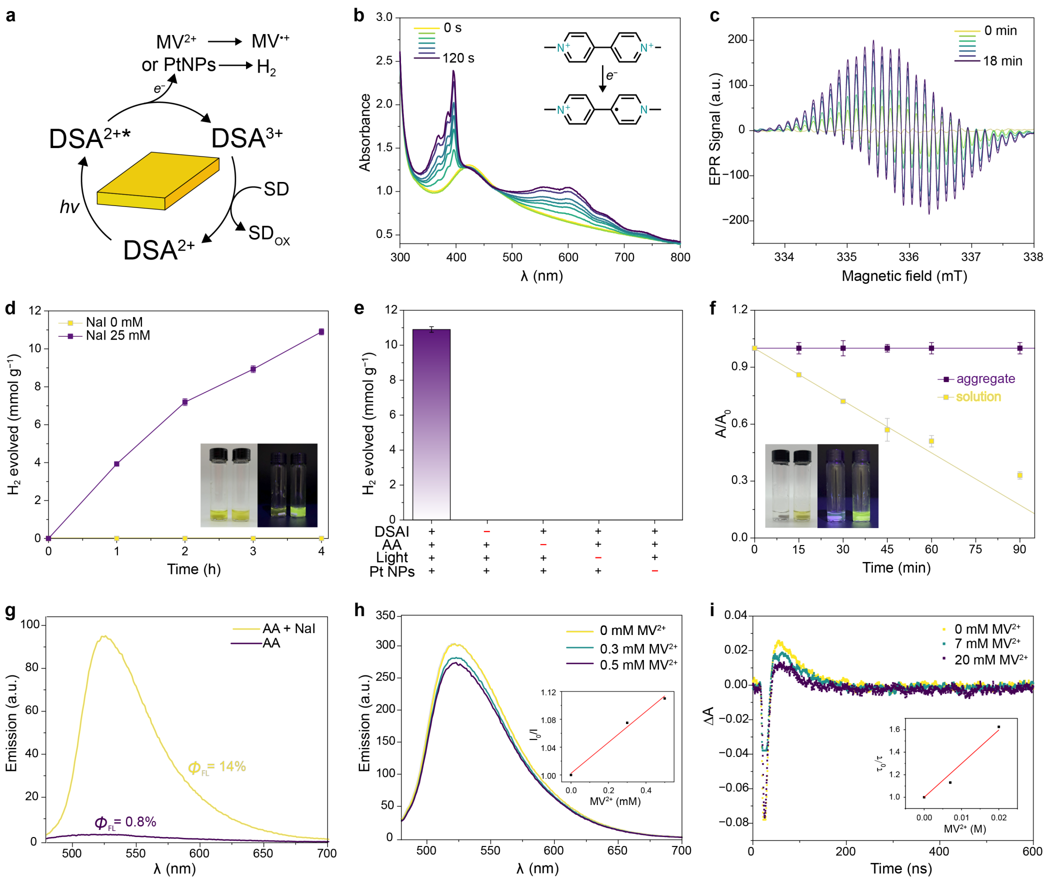

Figure 5. Light-driven hydrogen evolution by DSA^{2+} nanostructures. **a**, Proposed photocatalytic mechanism for methyl viologen (MV^{2+}) or hydrogen evolution in presence of a co-catalyst (PtNPs, platinum nanoparticles) and sacrificial donor (AA, ascorbic acid). **b**, Ultraviolet–visible absorption spectra over time of a mixture of DSAI (1.0 mM) and MV^{2+} (10 mM) under 450 nm (LED, 700 mW cm^{-2}) irradiation. **c**, Electron paramagnetic resonance (EPR) spectra over time of a mixture of DSAI (1.0 mM) and MV^{2+} (10 mM) under 450 nm (LED, 700 mW cm^{-2}) irradiation. **d**, H_2 light-driven evolution over time by irradiation of DSAI solution (yellow trace) or nanostructures (NaI 25 mM, purple trace) in ascorbic acid (1.0 M, pH = 4.0), in presence of PtNPs (8% mol) with white light (LED, 100 mW cm^{-2}). Inset shows DSAI samples in AA without (left) and with (right) NaI under daylight and UV light. **e**, Control experiments for H_2 produced by DSAI. **f**, Absorbance over time by irradiation of DSAI solution or DSAI nanostructures in ascorbic acid. Inset shows irradiated DSAI samples in AA without (left) and with (right) NaI under daylight and UV light. **g,** Emission spectra of DSAI 0.1 mM dissolved in ascorbic acid 1.0 M (pH = 4.0) and aggregated in ascorbic acid 1.0 M (pH = 4.0) with NaI 25 mM (λ_{exc} = 395 nm, at isoabsorbance). **h** DSAI 0.1 mM with NaI 0.1 M emission quenching with different MV^{2+} concentration (λ_{exc} = 470 nm at isoabsorbance). **i** Transient absorption kinetic traces at 420 nm of an aqueous solution of aggregates of DSAI 0.05 mM and 2 mM of NaI (yellow trace) and after addition of Methyl Viologen, [MV^{2+}] = 7 mM (green trace) and 20 mM (purple trace). λ_{ex} = 355 nm. $A_{355\ nm}$ = 0.31, 1 cm optical path, 4.4 mJ per pulse.

The previous Figs. 5g–i have been moved to Supplementary Figs. S102 and S103 in the revised submission.

Regarding the Supporting Information, we added: **Transient absorption spectroscopy.** Transient absorption in the femtosecond range was performed by means of an Ultrafast Systems HELIOS (HE-VIS) femtosecond transient absorption spectrometer by using, as an excitation source, a Coherent Astrella Laser, combined with an OPERA SOLO optical parametric amplifier (pulse width: 32 fs, 1 kHz repetition rate, selected output wavelengths: 420 nm). The Astrella system was composed of a Coherent Vitara™ that includes a modelocked Ti:Sapphire oscillator cavity pumped by the Coherent Verdi™ G-Series, a continuous-wave OPSL (optically pumped semiconductor laser) green laser, a Ti:Sa regenerative amplifier pumped by an intracavity-doubled, a DPSS (diode pumped solid state, REVOLUTION) Q-switched laser with a second-harmonic generator. Operating

at 527 nm and a 1-kHz repetition rate, pump power for the regenerative amplifier module, an optical pulse stretcher, and an optical pulse compressor. The overall temporal resolution of the system is 300 fs. Air-equilibrated solutions in 0.2 cm optical path cells were analysed under continuous stirring. The pump energy on the sample was 4 μJ/pulse at 420 nm. Surface Xplorer V4 software from Ultrafast Systems was used for the data acquisition and analysis. The 3D data surfaces were corrected for the chirp of the probe pulse prior to the analysis. Lifetimes were taken as average of values derived from the fitting of several decays at different wavelengths. Errors on lifetimes were estimated as average of the errors reported by the fitting software for each lifetime. Steady-state absorption spectra were registered before and after transient absorption experiments to check the photostability of the investigated system.
The LP980 setup from Edinburgh Instruments equipped with an Nd:YAG laser from Litron (Nano) was employed for transient absorption and time-resolved emission spectroscopy. The frequency-tripled output with a wavelength of 355 nm served as the excitation source. The laser pulse duration was 7 ns, and the pulse frequency was 5 Hz. The typical pulse energy used for transient absorption and emission studies was 4.4 mJ. Detection of transient absorption spectra occurred by kinetic traces detected at selected wavelengths were recorded using a photomultiplier tube. The spectroscopic experiments were performed at 293 K using a cuvette holder that allows temperature control.

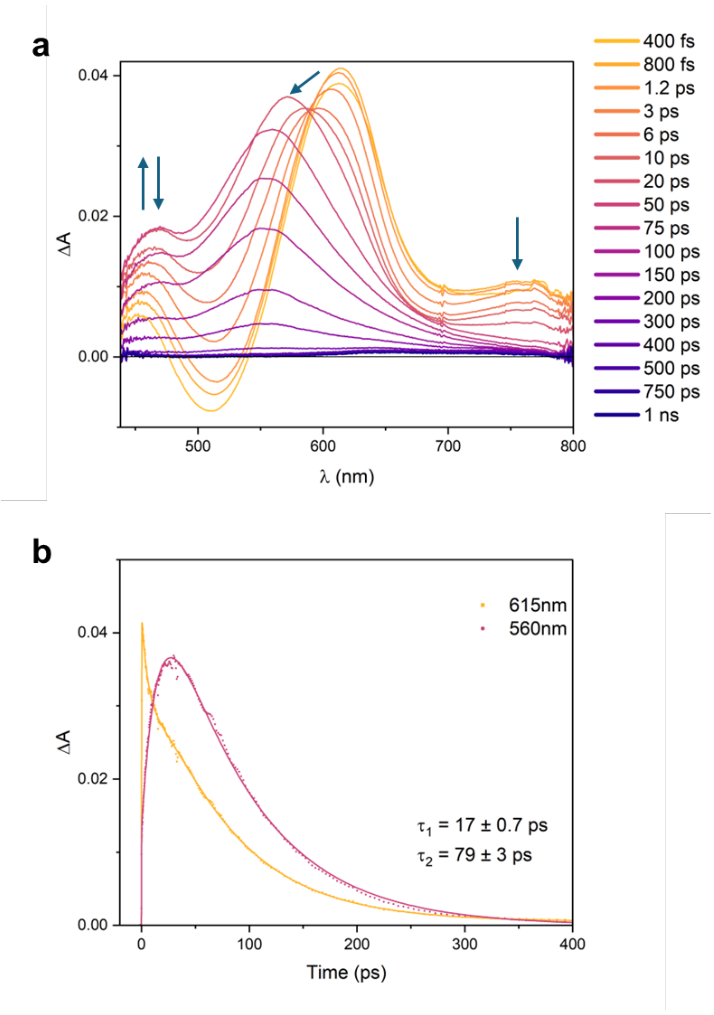

Figure S71. a) Transient absorption spectra at different delays of an aqueous solution of DSAI 0.05 mM. The arrows indicate the spectral changes associated with the different processes. b) Transient absorption kinetic traces at significant wavelengths together with the corresponding fitting decays (solid lines). λ_{exc} = 420 nm. $A_{420\ nm}$ = 0.22, 0.2 cm optical path, 4 μJ per pulse.

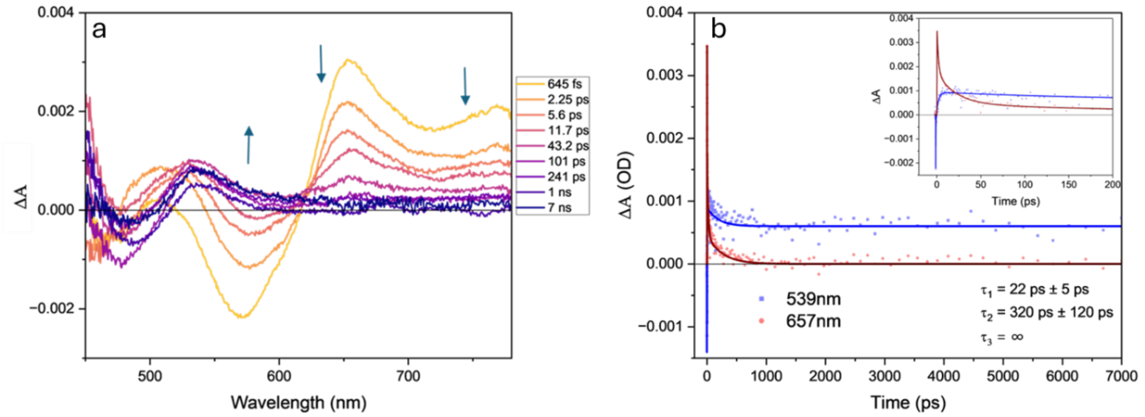

Figure S721. a) Transient absorption spectra at different delays of an aqueous solution of aggregates of DSAI 0.05 mM and 2 mM of NaI. The arrows indicate the spectral changes associated with the different processes. b) Transient absorption kinetic traces at significant wavelengths together with the corresponding fitting decays (solid lines). λ_{exc} = 420 nm. $A_{420\ nm}$ = 0.22, 0.2 cm optical path, 4 μJ per pulse.

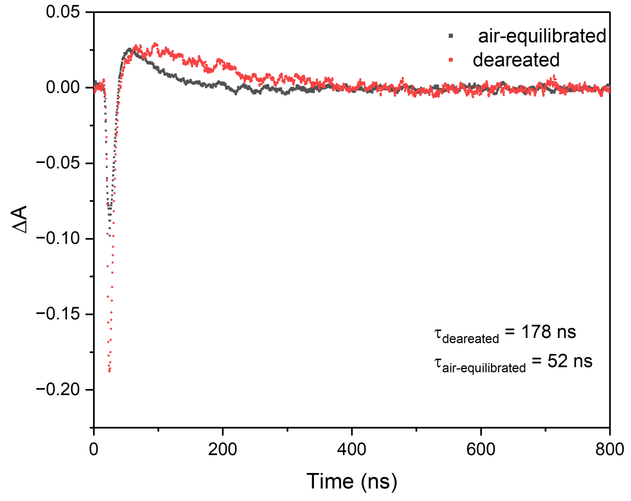

Figure S73. Transient absorption kinetic traces at 420 nm of an aqueous solution of aggregates of DSAI 0.05 mM and 2 mM of NaI in air-equilibrated (black) and deareated (red) condition. λ_{exc-} = 355 nm. $A_{355\ nm}$ = 0.31, 1 cm optical path, 4.4 mJ per pulse.

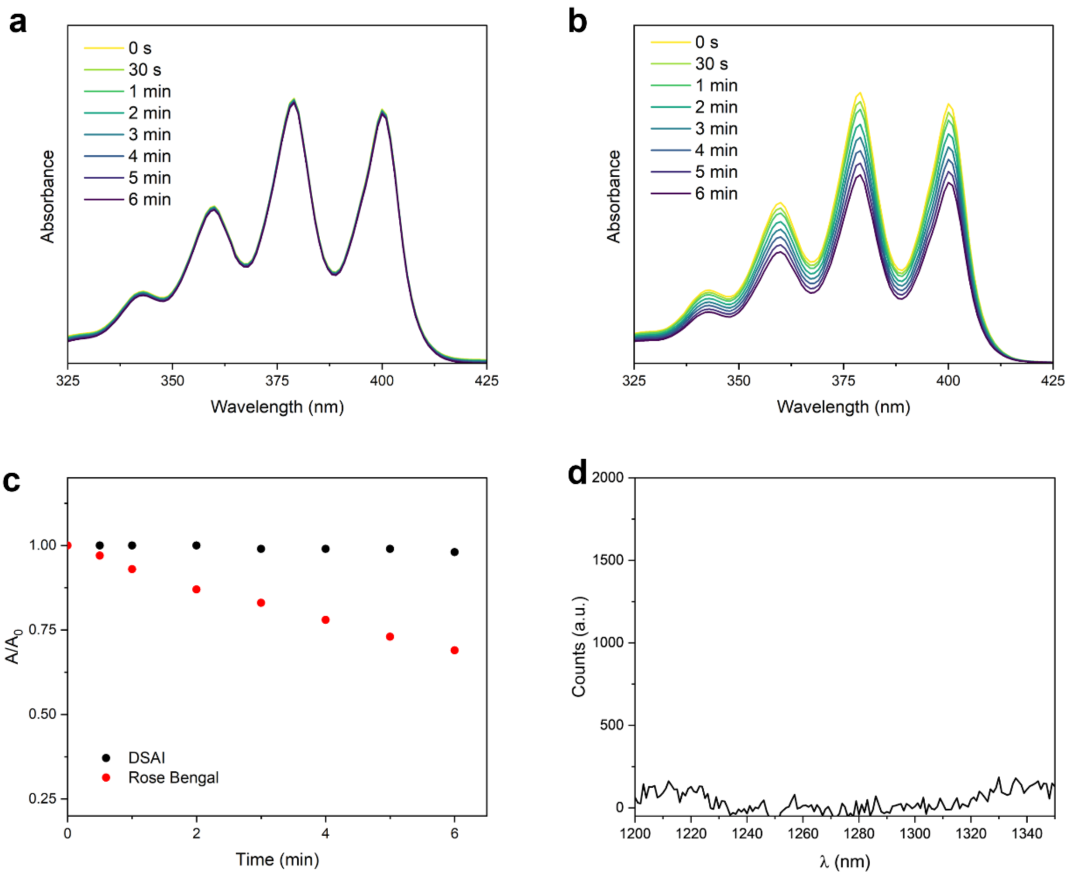

Figure S80. Singlet oxygen sensitization evaluated using 9,10-anthracenedipropionic acid (ADPA) as an indicator. a) ADPA UV-Vis spectra evaluated from 0 to 6 minutes irradiation under white light 28 mW cm^{-2} in presence of DSAI 10 μM, NaI 15 mM, ADPA 110 μM; b) ADPA UV-Vis spectra evaluated from 0 to 6 minutes irradiation under white light 28 mW cm^{-2} in presence of Rose Bengal 10 μM and ADPA 110 μM; c) normalized absorption intensity of ADPA at 378 nm after photodecomposition by singlet oxygen upon white light irradiation. d) Singlet oxygen emission spectrum in presence of DSAI 0.1 mM and NaI 5 mM in D_2O.

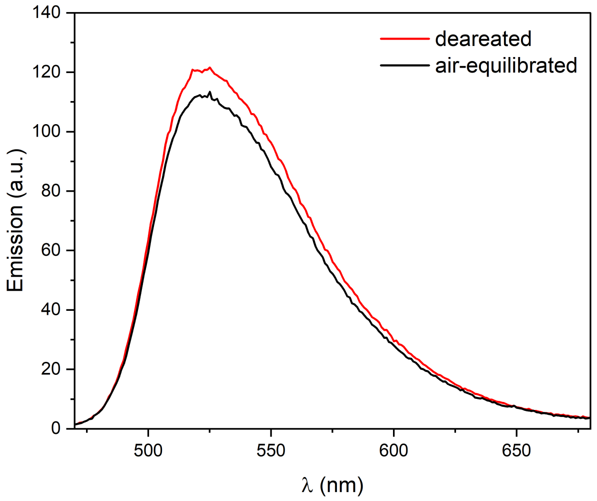

Figure S81. Emission spectra of DSAI 0.1 mM with NaI 100 mM under different atmospheres (λ_{exc} = 355 nm, at isoabsorbance).

Comment: *2. The correlation between increased fluorescence quantum yield and catalytic activity is clear. Could a more quantitative analysis be provided—e.g., does the catalytic rate scale linearly with the calculated excited-state population across different aggregation conditions (counterion, concentration, salt)?*

Answer: We thank the referee for their comment. We have performed a closer analysis between the fluorescence quantum yields and the catalytic rates of TMB oxidation. While the trend is clear (Figure R2) and a linear fitting could be performed (with $y = 0.509x - 0.503$, $y = 0.734x - 0.528$, and $y = 2.35x - 2.53$, respectively) with a good fit ($R^2 = 0.971, 0.963, 0.970$, respectively), we opted for not including this in our manuscript for the following reasons: (*i*) the photocatalytic activity as we have observed in the revised work is due to a combination of singlet and triplet localized excited states; (*ii*) the photocatalytic activity is also dependent on the size of the aggregates; (*iii*) the oxidation catalytic activity reported is for TMB, which could be affected both by the diffusion and sizes of aggregate, and also by its two-fold positive charge which could be undergo repulsive forces with the positively charged surface of the aggregates.

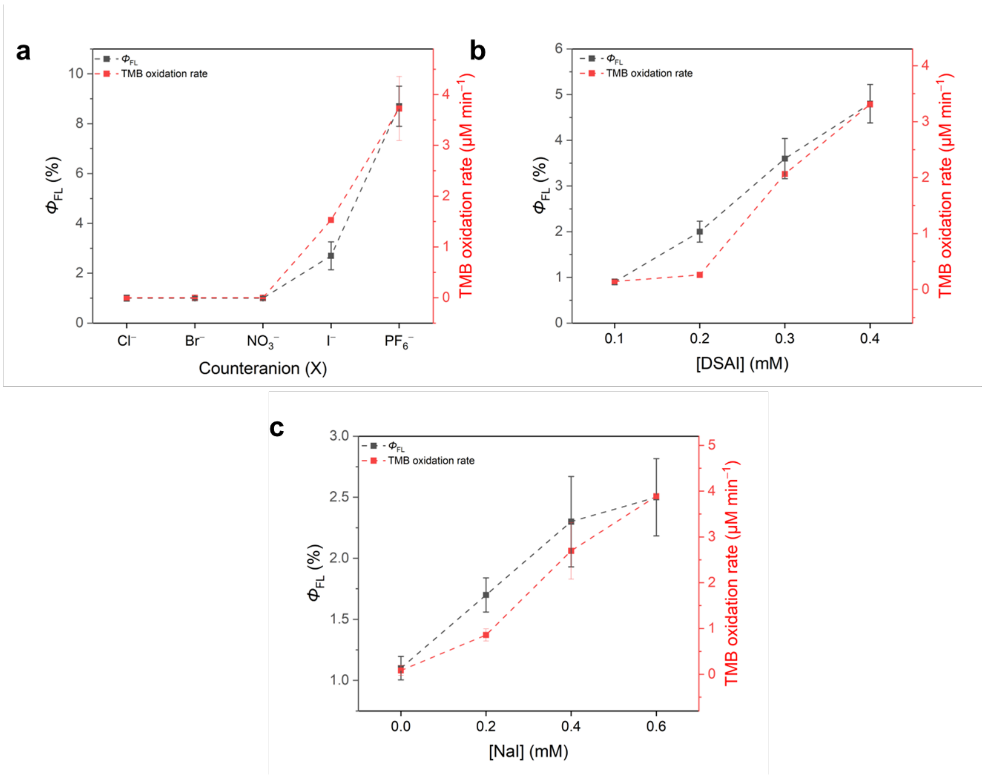

Figure R2. TMB oxidation rate *vs* fluorescence quantum yields (Φ_{FL}) related to a) DSAX 0.3 mM (data extracted from Figs. 3b-d), b) different concentrations of DSAI (data extracted from Figs. 3e-g and Supplementary Figs. S65, S68), and c) DSAI 0.1 mM with different concentrations of NaI (data extracted from Figs. 3h-j and Supplementary Fig. S66). At low Φ_{FL} (~1%), poor TMB oxidation (~0 μM min^{-1}) is obtained for each aggregation condition.

Comment: *3. The superior performance of kinetically trapped aggregates is attributed to higher excited-state availability from stacking faults. Could their smaller size and higher surface-to-volume ratio—and thus improved mass transport and substrate accessibility—also contribute significantly to the enhanced reaction rates?*

Answer: We thank the referee for highlighting this important point regarding size and the photocatalytic activity. Please note that we have addressed this point also as reply to Reviewer 1, comment 2.
We performed further experiments to evaluate the impact of crystals size and surface-to-volume ratio on photocatalysis using DSAI, whose annealing could be exploited to prepare crystals with tunable size, and $DSAPF_6$, in which two different procedures could be easily exploited due to its insolubility in water (specifically, sonication and nanoprecipitation).
First, DSAI was annealed at 95 °C and then the samples were stirred at different speeds (in presence of a magnetic stirrer) during the slow cooling phase to obtain crystalline samples with different sizes. The different sizes of the obtained crystals (at 800, 400, 200 and 0 rpm) were observed by fluorescence microscopy before and after irradiation (Figure R1a-d). These crystals were then used for photocatalytic H_2 evolution, which showed a clear trend with smaller sizes lead to higher photocatalytic activity (Figure R1e). While size plays an important role, we also note that aggregates with different dimensions also possess different photophysical properties. For example, in the original manuscript, we reported that kinetically trapped aggregates were smaller than their thermodynamic counterparts, but also differed in excited state availability (6% versus 14% of fluorescence quantum yield, respectively).
Second, we prepared $DSAPF_6$ crystals by two different methods: sonication or nanoprecipitation from a DMSO solution. Even in this case, smaller crystals lead to improved photocatalytic activity. However, similarly to the previous case, also the photophysical properties differ (longer lifetimes, higher fluorescence quantum yield, and higher photocatalytic performance toward both TMB oxidation and H_2 evolution for the nanoprecipitated crystals) (Fig. S124).

Revision: We have also included this further proof in our manuscript: "To further examine how crystal size influences photocatalysis, $DSAPF_6$ aggregates were prepared using either sonication or nanoprecipitation. As observed above, the smaller crystals (generated by nanoprecipitation) displayed enhanced photocatalytic activity. However, they also showed distinct photophysical behavior, including greater excited-state availability (Supplementary Fig. S124), indicating a complex interplay between size and excited-state dynamics that governs the photocatalytic performance."

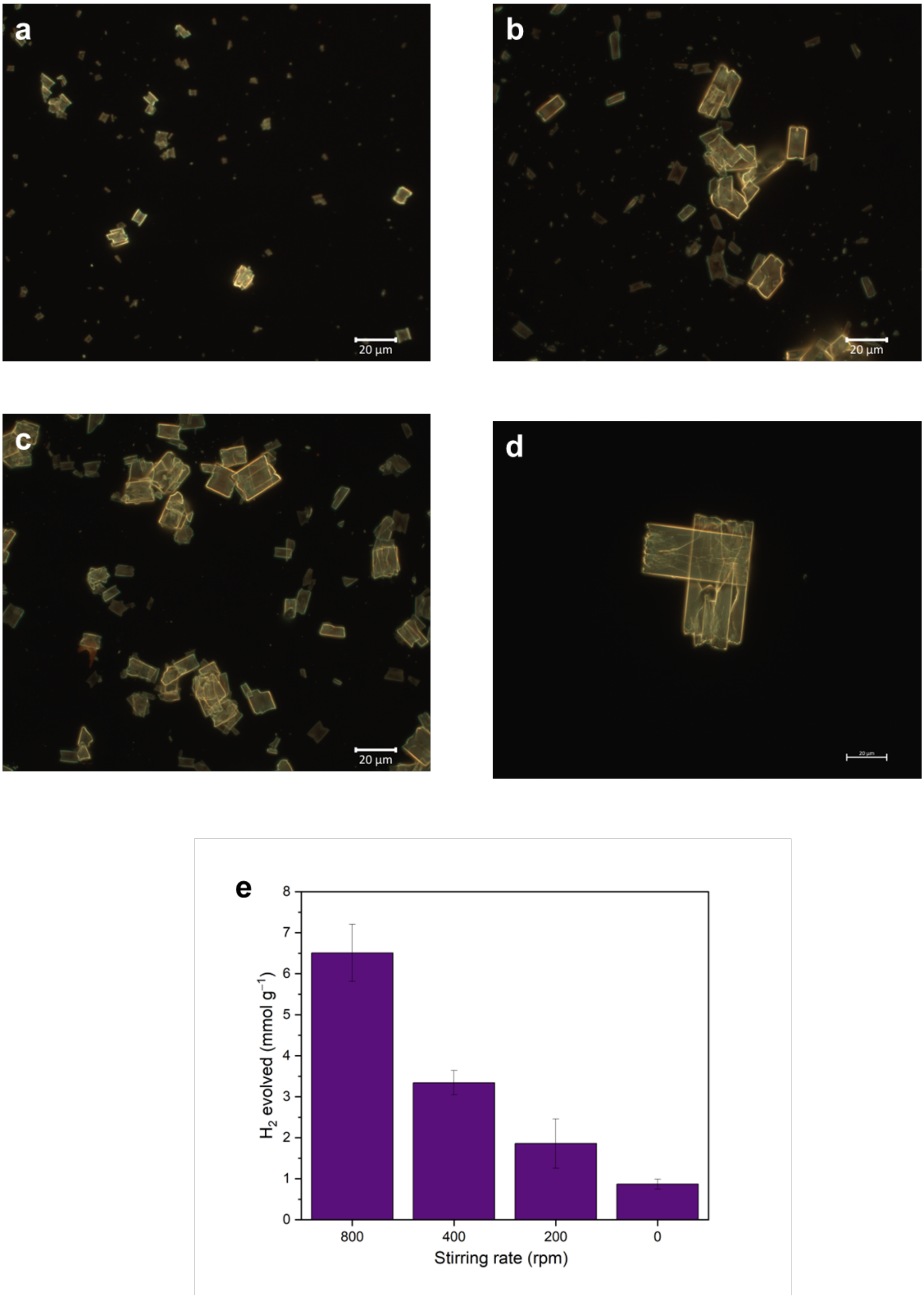

Figure R1. a) Widefield fluorescence image of DSAI 0.1 mM annealed and slowly cooled in presence of a stirring bar at 800 rpm, b) 400 rpm, c) 200 rpm and 0 rpm; e) H_2 evolution photosensitized by DSAI thermodynamic crystals obtained under different stirring conditions (800, 400, 200, and 0 rpm).

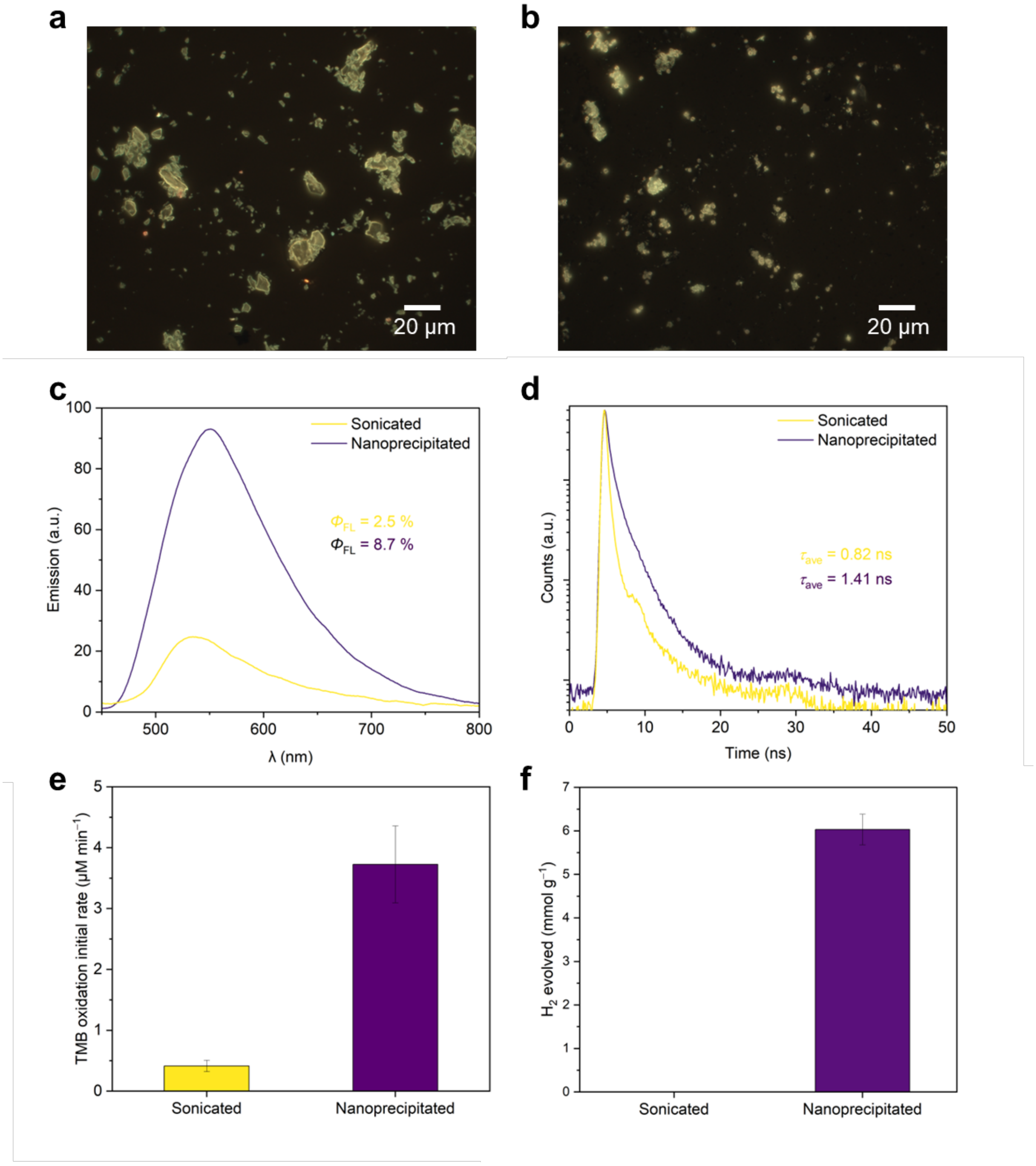

Figure S124. Widefield fluorescence images of $DSAPF_6$ 0.4 mg mL^{-1} a) sonicated in mQ-water and b) nanoprecipitated from DMSO in mQ-water. c) Fluorescence emission spectra and fluorescence quantum yields (Φ_{FL}) of $DSAPF_6$ 0.4 mg mL^{-1} sonicated *vs* nanoprecipitated in acetate buffer (0.1 M, pH = 5) containing 3.2% DMSO (*v/v*). d) Time-correlated single-photon counting (TCSPC) decay traces of the same samples. e) TMB oxidation rate (0.41, 3.7 µM min^{-1}) of same samples under white light (180 mW cm^{-2}). f) H_2 evolution produced by $DSAPF_6$ 0.4 mg mL^{-1} in ascorbic acid (1.0 M, pH = 4.0) sonicated *vs* nanoprecipitated from DMSO, in presence of PtNPs (8% mol) under 4 hours white light irradiation (100 mW cm^{-2}).

Comment: *4. Have the long-term (e.g., multi-day) photostability and recyclability of kinetic aggregates been assessed? Is there a risk of restructuring into less active thermodynamic forms during prolonged use?*

Answer: We are grateful to the reviewer for this comment. To clarify these important points, we performed several experiments to assess the photocatalytic stability and recyclability of the aggregates.

First, we confirm that kinetically trapped aggregates prepared by using excess salt, as the name *kinetically trapped* initially suggested but was not proven, are photostable and do not restructure into their thermodynamic form. This has been achieved through:

(i) Both freshly prepared samples and samples aged three days showed comparable photocatalytic activity (Supplementary Figure S121). The two DSAI samples were assessed for photocatalytic H_2 evolution under standard conditions (DSAI 0.10 mM, NaI 25 mM, ascorbic acid 1.0 M, pH = 4.0, PtNPs 8% mol).

(ii) The morphology is unchanged when comparing pre- and post-catalytic samples, as observed through SEM (Supplementary Fig. S122 in the revised manuscript)

(iii) When working under non-kinetically trapped conditions (i.e. without excess salt), the sample does indeed undergo growth also to the thermodynamic form. We purposefully utilized this approach, known as seeded-growth, to further confirm that smaller aggregates (ie. kinetically trapped) are more photocatalytically active (both in terms of TMB photooxidation and H_2 evolution) than their thermodynamic counterparts (Supplementary Figs. S119-S120).

Second, we investigated the recyclability of our system through different photocatalytic cycles, both for H_2 evolution (Supplementary Fig. S99 in the revised manuscript) and light-driven TMB oxidation (Supplementary Fig. 100 in the revised manuscript). When recovered, the solid continued to be photocatalytically active, as opposed to the filtrate that did not show any activity. Along the different cycles, however, the activity progressively decreased, but this could be also attributed to simple loss of solids during the filtration process.

Revision: We have made the following changes to the manuscript (highlighted in yellow in the revised manuscript). We have modified the following sentence: "We then compared the photocatalytic performance of thermodynamic and kinetically trapped aggregates, with the latter formed under high ionic strength conditions that suppress nucleation-elongation events."

We have also clarified that kinetically trapped aggregated do not change over time: "Moreover, the kinetically trapped aggregates do not grow into their thermodynamic counterparts over days, which we confirmed by aged samples that showed unchanged morphology and comparable photocatalytic activity (Supplementary Figs. S121-S122)."

We also changed Figure 6 in the revised manuscript underlying that *kinetic aggregates* are indeed *kinetically trapped.*

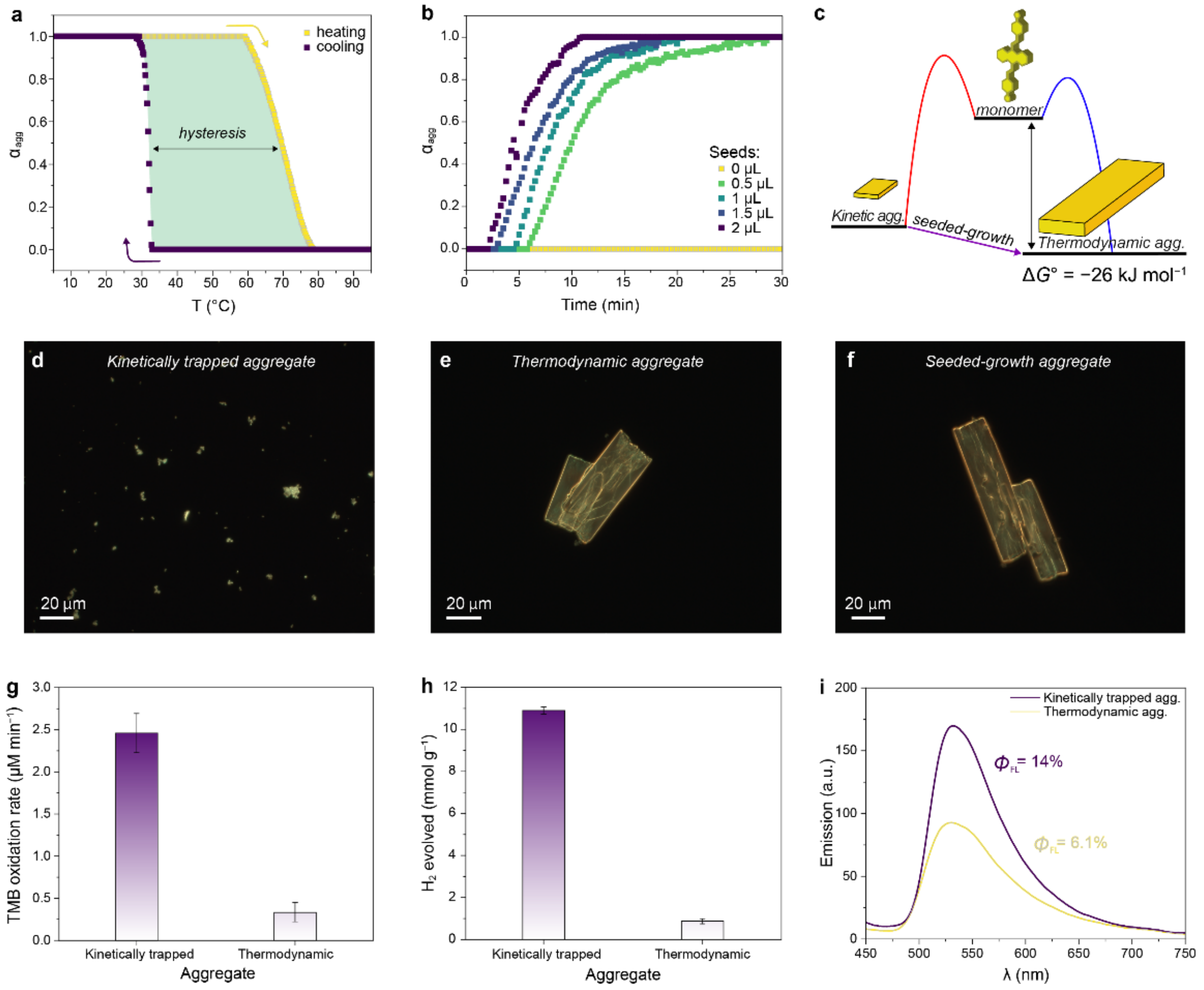

Regarding the Supporting Information, we added:

"The recyclability was tested by running 15 min irradiation experiments and filtering the samples after the quantification using polycarbonate membrane filter (0.1 µm pore size) under vacuum. $DSAPF_6$ 0.3 mM nanoprecipitated from DMSO in 3 mL acetate buffer was prepared as reported above. Subsequently, TMB (50 µL, 2 mg mL^{-1} in DMSO:mQ-water, 6:4) was added. After each cycle, fresh acetate buffer (3 mL) and TMB (50 µL) were added.

[...] Same procedure was used to prepare the DSAI seeded-growth sample. However, 396 µL were added to a vial instead of 400 µL. The remaining 4 µL was added at bath temperature of 50 °C (molar ratio 99:1 between dissolved DSAI molecules and DSAI seeds).

[…] Seeded-growth samples were prepared annealing 62 µL of DSAI 1.5 mM at 95 °C for 10 minutes. Then, 5 µL of DSAI 0.5 mM were added to the solution at 50 °C and left overnight. At room temperature, 1.6 µL of PtNPs, 25 µL of a stock solution of NaI 1.0 M and 906 µL of ascorbic acid (1.0 M, pH = 4.0) were added."

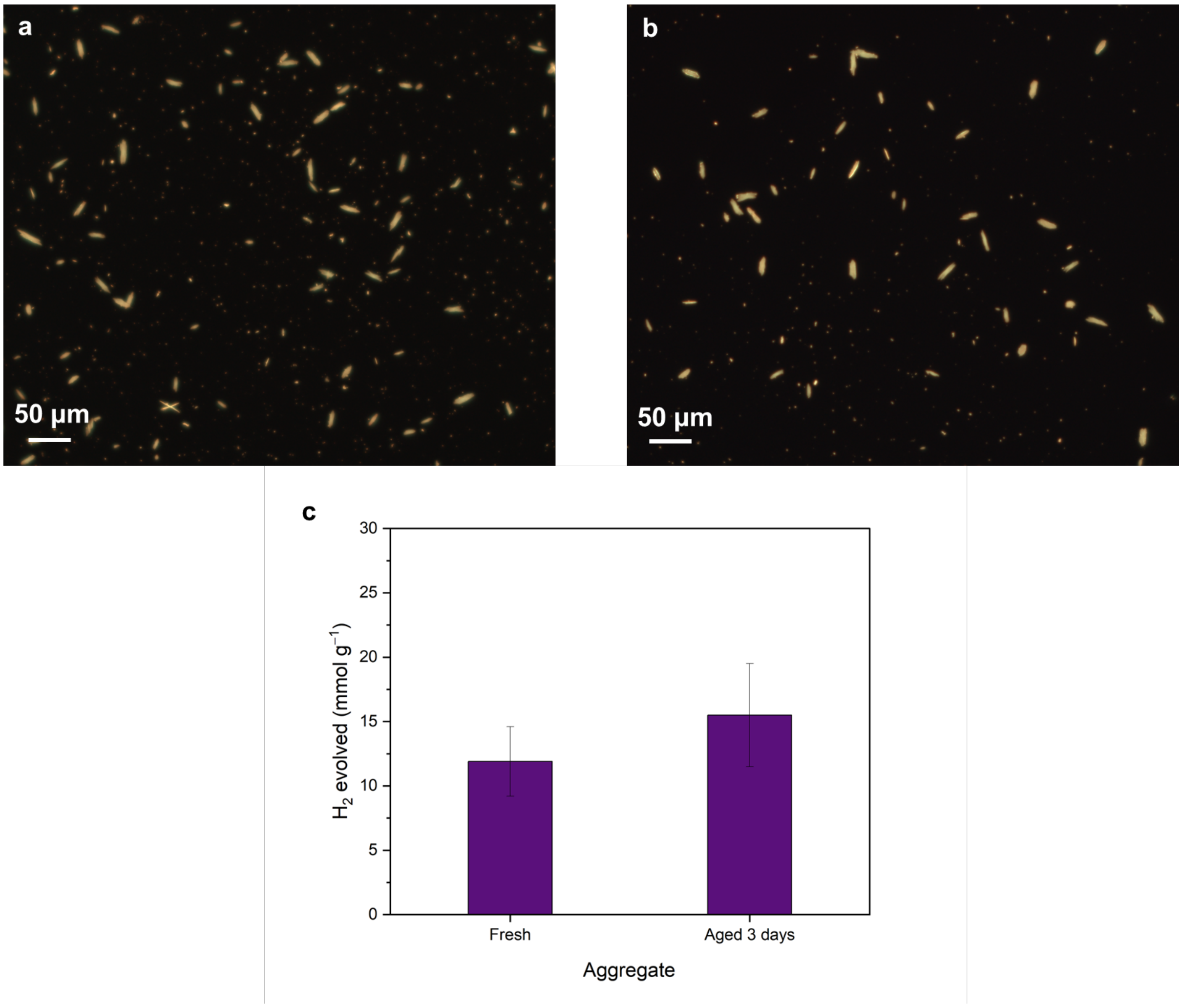

Figure S1212. Widefield fluorescence images of kinetically trapped DSAI aggregates (0.1 mM) with NaI (25 mM) in ascorbic acid (1.0 M, pH = 4.0), in presence of PtNPs (8% mol), a) freshly prepared and b) aged three days. c) H_2 evolved by fresh and aged three days kinetically trapped aggregate (DSAI 0.1 mM, NaI 25 mM, ascorbic acid 1.0 M, pH = 4.0, PtNPs 8% mol) after 4h irradiation under white light (100 mW cm^{-2}).

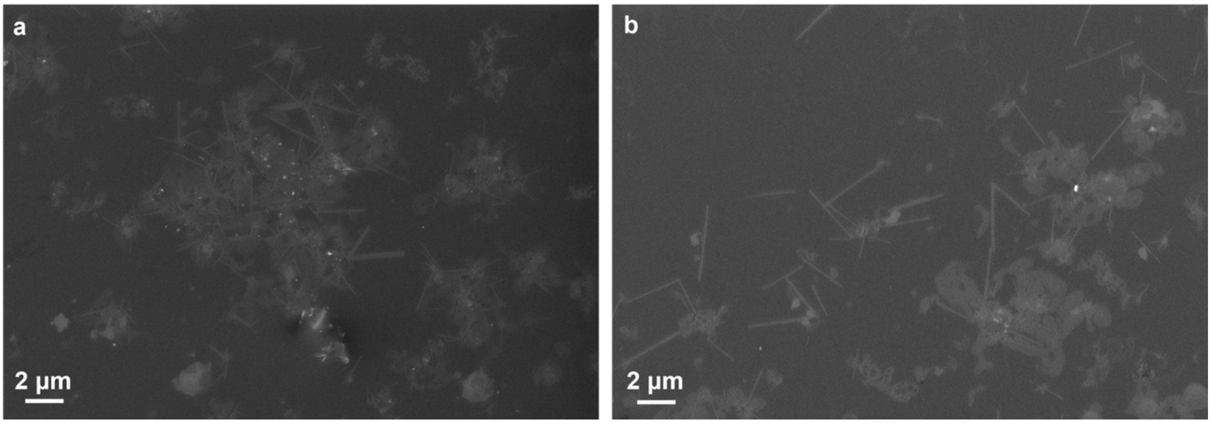

Figure S1223. SEM images of DSAI 0.1 mM in ascorbic acid (1.0 M, pH = 4.0) in presence of NaI (25 mM) and PtNPs (8% mol) aggregates a) pre- and b) post-photocatalytic H_2 evolution. These images show that the morphology is strictly retained in these kinetically trapped aggregates.

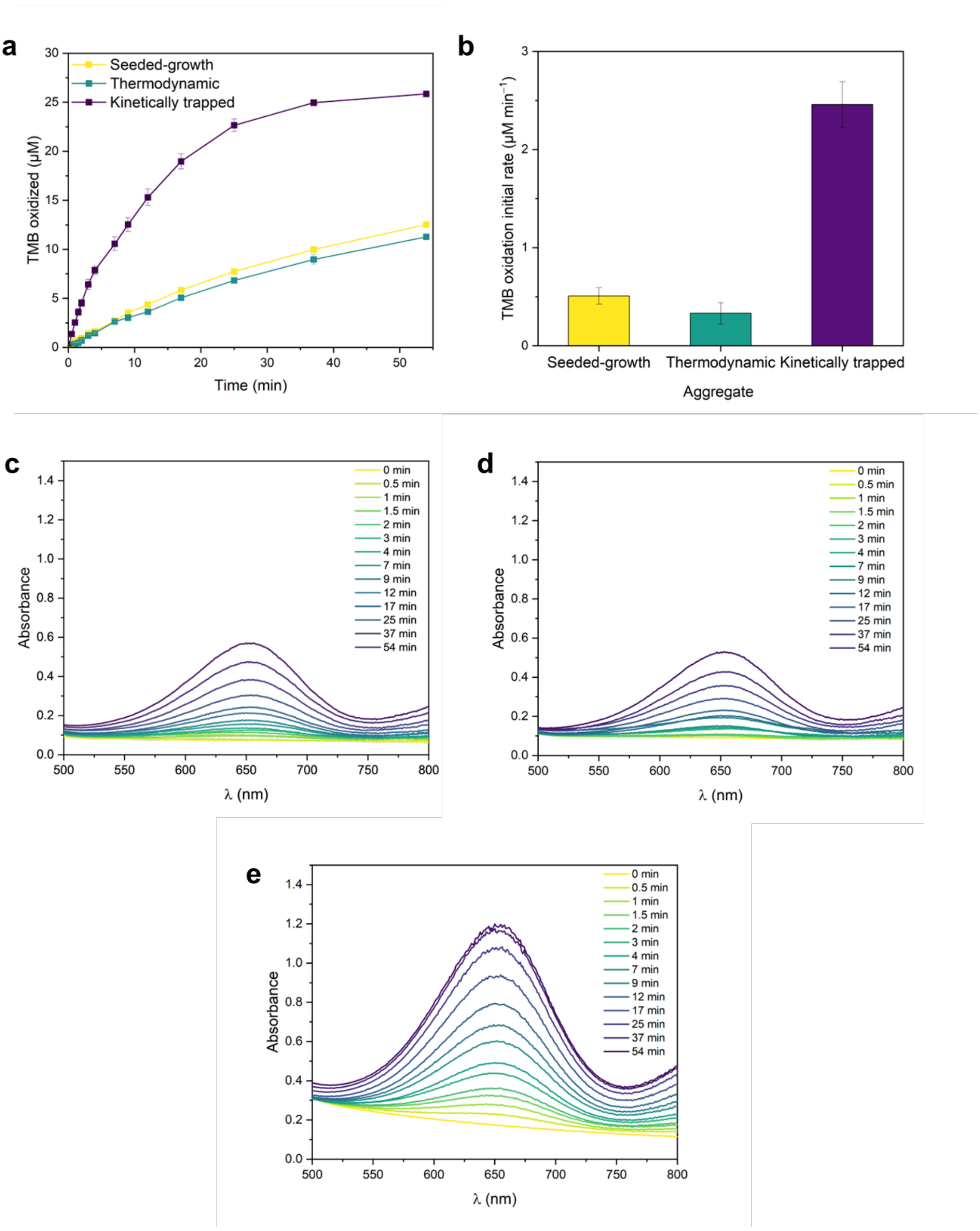

Figure S119. a) Irradiation of various DSAI aggregated states (0.1 mM) with NaI (0.6 mM) in presence of TMB (134 µM) over time in acetate buffer (0.1 M, pH = 5) under white light (180 mW cm^{-2}) under air. b) TMB oxidation rate extracted from traces in panel a): 0.51, 0.33, 2.44 µM min^{-1} (from left to right. c) UV-Vis traces using DSA seeded-growth aggregate; d) UV-Vis traces using DSAI thermodynamic aggregate; d) UV-Vis traces using DSAI kinetically trapped aggregate.

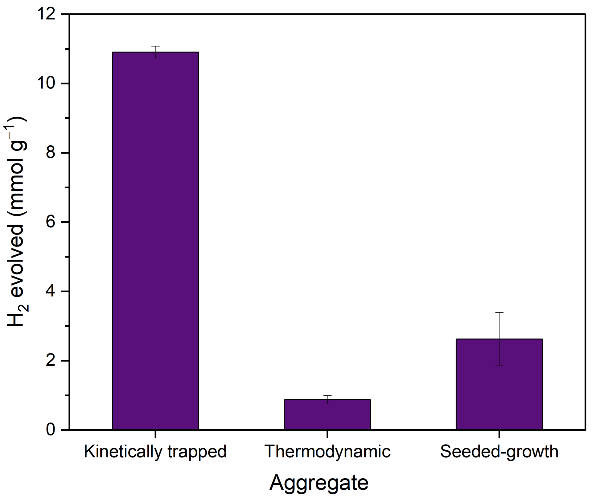

Figure S1204. H_2 evolution by irradiation of various DSAI aggregated states (0.1 mM, NaI 25 mM) with white light (LED, 100 mW cm^{-2}) in presence of ascorbic acid (1.0 M, pH = 4.0) and co-catalyst (PtNPs, 8% mol).

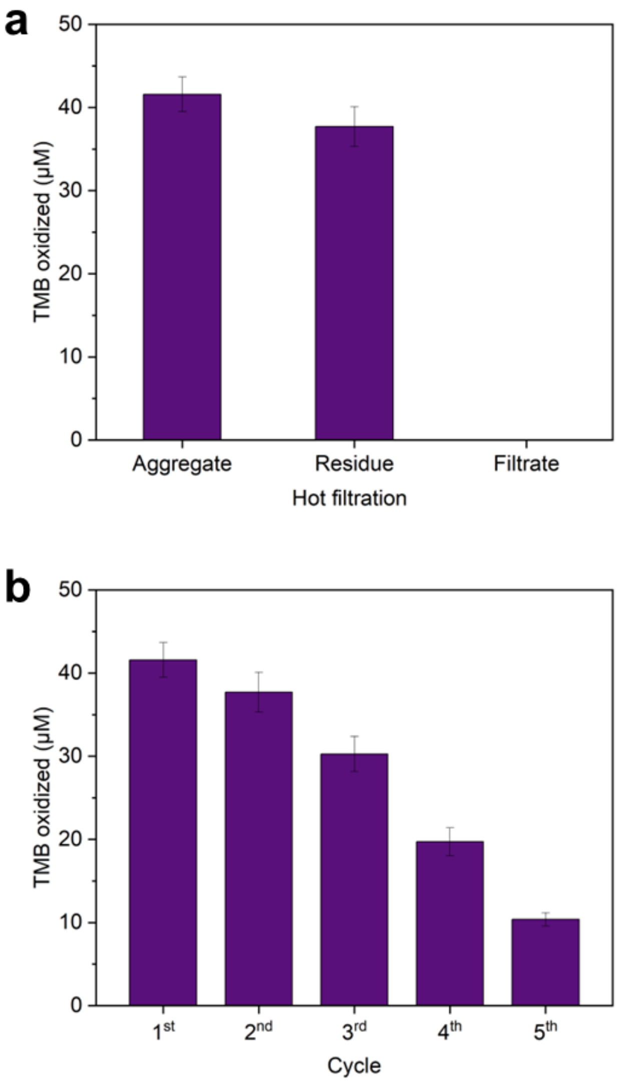

Figure S100. a) Hot filtration and b) recycling tests for light-driven TMB oxidation using $DSAPF_6$ (0.24 mg mL^{-1}) nanoprecipitated from DMSO in presence of TMB (134 μM) in acetate buffer (0.1 M, pH = 5) under white light (180 mW cm^{-2}) under air. 15 min each cycle; samples were filtered using a polycarbonate membrane filter (0.1 μm pore size). After each cycle. fresh acetate buffer and TMB were added.

Comment: *5. The generalization of AIP to a TPE derivative is compelling. What specific molecular features are deemed essential for AIP? Would a fully rigid, planar chromophore—lacking conformational flexibility—still benefit from this strategy?*

Answer: We thank the referee for their comment. We hypothesize that any molecular feature that contributes to loss of the excited states when the chromophore is free in solution – rotation, isomerization, vibration, etc. – could be potentially addressed (i.e. blocking this unproductive excited state deactivation) through aggregation. Fully rigid and planar chromophores, however, will inevitably lead to excited states delocalization due to strong π-π interactions in water. These systems will behave as the ones we discussed as traditional self-assembled dyes that undergo aggregation-caused quenching (ACQ) and lead to photocatalytic activity through exciton delocalization and charge separation.

Revision: In response to this comment, we have made the following changes to the manuscript. In the introduction, we have specified the following: "In this context, aggregation is employed not to stabilize excited states, but to extend them across multiple planar or rigid chromophores through π–π stacking or donor–acceptor interactions in crystalline assemblies.[1–5,28–35] In these photocatalytic systems, an aggregation-caused quenching (ACQ) phenomenon is observed, but is not the only requisite for photocatalysis. Rather, the organic nanostructures were shown to drive key reactions in artificial photosynthesis, including H_2 evolution and H_2O_2 production, when emission quenching leads to exciton migration and charge separation along the crystalline domains. However, such approaches inherently rely on electronic coupling between chromophores, often requiring planar scaffolds and specific packing motifs, which can be difficult to predict and design rationally."
In the concluding remarks we have added the following sentences: "In our system, the mechanistic decoupling of supramolecular structure from excited-state energetics simplifies rational design and predictive control. In this respect, aggregation-induced rigidification provides a supramolecular route that complements established covalent strategies for chromophore rigidification, including sterically constraining structures,[69] or preparing covalent organic frameworks, macrocycles, molecular cages and organic-inorganic superlattices.[17,42,70–72]"

Comment: *6. Beyond initiating aggregation via the reverse Hofmeister effect, do counterions (e.g., I^-, $PF6^-$) remain associated within the aggregate and influence the excited-state electronic structure or facilitate electron transfer?*

Answer: We thank the referee for this very helpful suggestion. To investigate whether the counterions remain associated or not within the aggregate, we performed TEM-EDX experiments. The micrographs disclosed that the anions are present only within the nanostructures, thus confirming that they remain associated within the aggregate (Supplementary Figs. S49-S53 in the revised manuscript).
Further experiments showed a strong correlation between aggregates size and photocatalytic activity. For instance, we prepared $DSAPF_6$ crystals by two different methods: sonication or nanoprecipitation. Similarly to what we have observed with DSAI thermodynamic and kinetically trapped aggregates, smaller $DSAPF_6$ crystals lead to improved photocatalytic activity. However, also the photophysical properties differ (longer lifetimes, higher fluorescence quantum yield, and higher photocatalytic performance toward both TMB oxidation and H_2 evolution for the nanoprecipitated crystals) (Supplementary Fig. S124). While both size and photophysical properties of the aggregates appear to influence their photocatalytic activity, the interplay between these deserves more comprehensive experiments that are beyond the scope of this manuscript and should be addressed in future work. To summarize, excited-state electronic structures is influenced by the counterions in the way they influence different assembly, including packing but also aggregates size, which strongly correlate with photophysical properties and consequent photocatalytic activity.

Revision: we have added the following section in the main text in which we discuss the counterion effect and their inclusion in the nanostructure "Increasing the ionic strength of the solution through salt addition screens the positive charges on the molecules, thereby reducing electrostatic repulsion and promoting hydrophobic collapse, ultimately leading to aggregation. Consistent with this mechanism, aggregation of DSAX (X = Cl^-, Br^-, NO_3^-, and I^-) can be induced by addition of the corresponding sodium salts (NaX), which increases charge screening and leads to formation of aggregates (Supplementary Figs. S44-S46). Importantly, aggregation can be induced by adding inorganic salts with counteranions different from those originally present with the dye, enabling solvated molecules to aggregate. For example, DSACl undergoes aggregation upon addition of NaI (Supplementary Figs. S47-S48), demonstrating that both ionic strength and counteranion identity contribute to aggregation. Supporting this interpretation, we observed that counteranions are not

merely dissociated but remain localized within the aggregates, confirming they are embedded within the aggregates and contribute to the overall aggregation (Supplementary Figs. S48-S54).

[…] To further examine how crystal size influences photocatalysis, $DSAPF_6$ aggregates were prepared using either sonication or nanoprecipitation. As observed above, the smaller crystals (generated by nanoprecipitation) displayed enhanced photocatalytic activity. However, they also showed distinct photophysical behavior, including greater excited-state availability (Supplementary Fig. S124), indicating a complex interplay between size and excited-state dynamics that governs the photocatalytic performance."

Regarding the Supporting Information, we added the following:

- DSACl with NaCl (0.05 mM and 1 M, respectively) was prepared in a vial by adding 1.1 mL of mQ-water, followed by 0.4 mL of 5 M NaCl to a 0.5 mL solution of 0.2 mM in DSACl previously dissolved in mQ-water. The sample was stirred for 15 min.
- DSACl with NaI (0.05 mM and 3 mM, respectively) was prepared in a vial by adding 1.5 mL of mQ-water, followed by 6 µL of 1 M NaCl to a 0.5 mL solution of 0.2 mM in DSACl previously dissolved in mQ-water. The sample was stirred for 5 min.
- DSABr with NaBr (0.05 mM and 0.03 M, respectively) was prepared in a vial by adding 1.5 mL of mQ-water, followed by 12 µL of 5 M NaBr to a 0.5 mL solution of 0.2 mM in DSABr previously dissolved in mQ-water. The sample was stirred for 15 min.
- $DSANO_3$ with $NaNO_3$ (0.05 mM and 0.1 M, respectively) was prepared in a vial by adding 1.5 mL of mQ-water, followed by 40 µL of 5 M $NaNO_3$ to a 0.5 mL solution of 0.2 mM in $DSANO_3$ previously dissolved in mQ-water. The sample was stirred for 15 min.
- $DSAPF_6$ samples were prepared either by sonication or nanoprecipitation. Sonicated samples were prepared by weighting DSAPF6 and adding mQ-water, followed by sonication for 10 minutes. For the nanoprecipitated sample, a solution of $DSAPF_6$ in DMSO (5 mM) was prepared and then 0.1 mL of this solution was added to a new vial containing 1.9 mL of mQ-water and under stirring. The sample was stirred for 60 min.

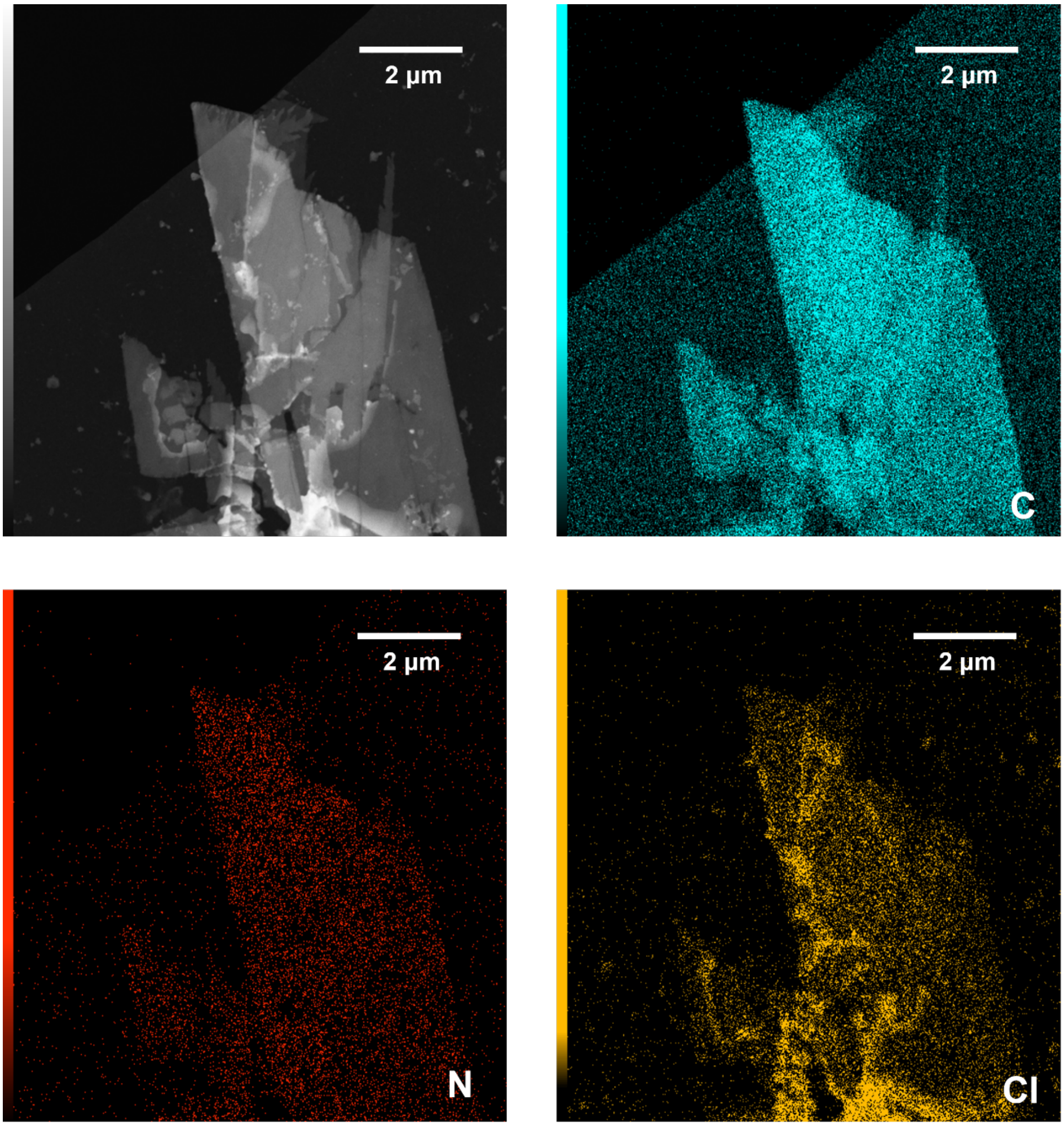

Figure S50. TEM-EDX micrographs of 0.05 mM DSACl in the presence of 1 M NaCl in mQ-water showing the content of carbon (top right), nitrogen (bottom left), and chloride (bottom right).

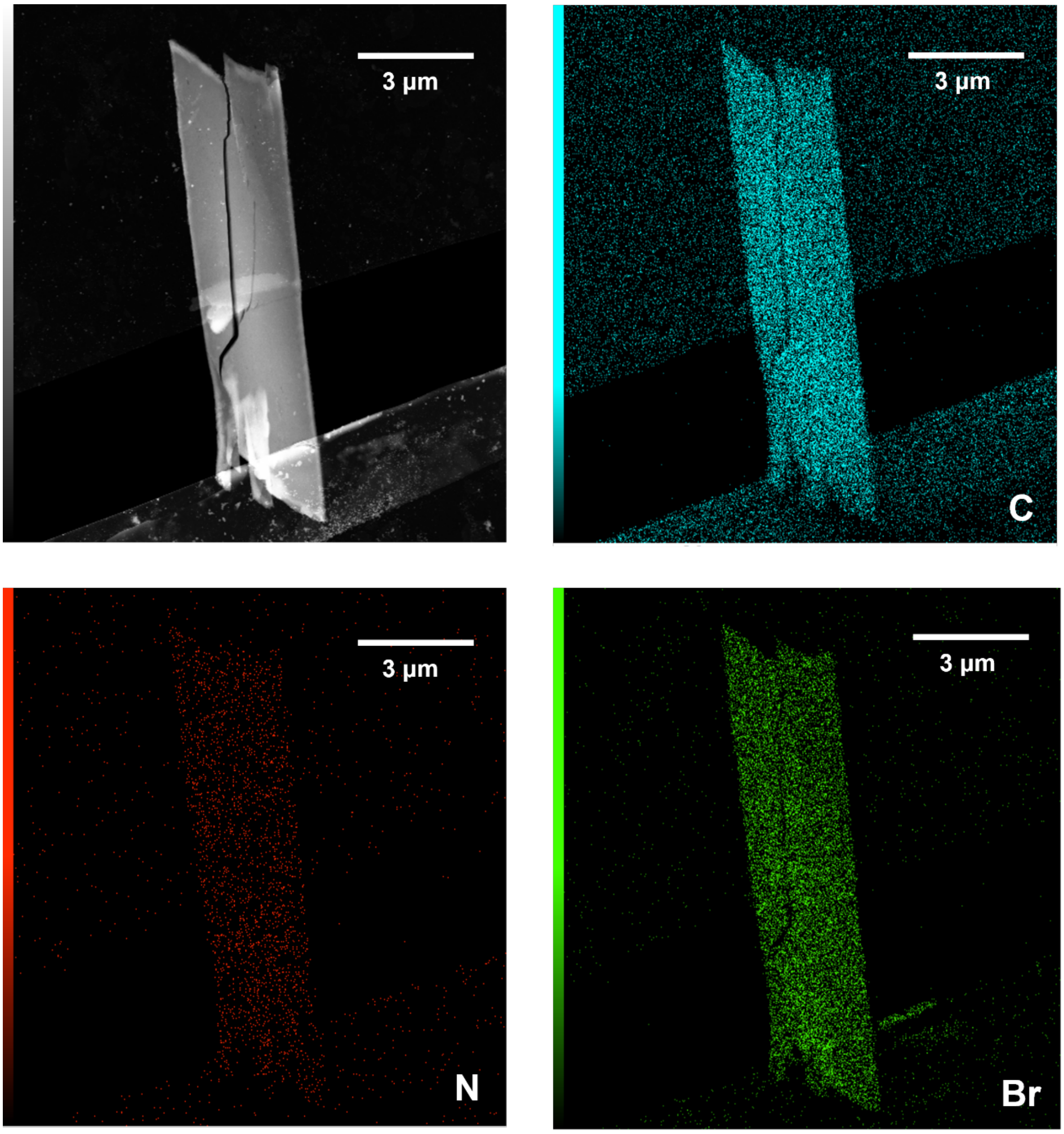

Figure S515. TEM-EDX micrographs of 0.05 mM DSABr in the presence of 0.03 M NaBr in mQ-water showing the content of carbon (top right), nitrogen (bottom left), and bromide (bottom right).

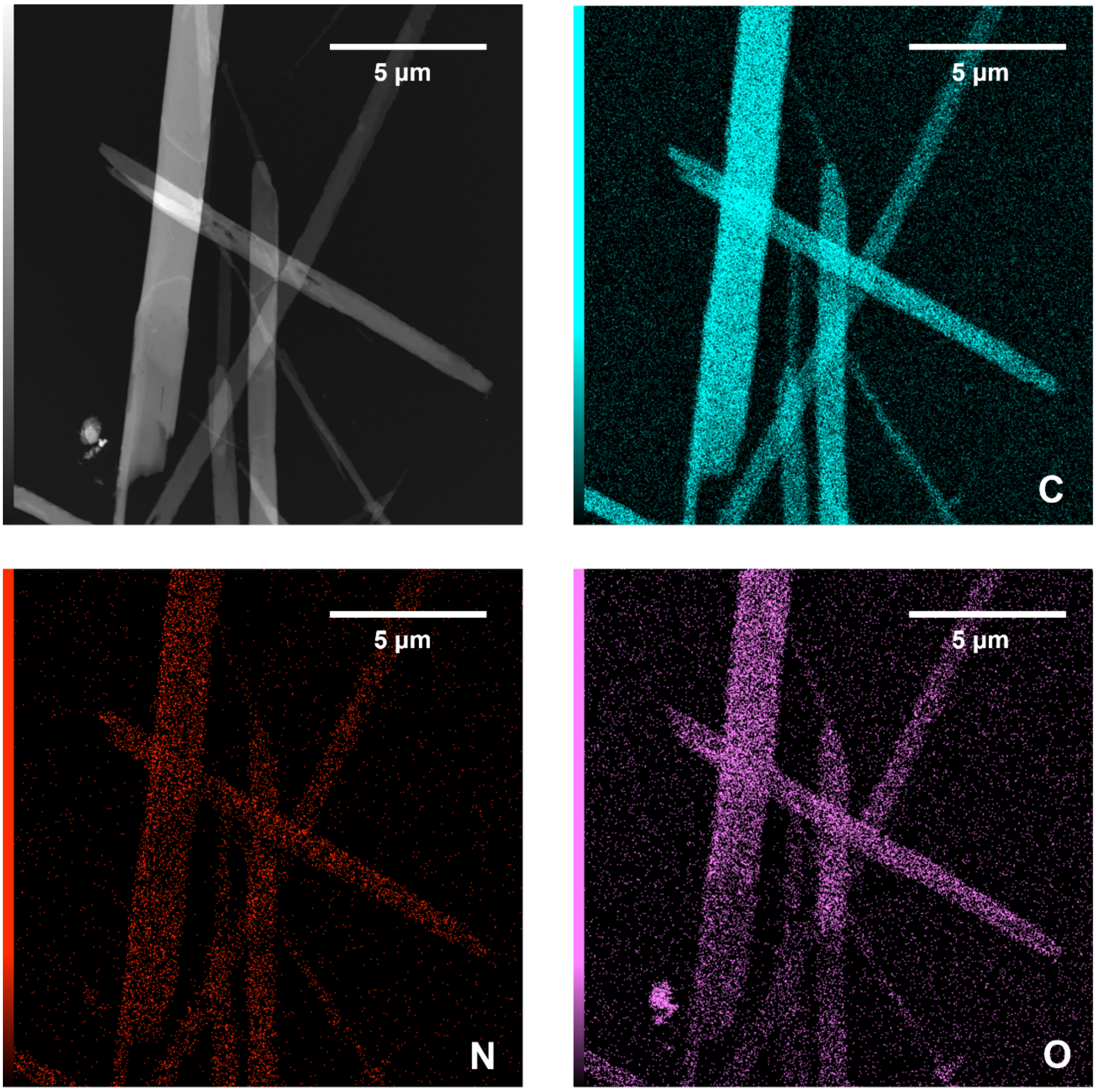

Figure S52. TEM-EDX micrographs of 0.05 mM $DSANO_3$ in the presence of 0.1 M $NaNO_3$ in mQ-water showing the content of carbon (top right), nitrogen (bottom left), and oxygen (bottom right).

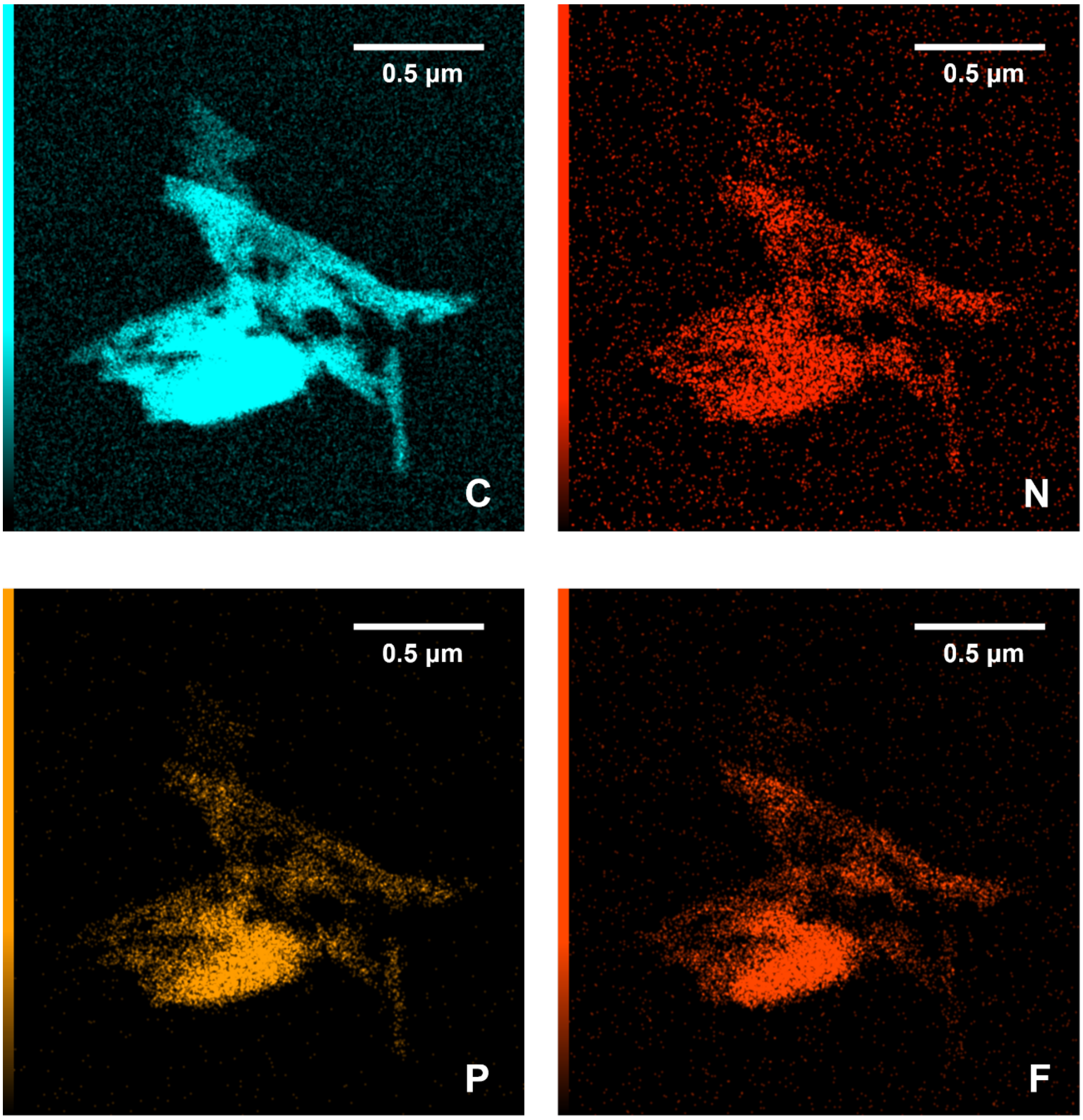

Figure S53. TEM-EDX micrographs of 0.3 mM DSAPF_6 in mQ-water nanoprecipitated from DMSO showing the content of carbon (top left), nitrogen (top right), phosphorous (bottom left) and fluorine (bottom right).

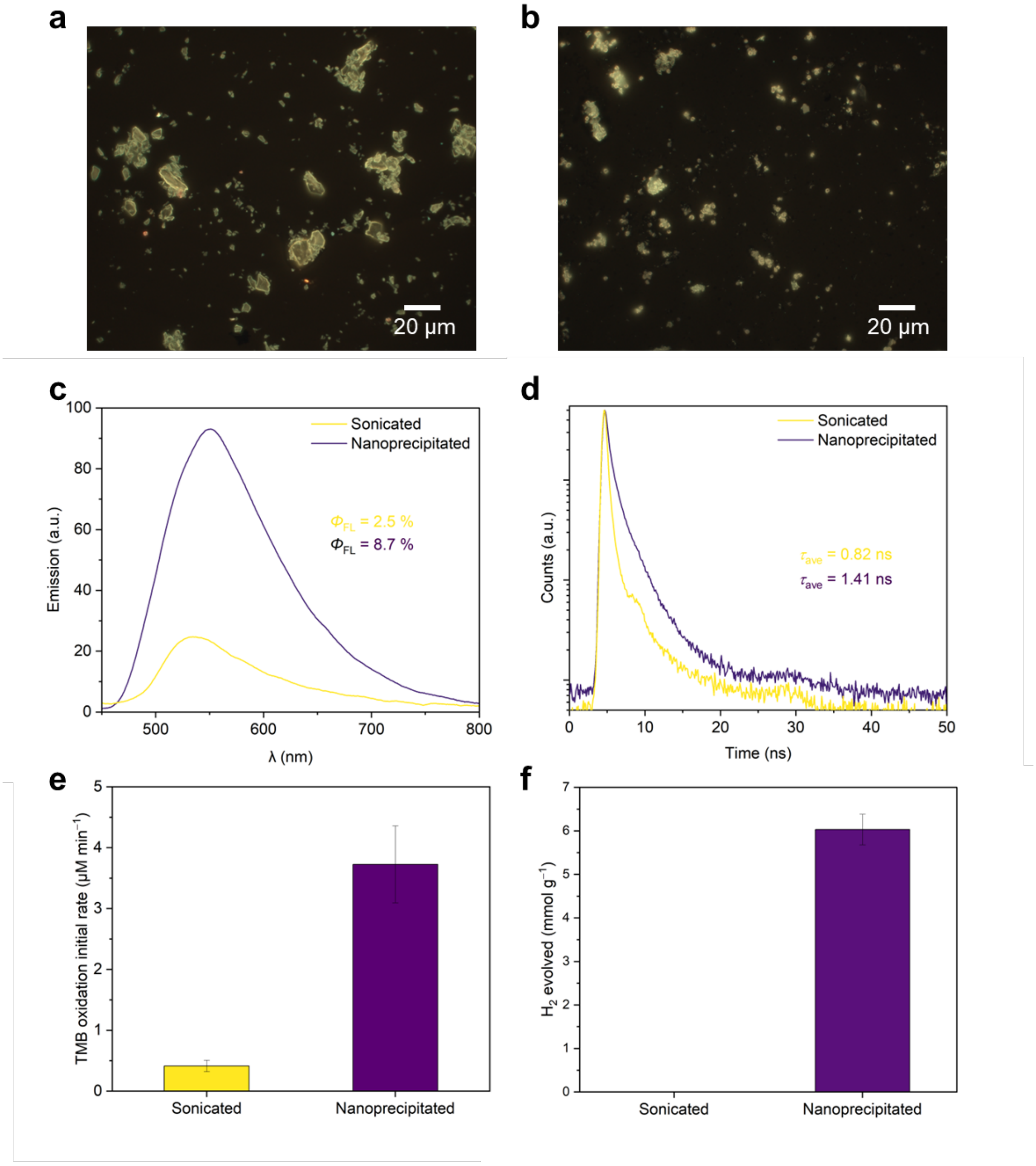

Figure S124. Widefield fluorescence images of $DSAPF_6$ 0.4 mg mL^{-1} a) sonicated in mQ-water and b) nanoprecipitated from DMSO in mQ-water. c) Fluorescence emission spectra and fluorescence quantum yields (Φ_{FL}) of $DSAPF_6$ 0.4 mg mL^{-1} sonicated *vs* nanoprecipitated in acetate buffer (0.1 M, pH = 5) containing 3.2% DMSO (*v/v*). d) Time-correlated single-photon counting (TCSPC) decay traces of the same samples. e) TMB oxidation rate (0.41, 3.7 µM min^{-1}) of same samples under white light (180 mW cm^{-2}). f) H_2 evolution produced by $DSAPF_6$ 0.4 mg mL^{-1} in ascorbic acid (1.0 M, pH = 4.0) sonicated *vs* nanoprecipitated from DMSO, in presence of PtNPs (8% mol) under 4 hours white light irradiation (100 mW cm^{-2}).

Comment: *7. Was wavelength-dependent reactivity observed? Such data could indicate whether catalytic activity is associated with specific vibronic transitions or aggregate species with distinct energies.*

Answer: We thank the reviewer for suggesting wavelength-dependent photocatalytic experiments. To address this comment, we have conducted additional experiments for H_2 evolution under irradiation at different wavelengths (Supplementary Fig. S98). The trend observed follows the absorption profile of the DSAI aggregate, with the highest activity corresponding with the aggregate absorption maximum.

Revision: We modified the main text and the supplementary information to include these experiments. The following sentence was modified in the main text "The apparent quantum yield for photocatalytic H_2 evolution was determined to be 0.2%, with the photocatalytic activity matching the absorption profile of the aggregate (Supplementary Fig. S98) and consistent with reported values for nanostructured chromophore amphiphiles.[25]" and the following Figure was addended to the Supplementary Information:

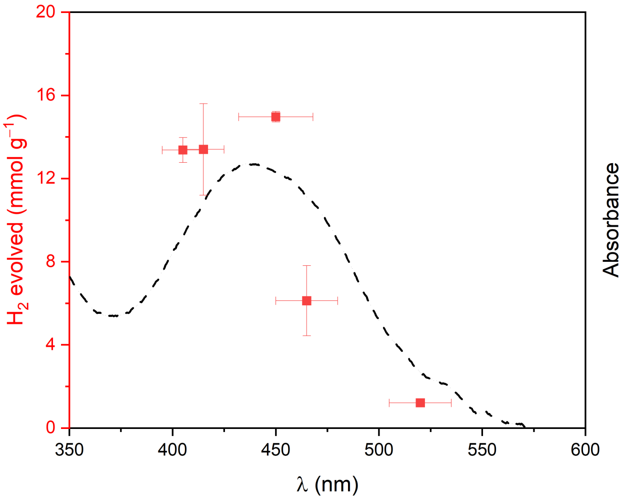

Figure S986. H_2 evolution produced by DSAI 0.1 mM in ascorbic acid (1.0 M, pH = 4.0) in presence of NaI 25 mM and PtNPs (8% mol) under 4 hours irradiation at different wavelengths at 26 mW cm^{-2} and DSAI absorption spectrum in the same conditions.

Comment: *8. With an apparent quantum yield of 0.2% for H2 evolution, what is identified as the primary efficiency-limiting factor—e.g., intrinsic electron transfer rate, back-electron transfer, sacrificial donor regeneration, or the co-catalyst interface?*

Answer: We thank the referee for this comment. According to the proposed mechanism for H_2 evolution in our system, the excited state of the chromophore interacts with the co-catalyst and transfers the electron to the Pt centre, which is then followed by regeneration of the chromophore by the sacrificial donor.

The known acceleration of the electron transfer rate upon introduction of metal co-catalysts in organic semiconductors, together with the high concentration of sacrificial donor employed here (1.0 M ascorbic acid), strongly point at the electron transfer step to the co-catalyst and the photosensitizer-co-catalyst interface as the main efficiency-limiting factors.

We identify these as particularly relevant limitations especially because we used premade commercial PtNPs. While this choice ensures reproducibility and enables direct comparisons across experiments and laboratories, electron transfer to pre-formed NPs is frequently constrained by surface chemistry and residual ligands rather than by optimal alignment with redox-active sites. We therefore anticipate that great improvements could be achieved by photodeposition rather than

impregnation as loading co-catalyst method. With photodeposited co-catalysts, nucleation occurs at electron-rich sites under illumination, promoting intimate electronic coupling and minimizing interfacial barriers; under this conditions, proton reduction rather than electron delivery becomes the rate-limiting step.

Revision: We added this lines to the main text: "We anticipate that further gains in efficiency could be achieved by improving electron-transfer kinetics and interfacial coupling between the aggregates and co-catalysts. In this context, in situ photodeposition of Pt nanoparticles is known to promote more efficient electron transfer and a better co-catalyst distribution than impregnation with pre-formed, ligand-capped Pt nanoparticles.[4]"

Comment: *9. Several mass spectra in the Supporting Information lack analysis, and some NMR spectra (e.g., Fig. S11, S24, S26) show impurity peaks. Purified spectra may be provided. Additionally, why does the NMR spectrum in Fig. S16 show two singlets? Could this indicate residual starting material?*

Answer: We thank the reviewer for the comment regarding some of the reported spectra. First, the ^{19}F NMR spectrum of $DSAPF_6$ (Supplementary Fig. S16) shows two signals because six equivalents ^{19}F are coupling with one ^{31}P (I = 1/2). Using the multiplicity rule, a doublet is expected from the ^{19}F NMR spectrum of $DSAPF_6$, although it may appear as two signals due to the very large coupling constant.
Second, we synthesized, purified and characterized again the compounds specifically mentioned by the referee, with the new ^{1}H, ^{13}C NMR and MS spectra now included in the Supplementary Information. We have also added, for DSAX compounds, the analysis of 234 and 242 m/z ions that are present in all ESI(+)-MS spectra, since they represent $[M - 2CH_3]^{2+}$, $[M - CH_3]^{2+}$ ions, respectively. Third, with the new batches we have performed again light-driven TMB oxidation, H_2O_2 production, and H_2 evolution experiments and compared them with the activities of the old batches. The results (Figs. R5-R8) showed that the activities are comparable and therefore do not influence the results from the original manuscript.

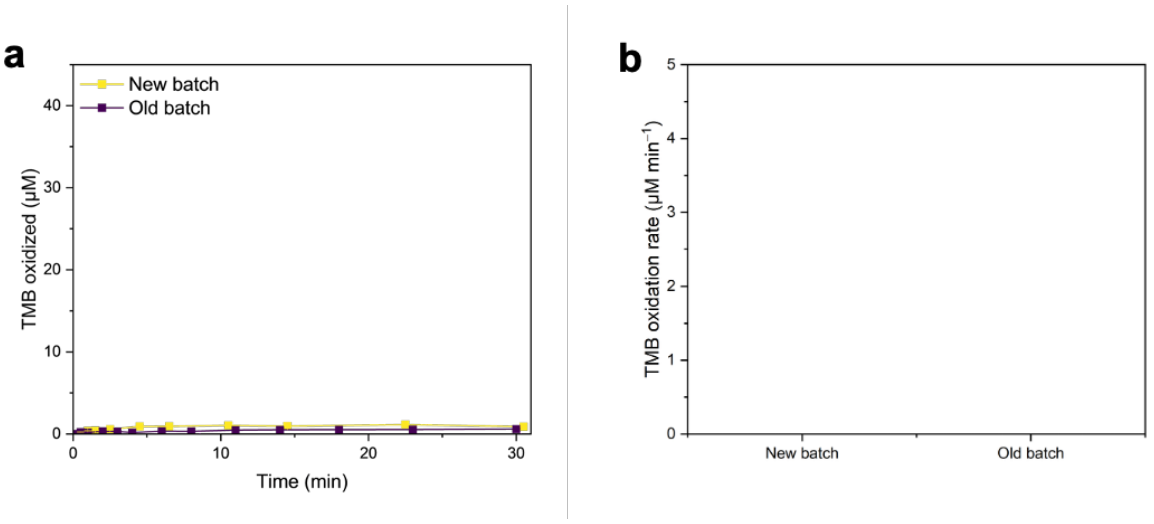

Figure R3. a) Irradiation of DSACl new batch *vs* old batch (0.3 mM) in presence of TMB (134 μM) over time in acetate buffer (0.1 M, pH = 5) under white light (180 mW cm^{-2}) and air. b) TMB oxidation rate extracted from traces in panel a): 0.0, 0.0 μM min^{-1}.

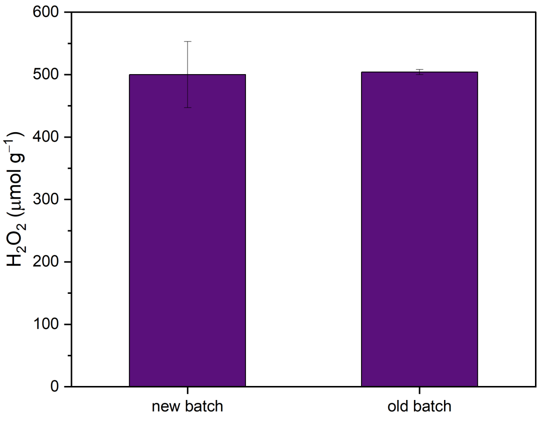

Figure R4. H_2O_2 produced by DSACl new batch *vs* old batch (0.5 mM) in presence of NaCl 0.9 M, in MOPS 0.1 M (pH = 7.0) after 1 hour under 415 nm irradiation (140 mW cm^{-2}).

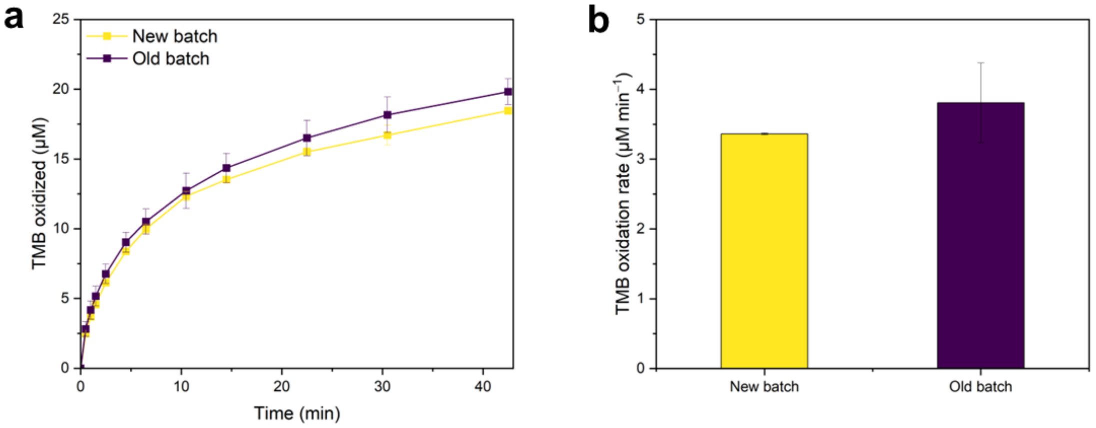

Figure R5. a) Irradiation of TBATPE new batch *vs* old batch (0.2 mM) aggregated with acetate buffer (60 mM, pH = 5.0) in presence of TMB (134 μM) over time under white light (180 mW cm^{-2}). b) TMB oxidation rate extracted from traces in panel a): 3.4, 3.8 μM min^{-1} (from left to right).

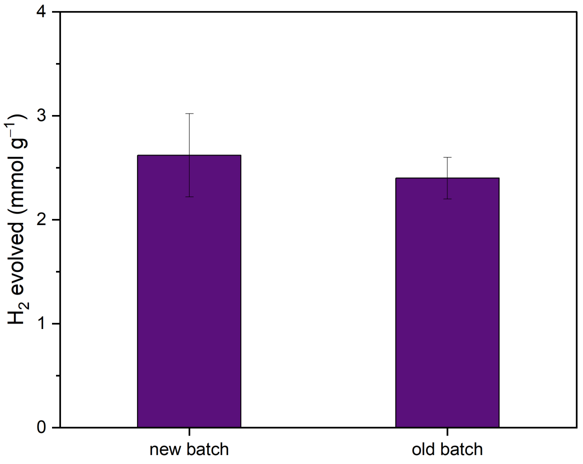

Figure R6. H_2 produced by TBATPE new batch *vs* old batch (0.1 mM) in ascorbic acid 0.8 M (pH = 4.0) with PtNPs 8% mol after 8 hours under 415 nm irradiation (140 mW cm^{-2}).

Revision: We have updated the Supplementary Information accordingly.

Comment: *10. Some recently published related papers may be of reference value, e.g., 10.1002/agt2.364, 10.1038/s41467-024-55060-w, 10.1007/s11426-025-2780-x, 10.1002/agt2.676.*

Answer: We thank the reviewer for the insightful suggestion, and we have updated the references in our manuscript accordingly.

Revision: These new citations were added in the main text as cit. 27, 41, 42, 43

Reviewer #3

This manuscript presents an interesting study on aggregation-induced photocatalysis (AIP), in which supramolecular dye polymerization rigidifies chromophores to stabilize localized singlet excited states and thereby enhances light-driven reactivity. The work integrates the molecular design with comprehensive spectroscopic, microscopic, and theoretical analyses, providing a new mechanistic perspective that contrasts exciton delocalization with excited-state confinement. While this manuscript is well organized, but the current data is not strong enough, and thus the authors are highly recommended to conduct more in-depth data collection and analysis. For example, more in-depth kinetic analysis, detailed energy-level mapping, and rigorous comparison with established photocatalytic benchmarks are needed to validate the universality and practical competitiveness of the reported AIP effect.

Answer: We are truly grateful for the evaluation of our manuscript and for recognizing the potential of our work. We have carefully addressed all the concerns raised and revised the manuscript accordingly. In the following, we provide concrete responses to the comments and suggestions point-by-point.

Comment: *The authors should specifically address the following points:*
1. In Figure 1b, the authors actually used PtNPs as a cocatalyst in the hydrogen evolution experiment, but this was not indicated in the figure, which may cause misunderstanding. It is recommended that the authors redesign the figure. Could the authors determine and discuss the excited-state redox potentials of the aggregated dye systems (both oxidation and reduction potentials) and compare them with the thermodynamic potentials for H+/H2 and O2/H2O2 (or O2/H2O) couples? This comparison would clarify whether the observed photocatalytic reactions are energetically feasible and further substantiate the proposed electron-transfer mechanism.

Answer: We apologize for the misunderstanding and thank the reviewer for the comment.
First, we revised Figure 1b to include the “Pt” and left “PtNPs” in Figure 5a to clearly indicate the presence of co-catalysts.
Second, we determined the oxidation and reduction excited state potentials of DSA^{2+} and compared them with thermodynamic potentials for H^+/H_2, $O_2/O^{\cdot-}$ and some common sacrificial agents, such as ascorbic acid and amines (Supplementary Fig. S88). From these experiments, we have calculated that the oxidation excited state potential of DSA^{2+} is sufficient to allow proton or oxygen reduction. For the calculation of excited states potentials, following the guidelines by Melchiorre and co-workers,[8] we registered electrochemistry for DSA^{2+} in DMF, using ferrocene as internal standard. Furthermore, in the same solvent, we recorded the absorption spectrum, and we extrapolated the onset value (472 nm, Supplementary Fig. S87). We have further compared the excited state potentials of our system to other common photosensitizers, such as $Ru(bpy)_3^{2+}$, $Ir(ppy)_3$, Eosin Y, 4CzIPN, TiO_2, and CdS (Supplementary Fig. S88).

Revision: We have updated Figure 1 in the main text to highlight that H_2 evolution was achieved in presence of Pt co-catalyst:

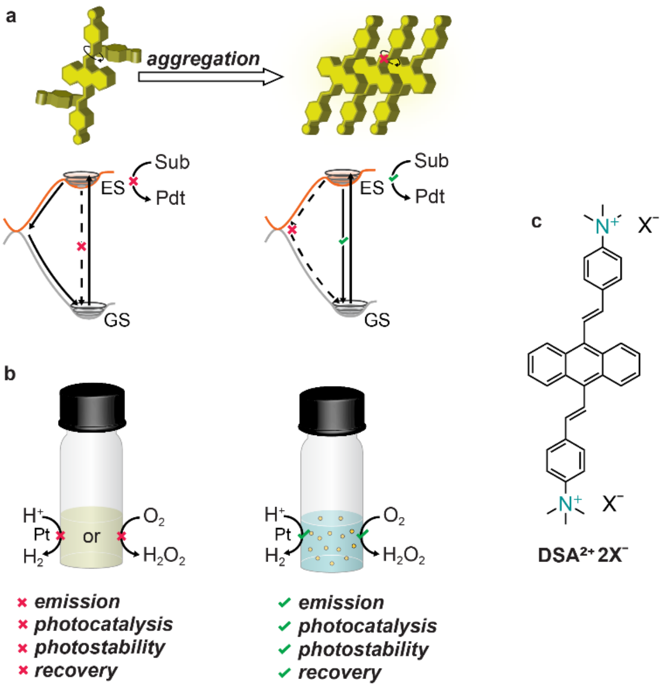

We have modified the following part in the main text (highlighted in yellow in the revised version): "The ability of the DSA^{2+} to photoreduce MV^{2+} implies that the aggregates could be also used for proton reduction reaction ($E°'$ = −0.35 V vs NHE at pH = 6). The aggregates do show promising electron transfer properties for photocatalysis, as evidenced by the calculated oxidation and reduction excited state potential when compared to commonly used photocatalysts (Supplementary Figs. S87-S88). "

In the Supporting Information, we added the following: "Cyclic voltammetry and differential pulse voltammetry of $DSAPF_6$ 1 mM were registered in DMF using $TBAPF_6$ as electrolyte (100 mM) and Ferrocene as internal standard (1 mM).

Reduction potential of the excited states was calculated employing the following equation[33]:

$$E_{red}^{*} = E_{red} + E_{0\text{-}0}(PS^{*}/PS) = 1.39 \text{ V vs Ag/AgCl}$$

The reduction peak potential (Supplementary Fig. S87) was used for E_{red} and $E_{0\text{-}0}(PS^{*}/PS)$ was estimated spectroscopically from the position of the long wavelength tail of absorption spectra (Supplementary Fig. S87). E_{red} is –1.24 V vs Ag/AgCl, while the position of the long wavelength tail is 472 nm.

Oxidation potential of the excited states were calculated employing the following equation[33]:

$$E_{ox}^{*} = E_{ox} - E_{0\text{-}0}(PS^{*}/PS) = -1.34 \text{ V vs Ag/AgCl}$$

The oxidation peak potential (Supplementary Fig. S87) was used for E_{ox} and $E_{0\text{-}0}(PS^{*}/PS)$ was estimated spectroscopically from the position of the long wavelength tail of absorption spectra (Supplementary Fig. S87). E_{ox} is 1.29 V vs Ag/AgCl, while the position of the long wavelength tail is 472 nm."

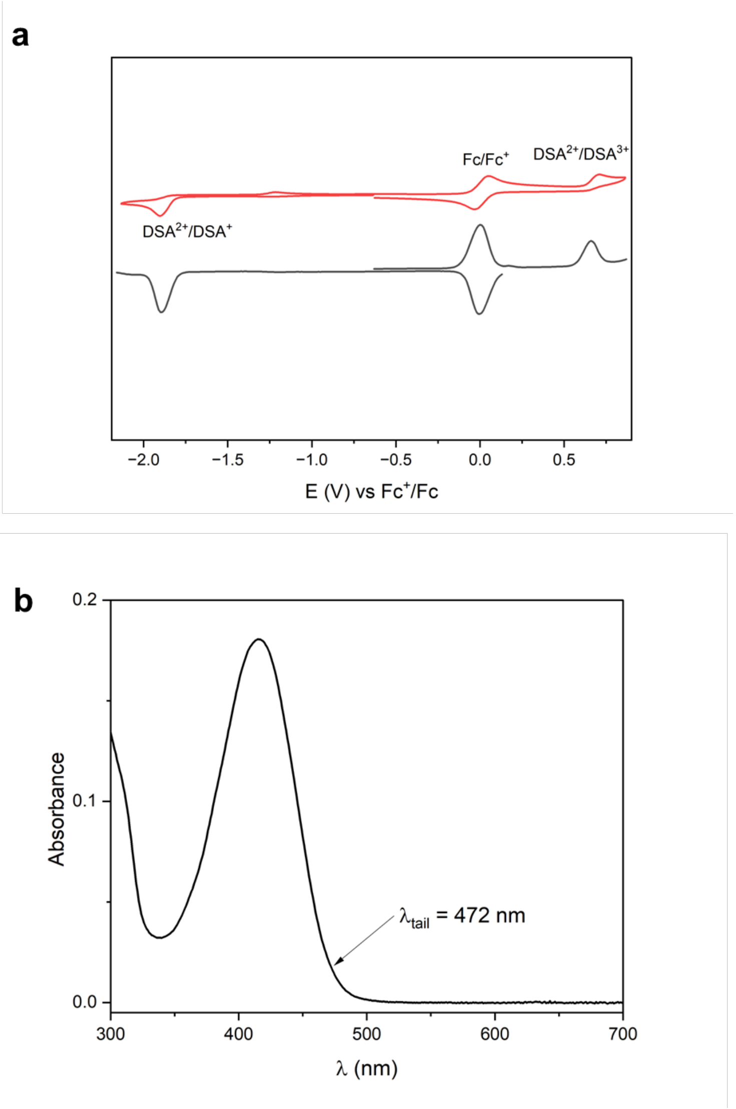

Figure S87 a) Cyclic (red trace) and differential pulse voltammetry (black traces) of $DSAPF_6$ 1 mM in DMF, using $TBAPF_6$ 100 mM as electrolyte and Ferrocene 1 mM as internal standard. Scan rate: 100 mV s^{-1}. b) Absorption spectrum of $DSAPF_6$ 10 µM in DMF and the calculated long wavelength tail value.

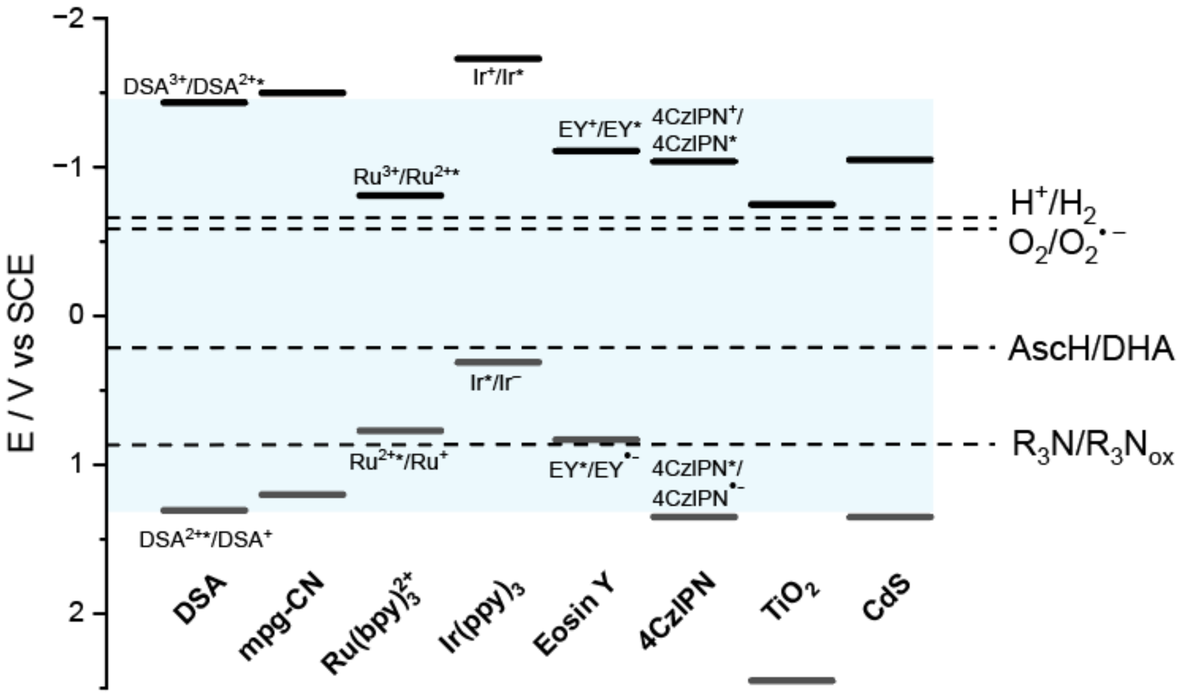

Figure S88. Calculated reduction and oxidation excited state potentials for DSA^{2+} vs SCE, compared to thermodynamic potentials for H^+/H_2, $O_2/O^{\cdot-}$, ascorbic acid and amines and relative positions of excited state potentials of DSA^{2+} compared with commonly used photocatalysts. The blue background serves as guide for the eyes from comparing the excited state potential with other common photocatalysts. Scheme adapted from reference.[38]

Comment: *2. The manuscript describes that aggregation increases fluorescence lifetime and simultaneously enhances photocatalytic activity; however, in some cases (e.g., higher DSAI concentration or increased NaI), the fluorescence lifetime decreases while activity rises. Could the authors clarify this apparent inconsistency? It raises questions about whether the observed enhancement truly originates from "excited-state stabilization" or from other factors such as intermolecular charge transfer or scattering effects in the aggregates.*

Answer: We thank the referee for pointing out this apparent discrepancy between fluorescence lifetimes and catalytic activity. In the revised version, thanks to the transient absorption spectroscopy, we observe the formation of the triplet excited state (see TAS results as response to Reviewer 2, comment 1, or below) and also confirm its reactivity both in the oxygen reduction reaction and in the hydrogen evolution reaction. The enhanced reactivity of the system therefore arises both form the singlet and the triplet localized excited states.

In addition to observing the formation of a triplet excited state in the aggregated chromophore, we examined the possibility of singlet-singlet annihilation, which could further contribute to the decrease in fluorescence lifetimes when the dyes are more aggregated. We performed laser intensity-dependence experiments and recorded TCSPC emission for DSAI aggregates (always keeping the ratio between the detected photon rate and the laser repetition rate under 2%). We observed that at lower laser intensities, the longer the lifetimes for DSAI aggregated in water, suggesting the occurrence of singlet-singlet annihilation in the sample. Moreover, when comparing two samples, both aggregated by addition of NaI, we observed shorted lifetimes at higher dye concentration.

Revision: We added this lines to the main text: "Increasing the concentration of NaI from 0.0 to 0.6 mM led to a progressive decrease in τ_{avg}, from 3.7 to 2.6 ns (Supplementary Fig. S68), which we

ascribe to a combination of triplet formation (see below) and singlet-singlet annihilation (Supplementary Figs. S69-S70).[53]"

We included these results in the Supplementary Information:

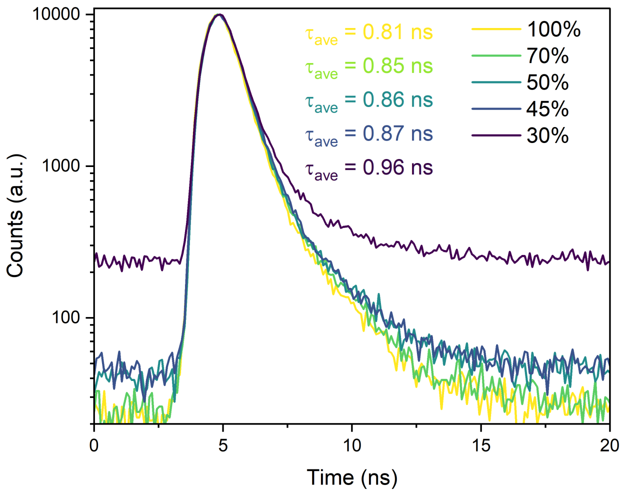

Figure S697. Time-correlated single-photon counting (TCSPC) measurements (λ_{exc} = 402.6 nm, λ_{em} = 520 nm) of DSAI 0.1 mM with NaI 1 mM by tuning the intensity of the exciting laser.

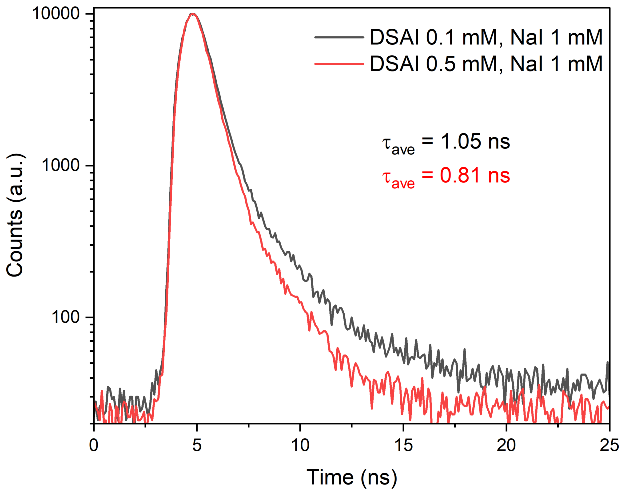

Figure S70. Time-correlated single-photon counting (TCSPC) measurements (λ_{exc} = 402.6 nm, λ_{em} = 520 nm) of DSAI 0.1 mM or 0.5 mM with NaI 1 mM.

Comment: *3. The authors emphasize that the iodide salt (DSAI) is the only sample exhibiting concentration-dependent aggregation and emission behavior. The reviewer would like to know why the addition of extra NaI leads to further aggregation of DSAI. Could the authors explain the underlying mechanism? Similarly, for compounds such as DSABr that hardly aggregate, would the addition of excess NaBr induce aggregation?*

Answer: We thank the reviewer for this insightful comment, which gives us the possibility to clarify better the focus on DSAI and the charge screening process. We chose DSAI as a proof-of-concept

molecule because it allowed us to compare the solution and the aggregate, both by increasing the concentration or by adding salts, while other salts gave either only solutions or only aggregates. We apologize for this misunderstanding.

The addition of salts (i.e. extra NaI to DSAI) leads to aggregation because they electrostatically screen repulsive forces among ammonium groups, inducing the hydrophobic collapse of the monomers and formation of nanostructures.

To further elucidate the generality of the charge screening approach, we conducted additional aggregation studies. First, as suggested by the reviewer, we studied the aggregation of the other counteranions by adding to DSABr, DSACl and $DSANO_3$ their respective sodium salt (NaBr, NaCl, and $NaNO_3$, respectively). All DSAX can be aggregated by addition of NaX salts (Supplementary Figs. S44-S46), resulting in similar photophysical and morphological properties. Second, to investigate whether the counterions remain associated or not within the aggregate, we performed TEM-EDX measurements (also included in the reply to Reviewer 2, comment 6). The micrographs disclosed that the anions are present only when the nanostructure is present and confirming that they remain associated within the aggregate (Supplementary Figs. S49-S53 in the revised manuscript).

Finally, to generalize the concept, we also studied the aggregation of DSACl through the addition of NaI, which led to the same charge screening phenomenon (Supplementary Figs. S47-S48).

Revision: We have modified the following two sentences in the results and discussion section, to better explain the focus on the DSAI dye: "Among the DSAX salts, only the iodide form (DSAI) exhibited concentration-dependent aggregation and emission behavior, appearing weakly emissive in dilute solutions and forming progressively fluorescent yellow suspensions at higher concentrations."

[…]"We further investigated the polymerization behavior of DSAI, which can exist either as molecularly dissolved species or as aggregates depending on concentration."

Furthermore, we added a new section in the main text to better describe the process of aggregation through addition of salts: "Increasing the ionic strength of the solution through salt addition screens the positive charges on the molecules, thereby reducing electrostatic repulsion and promoting hydrophobic collapse, ultimately leading to aggregation. Consistent with this mechanism, aggregation of DSAX (X = Cl^-, Br^-, NO_3^-, and I^-) can be induced by addition of the corresponding sodium salts (NaX), which increases charge screening and leads to formation of aggregates (Supplementary Figs. S44-S46). Importantly, aggregation can be induced by adding inorganic salts with counteranions different from those originally present with the dye, enabling solvated molecules to aggregate. For example, DSACl undergoes aggregation upon addition of NaI (Supplementary Figs. S47-S48), demonstrating that both ionic strength and counteranion identity contribute to aggregation. Supporting this interpretation, we observed that counteranions are not merely dissociated but remain localized within the aggregates, confirming they are embedded within the aggregates and contribute to the overall aggregation (Supplementary Figs. S48-S54)."

Regarding the Supporting Information, we added:

- DSACl with NaCl (0.05 mM and 1 M, respectively) was prepared in a vial by adding 1.1 mL of mQ-water, followed by 0.4 mL of 5 M NaCl to a 0.5 mL solution of 0.2 mM in DSACl previously dissolved in mQ-water. The sample was stirred for 15 min.
- DSACl with NaI (0.05 mM and 3 mM, respectively) was prepared in a vial by adding 1.5 mL of mQ-water, followed by 6 µL of 1 M NaCl to a 0.5 mL solution of 0.2 mM in DSACl previously dissolved in mQ-water. The sample was stirred for 5 min.
- DSABr with NaBr (0.05 mM and 0.03 M, respectively) was prepared in a vial by adding 1.5 mL of mQ-water, followed by 12 µL of 5 M NaBr to a 0.5 mL solution of 0.2 mM in DSABr previously dissolved in mQ-water. The sample was stirred for 15 min.

- $DSANO_3$ with $NaNO_3$ (0.05 mM and 0.1 M, respectively) was prepared in a vial by adding 1.5 mL of mQ-water, followed by 40 µL of 5 M $NaNO_3$ to a 0.5 mL solution of 0.2 mM in $DSANO_3$ previously dissolved in mQ-water. The sample was stirred for 15 min.
- $DSAPF_6$ samples were prepared either by sonication or nanoprecipitation. Sonicated samples were prepared by weighting DSAPF6 and adding mQ-water, followed by sonication for 10 minutes. For the nanoprecipitated sample, a solution of $DSAPF_6$ in DMSO (5 mM) was prepared and then 0.1 mL of this solution was added to a new vial containing 1.9 mL of mQ-water and under stirring. The sample was stirred for 60 min.

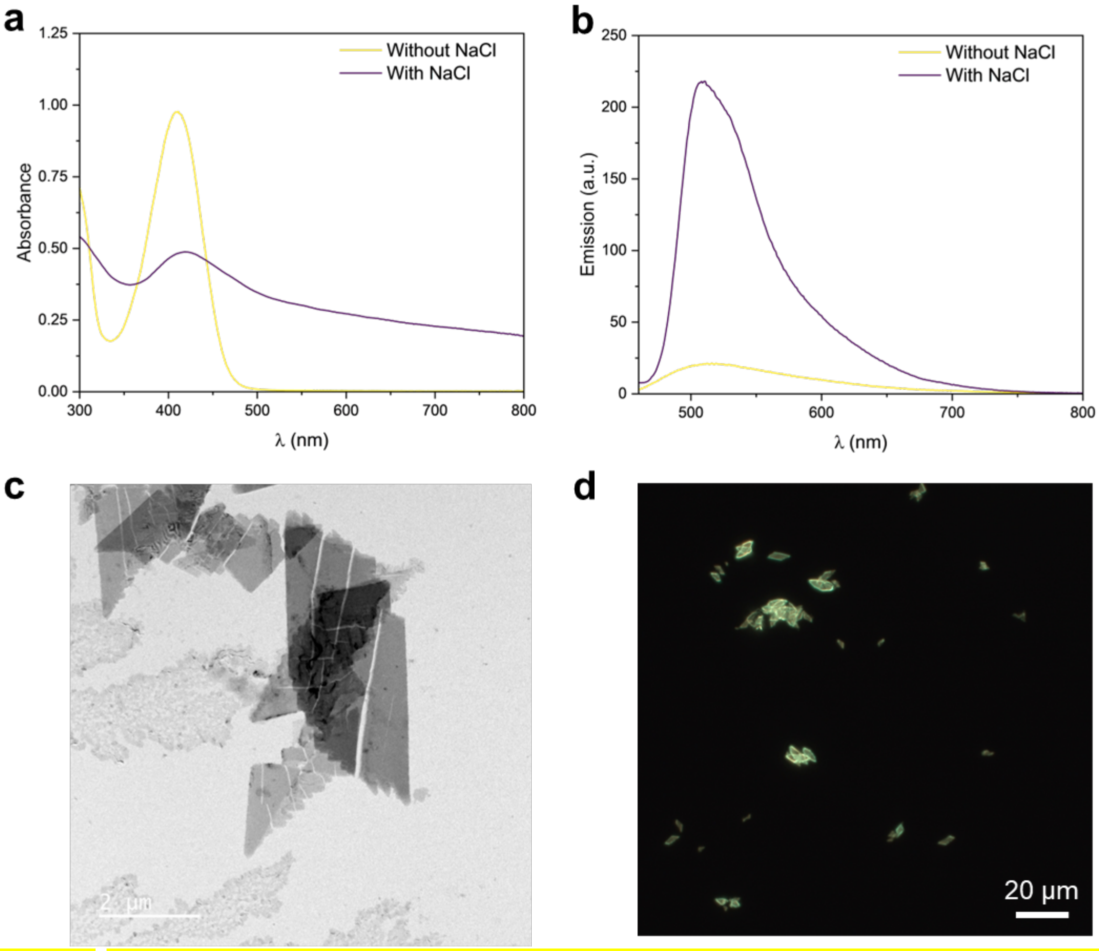

Figure S8. Study of DSACl (0.05 mM) aggregation in presence of 1 M NaCl by monitoring a) absorption spectra, b) enhancement of fluorescence emission; and c) morphological analysis of DSACl 0.05 mM in presence of NaCl 1 M by TEM and d) widefield fluorescence microscopy.

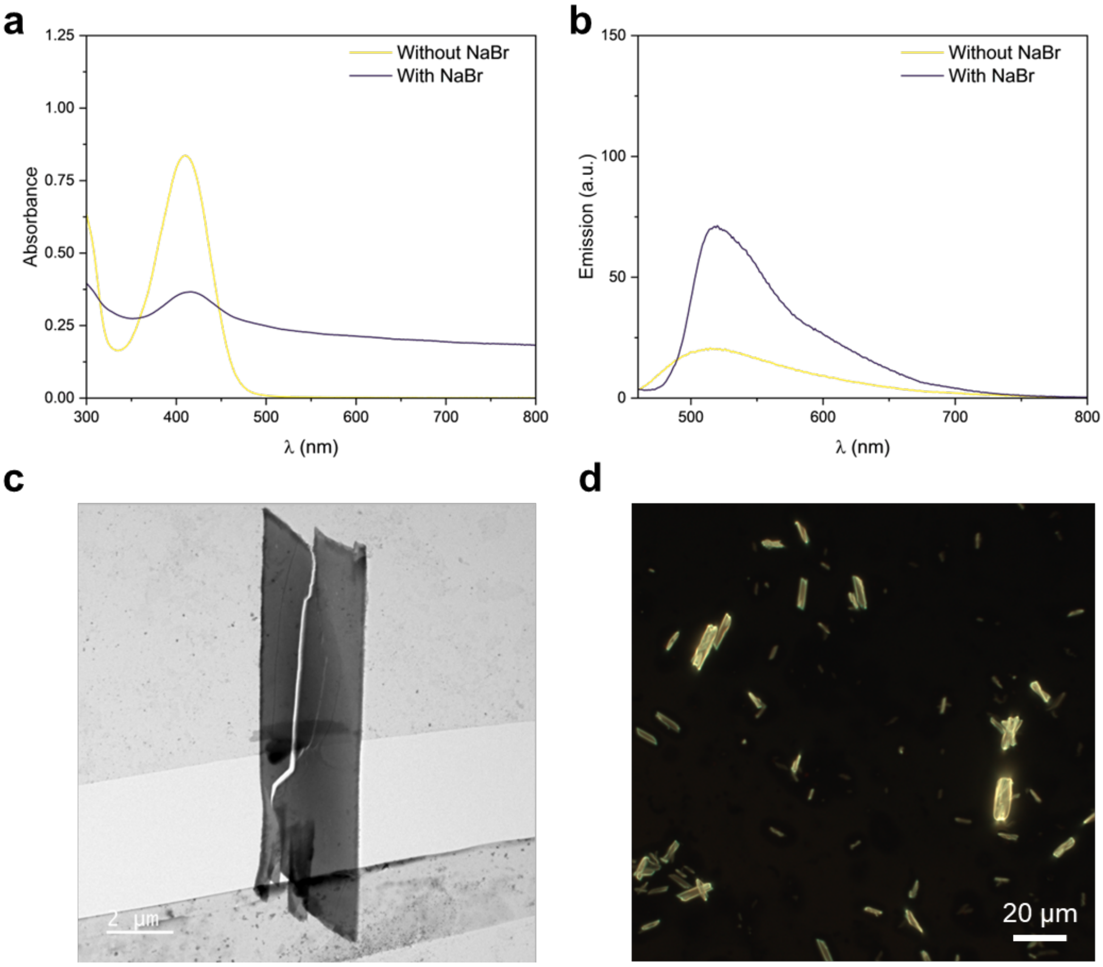

Figure S9. Study of DSABr (0.05 mM) aggregation in the absence and presence of 0.03 M NaBr by monitoring a) absorption spectra, b) enhancement of fluorescence emission; and c) morphological analysis of DSABr 0.05 mM in presence of NaBr 0.03 M by TEM and d) widefield fluorescence microscopy.

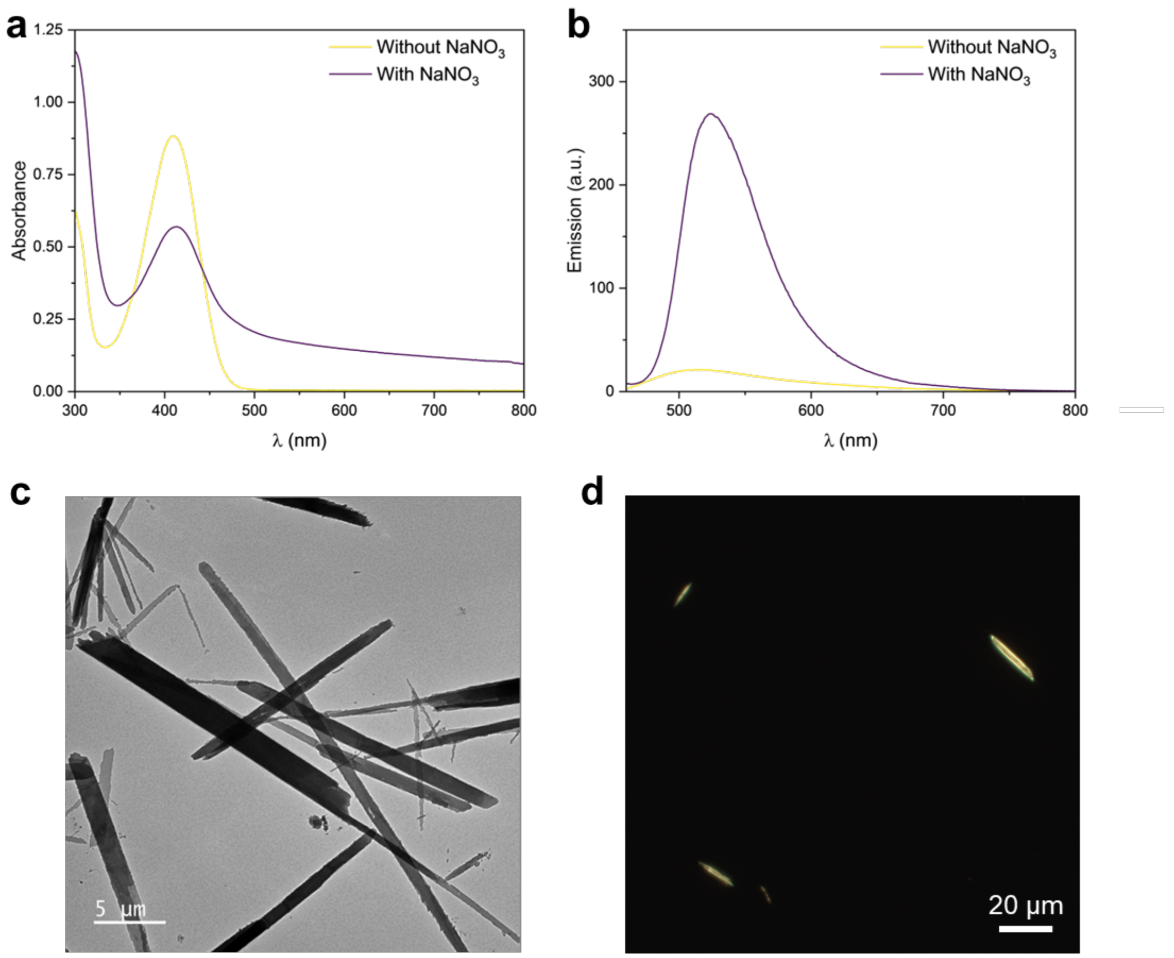

Figure S10. Study of $DSANO_3$ (0.05 mM) aggregation in the absence and presence of 0.1 M $NaNO_3$ by monitoring a) absorption spectra, b) enhancement of fluorescence emission; and c) morphological analysis of $DSANO_3$ 0.05 mM in presence of $NaNO_3$ 0.1 M by TEM and d) widefield fluorescence microscopy.

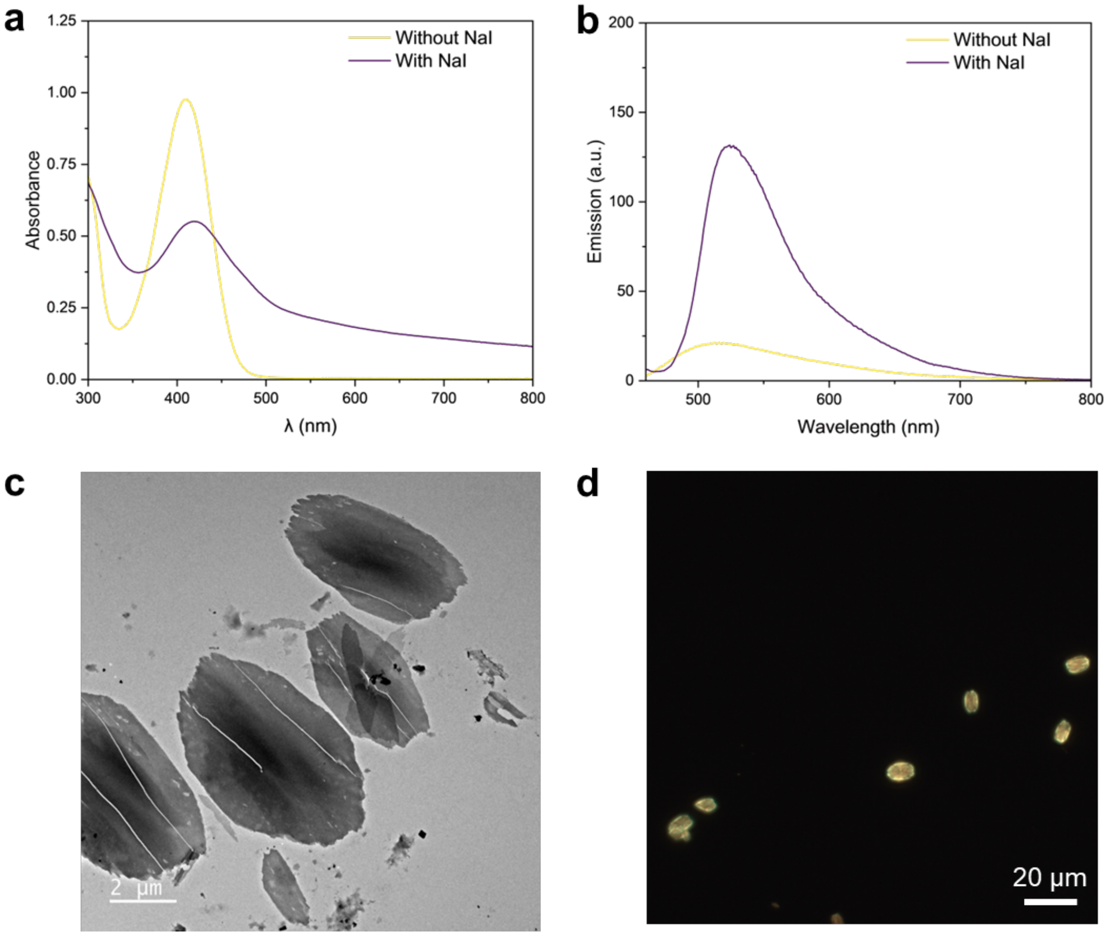

Figure S11. Study of DSACl (0.05 mM) aggregation in the absence or presence of 3 mM NaI by monitoring a) absorption spectra, b) enhancement of fluorescence emission; and c) morphological analysis of DSACl 0.05 mM in presence of NaI 3 mM by TEM and d) widefield fluorescence microscopy.

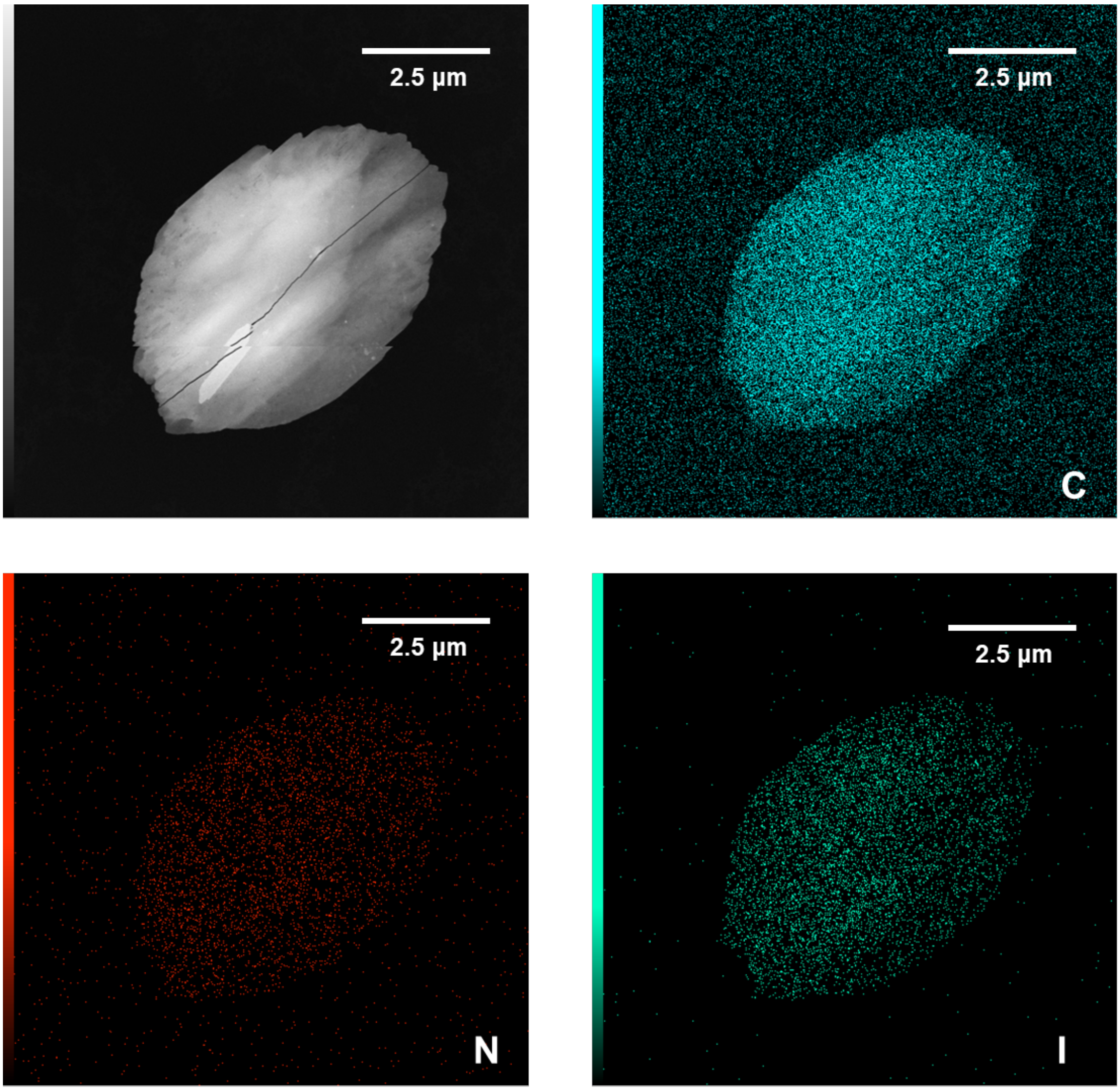

Figure S12. TEM-EDX micrographs of 0.05 mM DSACl in the presence of 3 mM NaI in mQ-water showing the content of carbon (top right), nitrogen (bottom left), and iodide (bottom right).

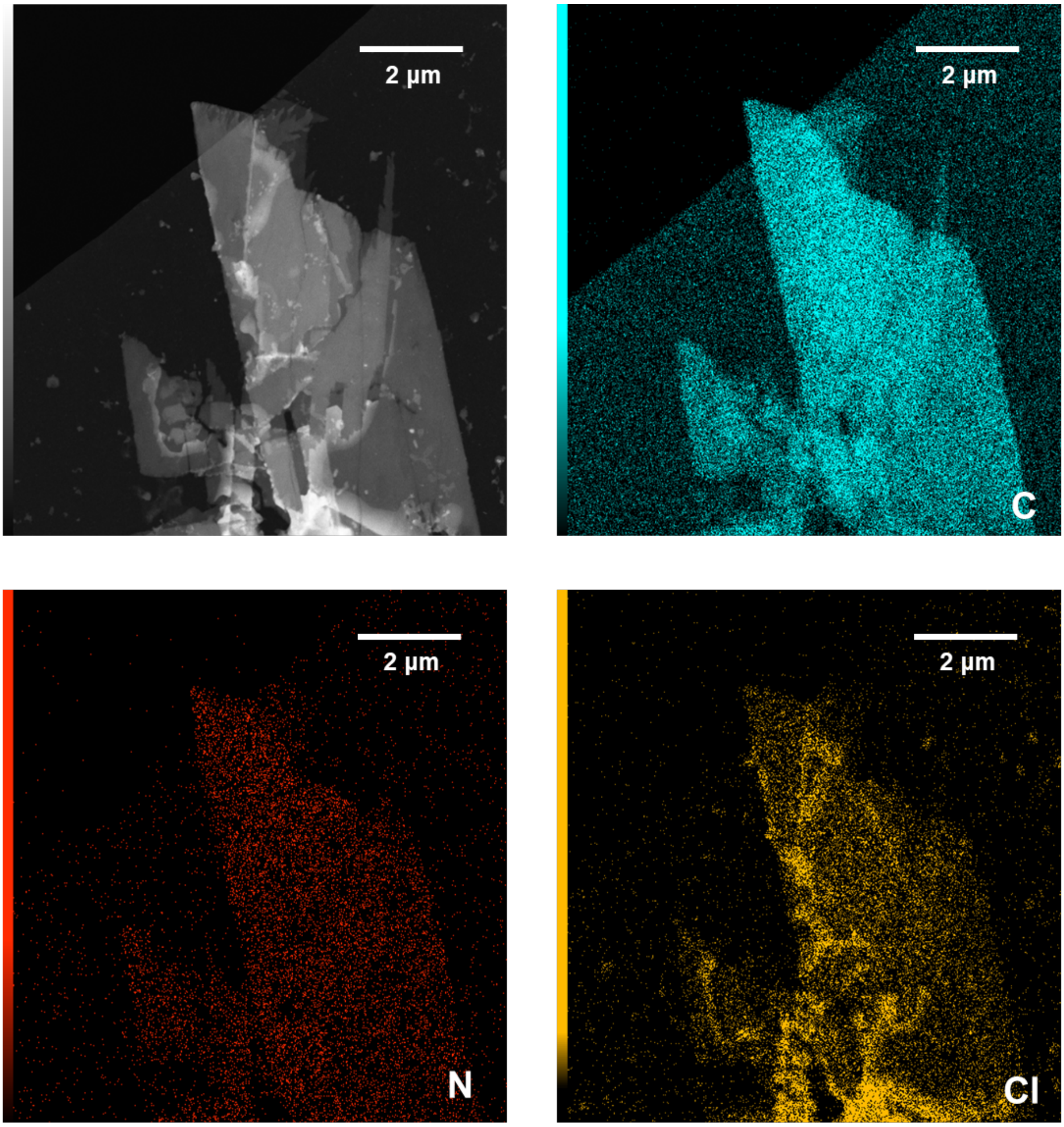

Figure S13. TEM-EDX micrographs of 0.05 mM DSACl in the presence of 1 M NaCl in mQ-water showing the content of carbon (top right), nitrogen (bottom left), and chloride (bottom right).

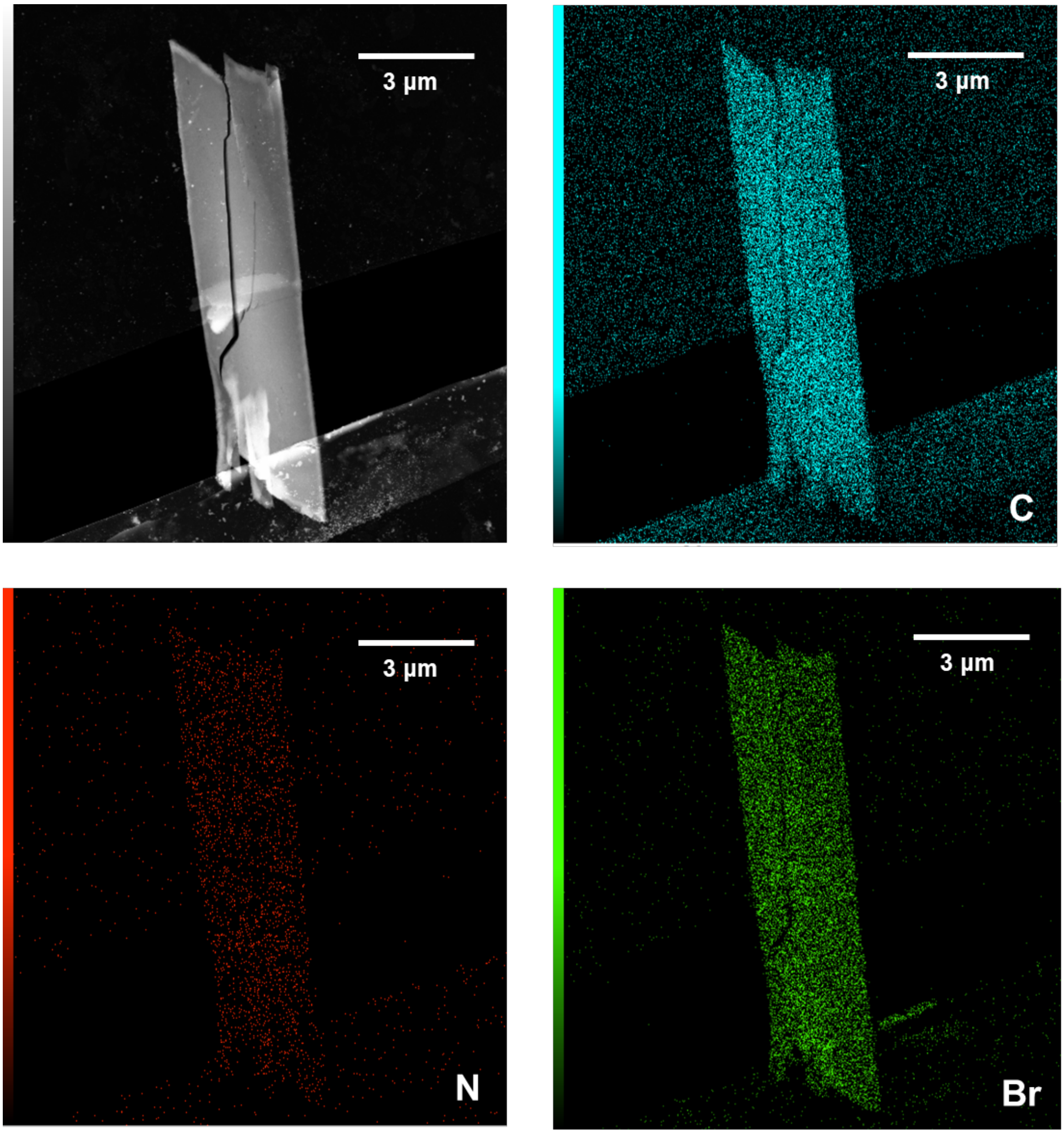

Figure S14. TEM-EDX micrographs of 0.05 mM DSABr in the presence of 0.03 M NaBr in mQ-water showing the content of carbon (top right), nitrogen (bottom left), and bromide (bottom right).

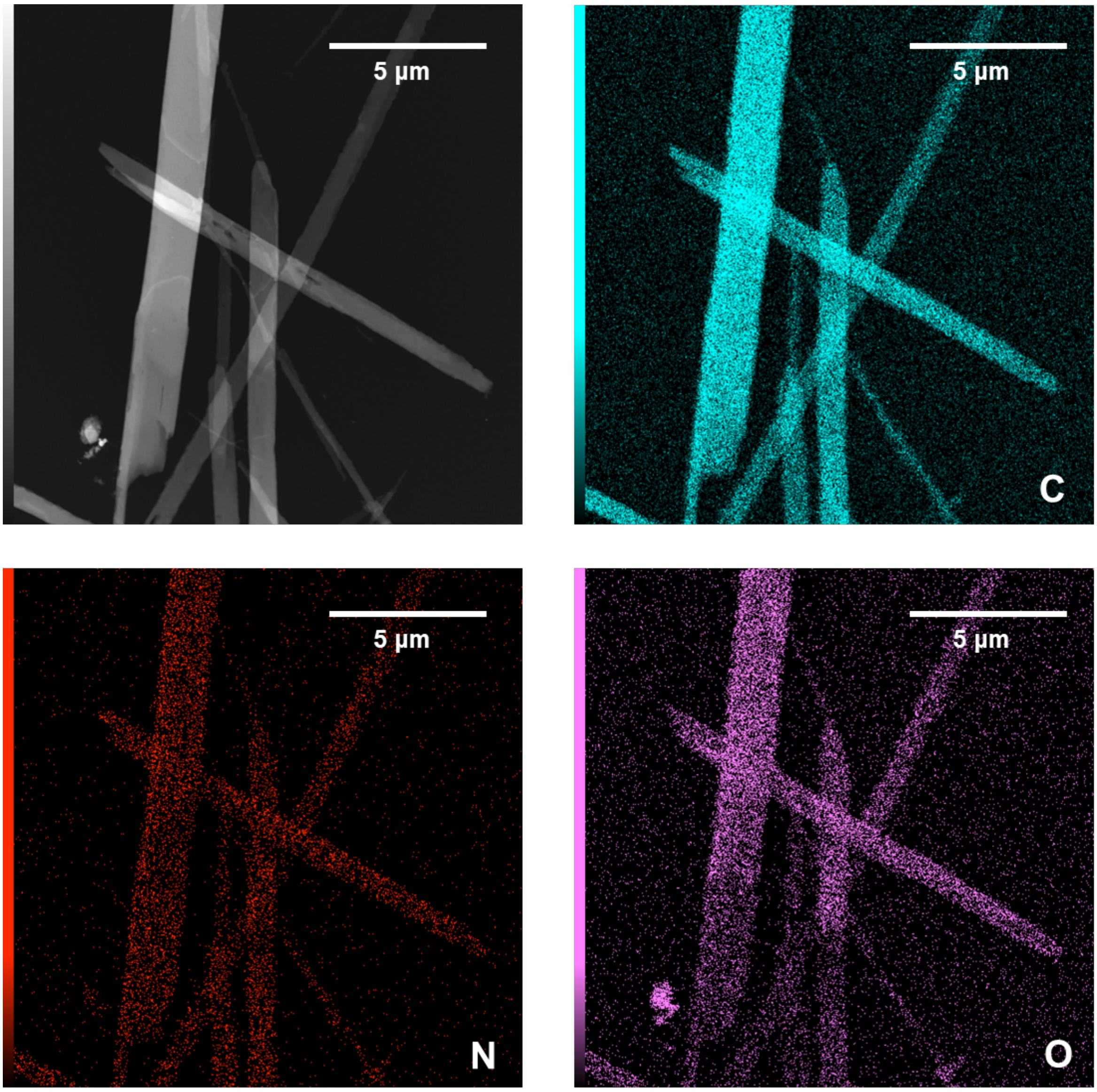

Figure S15. TEM-EDX micrographs of 0.05 mM $DSANO_3$ in the presence of 0.1 M $NaNO_3$ in mQ-water showing the content of carbon (top right), nitrogen (bottom left), and oxygen (bottom right).

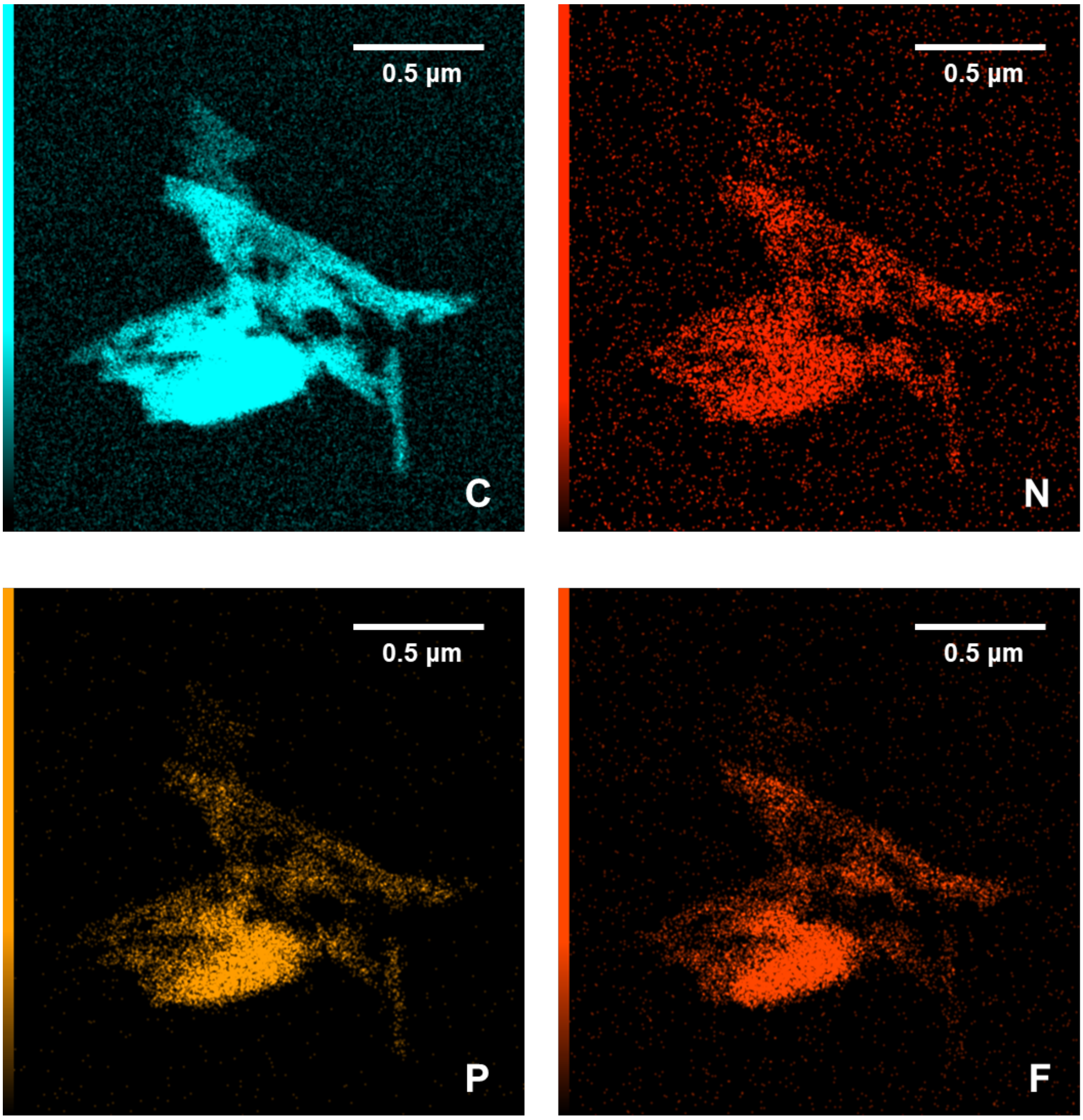

Figure S16. TEM-EDX micrographs of 0.4 mg mL^{-1} $DSAPF_6$ in mQ-water nanoprecipitated from DMSO showing the content of carbon (top left), nitrogen (top right), phosphorous (bottom left) and fluorine (bottom right).

Comment: *4. Compared with DSAI, DSAPF6 exhibits better aggregation and superior photocatalytic performance without the need for additional NaI. However, the authors mainly focus on DSAI in this work. Could they explain the reason for this choice?*

Answer: We thank the referee for their question, and we apologize that we were not clear in explaining why we chose DSAI instead of $DSAPF_6$. Our choice in focusing mainly on DSAI was dictated by the possibility of comparison between molecularly dissolved solutions and aggregated states with DSAI, which displays concentration-dependent aggregation and emission properties. In this context $DSAPF_6$ is always aggregated, also at low concentrations, which did not allow us to investigate and compare directly solutions with aggregates.

Revision: We added these lines to the main text: "Among the DSAX salts, only the iodide form (DSAI) exhibited concentration-dependent aggregation and emission behavior, appearing weakly

emissive in dilute solutions and forming progressively fluorescent yellow suspensions at higher concentrations"
[…]"We further investigated the polymerization behavior of DSAI, which can exist either as molecularly dissolved species or as aggregates depending on concentration"

Comment: *5. The authors measured the reduction potentials of the monomer and the aggregates; however, according to the cyclic voltammetry results (Figure S65), DSAI shows poor redox reversibility both in solution and in the solid state. How do the authors explain its excellent photocatalytic performance?*

Answer: The referee raises a good point about the electrochemical reversibility of anthracene and its derivatives. It is first important to note here that there is an important difference in the conditions used for electrochemistry and photocatalysis. The importance of the conditions used for anthracene derivatives is very much apparent if we consider that the cathodic wave on the return sweep can become reversible at a higher sweep rate.[9] Furthermore, also addition of acids has been reported to promote the redox reversibility of anthracene derivatives, by making the anthracene radical cations more persistent.[9]
Also, while a reversible electron transfer can be observed in electrochemistry in the absence of chemical reactions and an electrochemical irreversibility indicates a low electron transfer rate, photocatalytic reactions (either in solution or in the solid state) can be based on irreversible processes.

Comment: *6. The authors claim that DSAI exhibits nearly identical redox and photoexcited-state energy levels between the monomeric and aggregated states. This characteristic is rather uncommon—are there any known compounds with similar behavior?*

Answer: We thank the reviewer for this constructive question. As the reviewer correctly points out, looking beyond photocatalysis, a longstanding goal in photochemistry and materials science is to develop aggregated or solid-state materials that retain the well-defined photophysical properties of molecular chromophores in solution, as uncontrolled inter-chromophore interactions arising from supramolecular assembly remain a major obstacle to replacing crystalline inorganic semiconductors with organic materials in advanced optoelectronic and energy technologies.
An important recent example (now cited as reference 72 in the revised manuscript) confined organic emitters within a two-dimensional inorganic perovskite superlattice, demonstrating a "single-molecule-like aggregate" state in which near-equilibrium intermolecular distances (approximately 6 Å) suppress detrimental electronic coupling while retaining dense packing. Other examples of approaches have distanced chromophores by using sterically bulk substituents,[10,11] incorporating them into rigid and shape-persistent cages,[12] or using macrocycles to spatially and electronically isolate cationic dyes within an ionic lattice.[13]
The above examples are an overview of the approaches that have been used to transfer or retain the photophysical properties from molecules to solid state, which is indeed rare in photocatalysis.[14] We, however, prefer not to overstate this as exceptional accomplishment since many works simply do not report the comparison between solution and aggregate state, complicated by the fact that often it is not possible to have the same dye both as monomer and aggregate in water.

Revision: We have added the following discussion in the concluding remarks of the manuscript: "The excited-state energies and potentials remain nearly unchanged upon aggregation, as revealed by photophysical and electrochemical measurements. Thus, performance improvements are not due to altered energetics but to increased excited-state utilization, made possible by rigidification through crystallization. This contrasts with established crystalline dye assemblies, where redox shifts and

packing-induced band formation often dominate. In our system, the mechanistic decoupling of supramolecular structure from excited-state energetics simplifies rational design and predictive control. In this respect, aggregation-induced rigidification provides a supramolecular route that complements established covalent strategies for chromophore rigidification, including sterically constraining structures, [69]or preparing covalent organic frameworks, macrocycles, molecular cages and organic-inorganic superlattices.[17,42,70–72]"

Comment: *7. Could the authors perform femtosecond/picosecond transient absorption or time-resolved fluorescence measurements to directly track the excited-state dynamics of the monomeric and aggregated species? Such data would be essential to substantiate the proposed mechanism of localized singlet-state stabilization and suppressed non-radiative decay.*

Answer: We thank the reviewer for the important suggestion, and we have (partially) answered this as reply to Reviewer 2 (comment 1).
We also investigated the transient absorption spectroscopy of the DSAI both as solution and as aggregate (upon addition of NaI) by fs- and ns-pulsed excitation. The chromophore solutions (DSAI in water) displays a band peaked at 620 nm that shifts at 545 nm and then disappears with lifetimes of 17 ps and 79 ps attributed to fast isomerization of the styryl moieties of the DSAI. These results are in agreement with the fs-TAS spectra reported for 9,10-distyrylanthracene and derivatives.[7]
fs-TAS of the DSAI aggregates show significant differences: there is no shift of the excited state absorption (ESA) bands and the kinetic analysis shows a fast component of 21 ps, similar to that previously observed for the monomeric species, but the second component is slower with 320 ps lifetime, as expected by the rigidification of the structure that slows down decays related to photoisomerization pathways. At the end of the time range of measurement (7 ns) an ESA band peaked at 539 nm is observed, compatible with the formation of the lowest triplet excited state. In the ns-TAS experiment, aggregates show a decay lifetime of 178 ns. Importantly, the TAS features of DSAI aggregates mirror those reported in literature for unsubstituted 9,10-distyrilanthracene monomers in a rigid environment, where its intramolecular motion is restricted.[7]
We have also performed additional mechanistic investigations and found that the triplet state, as well as the singlet state, are both quenched by acceptors, either O_2 or MV^{2+}. These results showed that localized excited states (singlet and triplet) are both involved in photocatalysis.

Revision: We updated the main text and Figure 5 (5g−i): "In all cases, the fluorescence decays show lifetimes in the nanosecond range, pointing to formation of singlet excited states. This prompted us to further probe the excited states of DSAI solution and aggregates through femtosecond and nanosecond transient absorption (fs-TAS and ns-TAS, respectively) experiments. The monomeric chromophore in solution displayed a band peaked at 620 nm that shifts at 545 nm and then disappeared with lifetimes of 17 ps and 79 ps, respectively, attributed to fast isomerization of the styryl moieties of the DSAI (Supplementary Fig. S71). These results are in agreement with fs-TAS spectra reported for unsubstituted 9,10-distyrylanthracene and derivatives.[54] For DSAI aggregates, instead, fs-TAS showed significative differences: no shift of the excited state absorption (ESA) bands were observed and a kinetic analysis shows a fast component of 21 ps, similar to that previously observed for the monomeric species, but the second component is slower with 320 ps lifetime, as expected by the rigidification of the structure that slows down decays related to photoisomerization pathways and enhances excited state stabilization (Supplementary Fig. S72). At the end of the time range of measurement (7 ns) an ESA band peaked at 539 nm persisted, compatible with the formation of the lowest triplet excited state, whose presence was confirmed in the ns-TAS experiment (Supplementary Fig. S73), although no phosphorescence was observed in solid state measurements (at 77 K, Supplementary Fig. S74). Importantly, the emission and transient absorption spectra of DSAI aggregates mirror those reported for the monomeric dye in rigid environments (Supplementary Fig. S75),[53] further supporting the formation of localized excited states – both singlet and triplet – in

the aggregated state. These results show that aggregation enhances photocatalysis by increasing the population of localized excited states. The fact that three distinct aggregation triggers – counterion identity, concentration, and ionic strength – produce parallel trends in both emission and ROS generation supports the observation in which aggregation restricts intramolecular motion and increases the probability of productive excited-state processes."

[…]"Additionally, singlet oxygen emission and anthracene-9,10-dipropionic acid photodegradation experiments ruled out singlet oxygen sensitization (Supplementary Fig. S80). Both singlet and triplet excited states of aggregates are involved in electron transfer to O_2 and superoxide formation, as evidenced by quenching of fluorescence emission (Supplementary Fig. S81) and quenching of the triplet state observed by ns-TAS (Supplementary Fig. S73)."

[…] "Fluorescence experiments revealed that salt-induced aggregation leads to a marked increase in emission with Φ_{FL} going from 0.8% to 14% (Fig. 5g and Supplementary Fig. S102 for excitation-emission maps). Quenching experiments (Fig. 5h,I and Supplementary Fig. S103)[53] showed that the excited states of the aggregated DSAI are quenched in the presence increasing concentration of MV^{2+}, observed both for the singlet ($k_{SV}{}^{S}$ = 222 M^{-1} from fluorescence titration) and the triplet state ($k_{SV}{}^{T}$ = 32 M^{-1} from ns-TAS titration), consistent with the involvement in photocatalysis of both localized excited states "

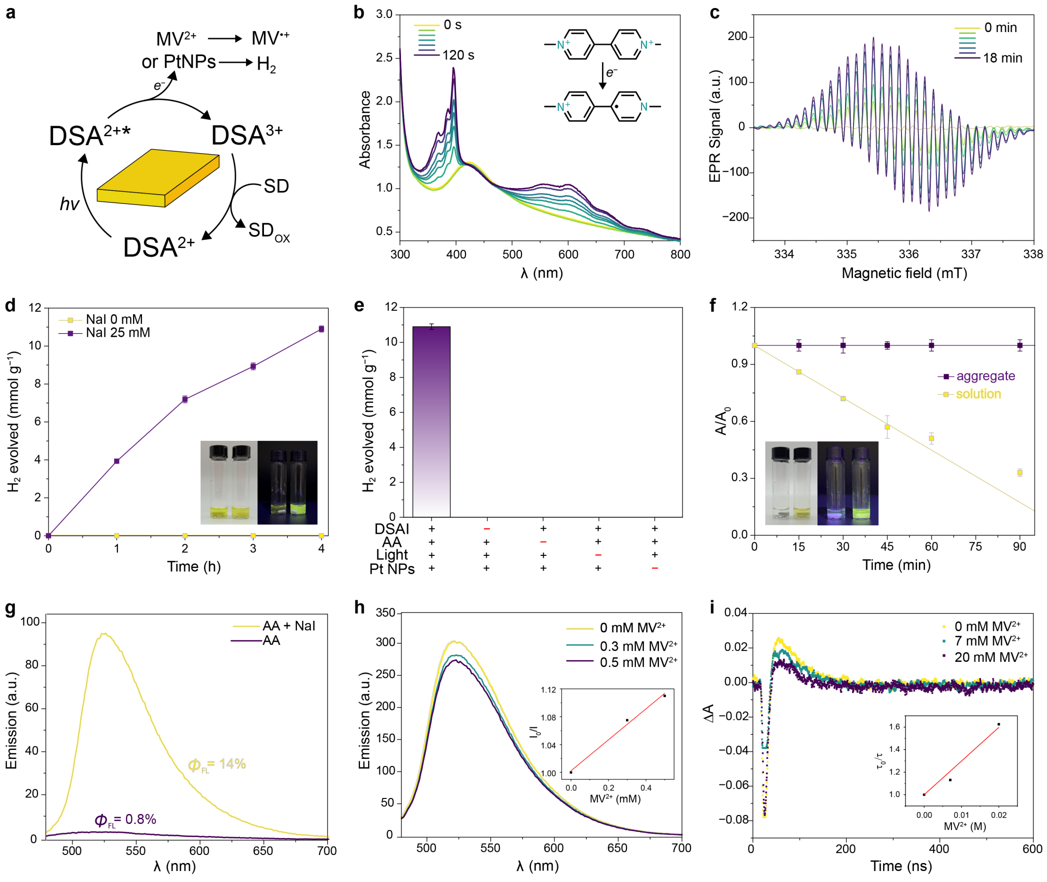

Figure 5. Light-driven hydrogen evolution by DSA^{2+} nanostructures. **a**, Proposed photocatalytic mechanism for methyl viologen (MV^{2+}) or hydrogen evolution in presence of a co-catalyst (PtNPs, platinum nanoparticles) and sacrificial donor (AA, ascorbic acid). **b**, Ultraviolet–visible absorption spectra over time of a mixture of DSAI (1.0 mM) and MV^{2+} (10 mM) under 450 nm (LED, 700 mW cm^{-2}) irradiation. **c**, Electron paramagnetic resonance (EPR) spectra over time of a mixture of DSAI (1.0 mM) and MV^{2+} (10 mM) under 450 nm (LED, 700 mW cm^{-2}) irradiation. **d**, H_2 light-driven evolution over time by irradiation of DSAI solution (yellow trace) or nanostructures (NaI 25 mM, purple trace) in ascorbic acid (1.0 M, pH = 4.0), in presence of PtNPs (8% mol) with white light (LED, 100 mW cm^{-2}). Inset shows DSAI samples in AA without (left) and with (right) NaI under daylight and UV light. **e**, Control experiments for H_2 produced by DSAI. **f**, Absorbance over time by irradiation

of DSAI solution or DSAI nanostructures in ascorbic acid. Inset shows irradiated DSAI samples in AA without (left) and with (right) NaI under daylight and UV light. **g,** Emission spectra of DSAI 0.1 mM dissolved in ascorbic acid 1.0 M (pH = 4.0) and aggregated in ascorbic acid 1.0 M (pH = 4.0) with NaI 25 mM (λ_{exc} = 395 nm, at isoabsorbance). **h** DSAI 0.1 mM with NaI 0.1 M emission quenching with different MV^{2+} concentration (λ_{exc} = 470 nm at isoabsorbance). **i,** Transient absorption kinetic traces at 420 nm of an aqueous solution of aggregates of DSAI 0.05 mM and 2 mM of NaI (yellow trace) and after addition of Methyl Viologen ([MV^{2+}] = 7 mM (green trace) and 20 mM (purple trace). λ_{exc} = 355 nm. $A_{355\ nm}$ = 0.31, 1 cm optical path, 4.4 mJ per pulse.

The previous Figs. 5g−i are now Supplementary Figs. S102 and S103 in the revised manuscript.

Regarding the Supporting Information we added: **Transient absorption spectroscopy.** Transient absorption in the femtosecond range was performed by means of an Ultrafast Systems HELIOS (HE-VIS) femtosecond transient absorption spectrometer by using, as an excitation source, a Coherent Astrella Laser, combined with an OPERA SOLO optical parametric amplifier (pulse width: 32 fs, 1 kHz repetition rate, selected output wavelengths: 420 nm). The Astrella system was composed of a Coherent Vitara™ that includes a modelocked Ti:Sapphire oscillator cavity pumped by the Coherent Verdi™ G-Series, a continuous-wave OPSL (optically pumped semiconductor laser) green laser, a Ti:Sa regenerative amplifier pumped by an intracavity-doubled, a DPSS (diode pumped solid state, REVOLUTION) Q-switched laser with a second-harmonic generator. Operating at 527 nm and a 1-kHz repetition rate, pump power for the regenerative amplifier module, an optical pulse stretcher, and an optical pulse compressor. The overall temporal resolution of the system is 300 fs. Air-equilibrated solutions in 0.2 cm optical path cells were analysed under continuous stirring. The pump energy on the sample was 4 μJ/pulse at 420 nm. Surface Xplorer V4 software from Ultrafast Systems was used for the data acquisition and analysis. The 3D data surfaces were corrected for the chirp of the probe pulse prior to the analysis. Lifetimes were taken as average of values derived from the fitting of several decays at different wavelengths. Errors on lifetimes were estimated as average of the errors reported by the fitting software for each lifetime. Steady-state absorption spectra were registered before and after transient absorption experiments to check the photostability of the investigated system.

The LP980 setup from Edinburgh Instruments equipped with an Nd:YAG laser from Litron (Nano) was employed for transient absorption and time-resolved emission spectroscopy. The frequency-tripled output with a wavelength of 355 nm served as the excitation source. The laser pulse duration was 7 ns, and the pulse frequency was 5 Hz. The typical pulse energy used for transient absorption and emission studies was 4.4 mJ. Detection of transient absorption spectra occurred by kinetic traces detected at selected wavelengths were recorded using a photomultiplier tube. The spectroscopic experiments were performed at 293 K using a cuvette holder that allows temperature control.

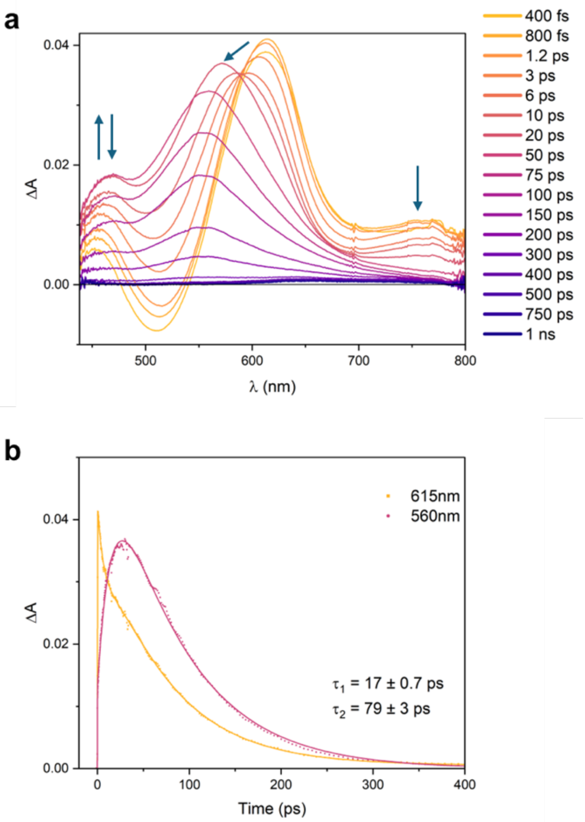

Figure S7117. a) Transient absorption spectra at different delays of an aqueous solution of DSAI 0.05 mM. The arrows indicate the spectral changes associated with the different processes. b) Transient absorption kinetic traces at significant wavelengths together with the corresponding fitting decays (solid lines). λ_{exc} = 420 nm. $A_{420\ nm}$ = 0.22, 0.2 cm optical path, 4 μJ per pulse.

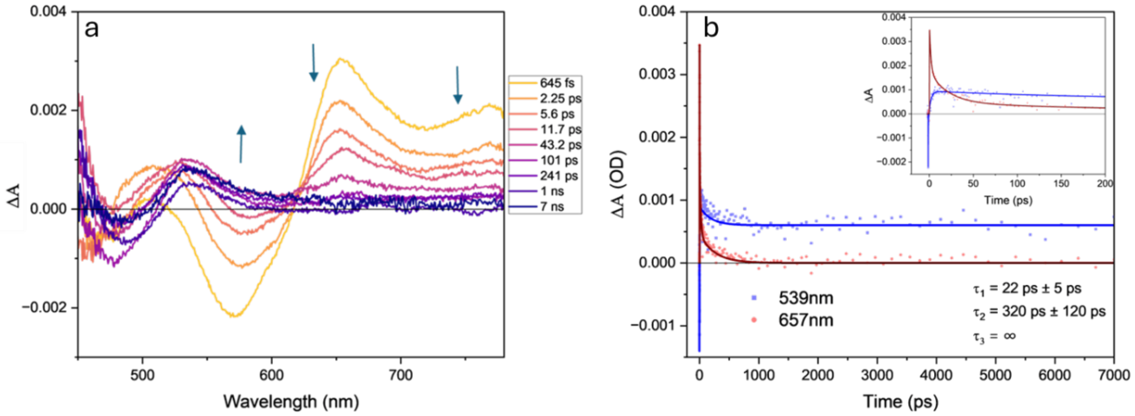

Figure S72. a) Transient absorption spectra at different delays of an aqueous solution of aggregates of DSAI 0.05 mM and 2 mM of NaI. The arrows indicate the spectral changes associated with the different processes. b) Transient absorption kinetic traces at significant wavelengths together with the corresponding fitting decays (solid lines). λ_{exc} = 420 nm. $A_{420\ nm}$ = 0.22, 0.2 cm optical path, 4 μJ per pulse.

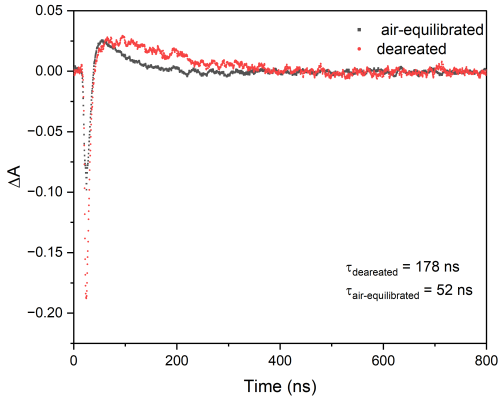

Figure S73. Transient absorption kinetic traces at 420 nm of an aqueous solution of aggregates of DSAI 0.05 mM and 2 mM of NaI in air-equilibrated (black) and deareated (red) condition. λ_{exc} = 355 nm. $A_{355\ nm}$ = 0.31, 1 cm optical path, 4.4 mJ per pulse.

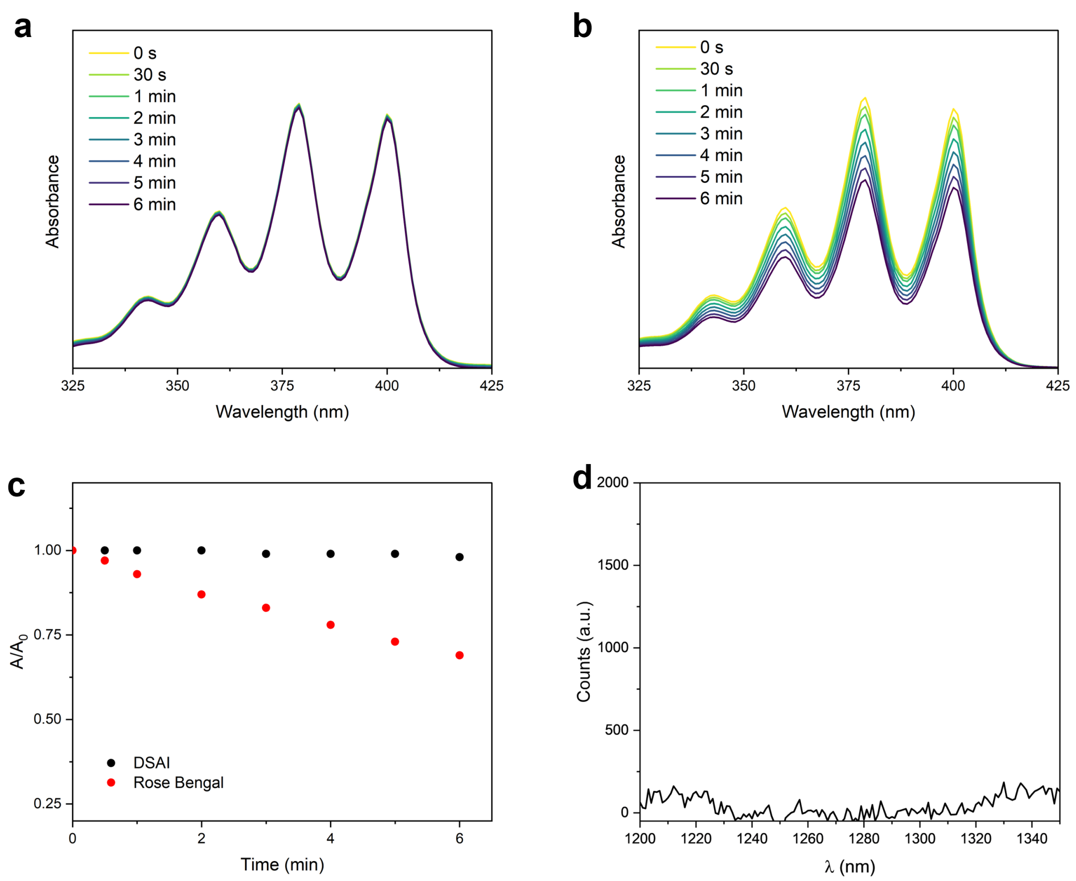

Figure S18. Singlet oxygen sensitization evaluated using 9,10-anthracenedipropionic acid (ADPA) as an indicator. a) ADPA UV-Vis spectra evaluated from 0 to 6 minutes irradiation under white light 28 mW cm^{-2} in presence of DSAI 10 μM, NaI 15 mM, ADPA 110 μM; b) ADPA UV-Vis spectra evaluated from 0 to 6 minutes irradiation under white light 28 mW cm^{-2} in presence of Rose Bengal 10 μM and ADPA 110 μM; c) normalized absorption intensity of ADPA at 378 nm after photodecomposition by singlet oxygen upon white light irradiation. d) Singlet oxygen emission spectrum in presence of DSAI 0.1 mM and NaI 5 mM in D_2O.

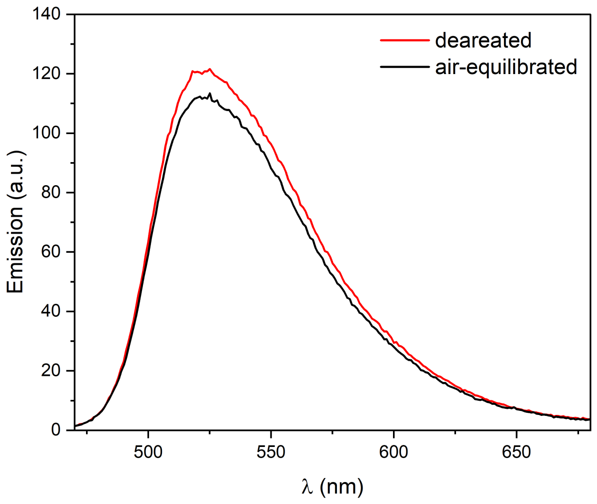

Figure S81. Emission spectra of DSAI 0.1 mM with NaI 0.1 M under different atmospheres (λ_{exc} = 355 nm, at isoabsorbance).

Comment: *8. Could the authors evaluate the generality of the photocatalytic behavior by testing additional substrates or redox reactions beyond H2 and H2O2 generation (e.g., organic transformations or alternative electron acceptors/donors)? Demonstrating catalytic versatility would strengthen the claim that aggregation-induced photocatalysis represents a broadly applicable design principle rather than a system-specific phenomenon.*

Answer: We thank the referee for the insightful suggestions, which helped us further demonstrate the generality and versatility of our system and AIP phenomenon.
We found that of our system can be further exploited in relevant photocatalytic oxidations, such as (*i*) glycerol oxidation to glyceraldehyde and (*ii*) 4-(methylthio)phenol oxidation to the respective sulfoxide (4-(methylsulfinyl)phenol). We also performed experiments with different sacrificial agents for H_2 and H_2O_2 generation, and with different co-catalyst for H_2 evolution.

Revision: We added these lines highlighted in yellow to the main text: "The ability to generate superoxide and hydrogen peroxide under ambient conditions, with no metal catalyst, highlights the potential of AIP-based supramolecular systems for sustainable photocatalysis. We also demonstrate this by using DSA^{2+} in light-driven oxidation of organic substrates such as 4-(methylthio)phenol in the presence of oxygen (Supplementary Fig. S85), as well as glycerol, an abundant feedstock that can be reconverted to valuable chemicals.[58] DSA^{2+} aggregates (DSAI 0.1 mM, NaI 25 mM) displayed excellent activity for glycerol oxidation to glyceraldehyde under white light irradiation (Supplementary Fig. S86)."

Regarding the Supporting Information, we added: "**Glycerol oxidation.** Samples were prepared in 9.0 mL screw cap vials (VWR®, 61×16.6 mm, 548-0821A) equipped with a micro stir bar (VWR® 3×8 mm, 442-4520). Vials were sealed with silicone/PTFE septum (VWR, 13 mm, red/grey, PTFE, 2.0 mm, 50° hardness, 548-0487A) and cap (VWR, screw cap, ND15, openTop, PP, 548-2416A). The preparation of the sample started with the making of a 0.1 mM stock solution of DSAI in glycerol 40 mM and TEMPO 7.5 mM. Then, 975 µL of this solution (0.1 µmol of photosensitizer) were taken and placed in a 9.0 mL screw cap vial, where 25 µL of a stock solution of NaI 1 M was added. Vials were then purged for 15 minutes with O_2 by using steel needles through the septum as inlet (longer needle inside the solution) and outlet (shorter needle in the headspace). After purging for the

specified time, the purging was stopped, the long needle was placed in the headspace, and then the short one was removed. The vials were then illuminated under stirring at 300 rpm using the homebuilt photoreactor made of white light LEDs with a light intensity of 100 mW cm^{-2} (measured using an Optical Power Meter PM100D at 450 nm with Optical Sensor S120VC from Thorlabs). Error bars indicate the standard error of the mean, calculated from two or three runs. Control experiments were conducted to assess the necessity of each component of the system: experiments without (1) TEMPO, (2) glycerol, (3) white light irradiation, (4) oxygen and (5) photosensitizer were performed. Glyceraldehyde detection and quantification was performed by high performance liquid chromatography (HPLC) using an Agilent Technologies 1260 Infinity II LC system coupled to diode array detector; the instrument is equipped with Hi-Plex H column 300×7.7 mm. Condition of analysis: $[H_2SO_4]$ = 5 mM in milliQ water as eluent, isocratic gradient, flow rate 0.7 mL·min^{-1}, injected volume 20 µL, temperature 50 °C."

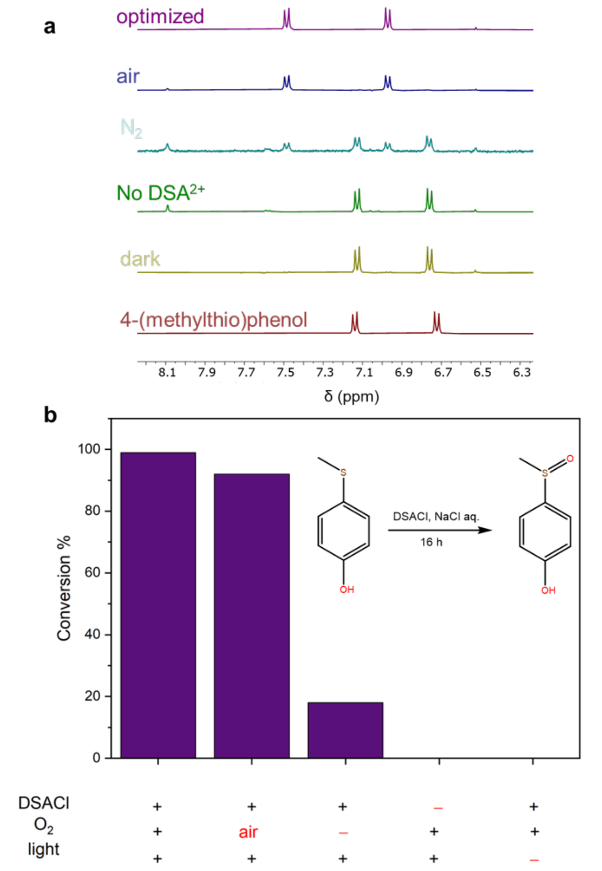

Figure S195. Photocatalytic oxidation of 4-(methylthio)phenol to 4-(methylsulfinyl)phenol using DSACl aggregates in water a) ^{1}H NMR traces, b) calculated conversion. Samples and controls were run with DSACl 1 mM in presence of NaCl 0.9 M in mQ-water, O_2 atmosphere for 16 hours under 415 nm irradiation (140 mW cm^{-2}). Product was quantified using NMR compared to internal standard 1,3,5-trimethoxybenzene in DMSO-d_6.

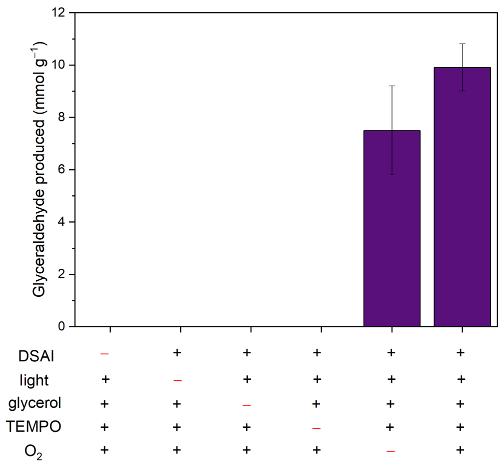

Figure S86. Glycerol oxidation to glyceraldehyde performed by DSAI 0.1 mM in presence of NaI 25 mM, glycerol 40 mM and TEMPO 7.5 mM, under white light irradiation (100 mW cm^{-2}) for 16 hours. TEMPO is acting as both a redox-mediator and a catalyst for the oxidative transformation.

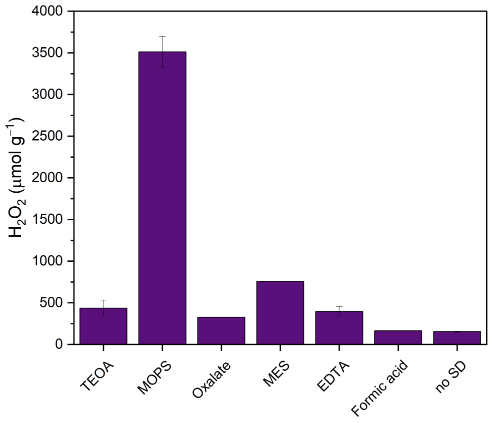

Figure S83. H_2O_2 produced by DSACl 0.5 mM, with NaCl 0.9 M in different sacrificial agents under 415 nm irradiation (140 mW cm^{-2}) after 4 hours. The higher activity observed with MOPS is attributed to more facile aggregation in the presence of this buffer.

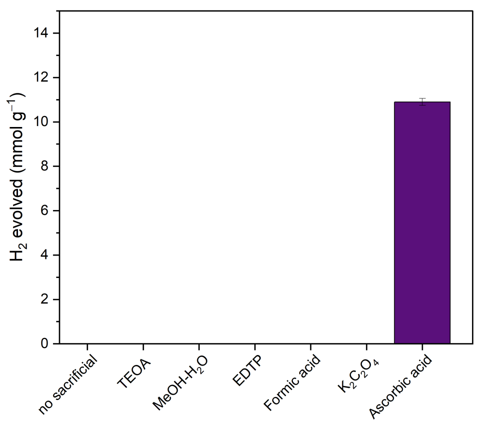

Figure S96. Different sacrificial agent tested for DSAI photosensitized H_2 evolution (DSAI 0.1 mM, PtNPs 8% mol, 4 hours under white light 100 mW cm^{-2}).

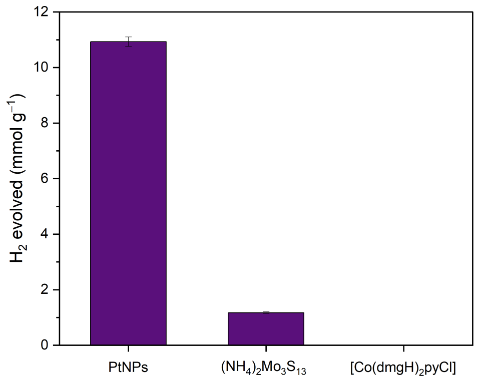

Figure S9207. Photosensitized H_2 evolution (DSAI 0.1 mM in ascorbic acid 1.0 M, pH = 4.0, NaI 25 mM), 4 hours under white light (100 mW cm^{-2}) with different co-catalyst 8% mol (PtNPs, $(NH_4)_2Mo_3S_{13}$, $[Co(dmgH)_2pyCl]$).

Reviewer #4

1. Summary of the key result: Dorđević et al. present an interesting study on photocatalysis triggered by the aggregation of certain supramolecular dye polymers.The authors propose that the photocatalytic performance induced by aggregation is not due to the well-established electronic coupling or altered redox potentials of dyes within the aggregates. Instead, they suggest that it is attributable to localized emissive singlet excited states, facilitated by the rigidification of the dye framework, imposed by packing forces.

Answer: We thank the reviewer for the accurate summary and for highlighting the key mechanistic insight – namely, that photocatalytic function arises from localized singlet and triplet excited states stabilized by aggregation, rather than from electronic coupling or redox shifts.

Comment: *2. Originality and significance: if not novel, please include reference*
The novelty of this work lies in its provision of new insights into aggregation-induced photocatalysis phenomena, suggesting a clear departure from the commonly established supramolecular photocatalysis via exciton delocalization and long-range charge separation. The authors conducted a thorough investigation into the relevant processes and mechanisms, which holds significant promise for advancing the field of supramolecular photocatalysis.

Answer: We sincerely thank the reviewer for their support for the publication of our manuscript and for their positive and encouraging evaluation of the novelty of our work. Below, we provide detailed responses to each constructive suggestion, which have helped us to further improve our manuscript.

Comment: *3. Data & methodology: validity of approach, quality of data, quality of presentation. High*

Answer: We thank the reviewer for the positive assessment of our experimental design, data quality, and overall presentation.

Comment: *4. Appropriate use of statistics and treatment of uncertainties. Yes*

Answer: We appreciate the reviewer's approval.

Comment: *5. Conclusions: robustness, validity, reliability. In part. There are key areas where further improvement and exploration are warranted (see below; 6. Suggested improvements).*

Answer: We thank the reviewer for this constructive assessment. We agree that further clarification and contextualization strengthen the conclusions, and we have carefully addressed the points raised in the suggested improvements section.

Comment: *6. (page 11; last paragraph): The time-correlated single photon counting (TCSPC) experiments (see also Fig. S56-S58) appear to be quite confusing and contradictory to the proposed hypothesis. While aggregation of the DSAI dye caused by charge-diffuse anions extended (albeit slightly) the singlet-state average lifetime (τavg), the opposite trend was observed when aggregation was triggered by increasing either the concentration of DSAI or ionic strength (addition of NaI). One would expect that stabilization of the excited-state by rigidification (i.e., suppression of non-radiative decay) will invariably result in an increase of τavg. As demonstrated by the clear visual indication*

(see Fig. S56-S57), a rapidly decaying component in proximity to the pulse profile manifests upon aggregation. This may suggest the existence of an additional reaction channel (e.g., charge delocalization) that depopulates rapidly the excited state, thereby challenging the conclusion that the system operates through localized singlet excited states stabilized by rigidification. It is imperative for the authors to provide a comprehensive analysis of the multiexponential fluorescence decay curves (contribution analysis of all lifetimes to the τavg).

Answer: We thank the referee for their comment. As they correctly pointed out there is an apparent inconsistency, as we observed a decrease in lifetimes either increasing the dye concentration or the ionic strength, thus increasing the presence of aggregates in the sample. In addition to formation of the triplet state (see also nanosecond transient absorption spectroscopy sections), we also examined the possibility of singlet-singlet annihilation, which would explain the decrease in lifetimes with increasing dye aggregation. We performed laser intensity-dependence experiments for the lifetimes of DSAI aggregates, always being sure that the ratio between the detected photon rate and the laser repetition rate is kept under the 2%. Indeed, TCSPC experiments performed at different intensities of exciting laser, reveal that lower intensities of the laser result in longer lifetimes for DSAI aggregated in water, pointing out the presence of singlet-singlet annihilation in the sample (Supplementary Fig. S69). Moreover, comparison of two samples, both aggregated by the presence of NaI, shows that higher dye concentrations lead to shorter lifetimes (Supplementary Fig. S70).
As suggested by the referee, the revised manuscript also includes the analysis of the multiexponential fluorescence decay as tables in the revised Supplementary Figs. S66-S68.

Revision: We added this lines to the main text: "Increasing the concentration of NaI from 0.0 to 0.6 mM led to a progressive decrease in τ_{avg}, from 3.7 to 2.6 ns (Supplementary Fig. S68), which we ascribe to a combination of triplet formation (see below) and singlet-singlet annihilation (Supplementary Figs. S69-S70).[53] "

We included these results in the Supplementary Information:

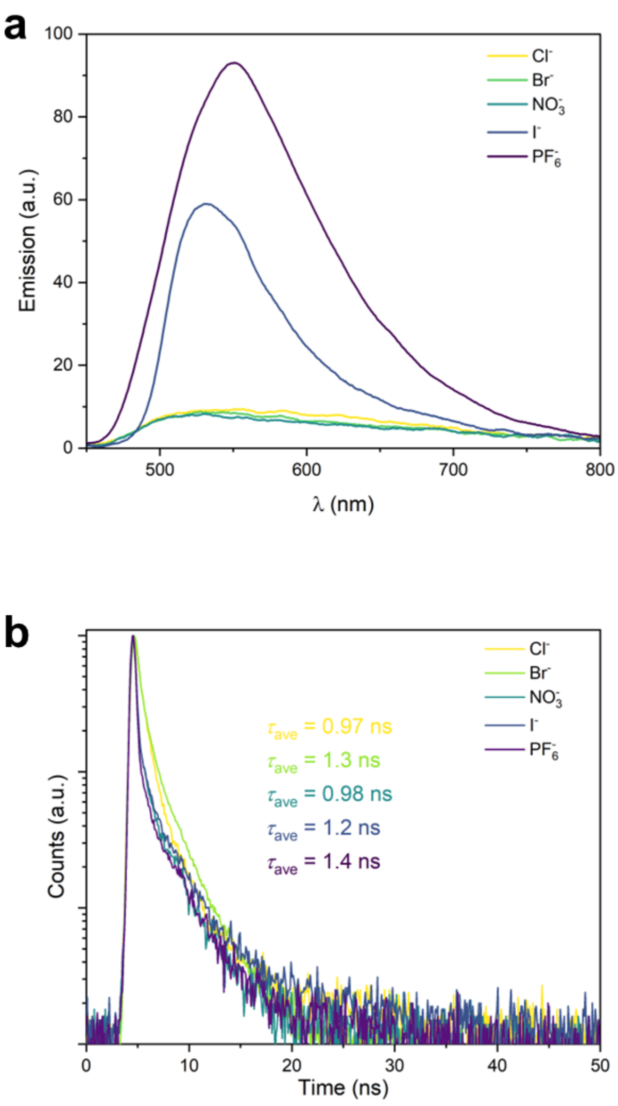

c

anions	τ_1	τ_2	τ_3
Cl^-	0.09	1.1	3.8
Br^-	0.09	1.3	4.9
NO_3^-	0.06	1.0	3.6
I^-	0.25	0.96	4.8
PF_6^-	0.13	1.2	4.1

Figure S21. a) Emission spectra of DSAX (0.3 mM) recorded with an integrating sphere in acetate buffer (0.1 M, pH = 5) containing 3.2 % DMSO (*v*/*v*). Fluorescence quantum yields (Φ_{FL}) increase from ≤1.0% (for Cl^-, Br^-, and NO_3^- salts) to 2.7% and 8.7% for DSAI and $DSAPF_6$, respectively. b) Time-correlated single-photon counting (TCSPC) decay traces of the same samples (λ_{exc} = 402.6 nm, λ_{em} = 520 nm). c) multiexponential fluorescence decay components.

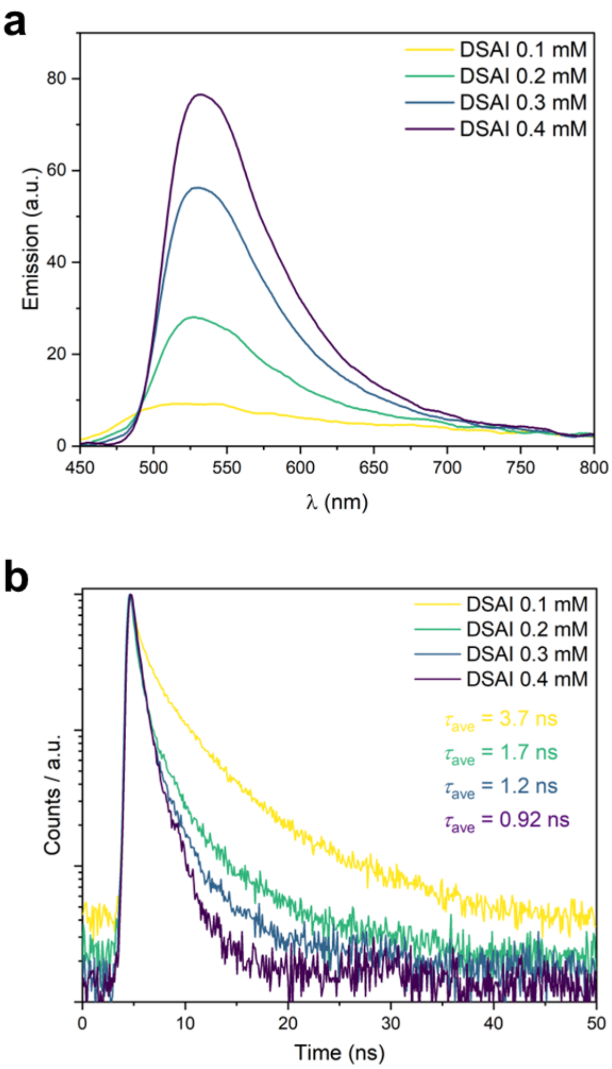

c

DSAI	τ_1	τ_2	τ_3
0.1 mM	0.25	1.8	6.4
0.2 mM	0.23	1.1	5.4
0.3 mM	0.25	0.96	4.8
0.4 mM	0.43	1.1	4.5

Figure S22. a) Emission spectra registered with integrating sphere of different concentrations of DSAI in acetate buffer (0.1 M, pH = 5) containing 3.2 % DMSO (*v*/*v*). Fluorescence quantum yields (Φ_{FL}) from ≤1% for 0.1 mM to 4.8% for 0.4 mM. DSAI 0.2 and 0.3 mM showed Φ_{FL} of 2.0 and 3.6%, respectively. b) Time-correlated single-photon counting (TCSPC) measurements (λ_{exc} = 402.6 nm, λ_{em} = 520 nm) of same samples. c) multiexponential fluorescence decay components.

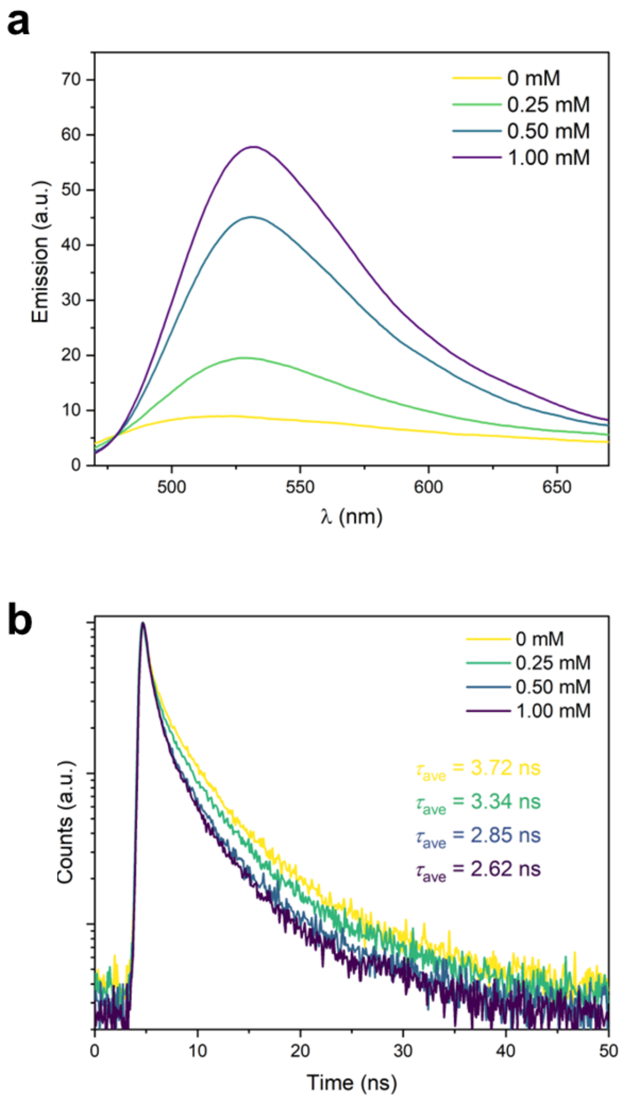

c

NaI	τ_1	τ_2	τ_3
0 mM	0.30	1.9	6.5
0.25 mM	0.28	1.8	6.4
0.5 mM	0.46	1.9	6.6
1.0 mM	0.44	1.8	6.3

Figure S23. a) Emission spectra registered with integrating sphere of DSAI 0.1 mM in acetate buffer (0.1 M, pH = 5.0) containing 3.2 % DMSO (*v*/*v*) with different concentrations of NaI. Fluorescence quantum yields (Φ_{FL}) from 1% for NaI 0.0 mM to 4.3% for NaI 1.0 mM. DSAI 0.1 with NaI 0.25 and 0.4 mM showed Φ_{FL} of 1.2 and 3.4%, respectively. b) Time-correlated single-photon counting (TCSPC) measurements (λ_{exc} = 402.6 nm, λ_{em} = 520 nm) of same samples. c) multiexponential fluorescence decay components.

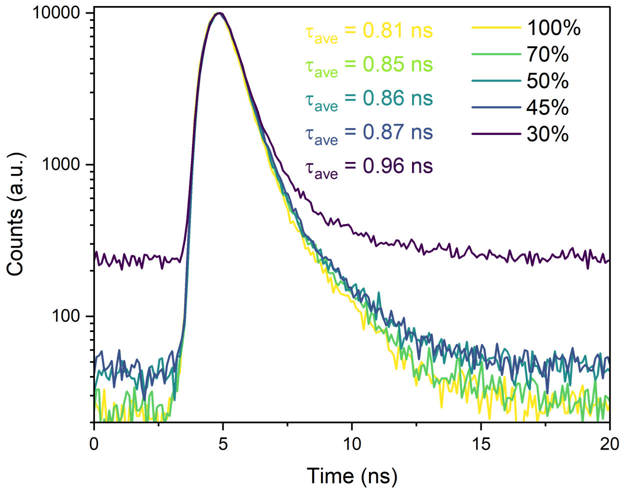

Figure S69. Time-correlated single-photon counting (TCSPC) measurements (λ_{exc} = 402.6 nm, λ_{em} = 520 nm) of DSAI 0.1 mM with NaI 1 mM by tuning the intensity of the exciting laser.

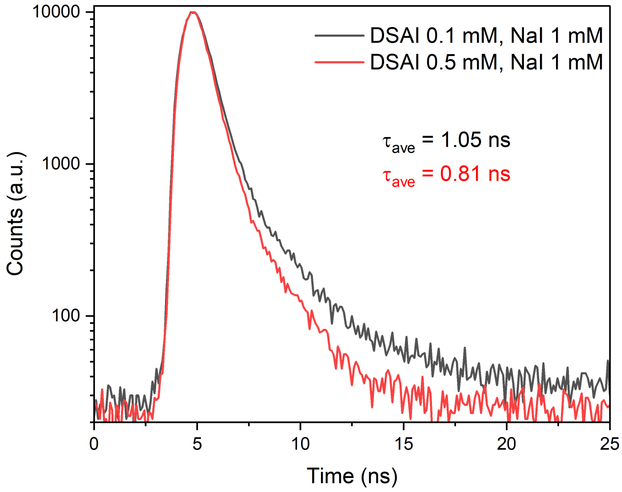

Figure S7024. Time-correlated single-photon counting (TCSPC) measurements (λ_{exc} = 402.6 nm, λ_{em} = 520 nm) of DSAI 0.1 mM or 0.5 mM with NaI 1 mM.

Comment: 7. *(Page 24 conclusion part): The primary conclusion of this study is that the observed aggregation-induced photocatalysis is based on localized excited-state reactivity, rather than extended electronic coupling between the arranged dyes. A thorough examination, however, of the crystal structure of DSAI aggregates discloses a centre-to-centre distance between dye rings of 5.67 Å with a slip angle of θslip = 44.6(0). This molecular arrangement exhibits characteristics consistent with slip-stacked packing featuring mixed electronic states, in which J-type exciton coupling predominates - resulting in increased emission. Therefore, the possibility of mixed electronic states involving both coulombic and charge transfer coupling cannot be ruled out. To ensure the validity of the primary conclusion of this study, it is essential to investigate whether DSAI's photoinduced radical*

signatures can be directly detected using Transient Absorption Spectroscopy, as these signatures may contribute to or be responsible for triggering the photocatalytic reactions presented.

Answer: We thank the reviewer for this insightful comment. We agree that the slip-stacked packing observed for DSAI aggregates ($\theta_{slip} \approx 44.6°$) could be consistent with *J*-type excitonic coupling. However, there are several arguments against the formation of *J*-aggregates, which collectively point to restricted intramolecular motion as the primary reason to enhancement of excitation and photocatalysis in the aggregated states:

i. Three-dimensional electron diffraction data of the DSAI aggregate showed there is quite large center-to-center distance of 5.67 Å and this would indicate weak or no intermolecular coupling, compared to usually observed π–π distance of about 3.35 Å in slip-stacked *J*-aggregates.[15]
ii. There is no apparent excitonic coupling between solution and aggregates both at the ground state and the excited state. In case of formation of *H*- or *J*-aggregates we would expect the absorption and emission profiles to exhibit bathochromic or hypsochromic shifts.
iii. As discussed in replies to Reviewers 2 and 3, we also performed transient absorption spectroscopy on DSAI aggregates. The TAS spectra closely resemble those reported for monomeric unsubstituted 9,10-distyrylantracene in rigid matrices, as described in the cited DSA study,[7] displaying ground-state bleach and excited-state absorption features characteristic of localized singlet (and subsequently triplet) excited states, with no detectable signatures of long-lived intermolecular radical ion pairs. These results confirm that photoexcitation does not generate persistent charge-separated states in the aggregates. Rather, aggregation stabilizes localized excited states by rigidifying the chromophore framework, thereby increasing their lifetime and population available for productive electron transfer to external acceptors.
iv. Previous work on the parent unsubstituted 9,10-distyrylantracene identified suppression of intramolecular motion as the primary origin of the enhanced fluorescence.[7] As mentioned above, our DSAI aggregate signature matches the TAS profiles recorded for monomeric DSA in rigid matrices (PMMA), thus further validating the role of self-assembly in restricting intramolecular motion enabling excited state accessibility.[7] It should be noted that, while restricted intramolecular motion is the dominant enabling mechanism, intermolecular stacking was proposed to further suppress non-radiative deactivation.[7]
v. To further prove that there are no or weak intermolecular coupling, we investigated the emission behavior of DSAI dispersed within sodium alginate. We found that it is comparable with that of the aggregate (λ_{max} = 525 nm), pointing to emission enhancement due restricted intramolecular motion rather than that to *J*-aggregates formation during assembly.

Taken together, while we do not exclude the presence of weak interactions inherent to slip-stacked packing, both our TAS data and the established photophysics of DSA demonstrate that photocatalysis in this system does not rely on extended exciton delocalization or intermolecular charge-transfer states. Instead, it is governed by localized excited-state reactivity enabled by aggregation-induced rigidification, fully supporting the primary conclusion of the manuscript.

Revision: We have added this discussion regarding the possibility of intermolecular coupling in slip-stacked aggregates: "While we do not exclude the presence of weak coupling interactions inherent to slip-stacked molecules, the quite large π-π stacking distances (>5 Å), lack of chromic shifts between solutions and aggregates, in addition to pump-probe spectroscopy demonstrate that DSAI aggregates operate with singlet and triplet excited states."
In the new section discussing transient absorption spectroscopy, we also added a discussion in which the TAS signatures of DSAI are compared to those of unsubstituted 9,10-distyrylantracene in rigid matrices: "To assess how supramolecular aggregation affects the excited states, we performed time-correlated single photon counting (TCSPC) to measure fluorescence lifetimes (τ_{avg}) under the different aggregation conditions. Consistent with the observed reverse Hofmeister effect in emission enhancement, counterion-dependent experiments revealed that charge diffuse ions anions extended

the singlet-state lifetime, with the τ_{avg} increasing from 0.97 ns for Cl^- to 1.4 ns for PF_6^- (Supplementary Fig. S66). Increasing the DSAI concentration from 0.1 to 0.4 mM led to a decrease of τ_{avg} from 3.7 to 0.92 ns (Supplementary Fig. S67). Increasing the concentration of NaI from 0.0 to 0.6 mM led to a progressive decrease in τ_{avg}, from 3.7 to 2.6 ns (Supplementary Fig. S68), which we ascribe to a combination of triplet formation (see below) and singlet-singlet annihilation (Supplementary Figs. S69-S70).[53] In all cases, the fluorescence decays show lifetimes in the nanosecond range, pointing to formation of singlet excited states. This prompted us to further probe the excited states of DSAI solution and aggregates through femtosecond and nanosecond transient absorption (fs-TAS and ns-TAS, respectively) experiments. The monomeric chromophore in solution displayed a band peaked at 620 nm that shifts at 545 nm and then disappeared with lifetimes of 17 ps and 79 ps, respectively, attributed to fast isomerization of the styryl moieties of the DSAI (Supplementary Fig. S71). These results are in agreement with fs-TAS spectra reported for unsubstituted 9,10-distyrylanthracene and derivatives.[54] For DSAI aggregates, instead, fs-TAS showed significative differences: no shift of the excited state absorption (ESA) bands were observed and a kinetic analysis shows a fast component of 21 ps, similar to that previously observed for the monomeric species, but the second component is slower with 320 ps lifetime, as expected by the rigidification of the structure that slows down decays related to photoisomerization pathways and enhances excited state stabilization (Supplementary Fig. S72). At the end of the time range of measurement (7 ns) an ESA band peaked at 539 nm persisted, compatible with the formation of the lowest triplet excited state, whose presence was confirmed in the ns-TAS experiment (Supplementary Fig. S73), although no phosphorescence was observed in solid state measurements (at 77 K, Supplementary Fig. S74). Importantly, the emission and transient absorption spectra of DSAI aggregates mirror those reported for the monomeric dye in rigid environments (Supplementary Fig. S75),[54] further supporting the formation of localized excited states – both singlet and triplet – in the aggregated state. These results show that aggregation enhances photocatalysis by increasing the population of localized excited states. The fact that three distinct aggregation triggers – counterion identity, concentration, and ionic strength – produce parallel trends in both emission and ROS generation supports the observation in which aggregation restricts intramolecular motion and increases the probability of productive excited-state processes."

Comment: *8. The authors posit that aggregation has no effect on excited-state energies or redox potentials, and that dyes behave independently of each other (i.e. there is no coupling), operating through localized singlet excited states stabilized by rigidification. In other words, the only beneficial function of aggregation is the rigidification of the dye framework, imposed by the packing forces. If this is true, it may be possible to hypothesize that a synthetic modification that renders the monomer (e.g., DSAI) more planar would maintain its aggregation-induced photocatalytic performance without requiring aggregation. In my opinion, this question doesn't need to be fully explained at this moment, but it deserves to be discussed.*

Answer: We thank the referee for their comment. As they suggested, aggregation-induced emission could be achieved also thanks to covalent rigidification of the monomer. One example is given by TPE 3D covalent organic frameworks, in which the rigid skeleton can restrict the relaxation of TPE units, enhancing PLQY.[16] Similarly, blue-emitters macrocycles based on triphenylamine and carbazole units were designed by introducing an *o*-methyl group to increase the torsion angle between the phenyl ring and the carbazole unit. This highly twisted conformation favors the solid-state emission.[17] Another tool could be the construction of covalent organic cages[18] as well as the incorporation of AIE-active fluorophore in a well-defined framework or macrocycle.[19]

Revision: We added this line to the discussion in our revised manuscript: "In our system, the mechanistic decoupling of supramolecular structure from excited-state energetics simplifies rational design and predictive control. In this respect, aggregation-induced rigidification provides a supramolecular route that complements established covalent strategies for chromophore

rigidification, including sterically constraining structures,[69] or preparing covalent organic frameworks, macrocycles, molecular cages and organic-inorganic superlattices.[17,42,70–72]"

Comment: *9. Page 1 last paragraph: Remove "that" from "confirming that the generality…"*

Answer: We thank the reviewer for this comment.
Revision: The manuscript has been updated accordingly.

Comment: *10. Figure 5d caption: Add "in the presence of co-catalyst PtNPs".*

Answer: We thank the reviewer for the suggestion and apologize for the omission.
Revision: The manuscript has been updated accordingly.

Comment: *11. References: appropriate credit to previous work? Yes*

Answer: We thank the reviewer for this positive assessment of the referencing and contextualization of prior work.

Comment: *12. Clarity and context: lucidity of abstract/summary, appropriateness of abstract, introduction and conclusions. Yes*

Answer: We thank the reviewer for the positive evaluation of the clarity, structure, and contextual framing of the manuscript.

Rebuttal References

1. Lancel, M., Lindgren, M., Monnereau, C. & Amara, Z. Kinetic effects in singlet oxygen mediated oxidations by immobilized photosensitizers on silica. *Photochem. Photobiol. Sci.* **23**, 79–92 (2024).

2. Samperi, M., Limón, D., Amabilino, D. B. & Pérez-García, L. Enhancing Singlet Oxygen Generation by Self-Assembly of a Porphyrin Entrapped in Supramolecular Fibers. *Cell Rep. Phys. Sci.* **1**, 100030 (2020).

3. Liu, X. *et al.* Efficient Quasi-Homogenous Photocatalysis Enabled by Molecular Nanophotocatalysts with Donor-Acceptor Motif. *Adv. Mater.* **37**, 2413440 (2025).

4. Kazantsev, R. V. *et al.* Crystal-Phase Transitions and Photocatalysis in Supramolecular Scaffolds. *J. Am. Chem. Soc.* **139**, 6120–6127 (2017).

5. McDowall, D. *et al.* Controlling Photocatalytic Activity by Self-Assembly – Tuning Perylene Bisimide Photocatalysts for the Hydrogen Evolution Reaction. *Adv. Energy Mater.* **10**, 2002469 (2020).

6. Yang, H. *et al.* Packing-induced selectivity switching in molecular nanoparticle photocatalysts for hydrogen and hydrogen peroxide production. *Nat. Nanotechnol.* **18**, 307–315 (2023).

7. Zhang, J. *et al.* Insights into the origin of aggregation enhanced emission of 9,10-distyrylanthracene derivatives. *Mater. Chem. Front.* **1**, 1422–1429 (2017).

8. Buzzetti, L., Crisenza, G. E. M. & Melchiorre, P. Mechanistic Studies in Photocatalysis. *Angew. Chem. Int. Ed.* **58**, 3730–3747 (2019).

9. Masnovi, J. M., Seddon, E. A. & Kochi, J. K. Electron transfer from anthracenes. Comparison of photoionization, charge-transfer excitation and electrochemical oxidation. *Can. J. Chem.* **62**, 2552–2559 (1984).

10. Schmidt, D. *et al.* Protein-like Enwrapped Perylene Bisimide Chromophore as a Bright Microcrystalline Emitter Material. *Angew. Chem. Int. Ed.* **58**, 13385–13389 (2019).

11. Almuhana, A. R. Y. *et al.* Retention of perylene diimide optical properties in solid-state materials through tethering to nanodiamonds. *J. Mater. Chem. C* **9**, 10317–10323 (2021).

12. Heckelmann, I. *et al.* Supramolecular Self-Assembly as a Tool To Preserve the Electronic Purity of Perylene Diimide Chromophores**. *Angew. Chem. Int. Ed.* **62**, e202216729 (2023).

13. Benson, C. R. *et al.* Plug-and-Play Optical Materials from Fluorescent Dyes and Macrocycles. *Chem* **6**, 1978–1997 (2020).

14. Cappelletti, D., Barbieri, M., Aliprandi, A., Maggini, M. & Đorđević, L. Self-assembled π-conjugated chromophores: preparation of one- and two-dimensional nanostructures and their use in photocatalysis. *Nanoscale* **16**, 9153–9168 (2024).

15. Kim, J. H., Schembri, T., Bialas, D., Stolte, M. & Würthner, F. Slip-Stacked J-Aggregate Materials for Organic Solar Cells and Photodetectors. *Adv. Mater.* **34**, 2104678 (2022).

16. Ding, H. *et al.* An AIEgen-based 3D covalent organic framework for white light-emitting diodes. *Nat. Commun.* **9**, 5234 (2018).

17. Yang, J.-C. *et al.* Novel supramolecular artificial light-harvesting systems based on AIE-active macrocycles for efficient white-light photocatalysis in water. *Chem. Sci.* **16**, 4741–4748 (2025).

18. Qin, Y. *et al.* Construction of Covalent Organic Cages with Aggregation-Induced Emission Characteristics from Metallacages for Mimicking Light-Harvesting Antenna. *Angew. Chem. Int. Ed.* **62**, e202308210 (2023).

19. Guo, C.-S. *et al.* Tetraphenylethylene tricycle-based sequential light-harvesting system through efficient Förster resonance energy transfer for visible light photocatalysis. *Sci. China Chem.* (2025) doi:10.1007/s11426-025-2780-x.

Re: NCHEM-25093064C
Point-by-point response to the reviewer's comments

Reviewer #1:

After reading thoroughly the thorough and extensive replies to the referees and the modified manuscript, the paper states more clearly the novelty, and has provided additional evidence to allay doubt concerning certain aspects of the work. The research seems robust, valid and reliable. All criticisms to the original submission have been addressed.

Answer: We sincerely thank the reviewer for the positive reassessment and for recognizing that the revised manuscript addresses all previous concerns and clearly establishes the novelty and robustness of the work.

Reviewer #2:

The authors have adequately revised their manuscript according to my previous comments and suggestions. The quality of the manuscript has been improved after the revision. The revised manuscript is recommended for publication in its present form.

Answer: We thank the reviewer for the constructive feedback and for recommending the revised manuscript for publication in its present form.

Reviewer #3:

The authors have made a substantial effort to address the concerns raised in the previous review and have significantly expanded the experimental and mechanistic scope of the manuscript. In particular, the inclusion of excited-state redox energetics, transient absorption spectroscopy (TAS), wavelength-dependent photocatalysis, and additional aggregation-control experiments has strengthened the mechanistic framework of aggregation-induced photocatalysis (AIP). Nevertheless, while many of the reviewer's questions are now addressed at a qualitative level, several key points require further refinement to confirm the mechanistic robustness and conceptual consolidation of the work.
I therefore recommend minor revision before the manuscript can be considered for publication in Nature Chemistry:

Answer: We sincerely thank the reviewer for taking the time to review our manuscript again and for providing positive feedback. We are grateful to the reviewer for raising additional concerns, which have guided us to further improve the manuscript.

Comment 1: While the authors now attribute the apparent decoupling between fluorescence lifetime and photocatalytic activity to singlet–singlet annihilation and triplet-state participation, the mechanistic picture remains largely qualitative. It would be important to clarify, at least semi-quantitatively, which excited state (singlet vs triplet) dominates the productive electron-transfer step under catalytic conditions, and how this correlates with the observed reaction rates.

Answer: We thank the referee for their comment. To determine which excited state (either singlet or triplet) dominates in photocatalytic conditions we refer to the Stern-Volmer analysis obtained by quenching experiments of DSAI aggregates in presence of increasing concentrations of methyl viologen (Fig. 5h,i in the revised manuscript). This analysis reveals that the singlet pathway dominates over the triplet (by approximately a factor of seven), with quenching constant of k_{sv}^{S} = 222 M^{-1} and k_{SV}^{T} = 32 M^{-1} respectively. To point out this observation, we have modified the main text accordingly.

Revision: We have rewritten the following section, found towards the end of the section dealing with photocatalytic H_2 production in the main text. "Fluorescence experiments revealed that salt-induced aggregation leads to a marked increase in emission with Φ_{FL} going from 0.8% to 14% (Fig. 5g and Supplementary Fig. S102 for excitation-emission maps), while ns-TAS showed the emergence of a triplet state in the aggregated material (Fig. 5h). To quantitatively determine which excited state (singlet versus triplet) dominates the productive electron-transfer step under catalytic conditions, we performed quenching experiments (Fig. 5h,i and Supplementary Fig. S103). These experiments reveled that both the excited states of the aggregated DSAI are quenched in the presence increasing concentration of the acceptor MV^{2+}, with the singlet (k_{SV}^{S} = 222 M^{-1} from fluorescence titration) prevailing over the triplet state (k_{SV}^{T} = 32 M^{-1} from ns-TAS titration)."

The following sentence was modified, in the Discussion section of the main text: "In this way, both singlet- and triplet-derived excited-state pathways can contribute to photocatalysis (with singlet excited states being prevalent under the present case), a concept that has been proposed for efficient solar-driven chemical transformations."

Comment 2: The authors justified the focus on DSAI due to its concentration-dependent assembly. However, to establish AIP as a general design principle, it is important to clarify if the mechanistic conclusions derived from salt-induced DSAI aggregates (kinetic traps) are fully transferable to

spontaneously aggregating systems like DSAPF6. A brief comparative discussion on whether the "excited-state confinement" mechanism remains consistent across different counterion-induced packing motifs would be beneficial.

Answer: We thank the referee for their suggestion. In order to highlight the generality of AIP observations, confirmed by photocatalytic experiments with different anions, we have included in the conclusion as brief comparative discussion the results obtained also for spontaneously aggregating systems like $DSAPF_6$.

Revision: We have revised the following section in the Discussion part of the main text. **"**These observations emphasize the critical role of counterion identity not only in solubility, but as a tunable design element for supramolecular photocatalysis. Notably, the AIP observation remains consistent across aggregates with different anions (PF_6^- and I^-) both for reactive oxygen species production and H_2 evolution, manifesting that excited state stabilization remains coherent also across different counterion-induced packing motifs."

Comment 3: The contrast between the poor redox reversibility in cyclic voltammetry and the high photocatalytic activity remains a point of curiosity. While the authors argue that sacrificial donors "save" the oxidized chromophore, this suggests the system's robustness is highly dependent on donor kinetics. The authors should discuss whether the structural reorganization or degradation pathways inferred from the CV results could limit the catalyst's long-term recyclability in more demanding or donor-limited environments.

Answer: We thank the reviewer for their comment. In the revised manuscript, we underline the necessity of sacrificial donors or acid-assisted stabilization of radical intermediates – as reported for anthracene derivatives[1] – to ensure photostability under photocatalytic conditions.

Revision: Importantly, this operational photostability is in apparent contrast with the limited redox reversibility observed in cyclic voltammetry (Supplementary Figure S87), underscoring that electrochemical irreversibility primarily reflects slow electron-transfer kinetics under electrochemical conditions rather than intrinsic instability under photocatalytic turnover. Under photocatalytic operation, rapid donor-mediated quenching and acid-assisted stabilization of radical intermediates[61] most likely prevents side reactions. We note that in more demanding or donor-limited environments such stability may become less effective, potentially exposing structural reorganization or degradation pathways inferred from electrochemical measurements.

<u>Reviewer #4</u>:

In the revised manuscript, the authors provide thorough and convincing responses to the initial comments raised. I am therefore satisfied that the work meets the publication standards of Nature Chemistry in its current form.

Answer: We are grateful to the Reviewer for confirming that our revisions satisfactorily address the initial comments and that the manuscript now meets the journal's publication standards.

References

1. Masnovi, J. M., Seddon, E. A. & Kochi, J. K. Electron transfer from anthracenes. Comparison of photoionization, charge-transfer excitation and electrochemical oxidation. *Can. J. Chem.* **62**, 2552–2559 (1984).
